# Skin health of community-living older people: a scoping review

**DOI:** 10.1007/s00403-024-03059-0

**Published:** 2024-06-01

**Authors:** Jan Kottner, Alexandra Fastner, Dimitra-Aikaterini Lintzeri, Ulrike Blume-Peytavi, Christopher E. M. Griffiths

**Affiliations:** 1 Institute of Clinical Nursing Science, Charité Universitätsmedizin Berlin, Charitéplatz 1, 10117 Berlin, Germany; 2 Department of Dermatology, Venerology and Allergology, Charité Universitätsmedizin Berlin, Berlin, Germany; 3 Department of Dermatology, King’s College Hospital, King’s College London, London, UK; 4 Centre for Dermatology Research, NIHR Manchester Biomedical Research Centre, The University of Manchester, Manchester, UK

**Keywords:** Ageing, Dermatology, Care, Community, Skin

## Abstract

The population of older people is steadily increasing and the majority live at home. Although the home and community are the largest care settings worldwide, most of the evidence on dermatological care relates to secondary and tertiary care. The overall aims were to map the available evidence regarding the epidemiology and burden of the most frequent skin conditions and regarding effects of screening, risk assessment, diagnosis, prevention and treatment of the most frequent skin conditions in older people living in the community. A scoping review was conducted. MEDLINE, Embase and Epistemonikos were systematically searched for clinical practice guidelines, reviews and primary studies, as well as Grey Matters and EASY for grey literature published between January 2010 and March 2023. Records were screened and data of included studies extracted by two reviewers, independently. Results were summarised descriptively. In total, 97 publications were included. The vast majority described prevalence or incidence estimates. Ranges of age groups varied widely and unclear reporting was frequent. Sun-exposure and age-related skin conditions such as actinic keratoses, xerosis cutis, neoplasms and inflammatory diseases were the most frequent dermatoses identified, although melanoma and/or non-melanoma skin cancer were the skin conditions investigated most frequently. Evidence regarding the burden of skin conditions included self-reported skin symptoms and concerns, mortality, burden on the health system, and impact on quality of life. A minority of articles reported effects of screening, risk assessment, diagnosis, prevention and treatment, mainly regarding skin cancer. A high number of skin conditions and diseases affect older people living at home and in the community but evidence about the burden and effective prevention and treatment strategies is weak. Best practices of how to improve dermatological care in older people remain to be determined and there is a particular need for interventional studies to support and to improve skin health at home.

**Supplementary Information:**

The online version contains supplementary material available at 10.1007/s00403-024-03059-0.

## Introduction

The world´s population is ageing. By 2050, it is anticipated that approximately 16% of the global population will be aged 65 years and older [1]. This demographic shift highlights the increased demand for effective and sustainable healthcare and long-term care services.

The majority of older people live in the community [2]. Accordingly, the World Health Organization (WHO) emphasises the need to establish structures and processes that enable individuals to live and to receive care at home for as long as possible. In 2015, the WHO introduced a public health framework aiming at promoting ‘healthy ageing’ [3]. Development and maintenance of ‘functional ability’ is a central concept and the approach aims to delay, or partially reverse age-related processes that lead to functional decline and increased dependency [4].

Skin ageing is influenced by intrinsic and extrinsic factors including genetic predisposition, nutrition, sun damage, environmental conditions, and personal habits such as smoking [3, 5]. Age-related skin changes include thinning of the epidermis, flattening of the dermal-epidermal junction, and loss of subcutaneous fat, collagen, and elastin in the dermis. Functionally, a reduction of the skin’s viscoelastic properties and a reduced capacity for barrier function and repair following insult is observed [6, 7]. These alterations increase the susceptibility to a wide range of age-related skin problems such as xerosis cutis, pruritus, shear-type injuries, bullae formation and colonisation by pathogenic bacteria. Age-related skin conditions may have a negative impact on the quality of life and mental health of the individual, thus leading to an overall deterioration in health [6].

Chronic systemic conditions in older people including cardiovascular and renal diseases, diabetes mellitus (DM) and concomitant medications such as diuretics also affect the skin [8–10]. For example, patients with DM are at risk of fungal and bacterial infections, as well as foot ulceration [11]. Age-related reduced mobility, incontinence, and malnutrition contribute to a variety of skin diseases such as incontinence-associated dermatitis (IAD) [12] and pressure ulceration [10]. Thus, the increased risk of skin problems in older people results from a combination of various direct and indirect factors, pathways, and interactions [13].

Since 2017, the WHO provides evidence-based guidelines for detecting and managing declines in physical and mental capacities in older people, with a focus on healthcare providers in the community and primary healthcare settings [4]. While there is a substantial body of evidence supporting preventative and therapeutic strategies for skin health in secondary or tertiary care settings, a summary of empirical evidence in the community and primary care settings is missing.

This scoping review aims to examine the extent and nature of available evidence regarding skin diseases in the primary care setting. The following questions will be answered:

1. What are the most frequent skin conditions in older people living in the community?
2. What is the burden of skin conditions in older people in the community setting?
3. What type of evidence exists regarding the effects of screening, risk assessment, diagnosis, prevention and treatment of the most frequent skin conditions in older people living in the community?

## Methods

### Protocol and registration

A scoping review was conducted guided by the Preferred Reporting Items for Systematic Reviews and Meta-Analyses extension for scoping reviews [14] and a detailed review protocol was published before [13].

### Eligibility criteria

The following inclusion criteria were used: studies conducted in community or primary care settings; participants being 60 years or older living at home; reporting of numerators/denominators/time period; descriptive and interventional study designs; systematic reviews; clinical practice guidelines; and publication from January 2010 onwards. The lower age limit of 60 years was adhered to by including only those studies in which this age limit was the inclusion criterion or in which data for this age limit could be clearly extracted. The following exclusion criteria were applied: hospital; long-term care; secondary; tertiary care settings; opinion papers; editorials; and case reports. No language restrictions were applied.

**Fig. 1:**
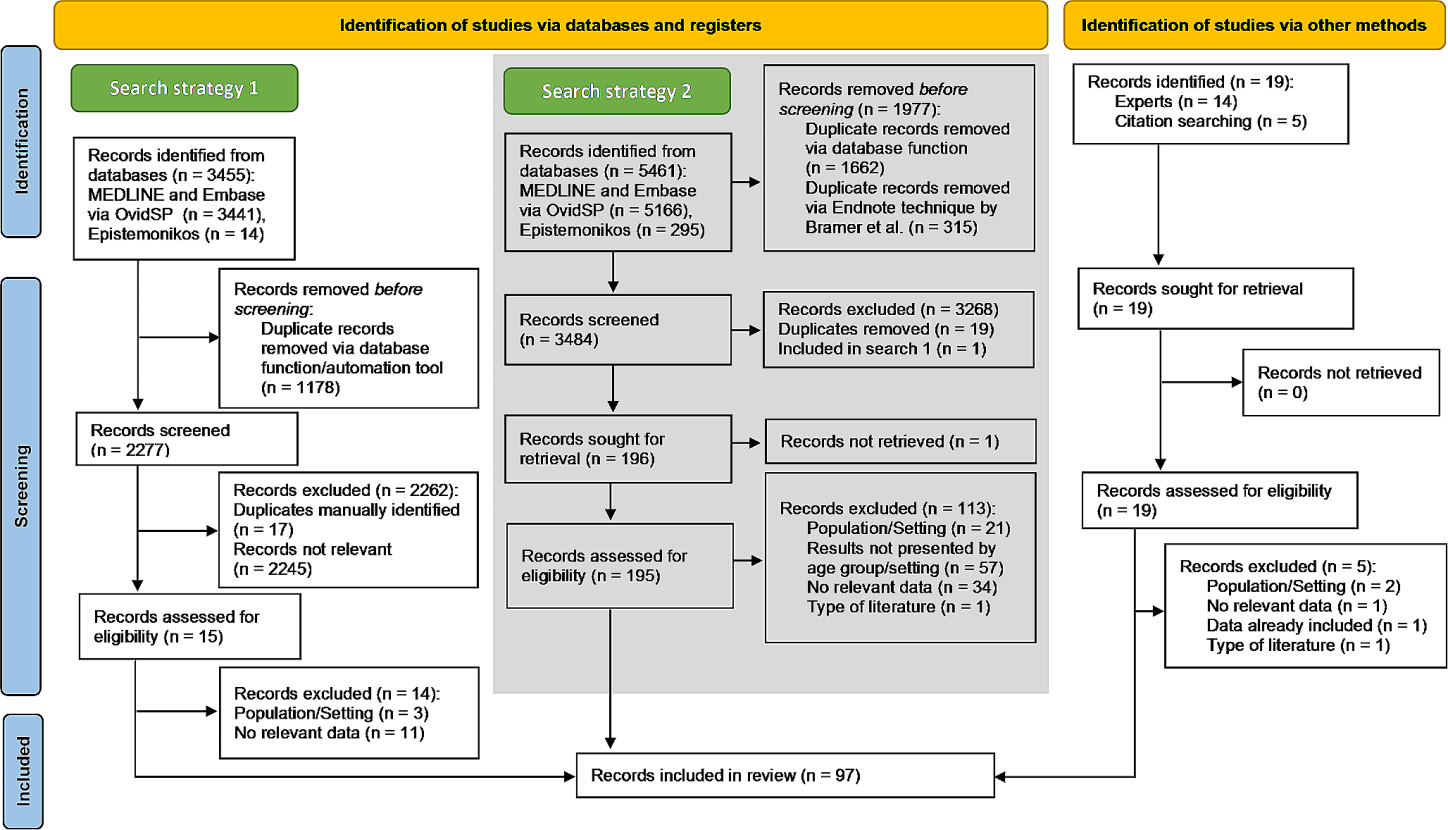
Flow chart of search and selection process

The original eligibility criteria outlined in the published protocol [13] included systematic and scoping reviews and clinical practice guidelines only and people 65 years and older (search strategy 1). These original eligibility criteria resulted in one reference only (Fig. 1). Therefore, the inclusion criteria were expanded to include people 60 years and older and various other study designs reporting empirical data (see above).

### Information sources

MEDLINE and Embase were searched via OvidSP, as well as Epistemonikos. Grey Matters [15, 16] and EASY datasets by DANS [17] were searched for grey literature. EASY was used instead of OpenGrey as it was archived with DANS [18]. Reference lists and experts in the field were consulted regarding relevant additional literature.

### Search

The second search strategy was conducted on 7 March 2023. Table 1 shows the search terms in MEDLINE and Embase via OvidSP. The same strategy was used for Epistemonikos. EASY and Grey Matters were searched using the terms ‘skin’, ‘community’ and ‘elderly’ as well as ‘older people’.

**Table 1:** Search results of concurrent searches in Embase and MEDLINE via OvidSP (search 2), conducted on 07 March 2023

#	Query	Results 7 Mar 2023
1	((Aged or elderly or geriatric or aging or ageing or older) and (Skin or dermatolog* or cutaneous*) and (condition* or disease* or problem* or health or screening* or assessment* or examination* or diagnos* or prevention or treatment* or management) and (epidemiolog* or prevalence or incidence or frequen* or burden) and (community-dwell* or “community dwell*” or community-living or “community living” or home-dwell* or “home dwell*” or home-nursing or “home nursing” or community-care or “community care” or home-care or “home care” or domesticity or “primary care” or community-based or “community based” or cross-sectional or cohort)).tw.	6,312
2	limit 4 to yr="2010 -Current”	5,166
3	remove duplicates from 5	3,504

### Selection of sources of evidence

Results of the searches were exported into EndNote. Titles and abstracts were independently screened by two reviewers (AF, DL) following the suggested method by Bramer, Milic and Mast [19]. Subsequently relevant results were exported to the free online software Rayyan, full-texts obtained and compared with the inclusion and exclusion criteria by both reviewers independently. Any discrepancies were discussed with a third reviewer (JK) if needed.

### Data charting process, data items and synthesis of results

AF and DL split the included records, extracted data using standardised extraction sheets and checked 80% of each other’s extractions for accuracy. In the case of discrepancies, JK was consulted. For each of the three review questions tables were developed to summarise characteristics and key results. Summarized results of the included reviews were extracted directly without referring to the underlying primary studies. No studies were included twice. Only the most recent estimates were extracted from cohort studies. A critical appraisal of the sources of evidence was not conducted.

## Results

### Selection of sources of evidence

The first search strategy yielded 3,455 citations. Of these 2,277 were screened, 15 assessed for eligibility, and finally one systematic review [20] included (Fig. 1). To increase the sensitivity the second search identified 5,461 potential records. After duplicates were removed, 3,484 citations were screened and 196 records sought for retrieval. One record could not be retrieved [21] and 113 were excluded. Most citations were excluded due to results not being presented for people ≥ 60 years or not separately for settings (*n* = 57), and data not being relevant (*n* = 46). Tables S1 and S2 (Supplementary material 1) list all excluded references with exclusion reasons from both searches. Additionally, 19 records were identified via experts and hand searching. Altogether, 97 records were included (Fig. 1). In total, 52 potential citations of grey literature were screened, of these 14 records were checked for eligibility. None met the inclusion criteria.

### Characteristics of sources of evidence

A detailed description of all 97 included publications can be found in Supplementary material 2. Table 2 presents the main characteristics: 84 publications were full-text articles and 13 were abstracts. Two abstracts referred to the same study [22, 23] and one abstract [24] referred to an included full-text article [25] indicating that in total 95 studies were included. Most studies were published after 2014 and in Europe (*n* = 40). The majority reported descriptive data. Multiple studies used data of the Rotterdam [26–28] and AugUR studies [29–31] for cross-sectional analyses. More than 50% of studies were based on registry or secondary data analyses and the reported ranges of age groups varied widely. A number of studies did not define ‘skin cancer’ [32–36].

**Table 2:** Years, geographic regions and types of included publications (*n* = 97)

Characteristics	*n* (%)
**Publication year** ^a^	
2010–2014	20 (20.6)
2015–2019	43 (44.3)
2020–2023	34 (35.1)
**Geographic region** ^b^	
Asia	22 (23.2)
Australia	5 (5.3)
Europe	40 (42.1)
North America	16 (16.8)
South America	6 (6.3)
Global	6 (6.3)
**Publication type** ^a^	
Conference abstracts (incl. poster)	13 (13.4)
Full-text articles	84 (86.6)
**Study type** ^b^	
Descriptive	92 (96.8)
Interventional	4 (4.2)
**Results** ^b^	
Review question 1 (epidemiology of skin conditions)	77 (81.1)
Review question 2 (burden of skin conditions)	30 (31.6)
Review question 3 (effects of screening, assessment, diagnosis, treatment)	14 (14.7)

^a^ total *n* = 97 records

^b^ total *n* = 95 studies

The majority of included articles (79 articles, 77 studies) described prevalence and/or incidence estimates (review question 1), 30 studies presented evidence regarding the burden of skin conditions including self-reported symptoms or quality of life (review question 2) and 14 studies reported effects of interventions (review question 3). Table S3 (Supplementary material 3) lists all included publications with their assigned review questions, study type, and geographic region.

### Results of individual sources of evidence

Table S4 (Supplementary material 4) describes the epidemiological evidence addressing review question 1. Table S5 (Supplementary material 4) summarises the scope of skin condition(s) addressed. Nine studies reported any skin condition in the sample [20, 37–44]. Eight studies investigated a selection of skin conditions/diseases [33, 34, 45–50] and 60 studies focused on a specific skin condition/disease or disease group (e.g. ‘eczema’) [22–32, 51–101].

The most often investigated skin condition was melanoma and/or non-melanoma skin cancer (*n* = 34) [22, 23, 26, 48–56, 60, 61, 63, 64, 66, 69, 71, 73, 75–80, 82–84, 86, 89, 93, 94, 97, 101], three studies did not specify ‘skin cancer’ [32–34]. Different types of ‘eczema’ (including atopic dermatitis) were included in eight studies [30, 31, 33, 34, 46, 65, 88, 91], pressure injuries [45, 68, 81, 85, 90, 99] and psoriasis [29, 33, 34, 57, 58, 100] each in six studies, and xerosis/ichthyosis in five studies [24, 25, 27, 45, 47, 95]. Other studies focused on vitiligo [33, 34, 47, 62] and parasites [33, 34, 47, 92], bullous pemphigoid [59, 87, 98], intertrigo [67, 74], herpes zoster [46, 70], seborrheic dermatitis [28], granuloma annulare [72], and Methicillin-resistant Staphylococcus aureus skin infections [96].

Table S6 (Supplementary material 5) presents details of included studies regarding review question 2. Most studies (*n* = 10) described self-reported skin concerns/complaints or symptoms such as pruritus [20, 34, 40, 42, 45, 65, 102–105]. Nine studies reported mortality [53, 63, 64, 71, 77, 79, 97, 98, 106] and seven studies dermatology-related medical visits [37, 42, 43, 107–109] or hospital admissions [110]. One report described depressive disorders in individuals with and without ‘skin conditions’ [111], one a potential association between ‘skin problems’ and quality of life [103] and another the perception of whether skin conditions were bothersome [104]. Two studies each reported disability adjusted life years (DALY’s) [106, 112] and complications due to herpes zoster [70, 113].

Table S7 (Supplementary material 5) describes details of the 14 articles answering review question 3. Four studies applied interventional designs [38, 68, 99, 114]. Most articles (*n* = 7) focused on interventions regarding ‘skin cancer’ (melanoma, melanoma and non-melanoma skin cancer, not specified), four on the prevention (sun protection, awareness/education campaigns) [35, 36, 64, 115], two on screening [48, 60] and one referred to both [77]. Two articles looked into the prevention of pressure injuries, one investigating different pads in incontinent persons [99], and one introduced a pressure injury prevention protocol, including screening and risk assessment [68]. Further, two articles focused on herpes zoster, one tested a skin prick test for predicting the development of herpes zoster [114] and one the effectiveness of the vaccine in the UK [116]. One study reported on a teledermatology project in a major city in Brazil [38]. Another study sent trained community health workers to screen older people in a city in India for eczema. Suspected cases were then referred to a dermatologist [65]. Finally, one study investigated the most common types of treatments for actinic keratoses [61].

### Synthesis of results

Table 3 summarises the most frequent skin conditions (prevalence 5% or higher) identified. These include skin conditions typically related to sun-exposure and age such as androgenetic alopecia, actinic keratoses and xerosis cutis, as well as neoplasms and inflammatory diseases.

**Table 3:** Summary of most frequent skin conditions identified in this scoping review

Most frequent skin conditions	Range (number of reports)		Geographical regions
	Prevalence	Incidence	
Androgenetic alopecia (male and female pattern)	6.9–83.0 (2)	-	USA [42], Finland [37]
Seborrheic keratosis	13.9–78.8 (5)	-	Germany [44], France [49], India [40], Brazil [38], Finland [37]
Lentigo solaris	7.2–71.6 (4)	-	France [49], Finland [37], Germany [44], Brazil [38]
Other benign skin tumors (including cherry angioma, dermal naevi, dermatofibroma, melanocytic nevi etc.)	3.7–63.2 (5)	-	Germany [44], Finland [37], France [49], Poland [41], Brazil [38]
Actinic keratoses	3.1–69.4% (11)	28,788 /100,000 person-years (1) 7.2 to 14.0 /10,000 (2)	Ecuador [66], Finland [37], USA [50, 61], Germany [20, 39, 44], Netherlands [20, 26], France [49], Switzerland [78], Taiwan [89, 93], Brazil [38], China [54]
Fungal infections of the body	0.6–64.0 (10)	0.04% (1)	Singapore [33], India [34, 40], USA [20, 42], Poland [41], Japan, Tunisia, Iran, Sri Lanka [20], Germany [44], Finland [37], Netherlands [67, 74]
Xerosis cutis	3.3–60.0 (8)	-	France [20, 95], India [40], Germany [24, 25, 45] Netherlands [27], Brazil [38]
Tinea pedis, onychomycosis	4.0-48.6% (4)	25.7% (1)	Germany [44], Japan, USA, Belgium, Tunisia [20], Finland [37], Brazil [20, 38]
Pigmentary disorders (including idiopathic guttate hypomelanosis, vitiligo, not specified)	0.5–35.1 (9)	-	India [34, 40, 47], Brazil [38], Finland [37], Poland [41], Singapore [33], China [62], Germany [44]
Rosacea	0.9–33.7 (4)	-	USA [42], Germany^1, 11^, Finland [37]
Eczema/dermatitis (including atopic, contact, seborrheic, not specified)	0.3–30.6 (17)	-	Germany [30, 31, 39, 44], Poland [41], USA [42], India [34, 40, 65], UK [20, 88], Tunisia [20], Sweden [20], Japan [46], Finland [37, 91], Singapore [33], Brazil [38], Netherlands [28]
Scabies infection	0–20.0% (3)	-	Multiple [92], Singapore [33], India [34]
Psoriasis	0.5–14.8 (11)	39.9 to 45.9 /100,000 person-years (1)	USA [42], Poland [41], Finland [37], Germany [31, 39, 44], Singapore [33], Greenland [57], Malaysia [58], India [34], Norway [100]
Incontinence associated dermatitis	14.7 (1)	-	Germany [45]
Pressure ulcer/injury	0.5–12.0% (9)	3.0 to 6.7% (2)	UK [20], Germany [45], Indonesia [81], Italy [20, 99], South Korea [85], UAE [68], Singapore [33], India [34], Brazil [90]
Bacterial skin infections	0.2–13.8 (7)	627/100,000 people/year with mr - MRSA wound colonization/infection (1)	Singapore [33], Germany [44], India [34, 40], USA [42], Poland [41], New Zealand [96], Tunisia, Belgium [20]
Herpes zoster	9.3–11.1 (3)	10.2 to 14.3 /1,000 person-years (1)	Japan [46], USA [70], Poland [41]
Non-melanoma skin cancer (including basal cell and squamous cell carcinoma)	0.4–6.9% (6) Prevalence in AK group: 16.4% (1)	20 to 1300 /100,000 person-years (4) > 6000 / 100,000 person-years (1) 3.0-5.6/100 person-years (2)	France [49], Finland [37], USA [22, 23, 42, 75], Germany [39, 48, 55], Australia [86] UK [83], England [82], Netherlands [55, 69] Scotland [55], China [51]
Viral infections excluding Herpes zoster	0.4–6.8 (6)	-	USA [42], Tunisia [20], Singapore [33], India [34], Germany [44], Brazil [38]
Drug rash	5.1 (1)	-	Japan [46]

**Abbreviations**: Actinic keratosis (AK), (cutaneous) Squamous cell carcinoma (SCC), Non-melanoma skin cancer (NMSC)

Reports about burden included self-reported skin symptoms and concerns, quality of life, mortality, service use such as physician consultations (Table S6, Supplementary material 5). Pruritus was self-reported by up to 40.6% [20, 34, 40, 42, 45, 102, 104, 105, 117] (100% in individuals with eczema [65]). 15% [108] to 31.9% [43] of participants visited their General Practitioner because of their skin condition. One study reported 43% of skin conditions identified needed further care by a physician, 33% could be self-managed, and 24% needed no further treatment [37].

Most evidence in relation to the effects of screening, risk assessment and diagnosis, prevention and treatment of skin conditions had a descriptive design and analysed incidence and/or prevalence estimates in relation to prevention of and screening for ‘skin cancer’ (Table S7, Supplementary material 5). Two studies [68, 99] focusing on pressure injury prevention reported a reduction in pressure injury prevalence and/or incidence. One study describing a teledermatology project reported that 49.8% of patients were repatriated to their primary care physician and the most common prescriptions were ‘emollients’, ‘topical antifungal’, ‘sunscreen’ and ‘topical corticosteroids’ [38].

## Discussion

One of the main findings of this scoping review was the unexpectedly low number of studies presenting empirical evidence about the epidemiology, burden and treatment effectiveness in skin conditions and diseases of people living at home and in the community. Furthermore, most of the evidence includes prevalence and incidence estimates and very little is reported about intervention effects. Only 4 out of 95 reports used an interventional study design. These findings highlight a substantial research gap as the vast majority of older people receives community care in their own homes. As because the community is the largest care setting globally, it is of utmost importance to improve community care [4], including skin and dermatological care.

Regardless, this scoping review suggests that the most prevalent skin conditions and diseases in older community-living people are related to intrinsic and extrinsic skin ageing including androgenic alopecia, seborrheic and actinic keratoses, lentigo, benign skin tumours and xerosis cutis [6, 13, 118]. Further, inflammatory skin diseases and fungal skin infections were reported as highly prevalent, which is also associated with skin ageing [119]. However, the vast majority of studies reported on melanoma and non-melanoma skin cancer indicating that there is a much stronger research interest in skin cancer compared to other more frequent diseases. This indicates, that life-limiting diseases may generate higher research interest than improving the quality of life in commoner skin conditions.

Several studies addressed the burden of skin-related concerns and complaints, particularly pruritus, which is a common symptom in older people living at home [120]. The burden of dermatology-related medical visits was also investigated in a number of reports; however, it was often not possible to distinguish between primary care visits and outpatient contacts. Nevertheless, the results suggest that skin-related problems play a large role in seeking health care in community-living older people.

The majority of reports focusing on the effects of interventions described prevention of and screening for ‘skin cancer’. This included screening programmes, prevention campaigns, sun-protection behaviour, or skin cancer knowledge/awareness. One study reported the effects of a teledermatology project in which half of the patients were referred to their primary care physician and the most common prescriptions included self-treatment such as emollients and physician-administered treatments such as topical antifungals, which are applied at home by the individuals themselves or by caregivers [38]. These results suggest that a large proportion may not require specialist dermatological care.

Considering the high and increasing prevalence of the variety of skin conditions and diseases in older people, it seems unlikely that care provided by dermatologists will be feasible either now or in the future for everyone in this population and setting [121]. This relates to questions about who should be responsible for the diagnosis and management of skin conditions in the community. Proposals about possible skin condition categories and care strategies in older people have been made [37, 122]. For example, there appears to be a group of skin conditions that might not have pathological relevance and might not require further treatment (e.g. androgenetic alopecia). Other skin conditions might be manageable either by self-treatment or by other health professionals such as nurses or medical assistants (e.g. xerosis cutis) [122]. At the same time, no need for medical treatment does not mean that the condition does not cause distress or discomfort [123, 124]. The results of our scoping review clearly suggest that the most frequent skin conditions in community-living older people fall into these two broad categories. Nevertheless, there are important diseases, which should be diagnosed and treated by dermatologists (e.g. skin cancer). Approaches such as teledermatology could play an important role in meeting this increasing demand which is in line with the WHO guideline on self-care which promotes self-management, self-screening and self-awareness with the help of health technology, as well as trained healthcare workers facilitating access to such and supporting individuals [125].

Our review found that most research had its origin in Europe, North America and Asia, with the majority providing data on high to high-middle sociodemographic index countries. No study from the continent of Africa was included, only Hahnel et al. [20] included epidemiological data from Tunisia in their review.

The results further indicate that there is a lack of clinical practice guidelines regarding the most frequent skin conditions in older people in community settings. Screened guidelines and Best Practice Recommendations did not include specific recommendations for older people living at home. One might argue that the recommendations of clinical practice guidelines are applicable to all settings and age groups, and that guidelines for community settings are not needed. However, there is a clear need to emphasise basic self-care interventions in older adults such as regular screening or when to seek professional help. At the same time our review results also show, that evidence about the prevention and treatment of major skin conditions in older adults is largely missing [126].

### Limitations

We searched in the bibliographic databases MEDLINE, Embase and Epistemonikos, which can be considered most important for our review questions and inclusion criteria and we focussed on current evidence from the last 12 years and ignored older publications. Additional searches in other smaller databases and extending the search period might have led to further hits but overall our results reflect the overall availability of evidence. To keep the number of hits manageable, we only use free text words for the electronic search. Furthermore, as described by Haw, Al-Janabi [127] guidelines may have been missed because a notable number of local guidelines can only be found on websites and are not indexed in electronic library databases. Because this was a scoping review, a risk of bias assessment was not conducted, and the quality of evidence was not evaluated. However, study reporting usually did not follow state-of-the-art methods and there was a substantial heterogeneity of methods and outcome assessments. Although data extraction was performed with the utmost accuracy, some data may be inaccurate due to unclear reporting.

## Conclusions

Older community-living people are affected by a high number of skin conditions and diseases, but the current evidence about the burden and effective prevention and treatment strategies is weak. Best practices on how to improve dermatological care in this expanding population are to be determined and there is a particular need for interventional studies to support and to improve skin health at home.

### Electronic supplementary material

Below is the link to the electronic supplementary material.

Supplementary Material 1

Supplementary Material 2

Supplementary Material 3

Supplementary Material 4

Supplementary Material 5

## Data Availability

No datasets were generated or analysed during the current study.
