# Supplementary material for: Skin health of community-living older people: a scoping review
Source: Arch Dermatol Res. 2024 Jun 1;316(6):319. doi: 10.1007/s00403-024-03059-0 (PMC11144137; doi:10.1007/s00403-024-03059-0)
Supplement: Supplementary file 1 — Supplementary Material 1 [file 403_2024_3059_MOESM1_ESM.pdf]

Supplementary material 1

Article title: Skin health of community-living older people: a scoping review

Authors: Jan Kottner[1], Alexandra Fastner[1], Dimitra-Aikaterini Lintzeri[2], Ulrike Blume-Peytavi[2], Christopher E. M. Griffiths[3,4]

Institutions

[1]Institute of Clinical Nursing Science, Charité Universitätsmedizin Berlin, Berlin, Germany.

[2]Department of Dermatology, Venerology and Allergology, Charité Universitätsmedizin Berlin, Berlin, Germany.

[3]Department of Dermatology, King's College Hospital, King's College London, London, UK.

[4]Centre for Dermatology Research, NIHR Manchester Biomedical Research Centre, The University of Manchester, Manchester, UK.

Corresponding author

Jan Kottner
Charité – Universitätsmedizin Berlin,
Institute of Clinical Nursing Science,
Charitéplatz 1,
10117 Berlin, Germany
Email: jan.kottner@charite.de

Table S1: Excluded records from search 1 with exclusion reasons.

No.	Reference	Exclusion reason
1	Arora M, Harvey LA, Glinsky JV et al. Electrical stimulation for treating pressure ulcers. *Cochrane Database Syst Rev* 2020; **1**:CD012196. PMID: 31962369. doi: 10.1002/14651858.CD012196.pub2.	No relevant data
2	Briggs R, McDonough A, Ellis G et al. Comprehensive Geriatric Assessment for community-dwelling, high-risk, frail, older people. *Cochrane Database Syst Rev* 2022; **5**:CD012705. PMID: 35521829. doi: 10.1002/14651858.CD012705.pub2.	No relevant data
3	Frost H, Haw S, Frank J. Interventions in community settings that prevent or delay disablement and promote healthy ageing in later life [abstract]. *Age and Ageing* 2011; **40:**ii28. doi: 10.1093/ageing/afr099	No relevant data
4	Golding-Day M, Whitehead P, Radford K, Walker M. Interventions to reduce dependency in bathing in community dwelling older adults: a systematic review. *Syst Rev* 2017; **6**:198. PMID: 29020974. doi: 10.1186/s13643-017-0586-4.	No relevant data
5	Hsieh P-L, Chen C-M. [Nurse-Led Care Models in the Context of Community Elders With Chronic Disease Management: A Systematic Review]. *Hu Li Za Zhi*. 2016; **63**:35-49. Chinese. doi: 10.6224/JN.63.4.35.	No relevant data
6	Ito J, Edirippulige S, Aono T, Armfield NR. The use of telemedicine for delivering healthcare in Japan: Systematic review of literature published in Japanese and English languages. *J Telemed Telecare* 2017; **23**:828-34. PMID: 29081269. doi: 10.1177/1357633X17732801.	No relevant data
7	Joyce P, Moore ZE, Christie J. Organisation of health services for preventing and treating pressure ulcers. *Cochrane Database Syst Rev* 2018; **12**:CD012132. PMID: 30536917. doi: 10.1002/14651858.CD012132.pub2.	Population
8	Kennedy MA, Hatchell KE, DiMilia PR et al. Community health worker interventions for older adults with complex health needs: A systematic review. *J Am Geriatr Soc* 2021; **69**:1670-82. PMID: 33738803. doi: 10.1111/jgs.17078.	No relevant data
9	Lin JS, Eder M, Weinmann S et al. In*: Behavioral Counseling to Prevent Skin Cancer: Systematic Evidence Review to Update the 2003 U.S. Preventive Services Task Force Recommendation. 2011;* Evidence Synthesis No. 82. AHRQ Publication No. 11-05152-EF-1. Rockville, MD: Agency for Healthcare Research and Quality.	Population
10	Reddy M. Pressure ulcers. *BMJ Clin Evid*. 2011;**2011**:1901. PMID: 21524319.	Setting
11	Reddy M. Pressure ulcers: treatment. *BMJ Clin Evid* 2015; **2015**.:1901. PMID: 26666978.	No relevant data
12	Shi C, Dumville JC, Cullum N et al. Alternating pressure (active) air surfaces for preventing pressure ulcers. *Cochrane Database Syst Rev* 2021; **5**:CD013620. PMID: 33969911. doi: 10.1002/14651858.CD013620.pub2.	No relevant data
13	Turner G, Clegg A, British Geriatrics S et al. Best practice guidelines for the management of frailty: a British Geriatrics Society, Age UK and Royal College of General Practitioners report. *Age Ageing* 2014; **43**:744-7. PMID: 25336440. doi: 10.1093/ageing/afu138.	No relevant data
14	Wang SY, Shamliyan TA, Talley KM et al. Not just specific diseases: systematic review of the association of geriatric syndromes with hospitalization or nursing home admission. *Arch Gerontol Geriatr* 2013; **57**:16-26. PMID: 23578847. doi: 10.1016/j.archger.2013.03.007.	No relevant data

Table S2: Excluded records from search 2 with exclusion reasons.

No	Reference	Exclusion reason
1	Abu-Ashour W, Twells LK, Valcour JE, Gamble JM. Diabetes and the occurrence of infection in primary care: a matched cohort study. *BMC Infect Dis* 2018; **18**:67. PMID: 29402218. doi: 10.1186/s12879-018-2975-2.	Results not presented by age group/setting
2	Akarsu S, Ozbagcivan O, Ilknur T, Semiz F, Fetil E. Sun-related risk factors, perceived seriousness of disease and accompanying non-melanoma skin cancer in patients with actinic keratoses. Dermatologica Sinica. 2018; **36**: 79-84. doi: 10.1016/j.dsi.2017.10.001.	Population/Setting
3	Akhtar A, Ahmad Hassali MA, Zainal H, Khan AH. Prevalence and treatment outcomes of skin infections among elderly population: a retrospective cross-sectional study. *J Dermatolog Treat* 2021; **32**:778-82. PMID: 31916477. doi: 10.1080/09546634.2020.1714539.	Population/Setting
4	Alani A, McCourt C, Morgan E et al. What is the true burden of skin cancer? Recent trends in incidence of nonmelanoma skin cancer pathology reports in Northern Ireland [abstract]. *BJD* (Main Plenary Sessions) 2018; **179** (Suppl. 1):13.	Results not presented by age group/setting
5	Alarcón B, Guarín N, Muñoz-Galindo I, Díaz J, Arévalo H. Prevalencia del cáncer en una aseguradora en salud en Colombia, 2013. *Revista Colombiana de Cancerología* 2015; **19**:210-221. doi: 10.10167j.rccan.2015.07.001.	Results not presented by age group/setting
6	Andrees V, Girbig G, Krensel M, Augustin M, Zander N. Zugang zum gesetzlichen Hautkrebsscreening : Organisatorische und regionale Faktoren in Deutschland [Access to statutory skin cancer screening : Organizational and regional factors in Germany]. *Hautarzt.* 2020; **71**:39-45. doi: 10.1007/s00105-019-04494-1.	No relevant data
7	Arnold A, Lutze S, Ladwig A et al. Prevalence analysis of frequent dermatoses in probands of a population-based study in Pomerania (SHIP-1) [abstract]. In: 39th Annual Meeting of the Arbeitsgemeinschaft Dermatologische Forschung (ADF). *Experimental Dermatology* 2012; **21**:e21. doi:10.1111/j.1600-0625.2011.01428.x.	No relevant data
8	Baccaro LF, Conde DM, Costa-Paiva L et al. Cancer in Women over 50 Years of Age: A Focus on Smoking. *Cancers (Basel)* 2015; **7**:450-9. doi: 10.3390/cancers7010450.	Results not presented by age group/setting
9	Bastuji-Garin S, Joly P, Lemordant P et al. Risk factors for bullous pemphigoid in the elderly: a prospective case-control study. *J Invest Dermatol* 2011; **131**:637-43. doi: 10.1038/jid.2010.301.	Population/Setting
10	Behbahani S, Malerba S, Karanfilian KM et al. Demographics and outcomes of eccrine porocarcinoma: results from the National Cancer Database. *Br J Dermatol* 2020; **183**:161-3. doi: 10.1111/bjd.18874.	No relevant data
11	Berisha M, Miftari-Basholli F, Ramadani N et al. Impact of the National Population Register in Improving the Health Information System of Malignant Diseases in Kosova. *Acta Inform Med* 2018; **26**:62-66. doi: 10.5455/aim.2018.26.62-66.	Results not presented by age group/setting
12	Bernard J, Alexis LG, Pierre LSC, DeGennaro V. A three-year epidemiological profile of cancers managed by a Haitian cancer program from 2016 to 2018 [abstract]. *Journal of Clinical Oncology* 2019; **37** (15_suppl.). doi: 10.1200/JCO.2019.37.15_suppl.e13079.	Results not presented by age group/setting
13	Bernard J, Alexis LG, Pierre LSC, DeGennaro V. A five-year epidemiological profile of patients with cancer managed by a Haitian cancer program [abstract]. *Journal of Clinical Oncology* 2022; **40**(16_suppl). doi: 10.1200/JCO.2022.40.16_suppl.10564.	Results not presented by age group/setting
14	Bliss DZ, Funk T, Jacobson M, Savik K. Incidence and Characteristics of Incontinence-Associated Dermatitis in Community-Dwelling Persons With Fecal Incontinence. *J Wound Ostomy Continence Nurs*. 2015; **42**:525-30. doi: 10.1097/WON.0000000000000159.	Population/Setting
15	Group AW, Busco S, Buzzoni C et al. Italian cancer figures--Report 2015: The burden of rare cancers in Italy. *Epidemiol Prev* 2016; **40**:1-120. PMID: 26951748.	No relevant data

16	Castillo-Cruz UDR, Cortes-Garcia JD, Castanedo-Cazares JP et al. Factors associated with dermatoporosis in a sample of geriatric patients in Mexico. *Gac Med Mex* 2023; **159**:49-54. doi: 10.24875/GMM.M22000737.	Population/Setting
17	Chen D, Yang X, Wang H, Fan W, He Y. Analysis of secular trends in incidence of skin cancer in China based on Joinpoint regression and age-period-cohort model [J]. *CHINESE JOURNAL OF DISEASE CONTROL & PREVENTION,* 2022, **26**:756-765. doi: 10.16462/j.cnki.zhjbkz.2022.07.003.	No relevant data
18	Chen HW, Coias J, Prasad S et al. Patients with limited life expectancy are screened for non-melanoma skin cancers at similar frequencies to healthy patients [abstract]. *Journal of Investigative Dermatology* 2019; **139**:S109. doi:10.1016/j.jid.2019.03.710.	No relevant data
19	Chowdhary V, Crowson C, Maradit-Kremers H, Davis MDP. Incidence of Systemic Lupus Erythematosus and Cutaneous Lupus Erythematosuis in A Population Based Cohort from 1993-2005 [abstract]. *Annals of the Rheumatic Diseases* 2014; **73**:626.	No relevant data
20	Claeson M, Pandeya N, Dusingize JC et al. Assessment of Incidence Rate and Risk Factors for Keratoacanthoma Among Residents of Queensland, Australia. *JAMA Dermatol* 2020; **156**:1324-32. doi: 10.1001/jamadermatol.2020.4097. PMID: 33026421.	No relevant data
21	Coups EJ, Stapleton JL, Hudson SV et al. Skin cancer surveillance behaviors among US Hispanic adults. *J Am Acad Dermatol* 2013; **68**:576-84. doi: 10.1016/j.jaad.2012.09.032. PMID: 23182066.	Results not presented by age group/setting
22	Deilhes F, Boulinguez S, Pages C et al. Advanced Cutaneous Squamous Cell Carcinoma Is Associated with Suboptimal Initial Management in a Cohort of 109 Patients. *Dermatology* 2019; **235**:516-21. doi: 10.1159/000500636. PMID: 31387094.	Population/Setting
23	Diepgen TL, Naldi L, Bruze M et al. Fragrance contact allergy in the European general population [abstract]. *Journal of Investigative Dermatology* 2013; **133**:S103.	Results not presented by age group/setting
24	Diepgen TL, Naldi L, Bruze M et al. High prevalence rates of contact allergy in the European general population [abstract]. *Journal of Investigative Dermatology* 2013; **133**:S101.	Results not presented by age group/setting
25	Diepgen TL, Ofenloch R, Bruze M et al. Prevalence of contact allergy in the European general population [abstract]. *Contact Dermatitis* 2014, **70** (Suppl. 1):94.	Results not presented by age group/setting
26	Diepgen TL, Naldi L, Bruze M et al. Contact allergy to p-phenylene diamine in the general population in five European countries [abstract]. *Contact Dermatitis* 2014, **70** (Suppl. 1):109.	Results not presented by age group/setting
27	Diepgen TL, Ofenloch R, Bruze M et al. Prevalence of fragrance contact allergy in the general population of five European countries: a cross-sectional study. *Br J Dermatol* 2015; **173**:1411-9. doi: 10.1111/bjd.14151. PMID: 26332456.	Results not presented by age group/setting
28	Diestmann K, Grochulska K, Pfeiffer C. Comparing prevalence of sensitisation to birch, and mugwort and of allergic disease and co-morbidities in an aged population (60–89 years) in south-west Germany [poster session]. *Allergy* 2014; **69** (Suppl. 99):209.	No relevant data
29	Egeberg A, Griffiths CEM, Williams HC et al. Clinical characteristics, symptoms and burden of psoriasis and atopic dermatitis in adults. *Br J Dermatol* 2020; **183**:128-38. doi: 10.1111/bjd.18622. PMID: 31630393.	Results not presented by age group/setting
30	Ferris L, Saul M, Lin Y, Weinstock MA, Geller A, Solano F, Bromberg Neuren E, Kirkwood JM. Preliminary outcomes of a primary care-based skin cancer screening program [abstract]. *Journal of Clinical Oncology*. 2016; **34**(15_supp). doi: 10.1200/JCO.2016.34.15_suppl.1508.	Results not presented by age group/setting
31	Ferris LK, Saul MI, Lin Y et al. A Large Skin Cancer Screening Quality Initiative: Description and First-Year Outcomes. *JAMA Oncol* 2017; **3**:1112-5. doi: 10.1001/jamaoncol.2016.6779. PMID: 28241191	Results not presented by

		age group/setting
32	Flohil SC, van der Leest RJ, Dowlatshahi EA et al. Prevalence of actinic keratosis and its risk factors in the general population: the Rotterdam Study. *J Invest Dermatol* 2013; **133**:1971-8. doi: 10.1038/jid.2013.134. PMID: 23510990.	Results not presented by age group/setting
33	Flohil SC, van der Leest RJT, Dowlatshahi EA et al. Prevalence of actinic keratoses and its risk factors: Rotterdam study [abstract]. *British Journal of Dermatology* 2012; **167**: e7.	Results not presented by age group/setting
34	George C, Tokez S, Hollestein L et al. Prevalence and risk factors of actinic keratosis: Results from the Rotterdam study [abstracts]. *Journal of Investigative Dermatolog*y 2022; **142**: S36.	Results not presented by age group/setting
35	Goldenberg G, Karagiannis T, Palmer JB et al. Incidence and prevalence of basal cell carcinoma in a large united states commercially insured population [abstract]. *Value in Health* 2015; **18**: A193-194.	Results not presented by age group/setting
36	Group AW. Italian cancer figures, report 2013: Multiple tumours. *Epidemiol Prev* 2013; **37**:1-152. PMID: 24259384.	No relevant data
37	Gruber V, Hofmann-Wellenhof R, Wolf P et al. Common Benign Melanocytic and Non-Melanocytic Skin Tumors among the Elderly: Results of the Graz Study on Health and Aging. *Dermatology* 2023; **239**:379-86. PMID: 36657431.doi: 10.1159/000529219.	Results not presented by age group/setting
38	Gontijo-Guerra S, Vasiliadis H-M, Berbiche D. Physical and mental multimorbidity patterns in older adults: using network analysis to explore different data sources [abstract]. In: Book of Abstracts [Internet]. IPA/SEPG Joint International Congress: 31. August – 03 September 2019; Santiago de Compostela, Spain. Published online by Cambridge University Press; Preliminary Version (Updated 15 August 2019). 122-123. doi: 10.1017/S1041610219001339	No relevant data
39	Hao EY, Rhodes JEJ, Nixon RL, Saunderson RB. A cross-sectional study of dermatological conditions in rural and urban Timor-Leste. *Australas J Dermatol* 2020; **61**:e395-e8. doi: 10.1111/ajd.13347. PMID: 32542648.	Results not presented by age group/setting
40	Henrikson NB, Morrison CC, Blasi PR et al. In: *Behavioral Counseling for Skin Cancer Prevention: A Systematic Evidence Review for the US Preventive Services Task Force* U.S. Preventive Services Task Force Evidence Syntheses, formerly Systematic Evidence Reviews. Rockville (MD)2018. PMID: 29697227.	Results not presented by age group/setting
41	Gandhi KK, Joo S, Preston JA, Gomez A. Rising Trends in the Incidence of Nonmelanoma Skin Cancer in the United States: Findings from an Administrative Database [abstract]. In: Abstracts of the 29th International Conference on Pharmacoepidemiology & Therapeutic Risk Management [Internet]: August 25-28, 2013; Montreal, Canada. *Pharmacoepidemiol Drug Saf* 2013; **22 Suppl 1**:1-521. doi: 10.1002/pds.3512.	Results not presented by age group/setting
42	Hofman A, Brusselle GG, Darwish Murad S et al. The Rotterdam Study: 2016 objectives and design update. *Eur J Epidemiol* 2015; **30**:661-708. doi: 10.1007/s10654-015-0082-x. PMID: 26386597.	Results not presented by age group/setting
43	Holmes RF, Davidson MW, Thompson BJ, Kelechi TJ. Skin tears: care and management of the older adult at home. *Home Healthc Nurse* 2013; **31**:90-101; quiz 2-3. doi: 10.1097/NHH.0b013e31827f458a. PMID: 23385174.	Publication type
44	Hoorens I, Vossaert K, Pil L et al. Total-Body Examination vs Lesion-Directed Skin Cancer Screening. *JAMA Dermatol* 2016; **152**:27-34. doi: 10.1001/jamadermatol.2015.2680. PMID: 26466155.	Results not presented by age group/setting
45	Hulse K, Carvil M, Chin KY, Butterworth M. New Melanoma Referrals in South East Scotland Over 18 Months [poster presentation]. *BJS* 2020; **107**:149.	Population/Setting

46	Ikram MA, Brusselle GGO, Murad SD et al. The Rotterdam Study: 2018 update on objectives, design and main results. *Eur J Epidemiol* 2017; **32**:807-50. doi: 10.1007/s10654-017-0321-4. PMID: 29064009.	Results not presented by age group/setting
47	Johansson M, Brodersen J, Gotzsche PC, Jorgensen KJ. Screening for reducing morbidity and mortality in malignant melanoma. *Cochrane Database Syst Rev* 2019; **6**. doi: 10.1002/14651858.CD012352.pub2. PMID: 31157404.	Results not presented by age group/setting
48	Kassi K, Djeha D, Gbery IP et al. Psoriasis in elderly patients in the Cote d'Ivoire: socio-demographic, clinical, and therapeutic aspects, and follow-up. *Int J Dermatol* 2016; **55**:e83-6. doi: 10.1111/ijd.13138. PMID: 26517980.	Population/Setting
49	Kelly J, Chiaravalloti A. Prevalence of dermatologic disease in rural Haiti. In: Epidemiology and Health Services Administration [abstract]. *Journal of the American Academy of Dermatology* 2014; **70**:AB83. doi:https://doi.org/10.1016/j.jaad.2014.01.345.	Results not presented by age group/setting
50	Keyghobadi N, Rafiemanesh H, Mohammadian-Hafshejani A et al. Epidemiology and trend of cancers in the province of Kerman: southeast of Iran. *Asian Pac J Cancer Prev* 2015; **16**:1409-13. doi: 10.7314/apjcp.2015.16.4.1409. PMID: 25743807.	Results not presented by age group/setting
51	Kim DH, Chepulis L, Keenan R et al. Prevalence of invasive cancer in a large general practice patient population in New Zealand. *J Prim Health Care* 2020; **12**:215-24. doi: 10.1071/HC19113. PMID: 32988443.	Results not presented by age group/setting
52	King R, Lubbe MS, Gerber JJ. Prevalence of dermatological disorders among patients in an urban area in Namibia [abstract]. *Eur J Epidemiol*. 2013; **28**:S110. doi:10.1007/s10654-013-9820-0	Results not presented by age group/setting
53	Koller EA, Qu L, Adler G. Disease burden in the community dwelling medicare population and impact on lifestyle interventions [abstract]. In: The Endocrine Society's 95th Annual Meeting and Expo: June 15–18, 2013; San Francisco. *Endocrine Reviews*. doi: 10.1093/edrv/34.supp.1	No relevant data
54	Koopmans I, Ford J, Steel N. Risks of skin cancer in older adults: Analysis of the English Longitudinal Study of Ageing [abstract]. In: Meeting Abstracts. *The Lancet* 2017; **390**: S54. doi: 10.1016/S0140-6736(17)32989-6.	Results not presented by age group/setting
55	Kottner J, Boronat X, Blume-Peytavi U et al. The epidemiology of skin care provided by nurses at home: a multicentre prevalence study. *J Adv Nurs* 2015; **71**:570-80. doi: 10.1111/jan.12517. PMID: 25159337.	No relevant data
56	Lai M, Pampena R, Mirra M et al. Characteristics and management of skin cancers in very elderly patients: A real-world challenge for clinicians. *Exp Dermatol* 2022; **31**:1554-62. doi: 10.1111/exd.14627. PMID: 35723894.	Population/Setting
57	Lapi F, Cassano N, Pegoraro V et al. Epidemiology of chronic spontaneous urticaria: results from a nationwide, population-based study in Italy. *Br J Dermatol* 2016; **174**:996-1004. doi: 10.1111/bjd.14470	Results not presented by age group/setting
58	Li J, Wang B, Deng Y et al. Epidemiological features of rosacea in Changsha, China: A population-based, cross-sectional study. *J Dermatol* 2020; **47**:497-502. doi: 10.1111/1346-8138.15301. PMID: 32207167.	Population/Setting
59	Li M, Wang P, Wu W et al. An epidemiological survey of psoriasis in 18 cities in Hainan province of China [poster session]. *Journal of Dermatology* 2012; 39 (Suppl. 1):243. doi: 10.1111/j.1346-8138.2012.01624.x.	Results not presented by age group/setting
60	Lichterfeld-Kottner A, Lahmann N, Lechner A, Blume-Peytavi U, Kottner J. The prevalence of dry skin in different health care settings in Germany [abstract]. In: 2nd European Dermato-Epidemiology Network (EDEN) Forum Abstracts. *Dermatologie in Beruf und Umwelt* 2018; 66:41.	Results not presented by age group/setting

61	Lima AS, Stein CE, Casemiro KP, Rovere RK. Epidemiology of melanoma in the South of Brazil: study of a city in the Vale do Itajai from 1999 to 2013. *An Bras Dermatol* 2015; **90**:185-9. doi: 10.1590/abd1806-4841.20153076. PMID: 25830987.	Population/Setting
62	Llor C, Hernandez S. [Infectious disease in primary care: 1-year prospective study]. Spanish. *Enferm Infecc Microbiol Clin* 2010; **28**:222-6. doi: 10.1016/j.eimc.2009.03.014. PMID: 19720435.	Results not presented by age group/setting
63	Lopes TS, Videira L, Saraiva D et al. Multicentre study of pressure ulcer point prevalence in a Portuguese region. *J Tissue Viability* 2020; **29**:12-8. doi: 10.1016/j.jtv.2019.11.002. PMID: 31796241.	Population/Setting
64	Maelegheer K, Dumitrescu I, Verpaelst N et al. Infection prevention and control challenges in Flemish homecare nursing: a pilot study. *Br J Community Nurs* 2020; **25**:114-21. doi: 10.12968/bjcn.2020.25.3.114. PMID: 32160023.	Results not presented by age group/setting
65	Mahler V. [Contact allergies in the elderly]. *Hautarzt* 2015; **66**:665-73. doi: 10.1007/s00105-015-3668-z. PMID: 26285897.	Population/Setting
66	Mehta NK, Nguyen SA, Chang BA, Nathan CA. Trend Analysis of Cutaneous Squamous Cell Carcinoma of the External Lip From 1975 to 2016. *JAMA Otolaryngol Head Neck Surg* 2021; **147**:624-31. doi: 10.1001/jamaoto.2021.0760. PMID: 33983364.	No relevant data
67	Mirza F, Mirza H, Yumeen S. Epidemiology of cutaneous mucoepidermoid carcinoma: A United States population-based cohort analysis using the Surveillance, Epidemiology, and End Results database [abstract]. *Journal of the American Academy of Dermatology* 2020; **83**(SUPPLEMENT):AB123. doi:https://doi.org/10.1016/j.jaad.2020.06.579.	No relevant data
68	Naess G, Kirkevold M, Hammer W et al. Nursing care needs and services utilised by home-dwelling elderly with complex health problems: observational study. *BMC Health Serv Res* 2017; **17**:645. doi: 10.1186/s12913-017-2600-x. PMID: 28899369.	Population/Setting
69	Ogrum A, Demir O. Does counseling have an effect on sun protection behaviors and early detection of skin cancer in middle-aged and older Turkish people? *Turkish Archives of Dermatology and Venereology*. 2020; **54**:9. Available from: https://link.gale.com/apps/doc/A659008754/AONE?u=anon~f5479264&sid=googleScholar&xid=b052e46d.	Results not presented by age group/setting
70	Oien RF, Wickstrom H, Akesson N et al. Clinical evaluation with long-term follow-up of patients with pressure ulcers in one Swedish county. *J Wound Care* 2020; **29**:472-8. doi: 10.12968/jowc.2020.29.8.472. PMID: 32804034.	Results not presented by age group/setting
71	Olsen CM, Green AC, Neale RE et al. Cohort profile: the QSkin Sun and Health Study. *Int J Epidemiol* 2012; **41**:929-i. doi: 10.1093/ije/dys107. PMID: 22933644.	Results not presented by age group/setting
72	Pacific K, Babbush K, Cohen S. Hidradenitis suppurativa in a cohort of sixty years and older [abstract]. *Journal of Investigative Dermatology.* 2020; **140**:S65.	No relevant data
73	Piontek K, Ittermann T, Arnold A, Baumeister S, Apfelbacher CJ. Divergence in the prevalence of self-reported and physician-reported diagnosis of atopic dermatitis in adults: Results from a population-based study [abstract].In: 47th Annual Meeting of the Arbeitsgemeinschaft Dermatologische Forschung (ADF). *Experimental Dermatology*. 2021; **30**:e37. doi: 10.1111/exd.14263.	Results not presented by age group/setting
74	Podder I, Kumar D. A cross-sectional observational study to analyze the clinico-demographic profile of geriatric dermatoses and assess their relationship with systemic disorders [abstract]. In: Abstract Book. 24th World Congress of Dermatology: 10-15 June 2019; Milan Italy. *Journal of the Dermatology Nurses' Association* 2020; **12.**	Population/Setting
75	Powell RJ, Hayward CJ, Snelgrove CL et al. Pilot parallel randomised controlled trial of protective socks against usual care to reduce skin tears in high risk people: 'STOPCUTS'. *Pilot Feasibility Stud* 2017; **3**:43. doi: 10.1186/s40814-017-0182-3. PMID: 29075507.	Results not presented by age group/setting
76	Prado G, D'Amore P, Tagliero A, Florez-White M, Acuna J. Melanoma incidence from 2001-2011 and predictors of stage at diagnosis []abstract]. *Journal of the American Academy of Dermatology* 2016; **74**(Supplement 1):AB120. doi:https://doi.org/10.1016/j.jaad.2016.02.469.	No relevant data

77	Reich K, Griffiths CEM, Paul C et al. Patient perspectives on the burden of atopic dermatitis: results from the Atopic Dermatitis Patient Satisfaction and Unmet Need Survey [oral presentation]. *British Journal of Dermatology* 2020; **183:**192.	Results not presented by age group/setting
78	Rodriguez-Blanco I, Florez A, Paredes-Suarez C et al. Actinic Cheilitis Prevalence and Risk Factors: A Cross-sectional, Multicentre Study in a Population Aged 45 Years and Over in North-west Spain. *Acta Derm Venereol* 2018; **98**:970-4.doi: 10.2340/00015555-3014. PMID: 30085328.	Results not presented by age group/setting
79	Romani L, Hamid M, Steer A et al. Prevalence of scabies in Fiji: A national study [abstract]. *American Journal of Tropical Medicine and Hygiene* 2012; **87**:173.	Results not presented by age group/setting
80	Ronneikko JK, Jamsen ER, Makela M et al. Reasons for home care clients' unplanned Hospital admissions and their associations with patient characteristics. *Arch Gerontol Geriatr* 2018; **78**:114-26. doi: 10.1016/j.archger.2018.06.008. PMID: 29957266.	No relevant data
81	Rosenbaum BE, Freitas D, Nosal SC, Meydani A. Skin Disease in the Uninsured: Diagnoses, Management Decisions, and Referral Outcomes of an Urban Free Clinic. *J Health Care Poor Underserved* 2016; **27**:834-45. doi: 10.1353/hpu.2016.0068. PMID: 27180711.	Population/Setting
82	Roshani Z, Akbari Kamrani AA, Shati M, Sahaf R. Prevalence of Types of Cancers in the Elderly Covered by Insurance of the Islamic Republic of Iran Broadcasting Company in 2015 - Comparison with Younger Groups. *Asian Pac J Cancer Prev* 2016; **17**:269-73. doi: 10.7314/apjcp.2016.17.s3.269. PMID: 27165237.	No relevant data
83	Sabban S, Sloane PD, Park SW, Ward KT, Halpert KD. The importance of taking off the shoes and looking for foot and toe abnormalities of old and disabled persons [abstract]. In: Annual Scientific Meeting Abstract Book. *Journal of the American Geriatrics Society* 2017; **65**:S250.	Results not presented by age group/setting
84	Sampogna F, Abeni D, Gieler U et al. Exploring the EQ-5D Dimension of Pain/Discomfort in Dermatology Outpatients from a Multicentre Study in 13 European Countries. *Acta Derm Venereol* 2020; **100**:adv00120. doi: 10.2340/00015555-3477. PMID: 32250441.	Population/Setting
85	Schikowski T, Seite S, Guo Q et al. Epidemiologic evidence for a negative association between air pollution and basal cell carcinoma in elderly Caucasian women [abstract]]. *Journal of the American Academy of Dermatology.* 2020; 83:AB131. doi: https://doi.org/10.1016/j.jaad.2020.06.613	No relevant data
86	Schikowski T, Seite S, Guo Q, Hüls A, Fuks K, Sugiri D, Moyal D, Krutmann J. Epidemiologic evidence for a negative association between air pollution and basal cell carcinoma in elderly Caucasian women [abstract]]. *J AM ACAD DERMATOL* 2020; 83:AB131	No relevant data
87	dos Santos MM, Amaral S, Harmen SP et al. The prevalence of common skin infections in four districts in Timor-Leste: a cross sectional survey. *BMC Infect Dis* 2010; **10**:61. doi: 10.1186/1471-2334-10-61. PMID: 20219136.	No relevant data
88	Schnass W, Hüls A, Vierkotter A et al. Air pollution and incidence of eczema in elderly women [abstract]. *Journal of Investigative Dermatology* 2017; **137**:S26.	No relevant data
89	Schofield J, Sherlock J, De Lusignan S. Trends in the attendance of people with skin conditions in English general practice 2006–16: a sentinel network database study [oral presentation]. *British Journal of Dermatology* 2020; **183**:23.	Population/Setting
90	Serrano AVO, Contreras F, Triana I et al. Prognostic factors for recurrence and mortality in patients with localized malignant melanoma: Analysis of the Epidemiological Registry of Malignant Melanoma in Colombia REMMEC ACHO [abstract]. *Journal of Clinical Oncology* 2022; **40**:e21572.	Population/Setting
91	Shah P, Bajaj S, Polsky D. Late-stage melanoma diagnosis in New York State (NYS) [abstract]. *Journal of Investigative Dermatology* 2020; 140:S88.	No relevant data
92	Siesling S, Visser O, Aarts MJ et al. [Fight against cancer in the Netherlands: current state of affairs]. *Ned Tijdschr Geneeskd* 2019; **163**. PMID: 31283131.	Results not presented by age group/setting
93	Singhal RR, Talati KN, Gandhi BP et al. Prevalence and Pattern of Skin Diseases in Tribal Villages of Gujarat: A Teledermatology Approach. *Indian J Community Med* 2020; **45**:199-203. doi: 10.4103/ijcm.IJCM_76_19. PMID: 32905082.	Results not presented by

		age group/setting
94	Sorbye LW, Hamran T, Henriksen N, Norberg A. Home care patients in four Nordic capitals - predictors of nursing home admission during one-year followup. *J Multidiscip Healthc* 2010; **3**:11-8. doi: 10.2147/jmdh.s8979. PMID: 21197351.	No relevant data
95	Steenrod AW, Smyth EN, Bush EN et al. A Qualitative Comparison of Symptoms and Impact of Varying Stages of Basal Cell Carcinoma. *Dermatol Ther (Heidelb)* 2015; **5**:183-99. doi: 10.1007/s13555-015-0081-6. PMID: 26324194.	Results not presented by age group/setting
96	Steglich RB, Cardoso S, Gaertner M et al. Differences in the diagnosis of primary cutaneous melanoma in the public and private healthcare systems in Joinville, Santa Catarina State, Brazil. *An Bras Dermatol* 2018; **93**:507-12. doi: 10.1590/abd1806-4841.20185767. PMID: 30066755.	No relevant data
97	Svensson A, Ofenloch RF, Bruze M et al. Prevalence of skin disease in a population-based sample of adults from five European countries. *Br J Dermatol* 2018; **178**:1111-8. doi: 10.1111/bjd.16248. PMID: 29247509.	Population/Setting
98	Takahashi P, Cha S, Kalava U. Venous ulcers and vitamin D levels: A cohort study in an older population [poster]. *European Geriatric Medicine* 2010; **1**:S118. doi: 10.1016/j.eurger.2010.07.009.	No relevant data
99	Telvizian T, Al Ghadban Y, Alawa J et al. Knowledge, beliefs, and practices related to cancer screening and prevention in Lebanon: community and social media users' perspectives. *Eur J Cancer Prev* 2021; **30**:341-9. doi: 10.1097/CEJ.0000000000000631. PMID: 32956077.	Results not presented by age group/setting
100	Tentolouris N, Papanas N, Panagoulias G et al. The association between Neuropad testing with foot ulceration in diabetes [abstract]. In: Abstracts of the 50th EASD Annual Meeting: September 15-19, 2014; Vienna, Austria. *Diabetologia* 2014; **57**(Suppl 1):S471. doi: 10.1007/s00125-014-3355-0.	No relevant data
101	Thume E, Nunes BP, Soares MU et al. Facchini LA. Health status of the elderly in bage, state of rio grande do sul, brazil [abstract]. *Age and Ageing* 2018; **47**:ii7–ii8. doi: 10.1093/ageing/afy030.02	No relevant data
102	Trautmann F, Meier F, Seidler A, Schmitt J. Utilization of the German skin cancer screening program and effects on melanoma incidence and disease severity-a secondary data-based analysis [abstract]. In: Grill E, Muller M, Mansmann U. Health-exploring complexity: an interdisciplinary systems approach HEC 2016: 28 August-2 September 2016, Munich, Germany. *Eur J Epidemiol* 2016; **31**(Suppl 1): S212. doi: 10.1007/s10654-016-0183-1.	Results not presented by age group/setting
103	Trihan JE, Laneelle D, Metcalfe N et al. Diabetes-associated dermatological manifestations in primary care and their association with vascular complications. *J Diabetes Metab Disord* 2020; **19**:989-96. doi: 10.1007/s40200-020-00594-1. PMID: 33520817.	Results not presented by age group/setting
104	Tun K, Shurko JF, Ryan L, Lee GC. Age-based health and economic burden of skin and soft tissue infections in the United States, 2000 and 2012. *PLoS One* 2018; **13**:e0206893. doi: 10.1371/journal.pone.0206893. PMID: 30383858.	Results not presented by age group/setting
105	Ultsch B, Weidemann F, Reinhold T et al. Health economic evaluation of vaccination strategies for the prevention of herpes zoster and postherpetic neuralgia in Germany. *BMC Health Serv Res* 2013; **13**:359. doi: 10.1186/1472-6963-13-359. PMID: 24070414.	No relevant data
106	Ultsch B, Reinhold T, Siedler A, Krause G, Wichmann O. Health Economic Evaluation of the Vaccination Against Herpes Zoster and Postherpetic Neuralgia in Germany [abstract]. *Value in Health* 2012; **15**:A394. doi: https://doi.org/10.1016/j.jval.2012.08.1118.	No relevant data
107	Unsal AA, Patel VR, Chung SY et al. Head and neck sweat gland adenocarcinoma: A population-based perspective of a rare entity. *Laryngoscope* 2017; **127**:2757-62. doi: 10.1002/lary.26593. PMID: 28397272.	No relevant data
108	Vogelgsang L, Loerbroks A, Apfelbacher C, Matterne U, Weisshaar E. Incidence of chronic pruritus and its determinants: Results from a population-based study [abstract]. In: 39th Annual Meeting of the Arbeitsgemeinschaft Dermatologische Forschung (ADF). *Experimental Dermatology* 2012; **21**:e41. doi: 10.1111/j.1600-0625.2011.01428.x.	Results not presented by age group/setting

109	Wernli KJ, Henrikson NB, Morrison CC et al. Screening for Skin Cancer in Adults: Updated Evidence Report and Systematic Review for the US Preventive Services Task Force. *JAMA* 2016; **316**:436-47. doi: 10.1001/jama.2016.5415. PMID: 27458949.	Results not presented by age group/setting
110	Williamson S, Merritt J, De Benedetto A. Atopic dermatitis in the elderly: a review of clinical and pathophysiological hallmarks. *Br J Dermatol* 2020; **182**:47-54. doi: 10.1111/bjd.17896. PMID: 30895603.	No relevant data
111	Wu JJ, Veverka KA, Lu M et al. A new longitudinal EHR database for research in dermatology [abstract]. *Journal of the American Academy of Dermatology* 2018; **79:**AB15. doi: 10.1016/j.jaad.2018.05.103.	Results not presented by age group/setting
112	Yew YW, Kuan AHY, George PP et al. Prevalence and burden of skin diseases among the elderly in Singapore: a 15-year clinical cohort study. *J Eur Acad Dermatol Venereol* 2022; **36**:1648-59. doi: 10.1111/jdv.18205. PMID: 35535625.	Population/Setting
113	Youlden DR, Soyer HP, Youl PH et al. Incidence and survival for Merkel cell carcinoma in Queensland, Australia, 1993-2010. *JAMA Dermatol* 2014; **150**:864-72. doi: 10.1001/jamadermatol.2014.124. PMID: 24943712.	No relevant data
