# Supplementary material for: Skin health of community-living older people: a scoping review
Source: Arch Dermatol Res. 2024 Jun 1;316(6):319. doi: 10.1007/s00403-024-03059-0 (PMC11144137; doi:10.1007/s00403-024-03059-0)
Supplement: Supplementary file 2 — Supplementary Material 2 [file 403_2024_3059_MOESM2_ESM.pdf]

Supplementary material 2

Article title: Skin health of community-living older people: a scoping review

Authors: Jan Kottner[1], Alexandra Fastner[1], Dimitra-Aikaterini Lintzeri[2], Ulrike Blume-Peytavi[2], Christopher E. M. Griffiths[3,4]

Institutions

[1]Institute of Clinical Nursing Science, Charité Universitätsmedizin Berlin, Berlin, Germany.

[2]Department of Dermatology, Venerology and Allergology, Charité Universitätsmedizin Berlin, Berlin, Germany.

[3]Department of Dermatology, King's College Hospital, King's College London, London, UK.

[4]Centre for Dermatology Research, NIHR Manchester Biomedical Research Centre, The University of Manchester, Manchester, UK.

Corresponding author Jan Kottner

Charité – Universitätsmedizin Berlin,

Institute of Clinical Nursing Science,

Charitéplatz 1,

10117 Berlin, Germany

Email: jan.kottner@charite.de

Author(s)	Akbari, M. E.; Rafiee, M.; Khoei, M. A.; Eshrati, B.; Hatami, H.
Titel	Incidence and survival of cancers in the elderly population in Iran: 2001-2005
Year of publication	2011[1]
Review Question (1/2/3)	1
Aim/purpose/objective	• Determine incidence of different types of cancers among elderly Iranian population<br>• Estimate the 5-year survival rate of different types of cancers among elderly Iranian population
Design	Analysis of registry data
Population (Inclusion/Exclusion Criteria)	Data from national cancer registry department of the Ministry of Health and Medical Education in Iran<br>• Types of cancers included: bladder, osophage , colorectal, gastric, breast, ovarian, cervicx, thyroid, lung, luchemia, lymphoma, prostate
Methods	• Time period analysed: 2001-2005<br>• Only cases with telephone number included → persons were called to find out about life status<br>• Analysis for crude, age adjusted and age-specific annual cancer incidence rates<br>• Trend analysis and survival estimates/probabilities
Sample characteristics	• n = 77862 cases of <u>any cancer</u> in people ≥ 60 years<br>• 66.5% men<br>• Mean age: 71.2 years<br>• Median age: 70 years<br>• 64.2% in age group 60-74 years<br>• 27.8% in age group 75-90 years<br>• 7.8% in age group 90+
Key findings relevant for review questions	• Highest incidence of cancer in five-year cohort among elderly population was for skin cancer (371 per 100,000).<br>• Incidence Rate of Skin Cancer in Iranian Aged Men Population (2001-2005): 458.1/100,000<br>• Incidence Rate of Skin Cancer in Iranian Aged Women Population (2001-2005): 278/100,000<br>• Incidence rate for ages 60-74 years: 301.2/100,000 (fig. 3)<br>• Incidence rate for ages 75-90 years: 450.1/100,000 (fig.3)<br>• Incidence rate for ages 90+ years: 2191/100,000 (fig. 3)
Notes	• Article has many typing and english language errors.<br>• Figure 3 includes no denominator – it was assumed it is /100,000 like the other figures<br>• 'Skin cancer' not defined<br>• National cancer registry department of the Ministry of Health and Medical Education in Iran: all pathology laboratories, daily cancer clinics, medical centers, other states central registries, coverage rate nearly 80% of majority types of cancers

Author(s)	Augustin, M.; Herberger, K.; Hintzen, S.; Heigel, H.; Franzke N.; Schäfer I.
Titel	Prevalence of skin lesions and need for treatment in a cohort of 90,880 workers
Year of publication	2011[2]
Review Question (1/2/3)	1
Aim/purpose/objective	• Determine prevalence of skin lesions and the need for care based on dermatological examinations in working adults in Germany
Design	Cross-sectional
Population (Inclusion/Exclusion Criteria)	Employees from 312 German companies<br>(Volunteers from companies of different branches of industry in Germany (car industry, insurance companies, banking, energy companies, chemical industry and printing houses, bank and postal service))
Methods	• Standardised whole-body examinations and interviews of the workers by dermatologists on premises of participating companies during working hours
Sample characteristics	• n = 90 880 employees (from 312 companies) (total sample)<br><br>Age 61–70:<br>• Total: n = 6860 (7.5%)<br>• Male: n = 3538 (7.3%)<br>• Female: n = 3322 (7.8%)
Key findings relevant for review questions	Prevalence of benign, noninflammatory skin diseases (see table below)<br><br>Prevalence of actinic keratosis<br>➔ App. 12% in 61-70 year old (fig. 1)<br><br>Prevalence of inflammatory skin diseases (see table below)

Prevalence of benign, noninflammatory skin diseases

**Age group: 61-70 years**	**n**	**%**
More than 40 naevi	497	7.24
Dermal naevi	1745	25.44
Papillomatous naevi	1082	15.77
Seborrhoeic keratosis	4577	66.72
Cafe´-au-lait patches	281	4.10
Histiocytoma ⁄dermatofibroma	1398	20.38
Solar (senile) lentigines	4912	71.60
Vitiligo	45	0.66
Lipoma	195	2.84
Other cysts	32	0.47

Prevalence of inflammatory skin diseases

**Age group: 61-70 years**	n	%
Atopic eczema	70	1.02
Xerotic eczema	131	1.91
Hand eczema, (cumulative toxic, allergic)	53	0.77
Acne	15	0.22
Intertrigo	38	0.55
Contact dermatitis	21	0.31
Lichen planus	9	0.13
Psoriasis	150	2.19
Rosacea	428	6.24
Other in flammatory diseases	126	1.84

Prevalence of clinically diagnosed dermatomycoses

**Age group: 61-70 years**	n	%
Onychomycosis	1207	17.6
Pityriasis versicolor	53	0.8
Tinea pedis	650	9.5
Tinea corporis	43	0.6
Other mycotic diseases	25	0.4
At least one condition	1613	23.5

Prevalence of clinically diagnosed bacterial diseases of the skin

**Age group: 61-70 years**	n	%
Bacterial folliculitis	247	3.60
Pyoderma	64	0.93
Other bacterial diseases	10	0.15

Prevalence of clinically diagnosed viral diseases of the skin

**Age group: 61-70 years**	n	%
Warts of the feet	160	2.33
Warts of the hands	30	0.44
Other viral diseases	54	0.79

Notes	-

<table>
<tr><td>Author(s)</td><td>Thomas Frese, Kristin Herrmann, Hagen Sandholzer</td></tr>
<tr><td>Titel</td><td>Pruritus as Reason for Encounter in General Practice</td></tr>
<tr><td>Year of publication</td><td>2011[3]</td></tr>
<tr><td>Review Question (1/2/3)</td><td>2</td></tr>
<tr><td>Aim/purpose/objective</td><td>Characterize the consultation prevalence of pruritus,
Characterize the frequency of diagnostic and therapeutic procedures,
Characterize the accompanying symptoms and results of encounter or diagnoses of patients with pruritus</td></tr>
<tr><td>Design</td><td>Cross-sectional</td></tr>
<tr><td>Population (Inclusion/Exclusion Criteria)</td><td>Patients of participating GP’s in Saxony, Germany
(Dutch population in Transition Project)</td></tr>
<tr><td>Methods</td><td>Data collection from October 1, 1999 to September 30, 2000
Comparison of collected data with data from Dutch Transition Project and Australian BEACH study
“Data was collected for one in ten patients previously know to the practicioner” on one day a week randomly
Standardised data collection form</td></tr>
<tr><td>Sample characteristics</td><td>Patients of 209 general practicioners
n = 8,877 (total, all age groups)
56.9% female</td></tr>
<tr><td>Key findings relevant for review questions</td><td>Patient Distribution* (pd) on Different Age Groups and Consultation Prevalence** (cp) of Pruritus
<table>
<tr><td></td><td colspan="2">SESAM 2 study (n = 64)</td><td colspan="2">Transition Project (consultation for newly occurred Pruritus) n = 5163</td><td colspan="2">Transition Project (consultation for previously know pruritus) n = 1854</td></tr>
<tr><td>Age (years)</td><td>Pd (%)</td><td>Cp (%)</td><td>Pd (%)</td><td>Cp (%)</td><td>Pd (%)</td><td>Cp (%)</td></tr>
<tr><td>65 to 74</td><td>14.1</td><td>0.5</td><td>11.5</td><td>3.7</td><td>16.3</td><td>1.9</td></tr>
<tr><td>> 75</td><td>18.8</td><td>1.0</td><td>9.6</td><td>3.4</td><td>14.4</td><td>1.8</td></tr>
</table>
Patient distribution (pd) is the percentage of the specified age group of the patients with pruritus (e.g. 14.1 % of all SESAM 2-patients with pruritus were 65 to 74 years old)
Consultation prevalence (cp) is the percentage of patients with pruritus in the age group related to all patients of the age group (e.g. among SESAM 2-patients from 65 to 74 years of age 0.5% encounter for pruritus)</td></tr>
<tr><td>Notes</td><td>-</td></tr>
</table>

Author(s)	Paul, C.; Maumus-Robert, S.; Mazereeuw-Hautier, J.; Guyen, C. N.; Saudez, X.; Schmitt A. M.
Title	Prevalence and risk factors for xerosis in the elderly: a cross-sectional epidemiological study in primary care
Year of publication	2011[4]
Review Question (1/2/3)	1
Aim/purpose/objective	• To establish the prevalence of xerosis and associated factors in elderly patients
Design	Cross-sectional
Population (Inclusion/exclusion Criteria)	Patients presenting in General Practitioner (GP) offices in France<br>Inclusion:<br>• Age ≥ 65 years<br>• Capable of answering questions related to their personal history since childhood
Methods	• Random selection of GPs from a comprehensive French database of GPs<br>• Consecutive recruitment of first 10 patients aged ≥ 65 years presenting to sampled GPs in their office over 1 week<br>• Completion of a survey focusing on personal and family history of atopy, environment, and life habits.<br>• Visual examination using the Overall Dry Skin score (ODS)<br>• For patients with ODS ≥1: evaluation of skin dryness intensity and pruritus using a five-point Likert scale.
Sample characteristics	• n = 756 (43% men)<br>• Mean age: 75.1 ± 6.9 years<br>- Without xerosis: 74.2± 6.7 years<br>- With xerosis: 75.9± 6.9 years
Key findings relevant for review question(s)	• "The prevalence of any degree of xerosis was 55.6% in the population."<br>• Factors significantly associated with xerosis were among others older age and female sex.<br>• "A higher severity of xerosis was associated with older age"
Notes	-

Overall Dry Skin score (ODS)
General Practitioner (GP)

Author(s)	Ritchie, S. R.; Fraser, J. D.; Libby, E.; Morris, A. J.; Rainey, P. B.; Thomas, M. G
Titel	Demographic variation in community-based MRSA skin and soft tissue infection in Auckland, New Zealand
Year of publication	2011[5]
Review Question (1/2/3)	1
Aim/purpose/objective	• To estimate the burden of skin and soft tissue infection caused by Staphylococcus aureus (S. aureus)<br>• To determine the effects of ethnicity and age on the rate of skin and soft tissue due to MRSA
Design	Secondary data analysis
Population (Inclusion/exclusion Criteria)	Residents in Auckland, New Zealand who had a wound swab culture taken<br>• No age limits<br>Exclusion:<br>• Non-wound swabs<br>• MRSA-screening swabs
Methods	• Culture and susceptibility results of all wound swabs processed by Auckland's only community microbiology laboratory in 2007 were retrieved<br>• Collection of demographic data and proportions of S. aureus, MRSA, nm-MRSA and mr-MRSA for a random sample of 1000 people who had a wound swab collected<br>• Incidence of a positive culture of S. aureus was calculated from comparative demographic information for the total Auckland population obtained from the 2006 Census dataset<br>• Estimation of relative rates of S. aureus and MRSA per ethnic group and age group.
Sample characteristics	• n = 1000 for complete assessment of demographic data, subtypes<br>• n = 47,047 wound swabs
Key findings relevant for review question(s)	• n = 23,853/47,047 were S. aureus<br>• n = 1872/23,853 positive for MRSA<br>(n = 1794 after duplicate swabs excluded)<br>• mr-MRSA constant (low) until age 80, then it increases sharply for NZ European<br>• "The highest incidence of culture of either subtype of MRSA from wound swabs was 627/100,000 observed in mr-MRSA in NZ European over the age of 80 years. At ages less than 70 years mr-MRSA infections were rare".
Notes	• Paper was retrieved from author

Author(s)	Wu, J.; Guo, Z.; Berman, R.; Coric, V.; Kumar, S.; Radhakrishnan, M.
Titel	Occurrence of nonmelanoma skin cancer in the elderly with and without Alzheimer's disease in the US
Year of publication	2011a[6]
Review Question (1/2/3)	1
Aim/purpose/objective	• To "estimate the [incidence] rates of non-melanoma skin cancer (NMSC) in the elderly with and without Alzheimer's disease"
Design	Secondary data analysis
Population (Inclusion/exclusion Criteria)	Data from PharMetrics database<br>Inclusion:<br>• Patients ≥ 65 years with a diagnosis of Alzheimer's disease (ICD-9-CM 331.0)<br>• Matched health plan enrollees
Methods	• Alzheimer's disease cohort identified retrospectively from 1/1/2003 to 12/31/2006 using the PharMetrics database<br>• Control cohort: Random sample of health plan enrollees matched to the AD cohort by age, gender, geographic location, calendar year<br>→ 1:1 ratio.<br>• Cohorts were followed for NMSC event from the index date for two years.<br>• Four different algorithms were used to identify NMSC cases: by ICD-9-CM alone, by Current Procedural Terminology (CPT) alone, by ICD-9-CM or CPT, by ICD-9-CM and CPT.
Sample characteristics	• n = 27,076 (per group)<br>• Mean age: 84.1 (SD 8.9) years<br>• Majority female
Key findings relevant for review question(s)	Incidence rate of NMSC in AD cohort:<br>• (ICD alone),3.9 per 100 person-years<br>• (CPTalone), 3.1 per 100 person-years<br>• (ICD and CPT), 3.0 per 100 person-years<br>• (ICD or CPT), 4.1 per 100 person-years<br><br>Incidence rate of NMSC in the non-AD cohort:<br>• (ICD alone), 5.2 per 100 person-years<br>• (CPT alone), 4.3 per 100 person-years<br>• (ICD and CPT), 4.2 per 100 person-years<br>• (ICD or CPT), 5.6 per 100 person-years.<br><br>→ Rate in non-AD cohort slightly higher ($p < 0.001$)
Notes	• Conference Abstract<br>• Second conference abstract:<br><br>**Wu (2011b)**[7]<br>Wu J, Guo Z, Berman R et al. 698. Risk of Non-Melanoma Skin Cancer in Elderly Patients with Alzheimer's Disease [abstract]. In: 27th International Conference on Pharmacoepidemiology and Therapeutic Risk Management, Chicago, United States. *Pharmacoepidemiology and Drug Safety* 2011; 20:S303-4.

	Incidence rate of NMSC in AD cohort:<br>• (ICD alone),39.2 per 1000 person-years<br>• (CPTalone), 30.6 per 1000 person-years<br>• (ICD and CPT), 30.1 per 1000 person-years<br>• (ICD or CPT), 41.1 per 1000 person-years<br><br>Incidence rate of NMSC in the non-AD cohort:<br>• (ICD alone), 52.4 per 1000 person-years<br>• (CPT alone), 43.0 per 1000 person-years<br>• (ICD and CPT), 41.9 per 1000 person-years<br>• (ICD or CPT), 56.2 per 1000 person-years.

Non-melanoma skin cancer (NMSC)

<table>
<tr><td>Author(s)</td><td>L. M. Hollestein, S. A. W. van den Akker, T. Nijsten, H. E. Karim-Kos, J. W. Coebergh, E. de Vries</td></tr>
<tr><td>Titel</td><td>Trends of cutaneous melanoma in The Netherlands: increasing incidence rates among all Breslow thickness categories and rising mortality rates since 1989</td></tr>
<tr><td>Year of publication</td><td>2012[8]</td></tr>
<tr><td>Review Question (1/2/3)</td><td>1/2</td></tr>
<tr><td>Aim/purpose/objective</td><td>To examine the controversy regarding the debate “that the epidemic of melanoma is largely due to overdiagnosis, since increases in incidence were mainly among thin melanomas and mortality rates remained stable” in the Netherlands</td></tr>
<tr><td>Design</td><td>Analysis of registry data</td></tr>
<tr><td>Population (Inclusion/Exclusion Criteria)</td><td>Data from Netherlands Cancer Registry (NCR)
Inclusion:
Patients with diagnosis of invasive primary cutaneous melanoma (C43) in time period
Exclusion:
Cases age <15 and >95 years
Diagnosed by autopsy</td></tr>
<tr><td>Methods</td><td>Patient characteristics from medical records
Time period 1989–2008
Vital status followed up until death or 2010
Analysis of mortality data for the period 1989–2009 from Statistics Netherlands (CBS).</td></tr>
<tr><td>Sample characteristics</td><td>Average population size (Netherlands) 1989 to 2008: 15.7 million
Diagnosed male (≥65): n = 6396
Diagnosed female (≥65): n = 7986</td></tr>
<tr><td>Key findings relevant for review questions</td><td>1.
Age-standardized incidence rates (per 100 000 person-years)
<table>
<tr><td></td><td>Male (≥65)</td><td>Female (≥65)</td></tr>
<tr><td>1989-1993</td><td>2.3</td><td>2.4</td></tr>
<tr><td>1994-1998</td><td>3.2</td><td>3.0</td></tr>
<tr><td>1999-2003</td><td>4.2</td><td>3.5</td></tr>
<tr><td>2004-2008</td><td>5.7</td><td>4.7</td></tr>
<tr><td>EAPC (95%CI)</td><td>5.98 (5.53 to 6.44)</td><td>4.33 (3.64 to 5.02)</td></tr>
</table>
2.
Mortality rates (year 2008):
F: 11/100,000 person- years (fig.3)
M: 18/100,000 person-years (fig.3)
(European Standardised rate)</td></tr>
<tr><td>Notes</td><td>NCR: “based on notification of all newly diagnosed malignancies in the Netherlands by the automated pathological archive (PALGA)). Additional sources are the national registry of hospital discharge, which accounts up to 8% of new cases, hematology departments and radiotherapy institutions.”</td></tr>
</table>

Author(s)	Joly, P.; Baricault, S.; Sparsa, A.; Bernard, P.; Bedane, C.; Duvert-Lehembre, S.; Courville, P.; Bravard, P.; Remond, B.; Doffoel-Hantz, V.; Benichou, J.
Titel	Incidence and Mortality of Bullous Pemphigoid in France
Year of publication	2012[9]
Review Question (1/2/3)	1/2
Aim/purpose/objective	• Reevaluate the incidence of Bullous Pemphigoid (BP) in France (compared to 15 years ago)<br>• Assess mortality of BP patients.
Design	Secondary data analysis
Population (Inclusion/Exclusion Criteria)	Data from pathology laboratories in Haute- Normandie, Limousin, and Champagne-Ardennes, France<br>Inclusion:<br>• "Patient living in one of the three regions during the time of the study with newly diagnosed bullous pemphigoid based on clinical, histological, and immunological criteria"<br>Exclusion:<br>• Biopsies from patients living outside the three regions during the study period
Methods	• Skin biopsy databases of pathology laboratories (three university hospitals, four general hospitals, four private-practice laboratories) of the three regions were consulted<br>• Additionally, the three pathology laboratories of the university hospitals are reference centers for the corresponding regions and exclusively performing direct immunofluorescence microscopy (listing was used)<br>• Reviewing medical records<br>• Study period from January 2000 to December 2005<br><u>Mortality data</u><br>• Data from a prospective BP cohort (incl. Jan 2000 to Dec 2003), actively followed up during a 12-month period in 25 dermatology departments from all 22 geographical regions of continental France
Sample characteristics	**1.**<br>• n = 502 incident BP patients in total included<br>• Mean age at diagnosis: 82.6 ± 8.8 years (Range 49 to 106 years)<br>**2.**<br>• n = 312 patients were followed up (12-month)<br>• Median age: 82.1 ± 9.7years (Range 20 to 100 years)
Key findings relevant for review questions	**1.**<br>• 92% of incident BP cases were ≥ 70 years<br>• 46% of incident BP cases were ≥ 85 years<br>• Incidence increased sharply with age<br><br>Incidence of BP:<br>➔ Population aged 70 years or above: 162 cases per million inhabitants per year<br>➔ Populations aged 75 years or above: 224 cases per million inhabitants per year<br>➔ Populations aged 80 years or above: 329 cases per million inhabitants per year

	➔ Populations aged 85 years or above: 507 cases per million inhabitants per year<br><br>**2.**<br>Age-specific standardised mortality rate<br><br>Population / No. of deaths over 1 year of follow-up / SMR / 95% CI:<br>Whole BP population (n = 312) / 118 / 6.6 / 5.5; 7.9<br>BP patients aged ≥ 70 years / 114 / 6.5 / 5.4; 7.8<br>BP patients aged ≥ 80 years / 87 / 5.5 / 4.4–6.8<br>BP patients aged ≥ 90 years / 38 / 4.1 / 2.9; 5.6<br><br>• High mortality of BP patients was observed in all age categories
Notes	• "The limited number of pathology laboratories in these three regions allowed easy identification of BP cases."

Population	No. of deaths over 1 year of follow-up	SMR	95% CI
Whole BP population (n = 312)	118	6.6	5.5; 7.9
BP patients aged ≥ 70 years	114	6.5	5.4; 7.8
BP patients aged ≥ 80 years	87	5.5	4.4–6.8
BP patients aged ≥ 90 years	38	4.1	2.9; 5.6

<table>
<tr><td>Author(s)</td><td>Bonaccorsi G., Lorini C., Santomauro F., Rasero L., Musco S., Del Popolo G.</td></tr>
<tr><td>Titel</td><td>Impact of different pads in elderly assisted in home care</td></tr>
<tr><td>Year of publication</td><td>2013[10]</td></tr>
<tr><td>Review Question (1/2/3)</td><td>1/3</td></tr>
<tr><td>Aim/purpose/objective</td><td>• To assess the potential different impact of two kinds of pads in the prevention of skin complication, in a large cohort of incontinent elderly assisted in home care</td></tr>
<tr><td>Design</td><td>Longitudinal study
Interventional</td></tr>
<tr><td>Population (Inclusion/Exclusion Criteria)</td><td>Inclusion:
• ≥ 65 years old
• Assisted at home
• Severe urinary incontinence
• Wearing pads provided by Local Health Unit</td></tr>
<tr><td>Methods</td><td>• 40 days follow up
• Four examinations at home by skilled nurses
→ Braden scale score (‘ulcer risk’)
→ NPUAP classification system (‘stage of skin pressure sores’ in incontinence care area)
→ subjective nursing evaluations (moisture, maceration, desquamation)
→ quesionnaires assessing opinion (of carer and subject) on products and if other incontinence care products were used simultaniously</td></tr>
<tr><td>Sample characteristics</td><td>• n = 377 (completed all four examinations; 200 A arm, 177 B arm)
• 78.2% female
• Mean age: 85.4±7.6</td></tr>
<tr><td>Key findings relevant for review questions</td><td>1.
• Pressure ulcer prevalence in arms: 22.5%/22.6%
• Pressure ulcer incidence (in whole ad-risk sample (no ulcer at first examination)): 6.2%
3.
A arm (‘innovative underwear belted-diaper’ with hook and loop fastening)

B arm (‘standard absorbent cellulose double-layer underwear brief-diaper’ with rubber bands and movable adhesive tapes)

Nurse evaluations on skin conditions
<table>
<tr><td>Examination</td><td>1</td><td>2</td><td>3</td><td>4</td></tr>
<tr><td>Arm</td><td>A n(%)
B n(%)</td><td>A n(%)
B n(%)</td><td>A n(%)
B n(%)</td><td>A n(%)
B n(%)</td></tr>
<tr><td>Wet skin</td><td>A 90 (46.4)
B 126 (72.0%)</td><td>A 13 (6.6)
B 118 (69.4)</td><td>A 16 (8.0)
B 119 (69.9)</td><td>A 12 (6.2)
B 119 (69.6)</td></tr>
<tr><td>Scratching lesions</td><td>A 16 (8.9)
B 4 (2.5)</td><td>A 6 (3.3)
B 6 (3.3)</td><td>A 3 (1.6)
B 5 (3.0)</td><td>A 2 (1.1)
B 2 (1.2)</td></tr>
</table></td></tr>
</table>

<table>
<tr>
<td></td>
<td>
<table>
<tr><td>Maceration</td><td>A 7 (3.9)
B 11 (6.6)</td><td>A 2(1.1)
B 11
(6.6)</td><td>A 1 (0.5)
B 15 (8.8)</td><td>A 0
(0.0)
B 14
(8.4)</td></tr>
<tr><td>Desquamation</td><td>A 0 (0.0)
B 6 (3.7)</td><td>A 0 (0.0)
B 2 (1.2)</td><td>A 0 (0.0)
B 6 (3.6)</td><td>A 0
(0.0)
B 3
(1.9)</td></tr>
</table>

Braden scale score by risk classes: No statistically sign. difference
Percentage of people using additional products decreased in A group statistically (5%) compared to B group
Trends in questionnaires positively related to product A

</td>
</tr>
<tr>
<td>Notes</td>
<td>

Conference abstract
Unclear reporting (pads, diaper, where was study conducted)
PU scales only
Biased – sponsor? Blinded?

</td>
</tr>
</table>

Author(s)	Danielsen, K.; Olsen, A. O.; Wilsgaard, T.; Furberg, A. S.
Titel	Is the prevalence of psoriasis increasing? A 30-year follow-up of a population-based cohort
Year of publication	2013[11]
Review Question (1/2/3)	1
Aim/purpose/objective	• To analyse trends in the prevalence of psoriasis over the past 30 years, separating age, birth cohort and time period effects
Design	Longitudinal study
Population (Inclusion/Exclusion Criteria)	Inhabitants in the municipality of Tromsø, Norway, 69° N<br>Inclusion:<br>• Aged 20–79 years
Methods	• Time period 1979 to 2008 was analysed<br>• Five surveys included (T2 (1979-1980) , T3, T4, T5, T6 (2007-2008))<br>• Surveys consisted of two general questionnairs (one enclosed in invitation letter, one handed out at screening)
Sample characteristics	n = 33,387 unique individuals born between 1915 and 1977
Key findings relevant for review questions	• Prevalence of psoriasis increased with each consecutive survey in all examined age groups and birth cohorts<br>• "Within each birth cohort the self-reported prevalence increased by time period (i.e. age)."<br>• Percentage of subjects with self-reported psoriasis increased from birth cohort 1910-1919 to birth cohort 1930-1939 from app. 7% to 12% respectively (Age group 70-79)<br>• Percentage of subjects with self-reported psoriasis increased from birth cohort 1910-1919 to birth cohort 1930-1939 from app. 8% to 12% respectively (Age group 60-69)
Notes	• Percentage of subjects with self-reported psoriasis interpreted from figure 1

Author(s)	Etzkorn, J. R.; Parikh, R. P.; Marzban, S. S.; Law, K.; Davis, A. H.; Rawal, B.; Schell, M. J.; Sondak, V. K.; Messina, J. L.; Rendina, L. E.; Zager, J. S.; Lien, M. H
Titel	Identifying risk factors using a skin cancer screening program
Year of publication	2013[12]
Review Question (1/2/3)	1
Aim/purpose/objective	• "This study details the relationship of a group of well-known risk factors with presumptive diagnoses in a large series of individuals self-referred for free skin cancer screening."
Design	Cross-sectional
Population (Inclusion/Exclusion Criteria)	Visitors at public events across Florida and Puerto Rico that offered skin cancer screening by the Mole Patrol™ (Moffitt Cancer Center, Tampa, FL) from 2007 to 2010
Methods	• Free skin cancer screening offered at public events across Florida and Puerto Rico, including sporting events, popular beaches, skin cancer screening training events, military-associated events, and community fairs and outdoor festivals<br>• Focused medical history via standardized form<br>• Screening conducted by volunteer physicians (dermatologists, surgical oncologists, and dermatopathologists), nurse practitioners and physician assistants (who worked at dermatology or cutaneous oncology practices)
Sample characteristics	• Screened people ≥ 60 years: n = 1,790
Key findings relevant for review questions	Frequency of Nonmelanoma Cancer Findings (participants with presumptive diagnoses of AK, SCC, and BCC) by age:<br>Age (years) / No. of participants / Frequency (%)<br>60-69 / 1,202 / 372 (30.9)<br>70-79 / 501 / 220 (43.9)<br>≥ 80 / 87 / 43 (49.4)<br><br>Frequency of Suspicious Pigmented Lesion Findings (presumptive diagnosis of atypical mole and melanoma) by age:<br>Age (years) / No. of participants / Frequency (%)<br>60-69 / 1,202 / 93 (7.7)<br>70-79 / 501 / 30 (6.0)<br>≥ 80 / 87 / 3 (3.4)
Notes	-

Frequency of Nonmelanoma Cancer Findings (participants with presumptive diagnoses of AK, SCC, and BCC) by age:

Age (years)	No. of participants	Frequency (%)
60-69	1,202	372 (30.9)
70-79	501	220 (43.9)
≥ 80	87	43 (49.4)

Frequency of Suspicious Pigmented Lesion Findings (presumptive diagnosis of atypical mole and melanoma) by age:

Age (years)	No. of participants	Frequency (%)
60-69	1,202	93 (7.7)
70-79	501	30 (6.0)
≥ 80	87	3 (3.4)

<table>
<tr><td>Author(s)</td><td>Flohil, S. C.; van der Leest, R. J. T.; Dowlatshahi, E. A.; Hofman, A.; de Vries, E.; Nijsten, T</td></tr>
<tr><td>Titel</td><td>Prevalence of actinic keratosis and its risk factors in the general population: the Rotterdam Study</td></tr>
<tr><td>Year of publication</td><td>2013[13]</td></tr>
<tr><td>Review Question (1/2/3)</td><td>1</td></tr>
<tr><td>Aim/purpose/objective</td><td>To investigate the prevalence of actinic keratosis (AK) and its associated risk factors</td></tr>
<tr><td>Design</td><td>Cross-sectional
(Data from longitudinal study (Rotterdam Study))</td></tr>
<tr><td>Population (Inclusion/Exclusion Criteria)</td><td>Inhabitants of the Ommoord district of Rotterdam, Netherlands
Inclusion:
>50 years of age</td></tr>
<tr><td>Methods</td><td>Full body skin examination (FBSE)
→ Exception of the feet and the skin covered by socks and underwear, respectively
→ Conducted by four trained physicians
Extrapolated prevalence for general dutch population (>50 years)
→ Population size from Statistics Netherlands</td></tr>
<tr><td>Sample characteristics</td><td>n = 2,061 (total)
n = 1,187 (≥ 70)
Female sex (55.0%).
Mean age at the date of FBSE: 71.6 years (SD 7.1; ranging from 51 to 98 years).</td></tr>
<tr><td>Key findings relevant for review questions</td><td>
<table>
<tr><th></th><th>Total study population n = 2,061</th><th>No AK (%) (n = 1,288)</th><th>1–3 AKs (%) (n = 433)</th><th>4–9 AKs (%) (n= 177)</th><th>≥ 10 AKs (%) (n = 163)</th></tr>
<tr><td>Mean age in years (SD) at FBSE</td><td>71.6 (7.1)</td><td>70.2 (7.2)</td><td>73.0 (6.4)</td><td>74.1 (6.5)</td><td>75.6 (6.2)</td></tr>
<tr><td>< 70</td><td>874 (42.4)</td><td>638 (49.5)</td><td>156 (36.0)</td><td>50 (28.2)</td><td>30 (18.4)</td></tr>
<tr><td>70–79</td><td>947 (45.9)</td><td>532 (41.3)</td><td>219 (50.6)</td><td>98 (55.4)</td><td>98 (60.1)</td></tr>
<tr><td>≥ 80</td><td>240 (11.6)</td><td>118 (9.2)</td><td>58 (13.4)</td><td>29 (16.4)</td><td>35 (21.5)</td></tr>
</table>
</td></tr>
<tr><td>Notes</td><td>-</td></tr>
</table>

Actinic keratosis (AK)
Full body skin examination (FBSE)

Author(s)	Y. Okuno; Y. Takao; Y. Miyazaki; F. Ohnishi; M. Okeda; S. Yano; H. Kumihashi; Y. Gomi; K. Maeda; T. Ishikawa; Y. Mori; H. Asada; H. Iso; K. Yamanishi
Titel	Assessment of skin test with varicella-zoster virus antigen for predicting the risk of herpes zoster
Year of publication	2013[14]
Review Question (1/2/3)	3
Aim/purpose/objective	• To clarify the incidence of and predictive and immunological factors for herpes zoster in a defined community-based Japanese population
Design	Cross-sectional
Population (Inclusion/exclusion Criteria)	Resindents in Shozu County, Kagawa Prefecture enrolled in study B of the SHEZ Study*.<br>Inclusion:<br>• ≥ 50 years
Methods	• Subjects received a varicella-zoster virus (VZV) skin test with VZV antigen; 48h later erythema and oedema were assessed.<br>• Participants completed questionnaires on Herpes zoster history.
Sample characteristics	• n = 5683<br>• Age ≥ 50 years<br>• Women: 54.7%
Key findings relevant for review question(s)	• Age-specific frequency (%) distribution of erythema and oedema diameters by the Varicella virus skin test: (see table below)

	0	0.01–4.99	5.00–9.99	≥ 10.00
Erythema diameter (mm)				
60-69	10.2	5.7	13.3	70.8
70-79	17.1	8.7	16.3	57.9
≥ 80	25.2	12.2	14.7	47.9
Oedema diameter (mm)				
60-69	27.6	5.6	27.1	39.7
70-79	37.8	7.4	26.7	28.1
≥ 80	53.0	6.3	22.4	18.3

Means of Erythema and Oedema diameter (mm) for subjects who developed Herpes zoster within a year after registration**:

	Development of Herpes Zoster	
	Yes	No
Erythema diameter (mm)	7 mm	14 mm
Oedema diameter (mm)	3 mm	7mm

- “The longest diameter for both erythema and oedema clearly decreased with increasing age”
- “…results suggest that the skin test reaction caused by the VZV antigen is a promising indicator for predicting the development of herpes zoster.”

Notes	*Consisting of residents in Shozu County aged 50 years or older on 1 October 2008<br>** Data were extracted from diagrams.

Varicella-zoster virus (VZV)

Author(s)	Robsahm, T. E.; Bergva, G.; Hestvik, U. E.; Moller, B.
Titel	Sex differences in rising trends of cutaneous malignant melanoma in Norway, 1954-2008
Year of publication	2013[15]
Review Question (1/2/3)	1
Aim/purpose/objective	• To present the incidence trends of cutaneous malignant melanoma (CMM) in Norway 1954–2008, according to period, sex, age, stage of disease, anatomical location, and geographical regions.<br>• To discuss the results in relation to sun exposure habits and sun awareness over time
Design	Analysis of registry data
Population (Inclusion/exclusion Criteria)	Residents of Norway
Methods	• All new cases of invasive CMM diagnosed between 1 January 1954 and 31 December 2008 were retrieved from the Cancer Registry of Norway*<br>• Calculation of incidence rates according to sex, age, stage of disease, primary anatomical site of the tumour and by geographical region, annual percentage change and age-period-cohort model<br>• Age-adjusted incidence rates based on direct standardization according to the European Standard Population
Sample characteristics	• n = 31,783 cases of CMM (all age groups)
Key findings relevant for review question(s)	• The incidence rate of CMM in men and women aged over 70 years, during the period 1954–2008, was higher compared to the incidence rates in the other age groups (<30, 30-49 and 50-69 years old).<br>• “The most pronounced increase in rate was observed in men, older than 70 years, showing an annual percentage increase of 5.2.” (1954-2008)<br>• Women (70+): APC (1954 – 2008) 3.9<br>• “For all melanomas combined, the relative cohort-specific risk increased considerably up to around 1945, after which it levelled off in men. For women, the cohort-specific rates continued to increase also in the younger cohorts.”<br><br>Incidence (2004-2008)<br>• Age 70+ years: 100/100,000 (male)<br>• Age 70+ years: 60/100,000 (female)<br>-> interpreted from figure 2
Notes	*The Cancer Registry of Norway has registered all cancer cases nationwide since its establishment in 1953

Cutaneous malignant melanoma (CMM)

Author(s)	Ashley Wysong, Eleni Linos, Tina Hernandez-Boussard, Sarah T. Arron, Hayes Glandstone, Jean Y. Tang,
Titel	Nonmelanoma Skin Cancer Visits and Procedure Patterns in a Nationally Representative Sample: National Ambulatory Medical Care Survey 1995-2007
Year of publication	2013[16]
Review Question (1/2/3)	2
Aim/purpose/objective	• To evaluate practice and treatment patterns of non-melanoma skin cancer (NMSC) in the United States over the last decade.<br>• To characterize differences according to sex, age, race, insurance type, and physician specialty.
Design	Secondary data analysis
Population (Inclusion/exclusion Criteria)	Data from the National Ambulatory Medical Care Survey (NAMCS)<br>Inclusion:<br>• ≥ 18 years<br>• ICD-9-CM codes 173.0–173.9, 232.0–232.9
Methods	• Cross sectional analysis of NAMCS data from 1995 to 2007<br>• Only outpatient visits included<br>• Benign skin conditions were not included<br>• No imputations on missing data<br>• Population-adjusted number of NMSC visit rates per 1,000 persons were calculated using U.S. Census Data.
Sample characteristics	**Physician visits due to NMSC**<br>• All patients with skin cancer diagnosis: n = 61,037,814<br>• Mean age: 68<br>• Male: n = 35,277,686, mean age: 69.2 ± 13<br>• Female: n = 25,760,128, mean age: 68.4 ± 15
Key findings relevant for review question(s)	Number of NMSC visits per 1,000 persons (population-adjusted rate)<br>• Males >65 years (fitted): app. 160/1000 persons (2007)<br>• Females >65 years (fitted): app. 60/1000 persons (2007)<br>• Significant increase in men (annual percentage change (APC)+5.23, p=.02), particularly those aged 65 and older (APC+4.80, p=.01)<br>• Visits in women aged 65 and older remained relatively stable (APC+0.95, p=.66).
Notes	• Rates and n are physician visits due to NMSC<br>NAMCS<br>• “Annual national survey of office visits made by ambulatory patients to a sample of approximately 1500 non-federally employed physicians”<br>• Medical practices (including dermatologists and nondermatologists) are selected based on multistage probability sampling techniques to yield a nationally representative sample<br>• Data extrapolated to make results applicable for entire US population

Non-melanoma skin cancer (NMSC)
Ambulatory Medical Care Survey (NAMCS)

<table>
<tr><td>Author(s)</td><td>Gontijo Guerra, S.; Vasiliadis, H. M.; Preville, M.; Berbiche, D.;</td></tr>
<tr><td>Titel</td><td>Skin conditions in community-living older adults: prevalence and characteristics of medical care service use</td></tr>
<tr><td>Year of publication</td><td>2014a[17]</td></tr>
<tr><td>Review Question (1/2/3)</td><td>1/2</td></tr>
<tr><td>Aim/purpose/objective</td><td>

Determine the frequency of skin conditions
Evaluate the agreement between two different data sources of information (self-report versus administrative)
Document medical care service use for skin conditions in a representative sample of community-dwelling older adults
</td></tr>
<tr><td>Design</td><td>Longitudinal study</td></tr>
<tr><td>Population (Inclusion/Exclusion Criteria)</td><td>Quebec’s community-living older adults
Inclusion

Age >65 years
Able to speak and understand French
Without severe/moderate cog. problems according to Mini Mental State Examination

Exclusion:

(Subjects living in Quebec’s northern regions were excluded owing to budgetary limitations – app. 10% of older population lived there in 2005)
</td></tr>
<tr><td>Methods</td><td>

Computer-assisted ESA questionnaire (ESA-Q)
Two face-to-face interviews (12 months apart)
Survey data were linked to individual-level information from R´egie d’assurance Maladie du Qu´ebec [RAMQ] medical services database
</td></tr>
<tr><td>Sample characteristics</td><td>

n = 2,494 (T1)
n = 1,985 (T2)
Mean age (based on weighted sample): 73.8 years (SD 6.02)
57.9% female
</td></tr>
<tr><td>Key findings relevant for review questions</td><td>
1.
<u>1-year prevalence rate physician-diagnosed skin condition (PDSC)</u>
(calculated for the 12-month period prior to (T1) and following (T2) the baseline interview)

(T1) 22.8%
(T2) 20.7%

<u>2-year period prevalence rate PDSC</u>

31.4%
Almost 10% of participants presented a PDSC during T1 and T2

Presence of Skin Condition over 2 yr
<table>
<tr><td>Age</td><td colspan="2">Self-reported</td><td colspan="2">Diagnosed</td></tr>
<tr><td></td><td>467/2,494 (18.7%)</td><td>OR (95% CI)</td><td>782/2,494 (31.4%)</td><td>OR (95% CI)</td></tr>
<tr><td>65-74</td><td>268/1,444 (18.6%)</td><td>1.03 (0.84–1.26)</td><td>459/1,447 (31.7%)</td><td>0.96 (0.81–1.14)</td></tr>
<tr><td>75+</td><td>199/1,047 (19.0%)</td><td></td><td>324/1,047 (30.9%)</td><td></td></tr>
</table>
</td></tr>
</table>

	<u>Prevalence rate of self-reported skin condition (SRSC)</u><br>• Baseline: 13.3%<br>• Follow-up interview: 13.1%<br>• Participants participating in both and reporting SRSC in both: 6.2%<br>**2.**
	Average number of dermatologic-related medical visits:<br>• Participants with a PDSC: 2.37 (SD 5.51) at T1 and 2.52 (SD 5.98) at T2 (p = .069).<br>• Majority dermatologist-visits (almost 60%) of medical care use for skin conditions, followed by GP-visits (23.8 and 29.0%)<br>• Of participants with PDSC at T1, 42.6% consulted exclusively a dermatologist and 31.9% a GP<br>• "Almost 16% of participants with a PDSC consulted more than one category of medical professionals for their skin conditions during T1" (T2 was similar for dermatologists and other non-dermatologist speiclist, but GP decreased significantly)
Notes	-

Physician-diagnosed skin condition (PDSC)

Author(s)	Samantha Gontijo Guerra, Michel Pre´ville, Helen-Maria Vasiliadis, and Djamal Berbiche
Titel	Association between Skin Conditions and Depressive Disorders in Community-Dwelling Older Adults
Year of publication	2014b[18]
Review Question (1/2/3)	2
Aim/purpose/objective	• "Examine the transversal and longitudinal relationship between depressive disorders and skin conditions among a population-based sample of community-living older adults age 65 years and over"
Design	Longitudinal study
Population (Inclusion/Exclusion Criteria)	Older adults in Quebec<br>Inclusion:<br>• French-speaking<br>• Community-dwelling<br>• ≥ 65 years<br>• MMSE ≥ 22
Methods	• Survey<br>• Two in-home face-to-face interviews, 1 year apart<br>• Survey/interview data was linked to RAMQ's database
Sample characteristics	• n = 2,494 T1<br>• n = 1,985 T2<br>• Mean age: 73.8 (SD 6.02)
Key findings relevant for review questions	• Self-reported skin condition (baseline):<br>Yes: n = 331 (13.3%)<br>No: n = 2,157 (86.7%)<br>• 'According to our initial hypothesis, we found evidence for cross-sectional associations between skin conditions and depressive disorders at both time periods.'<br>• 'At time 1, a higher prevalence of a depressive disorder was observed among participants with a ''skin condition'' compared to those without (10.5% vs 5.5%; x^2 = 11.81, p = .001). Similar results were found at time 2 (10.8% vs 5.2%, x^2 = 12.12, p 5 .000).'
Notes	-

Author(s)	Hsieh, Chi-Feng; Huang, Weng-Foung; Chiang, Yi-Ting
Titel	The Incidence of Actinic Keratosis and Risk of Non-Melanoma Skin Cancer in Taiwan
Year of publication	2014[19]
Review Question (1/2/3)	1
Aim/purpose/objective	• Investigate the incidence, the distribution, and the treatment types of actinic keratosis (AK) in Taiwan<br>• Evaluate the risk of AK to malignant neoplasm
Design	Secondary data analysis
Population (Inclusion/Exclusion Criteria)	Data of 2000 Longitudinal Health Insurance Database (LHID)<br>Inclusion:<br>• Individuals with one outpatient visit with a diagnosis of AK of ICD-9-CM code 702.0 identified by dermatologist<br>• At least two outpatient visits or one hospital admission with a diagnosis of AK
Methods	• Time period analysed: 1999 to 2010<br>• Cox proportional hazards model
Sample characteristics	No data
Key findings relevant for review questions	• Incidence rate of AK per 10,000 persons for ≥ 65 (range from 7.76 to 14.04) was higher than for < 65 years old (range from 1.24 to 2.67).<br>• 45.1% were elderly people.
Notes	Conference abstract

Actinic keratosis (AK)

Author(s)	ET Landis, SA Davis, A Taheri, SR Feldman
Titel	Top dermatologic diagnoses by age
Year of publication	2014[20]
Review Question (1/2/3)	2
Aim/purpose/objective	• To analyze data from the National Ambulatory Medical Care Survey (NAMCS) to provide a nationally representative analysis of the most common dermatologic diagnoses seen in the United States stratified by patient age and by both dermatologists and other physicians
Design	Secondary data analyses
Population (Inclusion/Exclusion Criteria)	Dataset of National Ambulatory Medical Care Survey (NAMCS)
Methods	• Time period analysed: 1993-2010<br>• Data from 2000 U.S. Census to calculate visits per 100,000 population for each age group<br>• "…secondary analysis focused on patients with a sole diagnosis of a skin condition presenting to any specialty"
Sample characteristics	No data
Key findings relevant for review questions	Frequency of skin-related visits to leading specialties by age group (interpreted from figure):<br>→ 65-74 years: 15% to Family or General Practitioner, 9% Internal Medicine<br>→ 75-84 years: 15% to Family or General Practitioner, 8% Internal Medicine<br>→ 85+ years: 17% to Family or General Practitioner, 6% Internal Medicine
Notes	• Annual survey of non-federally funded outpatient physicians<br>• Data entered by physicians: subset of or all their patient visits during a randomly assigned one week period<br>• Resulting data is weighted according to known geographic variables to approximate overall values for the entire U.S. population

<table>
<tr><td>Author(s)</td><td>Katherine L. Caretti, Darius R. Mehregan, David A. Mehregan,</td></tr>
<tr><td>Titel</td><td>A survey of self-reported skin disease in the elderly African-American population</td></tr>
<tr><td>Year of publication</td><td>2015[21]</td></tr>
<tr><td>Review Question (1/2/3)</td><td>1/2</td></tr>
<tr><td>Aim/purpose/objective</td><td>Identify prevalences of self-reported skin disease and skin-related concerns in elderly African-Americans
Gain insight into patient perceptions of skin disease and its effect on the seeking of medical care
Assess participants' knowledge and awareness of skin cancer</td></tr>
<tr><td>Design</td><td>Cross-sectional</td></tr>
<tr><td>Population (Inclusion/Exclusion Criteria)</td><td>Self-identified African-American senior citizens from a senior community center in Detroit</td></tr>
<tr><td>Methods</td><td>17-item survey questionaire
Investigator was present to answer questions if needed</td></tr>
<tr><td>Sample characteristics</td><td>n = 101,
Aged 60 to 91 years, median age: 71 years
75 % were female</td></tr>
<tr><td>Key findings relevant for review questions</td><td>1.
Prevalence of skin diseases in the study population (n = 101)
<table>
<tr><th></th><th>Subjects, n (%)</th></tr>
<tr><td>Eczema/dermatitis</td><td>29 (28.7)</td></tr>
<tr><td>Fungal skin infection</td><td>17 (16.8)</td></tr>
<tr><td>Alopecia</td><td>7 (6.9)</td></tr>
<tr><td>Viral skin infection</td><td>5 (4.9)</td></tr>
<tr><td>Urticaria</td><td>5 (4.9)</td></tr>
<tr><td>Keloid</td><td>3 (2.9)</td></tr>
<tr><td>Pseudofolliculitis barbae</td><td>2 (1.9)</td></tr>
<tr><td>Bacterial skin infection</td><td>2 (1.9)</td></tr>
<tr><td>Psoriasis</td><td>1 (0.9)</td></tr>
<tr><td>Seborrheic dermatitis</td><td>1 (0.9)</td></tr>
<tr><td>Lupus</td><td>1 (0.9)</td></tr>
<tr><td>Rosacea</td><td>1 (0.9)</td></tr>
<tr><td>Basal cell carcinoma</td><td>1 (0.9)</td></tr>
</table>
2.
Prevalence of skin complaints in the study population (n = 101)
<table>
<tr><th></th><th>Subjects, n (%)</th></tr>
<tr><td>Dry skin/pruritus</td><td>41 (40.6)</td></tr>
<tr><td>Moles</td><td>28 (27.7)</td></tr>
<tr><td>Hair loss</td><td>26 (25.7)</td></tr>
<tr><td>Skin discoloration</td><td>21 (20.8)</td></tr>
<tr><td>Wrinkles</td><td>16 (15.8)</td></tr>
<tr><td>Facial hair</td><td>14 (13.9)</td></tr>
<tr><td>Rashes</td><td>14 (13.9)</td></tr>
</table>
Most participants reported multiple skin concerns and that they would not consult a physician because of them.
69.3% reported at least one skin concern</td></tr>
</table>

	• Rashes and dry skin were most likely concern about which a physician would be consulted (20.8% and 17.8%, respectively)
Notes	-

Author(s)	M. Cybulski; E. Krajewska-Kulak
Titel	Skin diseases among elderly inhabitants of Bialystok, Poland
Year of publication	2015[22]
Review Question (1/2/3)	1
Aim/purpose/objective	• To assess the most frequent skin diseases in people over 60 years old among residents of a public nursing home (PNH) and students of the University of the Third Age (U3A) in Bialystok
Design	Cross-sectional
Population (Inclusion/Exclusion Criteria)	Students of U3A in Bialystok, Poland<br>(and residents of a public nursing home)<br>Inclusion:<br>• >60 years of age
Methods	• Diagnostic survey (authors' anonymous survey questionnaire)<br>→ 35 questions (open and closed, one or multiple choice)<br>→ review of patients' dermatological histories<br>• Time period of data collection: April to June 2015
Sample characteristics	• n =100 (39% 61 to 70 years)
Key findings relevant for review questions	Confirmed (dermatologist) skin disease (U3A students): 27.0 %<br>(table below)<br>• "Lesions were most frequently observed on the lower[40.7%] and upper extremities [29.6%] and the face [33.3%]"<br>• "74.1% of U3A participants, visited a dermatologist, but only when it was necessary"<br>• "18.5% of U3A members[…] systematically visited a dermatologist's surgery"
Notes	-

**Skin disease**	**U3A students**
Atopic dermatitis	18.5%
Vitiligo	3.7%
Furuncle	3.7%
Fungal infection	33.3%
Epidermal cyst	7.4%
Lichen planus	3.7%
Dandruff	3.7%
Psoriasis	14.8%
Balding	18.5%
Angioma	3.7%
Rash	3.7%
Shingles	11.1%
Erysipelas	3.7%
Erythema	3.7%
Acne	7.4%
Eczema	7.4%
Granuloma	3.7%

<table>
<tr><td>Author(s)</td><td>Etienne Duim, Felipe Henrique Cardoso de Sá, Yeda Aparecida de Oliveira Duarte, Rita de Cassia Burgos de Oliveira, Maria Lucia Lebrão</td></tr>
<tr><td>Titel</td><td>Prevalence and characteristics of lesions in elderly people living in the community</td></tr>
<tr><td>Year of publication</td><td>2015[23]</td></tr>
<tr><td>Review Question (1/2/3)</td><td>1</td></tr>
<tr><td>Aim/purpose/objective</td><td>Describe the profile and characteristics of community-dwelling older people with mobility restrictions and skin lesions</td></tr>
<tr><td>Design</td><td>Cross-sectional
(Data of longitudinal study (SABE study))</td></tr>
<tr><td>Population (Inclusion/Exclusion Criteria)</td><td>Cohorts A,B,C of SABE Study (Health, Welfare and Aging)
Inclusion:
• Community-dwelling older people from São Paulo
• ≥ 60 years
• Mobility restrictions (bedridden or wheelchair)</td></tr>
<tr><td>Methods</td><td>• Standardized interviews with trained staff
→ Sociodemographic data
→ Skin lesions
→ Chronic diseases
→ Use of health services
• Blood biomarkers
• Analyses of associations via univariate analysis</td></tr>
<tr><td>Sample characteristics</td><td>• n = 1,344
• 56.6% women
• Aged between 60 and 104
• Median age: 85</td></tr>
<tr><td>Key findings relevant for review questions</td><td>(presented results are weighted in order to represent elderly in São Paulo in year of interest)

20.7% of older people with mobility restrictions had skin lesions
Most common sites were
• sacral region (both sexes)
• scapular region (women)
• trochanteric region (men)

Distribution (%) of elderly people according to socio-demographic variables and the presence of skin lesions in the city of São Paulo, 2010:
<table>
<tr><th>Age</th><th>Skin lesions yes (%)</th><th>Skin lesions No (%)</th><th>Skin lesion Total (%)</th></tr>
<tr><td>60-69</td><td>30.6</td><td>69.4</td><td>100.0</td></tr>
<tr><td>70-79</td><td>0.0</td><td>100.0</td><td>100.0</td></tr>
<tr><td>80 and above</td><td>27.2</td><td>72.8</td><td>100.0</td></tr>
<tr><td>Total</td><td>20.7</td><td>79.3</td><td></td></tr>
</table></td></tr>
<tr><td>Notes</td><td>• Results and sample not comprehensible. Unclear compositions, unclear proportions and what they relate to.</td></tr>
</table>

Author(s)	RJ Hay; LC Fuller
Titel	Global burden of skin disease: a grand challenge to skin health
Year of publication	2015[24]
Review Question (1/2/3)	2
Aim/purpose/objective	• Assess the burden of skin diseases worldwide
Design	Secondary data analysis
Population (Inclusion/Exclusion Criteria)	World population
Methods	• Two stage literature review (incl. medical literature databases, national and regional health surveys, personal communications of unpublished data)<br>• Lead in data collection: GBD 2010 Skin Conditions Expert Group<br>• Vital registration with medical certification of causes of death<br>• Verbal autopsy data<br>• National or regional cancer registries<br>• No estimation of quality-of-life impact included
Sample characteristics	No data
Key findings relevant for review questions	DALYs lost by age per 100,000 (see table below)

DALYs lost by age per 100,000

**Disease**	**m/f 60 – 64**	**m/f 70-74**	**m/f 75-79**	**m/f 80+**
Eczema	96.1/119	93.9/116.2	93/115.1	92.8/113.8
Alopecia areata	16.3/26.5	15.7/25.5	15.3/25	14.7/24
Pruritus	46.6/62	53.6/71.7	68.6/91.9	95.6/131.4
Urticaria	31.8/49.2	30/46.6	29./45.2	27.5/42.7
Decubitus ulcer	45.1/36.5	101/90.9	150.3/133.4	211.2/209.3
Psoriasis	37.8/30.9	37.4/30.2	35/28.4	31.5/25.8
Cellulitis	33.5/25.3	40/34.4	50.5/37.1	55.1/41.1
Abscess, impetigo	45.1/37	57.5/49.6	67.1/54	73.7/62.2
Scabies	16.7/16.1	13.5/12.7	11.6/10.9	8.5/7.4
Fungal skin disease	56.6/51.6	54.9/49.8	51.1/46.1	44.8/39.1
Viral skin disease	21.8/20.4	21.5/20.3	21.4/20.3	21.4/20.6
Other skin diseases	194.5/144.3	271.3/177.8	315.1/199.1	358.2/223.1

Global death rates for non-melanoma skin cancer/melanoma/cellulitis/other bacterial skin infection (2010)

→ 70-74 years: 3.2/5.8/2.3/3.0

→ 75-79 years: 3.8/5.6/2.3/3.2

→ 80+ years: 12.6/10.9/4.5/7.4

- "The burden of NMSC rises by age but is significant in all ages over the age of 70. However, there is a more steeply increasing trend in this burden in those over 80 years."
- "Other key diseases with increasing impact in older age groups are pruritus, cellulitis and abscess."

Notes	-

Author(s)	V. Kiiski; P. Susitaival; A. Remitz; S. Reitamo
Title	Is atopic dermatitis more persistent than previously estimated?
Year of publication	2015[25]
Review Question (1/2/3)	1
Aim/purpose/objective	• To evaluate 12-month prevalence, total adulthood prevalence, and lifetime prevalence of atopic dermatitis (AD) in adults.
Design	Cross-sectional
Population (Inclusion/exclusion Criteria)	Nationwide health examination survey 'Health 2000'<br>Inclusion:<br>• Age ≥ 30 years
Methods	• Interviews<br>• Comprehensive clinical examination
Sample characteristics	• n = 8,028 (≥ 30 years)
Key findings relevant for review question(s)	• 12-month prevalence of AD for subjects (≥70): 5.6%<br>• Prevalence of adulthood AD ≥70: 9.5%<br>• In subjects with history of AD: 38% ≥ 70 years stay sympthomatic
Notes	• Population-based data from nationwide health examination survey 'Health 2000', carried out in 2000-2001, providing a representative sample of the general Finnish adult population<br>• Conference abstract

Atopic dermatitis (AD)

<table>
<tr><td>Author(s)</td><td>Lucia Romani, Andrew C Steer, Margot J Whitfeld, John M Kaldor</td></tr>
<tr><td>Titel</td><td>Prevalence of scabies and impetigo worldwide: a systematic review</td></tr>
<tr><td>Year of publication</td><td>2015[26]</td></tr>
<tr><td>Review Question (1/2/3)</td><td>1</td></tr>
<tr><td>Aim/purpose/objective</td><td>To assess the prevalence of scabies infection and impetigo worldwide.</td></tr>
<tr><td>Design</td><td>Secondary data analysis</td></tr>
<tr><td>Population (Inclusion/exclusion Criteria)</td><td>Inclusion:
Population-based studies, reporting on the prevalence of scabies and impetigo
Community-setting
Worldwide
No age limits</td></tr>
<tr><td>Methods</td><td>Systematic review</td></tr>
<tr><td>Sample characteristics</td><td>Included studies n = 48
Including samples up to n = 18,000 people</td></tr>
<tr><td>Key findings relevant for review question(s)</td><td>Scabies prevalence per country:
<table>
<tr><th>Country</th><th>Age*(years)</th><th>Prevalence (%)</th></tr>
<tr><td rowspan="2">Fiji</td><td>70</td><td>20%</td></tr>
<tr><td>67</td><td>15%</td></tr>
<tr><td>Vanuatu</td><td>70</td><td>10%</td></tr>
<tr><td rowspan="2">Brazil</td><td>70</td><td>5%</td></tr>
<tr><td>75</td><td>5%</td></tr>
<tr><td>Panama</td><td>66</td><td>10%</td></tr>
<tr><td>Solomom islands</td><td>70</td><td>0%</td></tr>
<tr><td>Timor-Leste</td><td>70</td><td>15%</td></tr>
</table>
*Reported as “age in the middle of each reported age group”.</td></tr>
<tr><td>Notes</td><td>Scabies prevalences were extracted from a graph (Figure 5).</td></tr>
</table>

Author(s)	Cinotti, E.; Perrot, J. L.; Labeille, B.; Biron, A. C.; Vierkotter, A.; Heusele, C.; Nizard, C.; Schnebert, S.; Barthelemy, J. C.; Cambazard, F.;
Titel	Skin tumours and skin aging in 209 French elderly people: the PROOF study
Year of publication	2016[27]
Review Question (1/2/3)	1
Aim/purpose/objective	• Evaluate global skin aging and specific aspects of intrinsic and extrinsic skin aging.<br>• Evaluate the prevalence of skin tumours (in an elderly French cohort).
Design	Cross-sectional
Population (Inclusion/exclusion Criteria)	PROOF (PROgnostic indicator OF cardiovascular and cerebrovascular events) cohort<br>Inclusion:<br>• Aged ≥ 65 years (when originally recruited in 2001-2003)<br>• Still alive 2013-2014 (present study)<br><br>Exclusion Criteria, PROOF study:<br>previous myocardial infarction, arrhythmia, cardiac pacemaker, stroke, neurological or psychiatric disease, insulin-dependent diabetes, chronic obstructive pulmonary disease, cerebral magnetic resonance results suggesting neurological diseases or initial dementia, and residence in institutions<br><br>+ Exclusion criteria, present study:<br>(1) history of aesthetic medical treatments<br>(2) application of make-up/any cosmetic product on the skin on the day of the examination
Methods	• Evaluation of skin aging and skin cancer<br>→ Independently by three dermatologists (face-to-face examination)<br>→ Whole skin surface was examined<br>→ SCINEXA score for degree of skin aging and prevalence of skin cancers<br>→ Clinically suspected malignent skin cancers were excised to confirm<br>→ Cutanous paramenters (e.g. Fitzpatrick classification, nevi, benign skin cancers)<br>• Mean value cutaneous parameter from scores made by the three investigators<br>• Assessment over three weeks in March/Nov 2013; March 2014 (subjects were assessed one time in these three weeks)
Sample characteristics	• n = 209 subjects (105 women and 104 men)<br>• Mean age: 77.5 (range: 74 to 81 years)

<table>
<tr><td>Key findings relevant for review question(s)</td><td>Prevalence of benign skin tumours (rate, n = 209)
<table>
<tr><th></th><th>Total body (No of subjects with lesions)</th><th>< 10 lesions</th><th>10-50 lesions</th><th>>50 lesions</th></tr>
<tr><td>Dermal naevus</td><td>70</td><td>57</td><td>13</td><td>0</td></tr>
<tr><td>Seborrheic keratosis</td><td>91</td><td>33</td><td>34</td><td>24</td></tr>
<tr><td>Cherry angioma</td><td>96</td><td>25</td><td>33</td><td>37</td></tr>
<tr><td>Acrochordon</td><td>73</td><td></td><td></td><td></td></tr>
<tr><td>Actinic Lentigo</td><td>99</td><td>0</td><td>14</td><td>85</td></tr>
</table>

Malignant skin tumours identified in skin examination were mainly basal cell carcinoma (11/14)
→ 1/14 squamous cell carcinoma
→ 2/14 melanoma
69.4% of subjects had AK (more frequently in men)
</td></tr>
<tr><td>Notes</td><td>-</td></tr>
</table>

Author(s)	Hsieh, C. F.; Chiang, Y. T.; Chiu, H. Y.; Huang, W. F.
Titel	A Nationwide Cohort Study of Actinic Keratosis in Taiwan
Year of publication	2016[28]
Review Question (1/2/3)	1
Aim/purpose/objective	• Investigate the incidence, the treatment pattern, the medical utilization and the risk of malignant neoplasm of the actinic keratosis (AK) patients in Taiwan
Design	Secondary data analysis
Population (Inclusion/Exclusion Criteria)	National Health Insurance Research Database (NHIRD)
Methods	• Analysis of national health insurance data<br>• Patients who had at least two outpatient visits or one hospital admission of AK (ICD-9-CM) code (702.0) identified by dermatologist were included<br>• Excluded patients who developed skin malignant neoplasm (ICD-9 code: 173) before the entry date<br>• Analysed period: first diagnosis 01.01 2004 to 31.12. 2011 (or 2003 to 2011)
Sample characteristics	• n = 35,933 patients with AKs<br>• n = 17,004 elderly patients (≥ 65 years of age)
Key findings relevant for review questions	Incidence rate for AKs from 2004 to 2011:<br>• Ranged from 1.66 to 2.18 per 10,000 people (all age groups)<br>• Highst incidence rate in elderly<br>• Among elderly patients it ranged from 7.22 to 10.59<br>• "Trend of decreasing incidence of actinic keratosis in elderly patients was also found."<br><br>Skin malignant neoplasm after AK diagnosis/during follow-up:<br>• No (%) of new cases in 17,004 elderly patients: 826 (4.86)<br>• Incidence per 10,000 patient-year [95%CI]: 122.66 [114.58 to 131.32]
Notes	• Database (NHIRD) covered 99% of nearly 23 million people in Taiwan<br>• Unclear reporting regarding time period assessed.

Actinic keratosis (AK)

Author(s)	T. Liu; R. Brienza
Title	What brings an olderveteran to an urgent visit (UV)?A review of the chief concerns by veterans aged 65 and older who presented for UV during a 6-month period at the west haven veterans affairs center of excellence in primary care education (VA COEPCE), an interprofessional academic patient aligned care team (PACT)
Year of publication	2016[29]
Review Question (1/2/3)	2
Aim/purpose/objective	• To investigate the causes of veterans' urgent visits
Design	Secondary data analysis
Population (Inclusion/exclusion Criteria)	Veterans who presented to the West Haven VA COEPCE, emergency departments for an urgent visit
Methods	• Review of urgent visits of older veterans (age ≥ 65 years)<br>• Time period analysed: 01.04.2014 to 30.09.2023
Sample characteristics	• n = 388<br>• 31% ≥ 80 years
Key findings relevant for review question(s)	• Dermatologic concerns comprised 6% of the urgent visits.
Notes	• Conference abstract

<table>
<tr><td>Author(s)</td><td colspan="6">F. Trautmann, F. Meier, A. Seidler and J. Schmitt</td></tr>
<tr><td>Titel</td><td colspan="6">Effects of the German skin cancer screening programme on melanoma incidence and indicators of disease severity</td></tr>
<tr><td>Year of publication</td><td colspan="6">2016[30]</td></tr>
<tr><td>Review Question (1/2/3)</td><td colspan="6">1/3</td></tr>
<tr><td>Aim/purpose/objective</td><td colspan="6">“To examine the uptake and effects of the German nationwide screening programme:
• What is the utilization of the German skin cancer screening programme in the federal state of Saxony?
• What is the incidence of skin cancer before and after the introduction of the screening programme in July 2008?
• How do disease severity and indicators of prognosis vary in patients with melanoma upon their participation in the skin cancer screening programme?”</td></tr>
<tr><td>Design</td><td colspan="6">Secondary data analysis</td></tr>
<tr><td>Population (Inclusion/exclusion Criteria)</td><td colspan="6">Database of a German health insurance company in Saxony</td></tr>
<tr><td>Methods</td><td colspan="6">• Cases of melanoma and non-melanoma skin cancer (NMSC) identified using an algorithm based on the International Classification of Diseases, 10th Revision
• Only individuals continuously insured 01 Jan. 2005 to 31. Dec. 2012 included
• ICD-10-GM codes: C43 for malignant melanoma and C44 for NMSC
• “incident”: preliminary skin cancer-free time interval was ≥ 2 years
• Cross-sectional and longitudinal analyses to determine the utilization of the screening programme as performed in Germany and effects on skin cancer incidence and disease severity</td></tr>
<tr><td>Sample characteristics</td><td colspan="6">n = 686,899</td></tr>
<tr><td rowspan="7">Key findings relevant for review question(s)</td><td colspan="6">1.</td></tr>
<tr><td rowspan="2">Age (years)</td><td rowspan="2">Total n</td><td colspan="2">Melanoma</td><td colspan="2">NMSC</td></tr>
<tr><td>N (%)</td><td>Prevalence %</td><td>N (%)</td><td>Prevalence %</td></tr>
<tr><td>60–69</td><td>276,484</td><td>647 (18.5)</td><td>0.2</td><td>5606 (18.2)</td><td>2.0</td></tr>
<tr><td>70–79</td><td>260,943</td><td>1084 (30.9)</td><td>0.4</td><td>11 308 (36.7)</td><td>4.3</td></tr>
<tr><td>≥ 80</td><td>149,472</td><td>737 (21.0)</td><td>0.5</td><td>10 274 (33.4)</td><td>6.9</td></tr>
<tr><td colspan="6">• “The average age at diagnosis was 66.2 years for melanoma and 73.6 years for NMSC.”
3.
• “The screening participation was highest in the age group 60–69 years among women (15.7%) and 70-79 years among men (15.5%).”
• “No significant increase in melanoma incidence was found after introduction of the screening programme.”
• “Fewer patients with melanoma within the screening programme were diagnosed with metastasis and/or received interferon alpha</td></tr>
</table>

	treatment compared with patients never participating in the programme.”
Notes	-

Non-melanoma skin cancer (NMSC)

<table>
<tr><td>Author(s)</td><td>Hui-Wen Tseng, Yow-Ling Shiue, Kuo-Wang Tsai, Wei-Chun Huang, Pei-Ling Tang, Hing-Chung Lam</td></tr>
<tr><td>Titel</td><td>Risk of skin cancer in patients with diabetes mellitus: A nationwide retrospective cohort study in Taiwan</td></tr>
<tr><td>Year of publication</td><td>2016[31]</td></tr>
<tr><td>Review Question (1/2/3)</td><td>1</td></tr>
<tr><td>Aim/purpose/objective</td><td>To investigate the risk of overall skin cancers, NMSC, and melanoma among a diabetes mellitus (DM) cohort and non-DM cohort with no history of skin cancer</td></tr>
<tr><td>Design</td><td>Secondary data analyses</td></tr>
<tr><td>Population (Inclusion/exclusion Criteria)</td><td>Longitudinal Health Insurance Database 2000 (LHID2000)</td></tr>
<tr><td>Methods</td><td>Analyses of data from LHID 2000
→ Original claims data of one million individuals randomly sampled from the *National Health Insurance Research Database (NHIRD) from 1996 to 2000
→ Age ≥18 years
DM cohort: newly diagnosed DM: min. 2 outpatient visits and one inpatient hospital admission
Time period analysed: 01.01.2000 to 31.12.2005 (for index date DM)
Complete NHI information
LHID2000 beneficiaries were enrolled from 2000 and followed up until end 2012
One DM cohort and one non-DM cohort was matched one-to-one matched by age, sex, index date, and comorbidities
Skin cancer codes ICD-9 172-173 after index date of DM were identified
Compare differences in incidence and distribution of diseases with regards to immunosuppression status</td></tr>
<tr><td>Sample characteristics</td><td>Diabetes melitus cohort:
– n= 41,898
– Mean age (SD): 57.42 (±14.1)
– ≥60 years: n= 18,318
Non-DM cohort:
– n= 41,898
– Mean age (SD): 57.42 (± 14.1)
– ≥60 years: n= 18,318</td></tr>
<tr><td>Key findings relevant for review question(s)</td><td>1.
Incidence rate ratios of ovarall skin cancer (melanoma and NMSC) in the DM and non-DM cohorts by age and sex subgroups:

For DM cohort:
Incidence rate for <60 years: 0.88/10,000 person-years
Incidence rate for ≥60 years: 6.79/10,000 person-years

For non-DM cohort:
Incidence rate for <60 years: 1.33/10,000 person-years
Incidence rate for ≥60 years: 4.72/10,000 person-years

IRR (95% CI): 1.44 (1.07-1.94); p = 0.02</td></tr>
</table>

Notes	*National Health Insurance Research Database: The National Health Insurance program in Taiwan is a compulsory, single-payer, tax-financed healthcare system that provides health care to 99% of the country's 23.75 million people. The database contains de-identified data to ensure patient anonymity.

Diabetes mellitus (DM)

Author(s)	Alexandridou M, Bollaerts K
Titel	Zoster Vaccine Effectiveness Against Incident Herpes Zoster and Post-Herpetic Neuralgia In Elderly In The UK
Year of publication	2017[32]
Review Question (1/2/3)	3
Aim/purpose/objective	• Assess the vaccine effectiveness (VE) against herpes zoster (HZ) and post-herpetic neuralgia (PHN) in elderly within the total population
Design	Secondary data analysis
Population (Inclusion/Exclusion Criteria)	UK Clinical Practice Research Datalink (CPRD)
Methods	• Subjects from birth cohorts 1943-1946 (routine) and 1934-1937 (catch-up) were identified<br>• Vaccinated subjects were compared to unvaccinated subjects using piecewise Cox regression model
Sample characteristics	• Routine birth cohorts: n = 79,274 subjects<br>• Catch-up cohorts: n = 48,193 subjects
Key findings relevant for review questions	(first 2 years of vaccination)<br>Routine birth cohorts:<br>• VE for HZ of 76.4% (95% CI: 70.6%-81.1%)<br>• VE for PHN of 68.3% (95% CI: 7.4%-89.1%)<br><br>(Subsequent 2+ years)<br>• VE estimates for HZ of 56.1% (95% CI: 29.2%-72.7%)<br><br>Catch-up cohorts:<br>• VE estimates were comparable<br><br>→ Insufficient evidence to determine the VE for other HZ complications
Notes	• Conference Abstract<br>• CPRD: anonymised patient data from a network of GP practices across the UK. Primary care data are linked to a range of other health related data -> longitudinal, representative UK population health dataset (https://cprd.com/)

Vaccine effectiveness (VE)
Herpes zoster (HZ)
Post-herpetic neuralgia (PHN)

Author(s)	N. Asokan, V. G. Binesh
Titel	Cutaneous problems in elderly diabetics: A population-based comparative cross-sectional survey
Year of publication	2017[33]
Review Question (1/2/3)	1/ 2 (RQ2)
Aim/purpose/objective	• Identify skin problems associated with diabetes mellitus among elderly persons in a village in Kerala
Design	Cross-sectional
Population (Inclusion/Exclusion Criteria)	Older people (65+ years) living in Thalikulam/Kerala, India
Methods	• People originally self-identifying as without diabetes were tested and transferred to diabetes group if fasting blood sugar test revealed that they had diabetes mellitus.<br>• Face-to-face study interviews were carried out in special camps arranged close to community<br>• All participants evaluated by one of authors for the presence of skin findings (emphasis on previously known/reported findings in diabetes mellitus)<br>• Recorded in a proforma and classified into categories<br>• Interviews conducted: May 2011 to February 2012
Sample characteristics	• n = 287 persons with diabetes mellitus<br>• n = 275 persons without diabetes mellitus<br>• n = 562 in total<br>• Mean age people with diabetes mellitus 73.45 (±6.72) years, 53.7% female<br>• Mean age persons without diabetes mellitus 73.87 (±6.87) years, 61.2% female
Key findings relevant for review questions	(see table below)

Skin findings	No. of patients with diabetes mellitus (n=287) (%)	No. of patients without diabetes mellitus (n=275) (%)	No. in both groups (n=562) (%)
Neurovascular manifestations	186 (64.8)	138 (50.2)	324 (57.7)
• Numbness of extremities	• 80 (27.9)	• 51 (18.5)	• 131 (23.3)
• Tingling sensation of extremities (RQ2)	• 123 (42.9)	• 87 (31.6)	• 210 (37.4)
• Ache in the extremities (RQ2)	• 96 (33.4)	• 73 (26.5)	• 169 (30.1)
• Burning sensation on the extremities (RQ2)	• 47 (16.4)	• 27 (9.8)	• 74 (13.2)
Bacterial infections	5 (1.7)	3 (1.1)	8 (1.4)
• Impetigo	• 2 (0.7)	• 1 (0.4)	• 3 (0.5)
• Folliculitis	• 1 (0.3)	• 1 (0.4)	• 2 (0.4)
• Furuncle	• 1 (0.3)	• 0	• 1 (0.2)
Fungal infections	112 (39.0)	117 (42.5)	229 (40.7)
• Candidiasis	• 52 (18.1)	• 56 (20.4)	• 108 (19.2)
• Dermatophytosis	• 66 (23.0)	• 71 (25.8)	• 137 (24.4)
• Tinea versicolor	• 7 (2.4)	• 6 (2.2)	• 13 (2.3)

	Metabolic manifestations<br>• Acanthosis nigricans<br>• Waxy skin<br>• Thick skin<br>• Finger pebbling (RQ2)<br>• Skin tags	102 (35.5)<br>• 12 (4.2)<br>• 4 (1.4)<br>• 9 (3.1)<br>• 55 (19.2)<br>• 26 (9.1)	51 (18.5)<br>• 3 (1.1)<br>• 0<br>• 2 (0.7)<br>• 26 (9.5)<br>• 8 (2.9)	153 (27.2)<br>• 15 (2.7)<br>• 4 (0.7)<br>• 11 (2.0)<br>• 81 (14.4)<br>• 34 (6.0)
	Autoimmune diseases<br>• Lichen planus<br>• Vitiligo	10 (3.5)<br>• 2 (0.7)<br>• 8 (2.8)	2 (0.7)<br>• 0<br>• 2 (0.7)	12 (2.1)<br>• 2 (0.4)<br>• 10 (1.8)
	Other/miscellaneous manifestations<br>• Nonspecific itching (RQ2)<br>• Ichthyosis/ xerosis<br>• Eczema/ dermatitis<br>• Seborrhoeic keratoses/ dermatosis papulosa nigra<br>• Idiopathic guttate hypomelanosis	 • 61 (21.3)<br>• 160 (55.7)<br>• 32 (11.1)<br>• 167 (58.2)<br>• 99 (34.5)	 • 61 (22.2)<br>• 132 (48)<br>• 40 (14.5)<br>• 143 (52)<br>• 98 (35.6)	 • 122 (21.7)<br>• 292 (52)<br>• 72 (12.8)<br>• 310 (55.2)<br>• 197 (35.1)
Notes	• Unclear to which extend skin was examined by professional: “All participants evaluated by one of authors for the presence of skin findings.”			

Author(s)	Jelena Barbaric, Mathieu Laversanne and Ariana Znaorb
Titel	Malignant melanoma incidence trends in a Mediterranean population following socioeconomic transition and war: results of age–period–cohort analysis in Croatia, 1989–2013
Year of publication	2017[34]
Review Question (1/2/3)	1
Aim/purpose/objective	• To analyse trends of malignant melanoma incidence in Croatia for men and women of different age groups by birth cohorts and time periods<br>• To interpret the trends in the context of national socioeconomic changes over time and the possible implications for future prevention in South-Eastern European postcommunist countries with high mortality rates
Design	Analyses of registry data
Population (Inclusion/Exclusion Criteria)	Croatian population*
Methods	• Incidence data for the period 1989–2013 from the Croatian National Cancer Registry was analysed<br>• Malignant melanoma of the skin (ICD-9 code 172 and ICD-10 code C43)<br>• UN population estimates were used to calculate age-specific rates<br>• Age Period Cohort model
Sample characteristics	• Age range: 25-79 years
Key findings relevant for review questions	<u>Incidence rates of malignant melanoma in Croatia (age-specific incidence rate per 100,000)</u><br>Men:<br>• 60-64years: Incidence increased from 8 to 25 (peak at 28)/100,000<br>• 65-69 years: Incidence increased from 13 to 40/100,000<br>• 70-74 years: Incidence increased from 18 to 50/100,000<br>• 75-79 years: Incidence increased from 19 to 45/100,000<br><br>Women:<br>• 60-64years: Incidence increased from 7.5 to 18 (peak at 18.5)/100,000<br>• 65-69 years: Incidence increased from 9 to 22/100,000<br>• 70-74 years: Incidence increased from 9 to 28/100,000<br>• 75-79 years: Incidence increased from 10 to 27/100,000
Notes	• Rates are based on figures with semi-log scale<br>• * Croatian national cancer registry: “covers the entire croatian population of app. 4.4 Million”<br>• Rate per 100,000 **population** assumed, not explicitly mentioned

<table>
<tr><td>Author(s)</td><td>George, L. S.; Deshpande, S.; Krishna Kumar, M. K.; Patil, R. S</td></tr>
<tr><td>Titel</td><td>Morbidity pattern and its sociodemographic determinants among elderly population of Raichur district, Karnataka, India</td></tr>
<tr><td>Year of publication</td><td>2017[35]</td></tr>
<tr><td>Review Question (1/2/3)</td><td>1</td></tr>
<tr><td>Aim/purpose/objective</td><td>‘Gather information regarding the morbidity pattern and its sociodemographic determinants among the elderly residing in the rural villages of Raichur, to understand the need for geriatric health-care facilities’</td></tr>
<tr><td>Design</td><td>Cross-sectional</td></tr>
<tr><td>Population (Inclusion/Exclusion Criteria)</td><td>Elderly citizens of six rural villages of Raichur District, India
Inclusion:
>60 years of age</td></tr>
<tr><td>Methods</td><td>Interview with a predesigned and pretested questionnaire
General physical examination
Systemic examination
Cross-check of medical records
Blood sugar estimation
Analysis of data with “Epi Info version – 3.5.3”</td></tr>
<tr><td>Sample characteristics</td><td>Mean age: 65 years (SD 5.44)
65–69 years (37%).
58% female
Random sample of n = 230</td></tr>
<tr><td>Key findings relevant for review questions</td><td><table>
<tr><td>Morbidity pattern</td><td>Male (n = 96)</td><td>Female (n = 134)</td><td>Total (n = 230)</td></tr>
<tr><td>Vitiligo</td><td>1 (25)</td><td>3 (75)</td><td>4 (1.7)</td></tr>
<tr><td>Pediculosis</td><td>0</td><td>1 (100)</td><td>1 (0.4)</td></tr>
<tr><td>Ichthyosis</td><td>1 (16.7)</td><td>5 (83.3)</td><td>6 (2.6)</td></tr>
</table></td></tr>
<tr><td>Notes</td><td>Units not specified in table. Estimates recalculated and confirmed proportions in brakets.</td></tr>
</table>

Author(s)	Elisabeth Hahnel, Andrea Lichterfeld, Ulrike Blume-Peytavi, Jan Kottner
Titel	The epidemiology of skin conditions in the aged: A systematic review
Year of publication	2017[36]
Review Question (1/2/3)	1/2 (RQ2)
Aim/purpose/objective	• Identify, summarize and evaluate the prevalence and incidence of skin conditions in aged individuals being 65 years and older
Design	Secondary data analysis
Population (Inclusion/Exclusion Criteria)	Inclusion:<br>• Secondary data published Jan. 2000 – Sept. 2014<br>• Subjects >65 years (or mean age minus two SD > 60 years)<br>• Reported skin problems and conditions<br>• Primary incidence/prevalence
Methods	• Systematic review
Sample characteristics	• Few point estimates available for non-institutional settings:<br>→ Domesticity (people living at home independently without receiving any care) (14.9%)<br>→ Medical practices (12.2%)<br>→ Home care (5.4%)
Key findings relevant for review questions	Prevalence

	Domesticity	Medical practices	Home care
Pressure ulcers		0.3% - 0.7% (UK)	22.9% (Italy)
Xerosis		55.6% (France)	
Eczema	1.6%; 2.1%; 3.4-5.7%; 30.6% (Sweden)	6.5% - 12.5% (UK)<br>11.9% (Tunisia)	
Itch/pruritus (RQ2)	13.8% (Norway)	6.4% (Tunisia)	
Sensitive skin (RQ2)	16% (USA)		
Actinic keratosis	4.7% - 24.2% (Netherlands)<br>10.4% (Germany)		
Benign skin tumor	18.6% (Sri Lanka)	10.4% (Tunisia)	
Fungal infection	14.3% (Sri Lanka)	45% - 64% (Japan)<br>16.9% (Tunisia)	
Onychomycosis		4% - 29% Brazil<br>41.4% (Tunisia)	
Tinea pedis		12% - 24% (Japan)	6.1% - 8.8% (USA)

<table>
<tr><td></td><td>
<table>
<tr><td>Dermatomycosis</td><td></td><td></td><td>1% - 40% (Iran)</td></tr>
<tr><td>Bacterial skin infection</td><td></td><td>8.7% (Tunisia)</td><td></td></tr>
<tr><td>Viral skin infection</td><td></td><td>6.8% (Tunisia)</td><td></td></tr>
</table>

Incidence
<table>
<tr><td></td><td>Domesticity</td><td>Medical practices</td><td>Home care</td></tr>
<tr><td>Pressure ulcers</td><td></td><td></td><td>6.7% (Italy)</td></tr>
<tr><td>Tinea pedis + Onychomycosis</td><td></td><td>25.7% (Belgium)</td><td></td></tr>
<tr><td>Mycoses</td><td>0.04% (USA)</td><td></td><td></td></tr>
<tr><td>Erysipelas</td><td></td><td>3/1000 - 5/1000 (Belgium)</td><td></td></tr>
</table>
</td></tr>
<tr><td>Notes</td><td>-</td></tr>
</table>

Author(s)	Yves Henchoz, Christophe Büla, Idris Guessous, Nicolas Rodondi, René Goy, Maurice Demont, Brigitte Santos-Eggimann
Titel	Chronic symptoms in a representative sample of community-dwelling older people: a cross-sectional study in Switzerland
Year of publication	2017[37]
Review Question (1/2/3)	2
Aim/purpose/objective	• Determine the prevalence of chronic symptoms in older people, and their associations with disability in basic activities of daily living (BADL), QoL, and their public health impact.
Design	Cross-sectional
Population (Inclusion/Exclusion Criteria)	Older people from the Lausanne cohort 65+ study and older people from the cantons of Vaud and Geneva, Switzerland<br>Inclusion:<br>• Community-dwelling<br>• Able to give informed consent
Methods	• Postal questionnaire<br>• Participants were asked about the occurrence of 14 chronic symptoms, including “skin problems” - for at least 6 months<br>• Assessment of BADL (basic activities of daily living) and QoL (quality of life)<br>• Regression analyses and models adjusted for different models
Sample characteristics	• n = 5300<br>• n = 2781 female<br>• Age: ≥ 68 years
Key findings relevant for review questions	**Weighted prevalence of reported chronic symptoms**<br>(data are weighted prevalence (95% CIs))<br>Skin problems<br>• Total sample (n = 5191): 9.8% (8.6% to 11.0%)<br>• Women (n = 2737): 9.3% (7.6% to 11.0%)<br>• Men (n = 2454): 10.5% (8.9% to 12.2%)<br>• P-value (logistic regression): 0.285<br><br>**Associations between chronic symptoms and unfavourable QoL (ORs)**<br>**Skin problems**<br>• Women (n = 2144): (M1) 1.7*, (M2) 1.5, (M3) 1.3<br>• Men (n=1973): (M1)1.0, (M2) 0.9, (M3) 0.9<br>*p<0.05<br><br>Logistic regression<br>• Model 1: adjusted for age, canton of residence, main city, Swiss citizenship, living arrangement, children, education, financial difficulties and depressive symptoms<br>• Model 2: adjusted for covariates in model 1 and the number of other chronic symptoms<br>• Model 3: adjusted for covariates in model 2 and disability in basic activities of daily living.
Notes	• “The pooled data set comprising 5300 community-dwelling adults aged 68 years and older is representative of older people in two French-speaking Swiss regions.”

Author(s)	Iizaka S, Nagata S, Sanada H.
Titel	Nutritional status and habitual dietary intake are associated with frail skin conditions in community-dwelling older people
Year of publication	2017[38]
Review Question (1/2/3)	1
Aim/purpose/objective	• To investigate the association of nutritional status and habitual dietary intake with skin conditions in community-dwelling older people
Design	Cross-sectional
Population (Inclusion/Exclusion Criteria)	Three community settings in Japan<br>Inclusion:<br>• ≥ 65 years of age<br>• Activities of daily living non-dependent (independent or support-need level according to Japan Long-term Care Insurance System)<br>• Ability to walk to the program with minimal assistance
Methods	• Survey as part of annual health check-up program conductd at<br>→ public apartments<br>→ community center<br>→ temporal housing region<br>→ conducted autum to winter<br>• Self-administered questionnaire<br>• Nutritional assessment<br>• Skin examination<br>• Exclusion from analyses if unkown demographics, not completed skin examination/nutritional assessment<br>• Conducted 2013-2014
Sample characteristics	• n = 118 (83.1% female)<br>• Mean age (SD): 74.1 years (4.8)
Key findings relevant for review questions	Dermatological disease, n (%):<br>• Eczema, 18 (15.3%)<br>• Atopic dermatitis, 1 (0.9)<br>• Zoster, 11 (9.3)<br>• Drug rush, 6 (5.1)
Notes	-

Author(s)	Chante Karimkhani, Danny V Colombara, Aaron M Drucker, Scott A Norton, Roderick Hay, Daniel Engelman, Andrew Steer, Margot Whitfeld,<br>Mohsen Naghavi, Robert P Dellavalle
Title	The global burden of scabies: a cross-sectional analysis from the Global Burden of Disease Study 2015
Year of publication	2017a[39]
Review Question (1/2/3)	2
Aim/purpose/objective	• To estimate the global burden of scabies infection by means of prevalence and disability-adjusted life-years (DALYs).
Design	Secondary data analysis
Population (Inclusion/exclusion Criteria)	As defined in the Global Burden of Disease (GBD) Study 2015<br>• No age limits
Methods	• Collection of scabies epidemiological data from a systematic literature search using the ICD-9 (code 133) and ICD-10 (code B86) codes for scabies between 1980–2014 and hospital insurance data.<br>• Estimation of prevalence.<br>• Estimation of YLDs/DALYs for 195 countries.
Sample characteristics	No data
Key findings relevant for review question(s)	• Global DALYs per 100 000 people (95% CI) by age:<br>→ 60–64 years 38.80 (19.51 – 67.56)<br>→ 65–69 years: 40.02 (20.31 – 71.07)<br>→ 70–74 years: 42.80 (21.40 – 76.67)<br>→ 75–79 years: 52.34 (25.26 – 92.46)<br>→ ≥80 years: 46.38 (24.03 – 78.30)
Notes	• Prevalence not reported by age group

Author(s)	C Karimkhani; RP.Dellavalle; LE Coffeng; C Flohr; RJ Hay; SM Langan; EO Nsoesie; AJ Ferrari; HE Erskine; JI Silverberg; T Vos; M Naghavi
Titel	Global Skin Disease Morbidity and Mortality An Update From the Global Burden of Disease Study 2013
Year of publication	2017b[40]
Review Question (1/2/3)	2
Aim/purpose/objective	• To measure the burden of skin diseases worldwide
Design	Secondary data analysis (incl. registry data)
Population (Inclusion/Exclusion Criteria)	Included: dermatitis (incl. common varieties of eczema: atopic, seborrheic, and contact dermatitis), psoriasis, cellulitis,pyoderma, scabies, fungal skin diseases,viral skin diseases, acnevulgaris, alopecia areata, pruritus, urticaria, decubitus ulcer, malignant skin melanoma, keratinocyte carcinoma(including basal and squamous cell carcinomas).
Methods	• Definition of selected skin conditions (see included) via ICD-10 codes<br>• Additional category “other skin and subcutaneous diseases” for remainder of miscellaneous skin conditions<br>• Thorough investigation of the world literature (via PubMed and Google Scholar) for data on the incidence, prevalence, remission, duration, severity, and mortality risk<br>➔ Languages Spanish and English<br>➔ Time period: 1980 through 2013<br>➔ Extraction from sources including systematic reviews, surveys, population-based disease registries, hospital inpatient data, outpatient data, and cohort studies
Sample characteristics	-
Key findings relevant for review questions	DALY Rate per 100,000 persons ≥ 60 years*:<br><br>• Melanoma: 30 to 50<br>• Keatinocyte carcinoma: 20 to 80 (increases with age)<br>• Dermatitis: 40 to 80 (decreases with age)<br>• Psoriasis: 40 to 70 (decreases with age)<br>• Cellulitis: 10 to 20 (slight increase with age)<br>• Pyoderma: 10 to 30 (slight increase with age)<br>• Scabies: 5 to 10 (decrease with age)<br>• Fungal skin diseases: 30 to 40<br>• Viral skin diseases: 10 to 20<br>• Acne vulgaris: ≤ 5<br>• Alopecia areata: ≤ 10<br>• Pruritus: not visible in graph<br>• Urticaria: 50 to 90 (increases with age)<br>• Decubitus ulcer: 10 to 100 (increases with age)<br>• Other skin and subcutaneous diseases: 60 to 70
Notes	*Interpreted from figure 1

Author(s)	Kim J, Choi Y, Shin J, Jang SY, Cho KH, Nam JY, Park EC
Title	Incidence of Pressure Ulcers During Home and Institutional Care Among Long-Term Care Insurance Beneficiaries with Dementia Using the Korean Elderly Cohort
Year of publication	2017[41]
Review Question (1/2/3)	1
Aim/purpose/objective	• "To assess whether type of long-term care service is a risk factor of the incidence of pressure ulcers among older adults with dementia who are receiving long-term care insurance".
Design	Secondary data analysis
Population (Inclusion/exclusion Criteria)	Long-Term Care Insurance beneficiaries (benefit level 1 or 2) in the Korean Elderly Cohort Dataset collected by the National Health Insurance Corporation<br>Inclusion:<br>• Diagnosis of dementia<br>• Age: 60 years and older<br>• Home care and institutional care beneficiaries
Methods	• Identification of pressure ulcer cases from 2008 to 2013, based on ICD-10 code of pressure ulcer (L89.1e4 and L89.9) and treatment procedure code (M0143) and from surgical records.<br>• Calculation of pressure ulcers frequencies and percentages for several sociodemographic variables (sex, age, region, income, primary caregiver, cohabitant) and comparison by long-term care service type.<br>• Association between type of long-term care services and incidence of pressure ulcer (HRs)<br>• Estimation of survival probability of a pressure ulcer diagnosis by long-term care service type.
Sample characteristics	• n = 4617 (home care)<br>• n = 3,224 (institutional care)
Key findings relevant for review question(s)	Incidence of Pressure Ulcer in home care: 0.5%
Notes	*" This dataset is representative of the country's elderly population"

Author(s)	Lee HJ, Ju YJ, Park EC, Kim J, Lee SG.
Title	Effects of home-visit nursing services on hospitalization in the elderly with pressure ulcers: a longitudinal study
Year of publication	2017[42]
Review Question (1/2/3)	2
Aim/purpose/objective	• To examine the relationship between home-visit nursing services and hospitalization related to pressure ulcer.
Design	Secondary data analysis
Population (Inclusion/exclusion criteria)	Beneficiaries of long-term care insurance (LTCI) in the Korean Elderly Cohort Dataset (South Korea)*<br>Inclusion:<br>• Diagnosis of pressure ulcer<br>• Required nursing care for pressure ulcers and received home-care services at least once under long-term care insurance
Methods	• Data collection for the period 2008 to 2013.<br>• Outcome variable: hospitalizations related to pressure ulcers after the use of home-care services under LTCI
Sample characteristics	• n = 4,807<br>• Mean age: 81.0 years<br>• 39.2% males, 60.8% female<br>• 74.8% no nursing care other than that for their pressure ulcers
Key findings relevant for review question(s)	• 859 (17.9%) patients were admitted to hospitals during the study period.<br>• Home-visit nursing service use was associated significantly with a lower risk of hospitalization related to pressure ulcers (odds ratio [OR] = 0.68, 95% confidence interval [CI] = 0.49–0.93; reference, no use).<br>• Older beneficiaries had a lower risk of hospitalization (OR = 0.99, 95% CI = 0.98–1.00).
Notes	*The dataset includes data from all medical claims under the National Health Insurance (NHI) from 2002 to 2013, comprises a sample of n= 558,147 people aged ≥ 60 years (app. 10.0% of the entire population aged ≥60 years).

Author(s)	N. Pandeya; C. M. Olsen; D. C. Whiteman
Titel	The incidence and multiplicity rates of keratinocyte cancers in Australia
Year of publication	2017[43]
Review Question (1/2/3)	1
Aim/purpose/objective	• To assess the incidence and multiplicity of keratinocyte cancers (basal cell carcinoma [BCC] and squamous cell carcinoma [SCC]) excised in Australia.<br>• To examine variations by age, sex, state, and prior skin cancer history
Design	Secondary data analysis
Population (Inclusion/exclusion Criteria)	Medicare dataset (prepared and posted online by the Australian Department of Health for research purposes in August 2016*)<br>Inclusion:<br>• Citizens and permanent residents in Australia<br>• Age ≥ 20 years in 2011<br>• Submission of at least one Medicare* claim during 2011-2014
Methods	• Individual-level data analysis for a systematic random sample of 10% of all persons registered with Medicare during 1997-2014.<br>• Focus on the eight Medicare Benefits Schedule (MBS) item codes reserved for keratinocyte cancer excisions.<br>• Additional data analysis from the QSkin study to derive age- and sex-specific estimates of the BCC:SCC ratio.<br>• Estimation of the crude person-based incidence, the lesion-based incidence and the type-specific incidence of keratinocyte cancers.
Sample characteristics	• n = 1,704,193 (total)
Key findings relevant for review question(s)	• The age-specific incidence of keratinocyte cancers (person-based) is increasing with age with a peak incidence around the age of 80 years.<br>• The age-specific incidence among 80-84-year-old people increased to “more than 6000 per 100,000 person-years”.<br>• “The incidence of keratinocyte cancer was consistently higher among men than women and this ratio increased with age.”
Notes	*Medicare “is the Australian universal health insurance scheme that subsidizes most medical services (but not those carried out in public hospitals, which are operated by state governments) for citizens and permanent residents”.

Author(s)	K. Thorslund; O. Seifert; K. Nilzen; C. Gronhagen
Titel	Incidence of bullous pemphigoid in Sweden 2005-2012: a nationwide population-based cohort study of 3761 patients
Year of publication	2017[44]
Review Question (1/2/3)	1
Aim/purpose/objective	• To estimate the incidence of bullous pemphigoid in Sweden from 2005 to 2012.
Design	Secondary data analysis
Population (Inclusion/exclusion Criteria)	Population of Sweden<br>Inclusion:<br>• Diagnosis of bullous pemphigoid by a dermatologist in the National Patient Register (NPR) for the first time between 2005 and 2012<br>• ≥ 20 years
Methods	• ICD-10 codes were used to identify the patients in the NPR Database.<br>• Incidence rate was estimated by dividing the average number of cases by the mean of the whole background population in Sweden during 2005-2012<br>• An additional sensitivity analysis was performed.
Sample characteristics	• n = 3761, 54% females<br>• Mean age at diagnosis = 78.9 years (SD 12.1, range 20 – 102)
Key findings relevant for review question(s)	• The incidence rate of BP shows “a marked increase after 80 years of age with an incidence peak between 90 and 99 years of age, 81.9/100,000 (95% CI 75.0–89.2)”.<br>• “…, 81.2% (n = 3053) were 70 years or older and 54.8% (n = 2062) were 80 years or older”<br>• Higher incidence in men from age 70 onwards<br><br>Age-specific incidence rate of BP per 100,000 people from 2005 to 2012 in Sweden:<br><table><tr><td>Age range</td><td>Incidence rate</td></tr><tr><td>60-69</td><td>5</td></tr><tr><td>70-79</td><td>15</td></tr><tr><td>80-89</td><td>50</td></tr><tr><td>90-99</td><td>81.9</td></tr><tr><td>100+</td><td>65</td></tr></table>
Notes	• The Swedish National Patient Register was launched by the National Board of Health and Welfare in 1964 and has virtually complete coverage of all inpatient care—both public and private—since 1987 in Sweden. The reporting is mandatory and from 2001 data from specialized outpatient care is also included, although primary care is not. In most of the patient the diagnosis was set in the outpatient care setting (81.2%, n = 3055)

	• Data on incidence rate per age group were extracted from Fig. 2a

Author(s)	Abuabara, K.; Magyari, A.; Margolis, D. J.; Langan, M.
Titel	The prevalence of atopic eczema across the lifespan: A U.K. population-based cohort study
Year of publication	2018[45]
Review Question (1/2/3)	1
Aim/purpose/objective	• Estimate the age-specific prevalence of active atopic eczema<br>• Examine how it varies by demographic factors
Design	Secondary data analysis
Population (Inclusion/Exclusion Criteria)	Data from The Health Information Network (THIN)<br>Inclusion:<br>• At least one of five diagnostic codes + at least two treatment codes<br>• Acceptable records in THIN
Methods	• Analysis regarding atopic eczema prevalence by age<br>→ Time period between 1994 and 2013 analysed<br>→ Active disease: at least one eczema-associated code (diagnostic or treatment) in any given year based on chronological age
Sample characteristics	• n = 848 435 (all ages)
Key findings relevant for review question(s)	• 8% with active disease at ages ≥ 75 years (prevalence)<br>• “The prevalence of active eczema by age followed a U-shaped curve.”<br>• “Prevalence of active physician-diagnosed eczema is highest during infancy and older age.”<br>• More common in males ≥ 75 years
Notes	• Conference Abstract<br>• THIN: “A primary-care electronic medical record database, which is representative of the general population in the U.K.

Author(s)	Joanne F. Aitken, Danny R. Youlden, Peter D. Baade, H. Peter Soyer, Adéle C. Green, B. Mark Smithers
Titel	Generational shift in melanoma incidence and mortality in Queensland, Australia, 1995–2014
Year of publication	2018[46]
Review Question (1/2/3)	1/2/3
Aim/purpose/objective	• Examine trends in melanoma incidence, mortality<br>• Assess whether the earlier reported patterns of stabilizing mortality and, in younger people, stabilizing incidence, have been sustained
Design	Analyses of registry data
Population (Inclusion/Exclusion Criteria)	Cases of Queensland Cancer Registry (Australia)
Methods	Analysis of extracted registry data<br>• Patients diagnosed with a first primary invasive or in situ melanoma (ICD-O-3 code "C44,"morphology code 8720–8790) including Lentigo maligna<br>• Time period 1995 – 2014<br>• Only first diagnosis per patient included in case of multiple primary invasive melanomas<br>• Incidence and mortality trends (annual percentage change)<br>• Age period cohort model (best fit)<br><br>Mortality via record linkage:<br>• Queensland Cancer Registry<br>• Queensland Register of Births, Deaths and Marriages<br>• National Death Index
Sample characteristics	• n= 97,114 (all ages)<br>• Median age at diagnosis: 60 years<br>• Total n = 39,271 (60+)<br><br>Cases in persons 60+ years:<br>• n = 17,186 invasive (male)<br>• n = 9,504 invasive (female)<br>• n = 7,776 in situ (male)<br>• n = 4,805 in situ (female)
Key findings relevant for review questions	1.<br><u>Incidence rates for age group 60+ in 1995-2014</u><br>M: 200 to 500/100,000<br>F: 100 to 200/100,000<br><br><u>Annual percentage change (APC)</u><br>• In situ 7.0 to 7.1<br>• Invasive 1.8 to 2.1<br><br><u>Incidence rates for age group 60+ in 1995-2014</u><br><u>Invasive melanoma</u><br>• Incidence of thin and thick "melanoma increased uniformly over whole study period"

<table>
<tr><td></td><td>

- Incidence rates for all “age groups 60 increased over 20 year study period” but “deceleration in almost all successive birth cohorts from 1930 onwards”

In situ melanoma

- Incidence increased over last 10 years uniformly
- Incidence rates increased but “began to slow from about the 1945 birth cohort”

2.
<u>Age-specific mortality rates of invasive melanomas in 1995-2014</u>

- Mortality rate increases with age
- Overall age-standardised mortality rate is stable but different when analysed by broad age groups
  → Only mortality rate in males 60+ years increased significantly (by 1.8% per year in study period)
- Total deaths due to melanoma between 1995 and 2014 in Queensland, n = 5061
- Median age at death was 70 for males and 68 for females
- 2/3 of deaths were male
- Mortality rates (2014)
  → Males ≥60 years: App. 40/100,000
  → Females ≥60 years: App. 15/100,00

3.

- Age-standardised incidence of melanoma only continues to increase in ≥60 year old persons
- (<60 years decline or stabilisation)
- Melanoma mortality only increased in males ≥60 years
- “The results support our assertion that public education campaigns and changing attitude to sun exposure have played a key role in the observed reduction of melanoma incidence among more recent birth cohorts”
- “These trends are likely explained, at least in part, by improved understanding and awareness, both among clinicians and the public, of the critical importance of early detection and treatment of this disease”

</td></tr>
<tr><td>Notes</td><td>

- Rates are interpreted from figure 2 with a log scale.

</td></tr>
</table>

Author(s)	Fiona Cowdell, Judith Dyson, Judith Long, Una Macleod
Titel	Self-reported skin concerns: An epidemiological study of community-dwelling older people
Year of publication	2018[47]
Review Question (1/2/3)	2
Aim/purpose/objective	• Identifying the frequency and impact of self-reported skin concerns among a cohort of community-dwelling older people
Design	Cross-sectional
Population (Inclusion/Exclusion Criteria)	Patients registered with 3 GP's in UK<br>Inclusion:<br>• 75 years and older<br>• Community-dwelling<br>• Able to give informed consent
Methods	• Self-reported questionnaire study<br>• (Invitation to a free health and care assessment was sent via letter to eligible patients registered with 3 GP-practices in NE-England<br>• Interview with EASYCare Standard 2010 questionaire)<br>• Questionnaire via the telephone (majority) or face-to-face meetings
Sample characteristics	• n = 1116 participants 55.5 % female<br>• 75–99 years (mean age 81.14 [SD 4.7])
Key findings relevant for review questions	Do you have any concerns about your skin?"<br>➔ Yes: n = 183 (16.5 %)<br>(see table below)<br>Most bothersome conditions:<br>→ Itch (a little 46.4%, a lot 27.7% and very much 5.4% [mean rating = 2.18])<br>→ Dry skin (a little 58.8%, a lot 11.5% and very much 4.1% [mean rating = 1.94])<br>→ Inflammation (a little 44.8%, a lot 27.7% and very much 1.5% [mean rating = 1.94])
Notes	-

Concern	Percentage of all 1116 respondents (number)
Dry skin	12.1 (146)
Itch	9.3 (104)
Inflammation	4.9 (55)
Leg or pressure ulcer	1 (11)
Precancerous or cancerous lesion	1.5 (17)
Skin infection	0.8 (9)
Aged appearance	10.2 (114)
Nail infection	2.2 (25)
Abnormal hair loss	1 (11)

Author(s)	K. P. Drewitz; K. Stark; M. E. Zimmermann; I. Heid; C. J. Apfelbacher
Titel	Prevalence and determinants of Psoriasis in a cross- sectional study of the elderly—results from the German AugUR study
Year of publication	2018[48]
Review Question (1/2/3)	1
Aim/purpose/objective	Investigate prevalence of and factors associated with psoriasis in an elderly population in Germany
Design	Cross-sectional<br>(Data from cohort AugUR study)
Population (Inclusion/Exclusion Criteria)	Data of longitudinal study (AugUR study)<br>→ Mobile elderly population in Regensburg, Germany and surrounding areas
Methods	• No data
Sample characteristics	• n = 1133<br>• Median age: 76.7
Key findings relevant for review questions	• 5.48% study participants reported to ever have been diagnosed with psoriasis<br>• Prevalence was highest in the group aged 80- 84 years (7.8%)<br>• Lowest prevalence in those aged 90- 95 (0%)
Notes	• Conference abstract

Author(s)	Dziunycz PJ, Schuller E, Hofbauer GFL.
Title	Actinic Keratosis in Patients Attending General Practitioners in Switzerland.
Year of publication	2018[49]
Review Question (1/2/3)	1
Aim/purpose/objective	• "To assess the prevalence of actinic keratosis (AK) in the outpatient Swiss population in general practice"
Design	Cross-sectional
Population (Inclusion/exclusion Criteria)	Patients of 59 General Practitioners in Switzerland<br>Inclusion:<br>• Age: 30 years or older<br>• Written informed consent
Methods	• Participating GPs captured AK diagnosis, stage and localization in consecutive patients via clinical examination.<br>• Questionnaire for patients regarding sun exposure.
Sample characteristics	• n = 2,844<br>• 55.7% female<br>• Median age: 60 years
Key findings relevant for review question(s)	• Prevalence of AK per age group:<br>→ 60 to <70 years: app. 25%<br>→ 70 to <80 years: app. 45%<br>→ 80 to <90 years: app. 55%<br>→ 90 to < 100 years: 69.4%<br>• "The prevalence of AK increased steadily with patient age".<br>• "Age was a strong risk factor for the diagnosis of AK".
Notes	• For age groups 60 to 90 years prevalence data were extracted from figure 2.

Actinic keratosis (AK)

Author(s)	Li Hu, Shaofei Jin, Ling Chen, Yongli Wang
Titel	Trends in the incidence and mortality of cutaneous melanoma in Hong Kong between 1983 and 2015
Year of publication	2018[50]
Review Question (1/2/3)	1/2
Aim/purpose/objective	• Investigate the trends in incidence and mortality rates of cutaneous melanoma (CM) in Hong Kong between 1983 and 2015
Design	Analyses of registry data
Population (Inclusion/Exclusion Criteria)	Data from Hong Kong Cancer Registry, Hospital Authority<br>Inclusion:<br>• ICD-10 code C43
Methods	• Time period analysed 1983 to 2015<br>• Joint-regression method<br>• Age-period-cohort model (best fit)
Sample characteristics	• Cases age group 65-85+:<br>→ n = 807 (incidence)<br>→ n = 471 (mortality)
Key findings relevant for review questions	**1.** (see table below)<br>Age-standardised incidence rate (65+ years): 3.3/100,000<br><br>**2.**<br>• "The age-standardised rate (ASR) of incidence and mortality increased with age"<br>➔ Overall ASR mortality (65+ years): 1.80<br><br>• "…a full age-period-cohort model best explained the relationships between the changes in the ASR and the explanatory variables (age, period, and cohort) for the total, male, and female"
Notes	• "Hospital Authority manages the Hong Kong's public hospital services, and the Hong Kong Cancer Registry is a population-based cancer registry and a member of the International Association of Cancer Registries."

Key findings, 1.:

	n (1983-2015)	AAPC: Average annual percentage changes (1983-2015)
Incidence overall (65-85+ years)	807	-1.4 (-2.5, -0.4)
Mortality overall (65-85+ years)	471	1.9 (0.4, 3.4)
Incidence males (65-85+ years)	395	-0.5 (-2.0, 1.0)
Mortality males (65-85+ years)	246	1.6 (-0.2, 3.3)
Incidence females (65-85+ years)	412	-2.6 (-5.2, 0.1)
Mortality females (65-85+ years)	225	2.4 (0.5, 4.3)

Cutaneous melanoma (CM)

Author(s)	A. Lichterfeld-Kottner, N. Lahmann, U. Blume-Peytavi, U. Mueller-Werdan, J. Kottner
Titel	Dry skin in home care: A representative prevalence study
Year of publication	2018[51]
Review Question (1/2/3)	1/2 (RQ2)
Aim/purpose/objective	• To measure the prevalence and severity of dry skin in the home care setting in Germany and to identify demographic and health characteristics related to skin dryness
Design	Cross-sectional study
Population (Inclusion/Exclusion Criteria)	Clients receiving nursing care at home by nurses funded by the statutory nursing insurance in Germany<br>Inclusion:<br>• ≥18 years
Methods	• Stratified random selection of home care services from list with all home care services in Germany, then random sample from clients registered with these home care services<br>• Standardized paper data collection form, filled out by trained nurses from local home care services (Assessment of skin condition, skin care interventions provided)<br>➔ Sent to research center when completed<br>➔ Time period data collection: July 2015
Sample characteristics	• n = 102 home care services<br>• n = 923 home care receivers (70% female)<br>• Median age: 83 years (IQR 77–88); (min-max range 21–104 years)<br>• Mean age: 80.6 (SD 11.1)
Key findings relevant for review questions	• Prevalence of (see table below)

IAD	14.7% (95% CI 12.6% to 17.2%)	Mostly moderate IAD (10.2%)
PUs (category I-IV)	3.6% (95% CI 2.6% to5.0%).	Trochanter/sacrum (2.1)<br>Heel/ankle (1%)
Ulcus cruris	2.4% (95% CI 1.6% to 3.6%)	-
Diabetic foot syndrom	1.0% (95% CI 0.5 to 1.8)%)	-
Chronic wound due to pAVK	0.3% (95% CI 0.1 to 1.0)	
Skin dryness	(51.7%; 95% CI 48.5 % to 54.9%)	Feet and legs (45.8%)/<br>Hands and arms (40%)<br>Mild (20.6% to 23.3%)<br>Severe (2.0 to 6.5%)<br>Skin cracks (1.3 to 2.5%)
Pruritus (RQ2)	19.5% (95% CI 17.1 to 22.2)	

	• “Skin care interventions (washing, bathing, showering and/or creaming) were applied in approximately 80% of the home care receivers”
Notes	-

Author(s)	M. G. H. Sanders; L. M. Pardo; O. H. Franco; R. S. Ginger; T. Nijsten
Title	Prevalence and determinants of seborrheic dermatitis in a middle-aged and elderly population: the Rotterdam Study
Year of publication	2018[52]
Review Question (1/2/3)	1
Aim/purpose/objective	• To estimate the prevalence of seborrheic dermatitis<br>• To investigate the reported determinants of seborrheic dermatitis
Design	Cross-sectional<br>(Data from Rotterdam cohort study)
Population (Inclusion/exclusion Criteria)	Residents of Ommoord district of Rotterdam, Netherlands<br>Inclusion:<br>• 45 years of age or older
Methods	• Full-body skin examination (FBSE) conducted by dermatology-trained physicians<br>• Diagnosis seborrheic dermatitis: greasy scaling + erythema + characteristic distribution in areas rich in sebaceous glands<br>• Literature search for potential determinants was carried out<br>• Home interview conducted before (95% within 6 months) FBSE<br>• Assessment of Depression and anxiety disorders
Sample characteristics	• n = 5498<br>• Females 57%<br>• Median age at FBSE: 67.9 years (IQR: 61.9–76.4)
Key findings relevant for review question(s)	Point prevalence of seborrheic dermatitis: 14.3%, n = 788<br><br>Age (years), median (IQR):<br>• Seborrhoeic dermatitis: 70.1 (63.1 to 77.4)<br>• No seborrhoeic dermatitis: 67.7 (61.7 to 76.3)<br>• p-value 0.027
Notes	-

Full-body skin examination (FBSE)

Author(s)	R. B. Steglich; K. M. d. P. A. Coelho; S. Cardoso; M. H. d. C. N. Gaertner; T. F. Cestari; S. C. Franco
Titel	Epidemiological and histopathological aspects of primary cutaneous melanoma in residents of Joinville, 2003-2014
Year of publication	2018[53]
Review Question (1/2/3)	1
Aim/purpose/objective	• To analyze epidemiological and pathological characteristics of primary cutaneous melanoma (CM) in Joinville, southern Brazil.
Design	Secondary data analysis
Population (Inclusion/exclusion Criteria)	Residents of Joinville, Brazil with a diagnosis of primary cutaneous melanoma and registered in one of the included databases<br>• Exclusion criteria:<br>– reports of cases from other cities<br>– cases of neoplasms affecting other organs and tissues<br>– revision reports of surgical pathology slides<br>– reports with inconclusive diagnosis without immunohistochemical evidence<br>– reports of residual neoplasia or enlargement of the surgical margin.
Methods	• Time period analysed: January 2003 and December 2014<br>• Collection of all reports of cases of primary cutaneous melanoma in the analysed time period from the only three laboratories of pathological anatomy of the city.<br>→ Use of “melanoma” and “lentigo maligna” in the diagnostic field and “skin and/or cutaneous” for the affected organ in 2/3 laboratory databases<br>→ 1/3 laboratories: diagnostic registry books (2003-2007) and database<br>→ Missing data (e.g. residency) obtained from 3 further databases (Hospitals, outpatient clinics, private doctors offices)<br>• Calculation of the gross CM incidence rate for each year using the Joinville population of the same year (estimated by the Brazilian Institute of Geography and Statistics) per 100,000 population.<br>• Calculation of distribution of CM cases according to anatomical site and age group.<br>• Calculation of gender-adjusted rates, and rates adapted to the Brazilian and world standard population for each year.
Sample characteristics	• n = 819 patients, 56.3% females<br>• n = 893 cases<br>• Mean age: 54.6 years (SD 16.5)
Key findings relevant for review question(s)	• CM in people 60 years and older, n (%):<br>• Total n = 347 cases<br>→ Superficial spreading melanoma: 149<br>→ Nodular melanoma: 95<br>→ Lentigo maligna melanoma: 54<br>• 38.9% of cases were 60 years or older
Notes	• Incidence rates are not reported per age group.

Cutaneous melanoma (CM)

Author(s)	M. Augustin, N. Kirsten, A. Korber, D. Wilsmann-Theis, G. Itschert, P. Staubach-Renz, J-T. Maul,N. Zander
Titel	Prevalence, predictors and comorbidity of dry skin in the general population
Year of publication	2019a[54]
Review Question (1/2/3)	1
Aim/purpose/objective	• Gain robust data for the working-age population on the prevalence and comorbidity of dry skin across Germany<br>• Determine the prevalence of dry skin in the German adult population
Design	Cross-sectional
Population (Inclusion/Exclusion Criteria)	Employees in 343 German companies
Methods	• Whole-body examinations conducted by experienced dermatologists<br>• Examinatons conducted voluntarily during working hours<br>• Overall dry skin score was applied<br>• Conducted between 2001 and 2005
Sample characteristics	• n = 48,630 (total sample)<br>• n = 4,525 employees 60-70 years of age
Key findings relevant for review questions	• Prevalence of dry skin in age group 60-70 years<br>➔ 36.4%
Notes	-

Author(s)	M Augustin - N Kirsten - A Körber - D Wilsmann-theis - G Itschert - P Staubachrenz - J Maul - N Zander
Titel	Epidemiology of dry skin in the general population
Year of publication	2019b[55]
Review Question (1/2/3)	1
Aim/purpose/objective	• Determine the prevalence of dry skin in the German adult population
Design	Cross-sectional
Population (Inclusion/Exclusion Criteria)	Employees of 343 German companies
Methods	• Full-body examinations by experienced dermatologists
Sample characteristics	• n = 48,630 (total sample)
Key findings relevant for review questions	• Older age was associated with xerosis → 38.4% in the group 60-70 years of age.
Notes	• Conference abstract<br>• Full article: Augustin_2019a

Author(s)	K. P. Drewitz; K. Stark; M. E. Zimmermann; I. M. Heid; C. J. Apfelbacher
Titel	Frequency and comorbidities of eczema in an elderly population in Germany: results from augur
Year of publication	2019[56]
Review Question (1/2/3)	1
Aim/purpose/objective	• Investigate the prevalence of atopic eczema (AE) and hand eczema (HE) and associated comorbidities in an elderly mobile population in Germany
Design	Cross-sectional<br>(Data of the AugUR cohort study)
Population (Inclusion/Exclusion Criteria)	Elderly population in Regensburg, Germany and surrounding areas
Methods	No data
Sample characteristics	• n = 1133<br>• Median age: 76.7 years
Key findings relevant for review questions	• 3.3% (95%-confidence interval (CI) 2.31-4.47) reported a previous diagnosis of AE (59% female)<br>• Frequency of AE was highest in the age group 85-89 years (4.4%, 95%-CI 1.22-10.99)<br>• Frequency of AE was lowest in age group 75-79 years (2.4%, 95%-CI 1.72-4.43)<br>• 2.7% (95%-CI: 1.79-3.76) of study participants reported a previous diagnosis of HE<br>• Frequency of HE was highest in age group 90-95 years (3.9%, 95%-CI 0.10-19.64)<br>• Lowest frequency of HE in age group 80-84 years (0.5%, 95%-CI 0.01-2.67)
Notes	Conference abstract

Atopic eczema (AE)
Hand eczema (HE)

Author(s)	S. Mekic; L. C. Jacobs; D. A. Gunn; A. E. Mayes; M. A. Ikram; L. M. Pardo; T. Nijsten
Title	Prevalence and determinants for xerosis cutis in the middle-aged and elderly population: A cross-sectional study
Year of publication	2019[57]
Review Question (1/2/3)	1
Aim/purpose/objective	• To identify the prevalence and determinants for generalized dry skin and localized dry skin in the middle-aged and elderly population.
Design	Cross-sectional
Population (Inclusion/exclusion Criteria)	The Rotterdam Study (RS) cohort*
Methods	• Full-body skin examination by a dermatology-trained physician (2010 to 2016)<br>• Evaluation of dry skin:<br>➔ Scaly or rough skin with/out erythema (not fitting other known skin disease)<br>➔ Dry skin score: Absent, localized, or generalized<br>➔ Assessment of associated diseases<br>• Individual data (age, sex, educational level, lifestyle and environmental factors, medication) collection from the RS database and direct interviews.
Sample characteristics	• n = 5547<br>• Age range: 51-101 years; mean age: 70 years<br>• 57% female
Key findings relevant for review question(s)	• 60% of the participants (95% confidence interval [CI], 58%-61%) had dry skin.<br>• Of the individuals with dry skin, 1 in 5 were severely affected and had generalized dry skin, whereas the rest had dry skin only on the extensor side of the extremities (localized dry skin).
Notes	*The cohort study started in 1990 and initially comprised 7,983 persons aged ≥ 55 years, living in the well-defined Ommoord district in the city of Rotterdam in the Netherlands. The cohort has been expanding since then. In 2008, 14,926 subjects aged ≥45 years comprised the Rotterdam Study cohort. Since 2016, the cohort is being expanded by persons aged 40 years and over. Reference: Ikram, M.A., Brusselle, G., Ghanbari, M. et al. Objectives, design and main findings until 2020 from the Rotterdam Study. Eur J Epidemiol 35, 483–517 (2020).

Author(s)	J. L. Meyers; S. D. Candrilli; D. A. Rausch; S. Yan; B. J. Patterson; M. J. Levin
Title	Costs of herpes zoster complications in older adults: A cohort study of US claims database
Year of publication	2019[58]
Review Question (1/2/3)	2
Aim/purpose/objective	• To assess the incidence of Herpes Zoster (HZ)-related complications other than postherpetic neuralgia (PHN);<br>• To record the incremental healthcare resource utilization due to these complications;<br>• To determine the associated costs compared to uncomplicated HZ in a large cohort of immunocompetent individuals aged ≥50 years.
Design	Secondary data analysis
Population (Inclusion/exclusion Criteria)	Commercially insured persons (US) enrolled in managed healthcare plans with complete medical and pharmacy claims in the included databases:<br>→ Truven Health Analytics MarketScan Commercial Claims and Encounters<br>→ Medicare Supplemental and Coordination of Benefits administrative claims data<br>Inclusion:<br>• Age ≥50 years<br>• Presence of a primary or secondary diagnosis of HZ<br>• Without PHN<br>• Continuous health plan enrolment from min. six months before until 12 months after the HZ index date.<br>• No vaccination against HZ<br>• Immunocompetent
Methods	• Case identification based on the ICD-9-CM diagnosis code 053.xx<br>• Identification of HZ-related complications via ICD-9-CM codes on or after index date (depending on complication)<br>• Time period analysed: 2008 to 2013<br>• Follow-up for 1 year after HZ onset.<br>• Calculation of all-cause healthcare resource utilization and direct healthcare costs from six months before until 12 months after HZ onset.<br>• Comparison of the mean costs for HZ patients with complications to the mean costs for patients with uncomplicated HZ.<br>• Estimation of the mean incremental costs adjusted for demographics, comorbidities, type of complication (3 categories: cutaneous, neurologic, ophthalmic) and time period with multivariable regression models.
Sample characteristics	• n = 236,180 cases (without PHN)<br>– Patients with non-PHN complications: N= 22,948, mean age (SD): 65.4 (11.6)<br>– Patients with uncomplicated HZ: N= 213,232, mean age (SD): 63,6 (10,7)

Key findings relevant for review question(s)	• "The proportion of patients with any non-PHN complication increased with age": 8.9% in 60-64 year old 9.7% in patients aged 65-69, 11.1% in patients aged 70-79 and 13.3% in patients aged ≥80 years.<br>• "The proportion of patients with neurologic complications was twice as high in patients aged ≥80 years as in patients aged 50–59, whereas for both cutaneous and ophthalmic complications the relative increase between these age groups was approximately 50%".<br>• "The mean incremental unadjusted costs increased with advancing age up to the group aged 70–79 years and then decreased for HZ patients aged ≥80 years".<br>• The mean incremental adjusted costs for patients with any HZ complication increased monotonically with age and this age-related pattern was observed for each of the complication categories separately".
Notes	-

Herpes Zoster (HZ)
Postherpetic neuralgia (PHN)

Author(s)	Sari SP, Everink IH, Sari EA, Afriandi I, Amir Y, Lohrmann C, Halfens RJ, Schols JM.
Titel	The prevalence of pressure ulcers in community-dwelling older adults: A study in an Indonesian city
Year of publication	2019[59]
Review Question (1/2/3)	1
Aim/purpose/objective	• "To investigate the prevalence and the characteristics of pressure ulcers (PU) in community-dwelling older adults in Indonesia"
Design	Cross-sectional
Population (Inclusion/exclusion Criteria)	Residents in 6 "Kelurahan"* of Bandung, the capital city of the West Java province (n = 6,745)<br>Inclusion:<br>• Age ≥ 60 years
Methods	• Random selection of n = 325 older adults.<br>• Use of the Indonesian version of the International Prevalence Measurement of Care Quality (LPZ-International) questionnaire.<br>• Assessment of PUs by skin inspection according to NPUAP-EPUAP-PPPIA Guidelines (6 categories).<br>• Assessment of risk of PUs for each participant using the Braden scale.<br>• Assessment of care dependency scale.
Sample characteristics	• n = 325<br>• Mean age: 72.1<br>• 67.7% female
Key findings relevant for review question(s)	• Overall prevalence of PU (all categories): 10.8% (95% confidence interval [CI], 5.8-15.8).<br>• Prevalence of PU excluding category 1 (non-blanchable erythema): 5.2% (95% CI 0.2-10.2).
Notes	* "There are 30 districts in Bandung, making up the six regions of the city. Every district has smaller municipalities, called Kelurahan."

Author(s)	E. S. d. Silva; S. C. Dumith
Titel	Non-use of sunscreen among adults and the elderly in southern Brazil
Year of publication	2019[60]
Review Question (1/2/3)	3
Aim/purpose/objective	• To investigate the prevalence of non-use of sunscreen and its associated factors in the adult and elderly population of a municipality in southern Brazil.
Design	Cross-sectional
Population (Inclusion/exclusion Criteria)	Individuals living in the urban area of Rio Grande, Brazil<br>Inclusion:<br>• ≥18 years<br>Exclusion:<br>• institutionalization in nursing homes, hospitals, or prisons<br>• having a physical and/or mental disability that prevented answering the questionnaire.
Methods	• Multiple-stage sampling strategy to obtain a representative sample of the urban population of the city, based on data of the 2010 Population Census<br>• Data collection between April and July of 2016<br>• Use of a pre-coded questionnaire with closed questions. Outcome assessment through the question ‘‘Do you usually use sunscreen?’’<br>• Calculation of crude and adjusted prevalence ratio (PR) (Poisson regression with robust adjustment for variance)
Sample characteristics	• n (total) = 1300<br>• ≥60 years old: n = 315
Key findings relevant for review question(s)	• Of the individuals ≥ 60 years old:<br>– 52.7% did not use sunscreen.<br>– Crude prevalence rate (95% CI): 1.68 (1.42-1.99)<br>– Adjusted prevalence rate (95% CI): 1.96(1.67-2.31)<br>• “The greater the age, the higher the probability of non-use of sunscreen (p<0.001)”.
Notes	-

Author(s)	L. Tizek, M.C. Schielein, F. Seifert, T. Biedermann, A. Boehner, A. Zink
Titel	Skin diseases are more common than we think: screening results of anm unreferred population at the Munich Oktoberfest
Year of publication	2019[61]
Review Question (1/2/3)	1
Aim/purpose/objective	• Point prevalence of skin diseases in unreferred population outside of typical medical setting.
Design	Cross-sectional
Population (Inclusion/Exclusion Criteria)	Visitors of ‘Bavarian Central Agricultural Festival’ (‘Bayerisches Zentrales Landwirtschaftsfest (ZFL)’))<br>• ≥18 years
Methods	• Self-administered paper-based questionaire (23 items)<br>• Skin examination (dermatologist using dermatoscope)<br>• Descriptive data<br>• Analyses of associations between skin diseases and potential risk factors
Sample characteristics	• Participants total: n = 2701<br>• Participants 60 +: n = 923<br>• Participants 60-69: n = 688<br>• Participants 70+: n = 235
Key findings relevant for review questions	Prevalence among participants in the skin cancer screening, n (%)<br>**Any skin diseases**<br>• 60-69 years (n = 661): 492 (74.4)<br>• 70 + years (n = 225):187 (83.1)<br>**AK** (= actinic keratosis)<br>• 60-69 years (n = 661):276 (41.8)<br>• 70 + years (n = 225):147 (65.3)<br>**KC** (= keratinocyte carcinomas including basal cell carcinoma and squamous cell carcinoma)<br>• 60-69 years (n = 661): 36 (5.4)<br>• 70 + years (n = 225): 15 (6.7)<br>**Rosacea**<br>• 60-69 years (n = 661): 223 (33.7)<br>• 70 + years (n = 225): 74 (32.9)<br>**Eczema**<br>• 60-69 years (n = 661): 70 (10.6)<br>• 70 + years (n = 225):18 (8.0)<br>**Psoriasis**<br>• 60-69 years (n = 661): 8 (1.2)<br>• 70 + years (n = 225): 2 (0.9)<br>**Acne**<br>• 60-69 years (n = 661): 2 (0.3)<br>• 70 + years (n = 225): 0
Notes	-

<table>
<tr><td>Author(s)</td><td>Z.C. Venables, T. Nijsten, K.F. Wong, P. Autier, J. Broggio, A. Deas, C.A. Harwood, L.M. Hollestein, S.M. Langan, E. Morgan, C.M. Proby, J. Rashbass and I.M. Leigh</td></tr>
<tr><td>Titel</td><td>Epidemiology of basal and cutaneous squamous cell carcinoma in the U.K. 2013–15: a cohort study</td></tr>
<tr><td>Year of publication</td><td>2019a[62]</td></tr>
<tr><td>Review Question (1/2/3)</td><td>1</td></tr>
<tr><td>Aim/purpose/objective</td><td>• To estimate the incidence of basal cell carcinoma (BCC) and cutaneous squamous cell carcinoma (cSCC) in the U.K.</td></tr>
<tr><td>Design</td><td>Analyses of registry data</td></tr>
<tr><td>Population (Inclusion/exclusion Criteria)</td><td>• Data from the national cancer registration and analyses service (NCRAS), England, combined with Patient Administration System and Cancer Outcomes and Services dataset (COS)
• Data from Information Services Devision Scotland, Northern Ireland Cancer Registry (and Welsh Cancer Intelligence and Surveillance Unit)
Inclusion:
• Diagnosis of BCC or cSCC between 2013 and 2015 with ICD-10 site codes and/or ICD-02 morphology and beviour codes</td></tr>
<tr><td>Methods</td><td>• Welsh data was excluded except for overall tumour count
• European age-standardized incidence rates (EASRs)
• Date of diagnosis of first BCC and cSCC per patient per annum (PPPA): Date of receipt of pathology sample</td></tr>
<tr><td>Sample characteristics</td><td>
<table>
<tr><td></td><td colspan="2"><u>First BCC:</u></td><td colspan="2"><u>First SCC</u></td></tr>
<tr><td></td><td>Male</td><td>Female</td><td>Male</td><td>Female</td></tr>
<tr><td>60-69 years; n (%)</td><td>35,487 (24.5)</td><td>27,893 (22.5)</td><td>7,352 (15.2)</td><td>3,930 (13.7)</td></tr>
<tr><td>70-79 years</td><td>46,551 (32.2)</td><td>33,990 (27.4)</td><td>16,158 (33.5)</td><td>7,735 (26.9)</td></tr>
<tr><td>80-89 years</td><td>30,631 (21.2)</td><td>25,913 (20.9)</td><td>17,822 (36.9)</td><td>10,385 (36.2)</td></tr>
<tr><td>90+</td><td>4,973 (3.4)</td><td>6,554 (5.3)</td><td>3,997 (8.3)</td><td>4,569 (15.9)</td></tr>
<tr><td>Median age at time of incident tumour</td><td colspan="2">app. 71 years (IQR 62–80)</td><td colspan="2">app. 79 years (IQR 71–85)</td></tr>
</table>
</td></tr>
<tr><td>Key findings relevant for review question(s)</td><td>• “The age-specific rates for both BCC and cSCC clearly increase with age and are much higher and steeper in males than females”
• The age-specific incidence of BCC in the U.K. (excluding Wales) in 2015 was
→ App. 300 per 100,000 person years in males, 60-64 years
→ App. 200 per 100,000 person years in females, 60-64 years
→ App. 1250 per 100,000 person years in males, 90+ years, (peak at 1300/100.000 person years in 85-89 year olds)
→ App. 650 per 100,000 person years in females, 90+ years, (peak at 700/100.000 person years in 85-89 year olds)</td></tr>
</table>

	• The age-specific incidence of cSCC in the U.K. (excluding Wales) in 2015 was<br>→ App. 50 per 100,000 person years in males, 60-64 years<br>→ App. 20 per 100,000 person years in females, 60-64 years<br>→ App. 1000 per 100,000 person years in males, 90+ years<br>→ App. 500 per 100,000 person years in females, 90+ years
Notes	• Mandatory for all National Health Service pathology laboratories to report to NCRAS, for private ones it is recommended<br>• Age specific rates for both BCC and SCC were extracted from Fig. 4.

Basal cell carcinoma (BCC)
cutaneous squamous cell carcinoma (cSCC)

Author(s)	ZC Venables; P Autier; T Nijsten; KF Wong; SM Langan; B Rous; J Broggio; C Harwood; K Henson; CM Proby; J Rashbass; IM Leigh
Titel	Nationwide Incidence of Metastatic Cutaneous Squamous Cell Carcinoma in England
Year of publication	2019b[63]
Review Question (1/2/3)	1
Aim/purpose/objective	• To assess the national incidence of cSCC and metastatic cSCC (mcSCC) in England from 2013 through 2015
Design	Analysis of registry data
Population (Inclusion/Exclusion Criteria)	Data of National Cancer Registration and Analysis Service (NCRAS)*<br>Inclusion:<br>• Registered cSCC (and mcSCC) cases in England from 2013 to 2015<br>Exclusion:<br>→ In situ cSCC, Bowen disease, mucosal cSCC, and genital cSCC, metastatic cSCCs of unknown origin or potentially from other primary sources, metastasis originally diagnosed before 2013
Methods	• Cutaneous SCCs identified using topographical code C44 from ICD-10 and morphology codes 8050 to 8052, 8070 to 8078, and 8082 to 8084; and behavior code 3 (malignant) from ICD-O-2<br>• Nodal or distant mcSCC were identified from the persons identified in the database with a diagnosis of cSCC<br>→ Via keywords, staging data, hospital episode statistics operation code data, mortality data from Office for National Statistics (ONS)<br>→ Review pathology reports with specific codes<br>• Age-standardized incidence rates using the 2013 European Standard Population, reported per 100,000 person-years (PY)<br>• Time perid analysed: 2013 through 2015
Sample characteristics	• n = 93 890 cases of cSCC (total)<br>• n = 71,948 cases of cSCC (60+)<br>• Median age: 80 years [IQR, 72-86]
Key findings relevant for review questions	Primary cSCCs occurred predominantly in patients 70 years and older → 78.8% ≥70 years (6. 1% ≤ 59 years)<br>Age-standardised incidence rate:<br>→ 77.3 per 100,000 PY (95% CI, 76.6-7.80) in male patients<br>→ 34.1 per 100,000 PY(95%CI, 33.7-34.5) in female patients
Notes	"National Health Service pathology laboratories are required and all private pathology laboratories in England are recommended to submit all pathology reports of cancer to the NCRAS. These pathology reports are enhanced with information from the Patient Administration System and Cancer Outcomes and Services Dataset to create a cancer record."

cutaneous squamous cell carcinoma (cSCC)

<table>
<tr><td>Author(s)</td><td>G Bianchi, M.; Santos, A.; Cordioli, E.</td></tr>
<tr><td>Titel</td><td>Benefits of Teledermatology for Geriatric Patients: Population-Based Cross-Sectional Study</td></tr>
<tr><td>Year of publication</td><td>2020[64]</td></tr>
<tr><td>Review Question (1/2/3)</td><td>1/3</td></tr>
<tr><td>Aim/purpose/objective</td><td>• Evaluate the proportion of lesions in individuals aged 60 years and older that could be managed using teledermatology in conjunction with primary care physicians
• Assess the most frequent skin lesions, the most common treatments provided to patients, and the distribution and causes of referrals made by the teledermatologists</td></tr>
<tr><td>Design</td><td>Cross-sectional
Interventional</td></tr>
<tr><td>Population (Inclusion/Exclusion Criteria)</td><td>Individuals 60 years and older in Sao Paulo, waiting for an appointment with a dermatologist in July 2017</td></tr>
<tr><td>Methods</td><td>• Platform and mobile app developed by municipal health department and Hospital Israelita Albert Einstein
Standard protocol for taking pictures:
• One photo with enough distance to include the entire part of the body part in question (face, arm, leg, trunk)
• Second photo in close-up, around 15 cm away from the lesion
• Third photo in a lateral view to capture the volume of the lesion
→ then upload on platform with short clinical history and patient data for recruited dermatologists
→ dermatologists would decide between 3 options (biopsie/inpresence appointment; dermatologist consultation; primary care physician)
• Study period/triage done by dermatologists: July 2017 to July 2018</td></tr>
<tr><td>Sample characteristics</td><td>• n = 6320 referrals were made
• n = 12,614 lesions diagnosed</td></tr>
<tr><td>Key findings relevant for review questions</td><td>1.
Most common causes of consultation in individuals 60+ years:
• Seborrheic keratosis - 13.93% (1757/12,614)
• Benign tumors (melanocytic nevus, benign neoplasms, epidermoid cysts) - 14.96% (1889/12,614)
• Pigmentary disorders (solar lentigo and leucoderma) - 10.38% (1309/12,614)
• Actinic keratosis - 4.80% (605/12,614)
• Onychomycosis - 6.77% (854/12,614)
• Warts - 2.12% (267/12,614)
• Xerosis - 3.25% (410/12,614)

<table>
<tr><td></td><td>60-69 years</td><td>70-79 years</td><td>80-89 years</td><td>≥ 90 years</td></tr>
<tr><td>Most frequent diseases</td><td>n</td><td>n</td><td>n</td><td>n</td></tr>
<tr><td>Leuchoderma</td><td>255</td><td>103</td><td>90</td><td>0</td></tr>
<tr><td>Wart(s?)</td><td>176</td><td>67</td><td>23</td><td>1</td></tr>
<tr><td>Xerosis</td><td>261</td><td>103</td><td>39</td><td>7</td></tr>
<tr><td>Solar lentigo</td><td>584</td><td>248</td><td>74</td><td>6</td></tr>
<tr><td>Benign neoplasm</td><td>443</td><td>173</td><td>13</td><td>1</td></tr>
</table></td></tr>
</table>

Epidermoid cyst	309	130	20	2
Onychomycosis	543	261	43	7
Actinic keratosis	300	208	79	18
Melanocytic nevus	572	190	34	2
Seborrheic keratosis	974	590	171	22
**Most frequent group of disease**	%	%	%	%
Benign tumors	26	28	26	21
Pigmentary disorders	14	12	11	6
Eczemas	12	11	12	15
Precancerous/malign tumors	6	10	17	35
Infectious diseases	12	11	10	8

3.

- Patients were sent back to their primary care physicians in 66.7% (8408/12614) of cases of lesions
- 49.8% (3148/6320) of patients were referred back to their primary physicians
- 42.1% (2661/6320) to consultation with an in-presence dermatologist
- 8.1% (511/6320) were referred directly to biopsy
- Most common prescriptions:
    - Emollients 31.2% (909/2856),
    - Topical antifungal 29.5% (843/2856),
    - Sunscreen 27.9% (796/2856),
    - Topical corticosteroids (low and high potency) 24.4% (697/2856),
    - Oral antifungals 7.2% (205/2856),
    - Hydroquinone in 1.3% (38/2856)

- Mean waiting time for face-to-face dermatologist was 6.7 months before the project
- Dropped to 1.5 months during the project (reduction of 78%)

Notes: -

Author(s)	Ching-Yuan Chang, Haesuk Park and Jenny Lo-Ciganic
Titel	The prevalence of sun protective behaviors across different age groups in the US population: Findings from the 2015 US Health Interview Survey
Year of publication	2020[65]
Review Question (1/2/3)	3
Aim/purpose/objective	• To examine the association between age and sun protective behaviors, adjusting for potential confounders
Design	Cross-sectional
Population (Inclusion/Exclusion Criteria)	US adults without any skin cancer from 2015 National Health Interview Survey
Methods	• Age categories: 18-29, 30-49, 50-69, and ≥70 years old<br>• Weighted multivariable logistic regression model<br>→ Adjusted odds ratios (aORs) with 95% confidence interval(95%CI)<br>• Sun protective behaviors included:<br>1)wearing; sun-protective clothing<br>2)shade use<br>3)hat use<br>4)sunscreen use<br>5)infrequent indoor tanning device use
Sample characteristics	• n = 236,234,809 eligible participants (2015)<br>• 12.0% aged ≥70 years
Key findings relevant for review questions	• "When compared with participants aged 18-29, participants aged ≥70 (aOR=6.24, 95% CI =4.07-9.58) were associated with having more sun protective behaviors"<br><br><u>Wearing sun-protective clothing:</u><br>• Aged ≥ 70: OR=3.64, 95% CI=3.21-4.12<br><u>Shade use:</u><br>• Aged ≥ 70: OR=2.14, 95%CI=1.88-2.43)<br><u>Using indoor tanning device:</u><br>• Aged ≥ 70: OR=0.22, 95% CI=0.17-0.28)
Notes	• Conference Abstract<br>• Not clearly stated if "n = 236,234,809 eligible participants" is the actual final sample<br>• "This cross-sectional study included a nationally representative of US adults without any skin cancer from the 2015 National Health Interview Survey"

Author(s)	IHJ Everink, J Kottner, JCM van Haastregt, R Halfens, JMGA Schols
Titel	Skin areas, clinical severity, duration and risk factors of intertrigo: A secondary data analysis
Year of publication	2020[66]
Review Question (1/2/3)	1
Aim/purpose/objective	• "To describe the skin areas most often affected by intertrigo, the clinical severity and duration of intertrigo at these skin areas and possible risk factors"
Design	Secondary data analysis
Population (Inclusion/Exclusion Criteria)	Data sets generated by the International Prevalence Measurement of Care Quality (LPZ – Landelijke Prevalentiemeting Zorgkwaliteit)
Methods	• LPZ data gathered in hospitals, care homes and community care in the Netherlands 2013 to 2016 was analysed<br>• In community setting a sample is measured in a time period of four consecutive days<br>• In this analysis only participants that participated in in-depth intertrigo module
Sample characteristics	• Total sample (n = 7865)<br>• Mean age (SD): 80.1 (12.6)<br>• Median age (IQR): 83 (75.0–88.0)<br>• n = 234 (3.0%) in community care/ cared for at home
Key findings relevant for review questions	• n = 22/234 had intertrigo in community care
Notes	-

<table>
<tr><td>Author(s)</td><td>Fors, M.; Gonzalez, P.; Viada, C.; Falcon, K.; Palacios, S.</td></tr>
<tr><td>Titel</td><td>Actinic keratoses in subjects from la Mitad del Mundo, Ecuador</td></tr>
<tr><td>Year of publication</td><td>2020[67]</td></tr>
<tr><td>Review Question (1/2/3)</td><td>1</td></tr>
<tr><td>Aim/purpose/objective</td><td>To assess the prevalence of actinic keratosis (AK) and non melanoma skin cancer in a rural area of Quito.
To evaluate the relationship of AKs with age, sex, ethnicity,education level, alcohol consumption, smoking history and sun exposure habits.</td></tr>
<tr><td>Design</td><td>Cross-sectional</td></tr>
<tr><td>Population (Inclusion/Exclusion Criteria)</td><td>Adult population of seven towns located in la Ruta Escondida de la Mitad del Mundo, Ecuador
Inclusion:
40 years or older</td></tr>
<tr><td>Methods</td><td>Full-body skin examinations
Clinical diagnosis with dermoscopy aid
Skin biopsy to confirm diagnosis
Prevalence and logistic regression</td></tr>
<tr><td>Sample characteristics</td><td>n = 254 Ecuadorian adults (total)
n = 96 (>65)
71.3% female
Mean age: 60.8 ± 13.8 years
Median age: 60 years</td></tr>
<tr><td>Key findings relevant for review questions</td><td>Actinic keratoses more frequent in subjects older than 65 years
<table>
<tr><td></td><td colspan="2">Actinic Keratoses</td></tr>
<tr><td>Age group</td><td>No
n = 197 (77.6%)
No. (%)</td><td>Yes
n = 57 (22.4%)
No. %</td></tr>
<tr><td>40-65</td><td>127 (80.4)</td><td>31 (19.6)</td></tr>
<tr><td>>65</td><td>70 (72.9)</td><td>26 (27.1)</td></tr>
</table></td></tr>
<tr><td>Notes</td><td>-</td></tr>
</table>

Author(s)	Jan Kottner, Irma Everink, Jolanda van Haastregt, Ulrike Blume-Peytavi, Jos Schols
Title	Prevalence of intertrigo and associated factors: A secondary data analysis of four annual multicentre prevalence studies in the Netherlands
Year of publication	2020[68]
Review Question (1/2/3)	1
Aim/purpose/objective	• To measure the prevalence of intertrigo in hospitals, care homes, and home care and to identify demographic and health characteristics being associated with intertrigo.
Design	Secondary data analysis
Population (Inclusion/exclusion Criteria)	Subjects living in care homes, hospitals, and home care organizations in the Netherlands who underwent skin examinations for intertrigo in the context of four annual cross-sectional prevalence studies from 2013 to 2016.<br>Inclusion:<br>• Age ≥18 years
Methods	• Complete skin inspection regarding intertrigo presence/absence<br>• Analysis of prevalence studies being part of International Prevalence Measurement of Care Quality (LPZ) project.<br>→ Two questions about intertrigo (presence and when occurred)<br>→ One trained nurse rater conducted head-to-toe skin examinations in home care clients.<br>→ Sample of home care clients was assessed
Sample characteristics	• Total sample: n= 40,340<br>→ Care homes n= 24,987<br>→ General hospital n= 8724<br>→ **Home care n = 3410 (8.5%)**<br>→ University hospital n= 2629<br>• Mean age (SD): 78.1 (14.0)<br>• Median (IQR): 82.0 (72.0 to 88.0)
Key findings relevant for review question(s)	Prevalence of intertrigo in home care:<br>→ 326/3410 = 9.6% (95% CI 8.6% to 10.6%).
Notes	-

Author(s)	S. Prasad; N. Hussain; S. Sharma; Ch; S. y; J. Kurien
Titel	Impact of Pressure Injury Prevention Protocol in Home Care Services on the Prevalence of Pressure Injuries in the Dubai Community
Year of publication	2020[69]
Review Question (1/2/3)	1/3
Aim/purpose/objective	• To assess the impact of the newly developed pressure injuries (PIs) prevention protocol for home care patients in Dubai.
Design	Longitudinal/cohort<br>Interventional
Population (Inclusion/exclusion Criteria)	Home care patients registered in 13 primary health care centers in Dubai, UAE (n = 1,200).<br>Inclusion:<br>• Compromised mobility (bedbound/chairbound)<br>• Comorbid chronic debilitating illness like diabetes, hypertension, cerebrovascular accidents<br>• 75.5 ± 14.5 years of age
Methods	• Assessment of PIs prevalence and incidence before and after 6 months of implementing the PIs prevention protocol using a standardized skin assessment scale (Braden Scale):<br>→ 1st phase, before protocol implementation: Data collection for patients at risk for pressure injuries from January to September 2018.<br>→ 2nd phase, 6 months after protocol implementation: Data collection from October 2018 to July 2019.<br>• Home care nurses recorded prevalence and incidence data on standardized, validated data collection sheet/tools daily<br>→ Monthly prevalence and incidence rates were calculated by dividing the number of patients identified with new and existing PIs over the total number of home care patients at risk of PI.<br>• Assessment of staff attitude, compliance, potential barriers for implementation
Sample characteristics	• n = 249<br>• 75.5 ± 14.5
Key findings relevant for review question(s)	**1.**<br>• Prevalence rate: 9.0 to 12.0%<br>• Incidence rate: 4.0 to 6.0% (figure 1: 3.0 to 5.0%)<br>**3.**<br>• Incidence and prevalence rate: app. 2.0%<br>• The prevalence and incidence of PIs “declined rapidly” about 3 months after the protocol implementation. “This window period was the time taken by the home care nurses to familiarize and incorporate the protocol steps into their routine home care practice.”<br>• Overall protocol compliance: 76.7%
Notes	„The most significant positive finding noted was the nearly 100.0% compliance to use of Braden Scale to quantify the PI risk for all home care patients, consistent instructions for repositioning, and an appropriate care plan addressing the identified risk groups.”

Author(s)	E. Sideris; S. J. Thomas
Titel	Patients' sun practices, perceptions of skin cancer and their risk of skin cancer in rural Australia
Year of publication	2020[70]
Review Question (1/2/3)	3
Aim/purpose/objective	• To examine adults' skin cancer knowledge, sun behaviors and demographic factors in a regional Australian town
Design	Cross-sectional
Population (Inclusion/exclusion Criteria)	Patients of Mudgee General Practice<br>Inclusion:<br>• ≥ 18 years
Methods	• Patients completed a 20-question survey regarding their sun practices, any risky or protective sun behaviours undertaken, participants' knowledge and awareness of skin cancer<br>• Data collection: October 2015
Sample characteristics	• n = 179, (1.67 females to every male participant)<br>• Age range: 18-89 years<br>• 20.11% aged 61 to 75;<br>• 10.61% aged over 75<br>• Self-reported skin type: *fair* or *tanned* (47.89% and 49.72%), 2.79% of participants reporting *dark* skin type
Key findings relevant for review question(s)	• "Males and those at greatest current risk of skin cancer in Australia, those over 60 years, had the poorest skin cancer knowledge and were least likely to recognize skin cancer or use some sun protection measures".<br>• 75% of 61-75 years, 42.1% of >75 years old participants identified *melanoma* as skin cancer correctly<br><br>Identification of risk factors:<br>→ Changing lesion: 75% of 61-75 years, 36.8% of >75 years old participants<br>→ Odd-shaped lesion: 41.7% of 61-75 years, 21.1% of >75 years old participants<br>→ Previous skin cancer: 50% of 61-75 years, 47.4 % of >75 years old participants<br>→ Sunburn: 72.2 % of 61-75 years, 73.7 % of >75 years old participants<br>→ Fair skin: 58.3 % of 61-75 years, 36.8 % of >75 years old participants<br>→ Family history of skin cancer: 22.2 % of 61-75 years, 10.5 % of >75 years old participants
Notes	-

Sun protection behavior (used)	61-75 years (%)	>75 years (%)
Sunscreen	52.8	36.8
Sunglasses	52.8	47.4
Hat	83.3	84.2
Clothing	55.6	63.2
Shade	52.8	36.8
Nothing	2.8	0

Author(s)	S. P. Sinikumpu; J. Jokelainen; A. K. Haarala; M. H. Keranen; S. Keinanen-Kiukaanniemi; L. Huilaja
Titel	The High Prevalence of Skin Diseases in Adults Aged 70 and Older
Year of publication	2020[71]
Review Question (1/2/3)	1/2
Aim/purpose/objective	• To determine the prevalence of skin diseases in adults aged > 70<br>• To study the association between cutaneous diseases and socioeconomic status, sex, and living status in the older population.
Design	Cross-sectional
Population (Inclusion/exclusion Criteria)	The surviving mothers and fathers of the Northern Finland Birth Cohort 1966 (NFBC1966) *<br>Inclusion:<br>• Living in the Oulu area
Methods	• Time period skin data collection: May 2018 to March 2019<br>• Dermatological whole-body skin examination in a 20-minute visit.<br>• Diagnoses of skin disorders classified by the International Classification of Diseases, Tenth Revision (ICD-10).<br>• Calculation of the overall prevalence of the skin diseases.<br>• Skin findings, severity of skin diseases were classified in 3 categories:<br>(1) no further care needed,<br>(2) expected to recover with self-treatment<br>(3) need medical care by physician
Sample characteristics	• n = 552, 62.7% females<br>• Mean age= 78.4 years (SD 4.18)<br>• Age range: 70 – 93 years

Key findings relevant for review question(s)	**2.**<br>• "75.7% of the study population had at least one skin disease that required treatment or follow-up."<br>• "More than one-third of the study cases (39.1%) had three or more skin diseases, with fungal skin infections the most common."<br><br>Treatment requirement**, n (%):<br>• No further care needed: 132 (24.3)<br>• Self-treatment needed: 177 (32.6)<br>• Requires further care by physician: 234 (43.1)<br>** not included: benign skin tumors, pattern hair loss<br>**1.**<br>Prevalence of skin diseases

**Skin Condition**	**Patients, n (%)**
Melanocytic nevi	276 (50.1%)
Cherry angiomas	348 (63.2%)
Seborrheic keratosis	434 (78.8%)
Lentigo senilis	383 (69.5%)
Actinic keratosis	123 (22.3%)
Bowen's disease	9 (1.63%)
Melanoma	3 (0.54%)
Basal cell carcinoma	28 (5.07%)
Squamous cell carcinoma	2 (0.36%)
Atopic dermatitis	2 (0.36%)
Hand eczema	47 (8.51%)
Seborrheic dermatitis	56 (10.1)
Nummular eczema	51 (9.24%)
Contact eczema	4 (0.72)
Neurodermatitis	7 (1.27%)
Asteatotic eczema	115 (20.8%)
Psoriasis	6 (1.09%)
Lichen planus	14 (2.54%)
Dermatitis herpetiformis	0 (0%)
Discoid lupus erythematosus	0 (0%)
Vitiligo	11 (1.99%)
Urticaria	4 (0.73%)
Bullous pemphigoid	0 (0%)
Rosacea	137 (25.0%)
Pityriasis versicolor	6 (1.09%)
Onychomycosis	165 (29.9%)
Tinea pedis	268 (48.6%)
Tinea corporis	8 (1.45%)
Folliculitis	20 (3.62%)
Moisture-associated skin damage	50 (9.06%)
Verruca plantaris	21 (3.8%)
Verruca palmaris	13 (2.36%)
Alopecia areata	5 (0.91%)
Androgenetic alopecia	171 (83.0%)
Female pattern hair loss	78 (22.5%)
Hyperhidrosis	3 (0.54%)
Hidradenitis suppurativa	1 (0.18%)

Notes	* The NFBC1966 is composed of the offsprings of the mothers who lived in the two northernmost provinces in Finland (Oulu or Lapland) and whose expected delivery date fell between January 1 and December 31, 1966.

Author(s)	Selin Tokez, Marlies Wakkee, Marieke Louwman, Eline Noels, Tamar Nijsten, Loes Hollestein
Titel	Assessment of Cutaneous Squamous Cell Carcinoma (cSCC) In situ Incidence and the Risk of Developing Invasive cSCC in Patients With Prior cSCC In situ vs the General Population in the Netherlands, 1989-2017
Year of publication	2020[72]
Review Question (1/2/3)	1
Aim/purpose/objective	• To estimate annual population-based age-standardized incidence rates of histopathologically confirmed cSCC in situ stratified by sex, age, and body site<br>• To assess the risk of developing invasive cSCC among patients with cSCC in situ compared with the general population
Dsign	Analyses of register data
Population (Inclusion/exclusion Criteria)	• Cases of histopathologically confirmed skin cancers in the *Netherlands Cancer Registry (NCR)<br><br>Inclusion:<br>• First incident (histopathologically confirmed) cSCC in situ<br>Exclusion:<br>• "… any patients with cSCC in situ who received treatment solely from a general practitioner or pathologist were excluded to ensure reliable trend analyses"
Methods	• Time period analysed: 01.01.1989 to 31.12.2017<br>• Data from patients with a diagnosis of Bowen disease or carcinoma in situ were retrieved.<br>• ICD-O-3 codes were used.<br>• Annual crude and age-standardized incidence rates per 100,000 person-years were calculated using the population size obtained from Statistics Netherlands.<br>• The European Standard Population, 2013 edition was used to calculate European standardized rates.<br>• Estimation of the risk of developing invasive cSCC during follow-up.
Sample characteristics	• n = 13,246 cases 70 to 79 years of age<br>• n = 9,529 cases ≥ 80 years of age<br>• Median age at diagnosis for males: 73 (IQR, 65-80)<br>• Median age at diagnosis for females: 75 years (IQR, 67-82 years)
Key findings relevant for review question(s)	European Standardized rates of cSCC in situ per 100,000 person-years from 1989 to 2017:<br>For men:

Age	Period							
	1989-1993	1994-1998	1999-2003	2004-2008	2009-2013	2014-2015	2016	2017
70-79	47.2	63.7	67.9	88.2	125.5	129.8	200.0	273
≥ 80	77.5	110.8	122.5	153.3	202.3	213.8	373.6	540.9

For women:

Age	Period							
	1989-1993	1994-1998	1999-2003	2004-2008	2009-2013	2014-2015	2016	2017
70-79	38.4	55.1	65.5	94.5	136.6	152.5	232.0	299.9
≥ 80	68.5	87.8	110.4	154.0	207.2	223.5	341.9	482.7

	• "The highest rates for both sexes were found in 2017 among the oldest age group (≥80 years)… "
Notes	• *The Netherlands Cancer Registry (NCR) registers all cases of histopathologically confirmed skin cancers, including cases of cSCC in situ.<br>• There was a change in registration methods in 2016

Author(s)	Hung Fu Tseng, Katia Bruxvoort, Bradley Ackerson, Yi Luo, Hilary Tanenbaum, Yun Tian, Chengyi Zheng, Bianca Cheung, Brandon J. Patterson, Desiree Van Oorschot, and Lina S. Sy
Titel	The Epidemiology of Herpes Zoster in Immunocompetent, Unvaccinated Adults ≥50 Years Old: Incidence, Complications, Hospitalization, Mortality, and Recurrence
Year of publication	2020[73]
Review Question (1/2/3)	1 (RQ1),2 (RQ2)
Aim/purpose/objective	• To estimate the incidence of Herpes Zoster, the proportion of Herpes Zoster-related complications, the proportion of HZ patients that require hospitalization, the Herpes Zoster case-fatality rate, and the Herpes Zoster recurrence rate, among an immunocompetent and Zoster vaccine live (ZVL) unvaccinated population aged ≥50 years.
Design	Secondary data analysis of electronic health records (EHRs)
Population (Inclusion/exclusion Criteria)	Data of EHRs of *Kaiser Permanente Southern California (KPSC)<br>Inclusion:<br>• Immunocompetent adults (Exclude: HIV, leukemia, lymphoma diagnoses, immunosuppressing agents in prior year)<br>• Age ≥50 years<br>• No vaccination with Zoster Vaccine Live (ZVL)<br>• Members of KPSC for at least 1 year prior to entering cohort
Methods	• Incident herpes zoster (HZ) patients identified via (ICD)-9 codes 053.xx (or ICD-10 codes B02.xx beginning from October 2015)<br>• Follow-up until: (1) incident HZ, (2) receipt of ZVL, (3) end of membership with KPSC (including death), (4) 31 December 2015<br>• Person-time accumulated from each individual during the interval was summed as the denominator and counted for each age category.<br>• Sample of patients with HZ diagnosis and min. 2 additional HZ-related medical encounters within 6 months was further reviewed for proportion of complications<br>• HZ-related hospitalizations: Review of medical records for all HZ patients in 2015<br>• HZ-related death determined from automated data and electronic health records<br>• Recurrent HZ identified from a cohort initially diagnosed with HZ in 2007–2008 and followed through 2016
Sample characteristics	• Total sample size: n= 40,893 (all incident patients)<br>• Incident patients ≥ 60 years: n = 24,551
Key findings relevant for review question(s)	• Herpes Zoster incidence is increasing with age

Age	Incidence rate (RQ1)	Cutaneous complica-tions* (RQ2)	Neurological complica-tions* (RQ2)	Other complica-tions* (RQ2)
60-64 years	10.21/1000 person-years (95% CI, 9.98–10.44).	4.97 (1.13–8.81)	0.83 (.00–2.44)	0.83 (.00–2.44)
65-69 years	11.72/1000 person-years (95% CI, 11.42–12.03)	8.82 (2.66–14.97)	1.26 (.00–3.71)	0

70-74 years	12.87 /1000 person-years (95% CI, 12.48–13.27)	8.11 (3.08–13.15)	0.90 (.00–2.66)	2.70 (.00–5.71)
75-79 years	14.28 /1000 person-years (95% CI, 13.8–14.79)	13.28 (5.30–21.25)	0	2.95 (.00–6.97)
≥ 80 years	13.99 /1000 person-years (95% CI, 13.57–14.42)	11.60 (6.86–16.33)	1.74 (.00–3.69)	1.74 (.00–3.69)

*Complications: Confirmed cases and weighted proportions, among incident HZ patients

Hospitalisation rate (RQ2):

- “The hospitalization rate among the population was 8.49/100 000 person-years (95% CI, 6.72–10.73) and sharply increased with age.“
  - → 9.74 in 60-64 year old, 39.57/100,000 person-years in ≥80 year old
- “The case-fatality rate of HZ was extremely low.”

Notes	*Kaiser Permanente Southern California (KPSC): “Integrated health care organization that provides prepaid comprehensive health care to more than 4.6 million members […] The demographic composition at KPSC is representative of the Southern California population.”

Author(s)	Yik Weng Yew, Amanda Hui Yu Kuan, Lixia Ge, Chun Wei Yap, Bee Hoon Heng
Titel	Psychosocial impact of skin diseases: A population-based study
Year of publication	2020[74]
Review Question (1/2/3)	1
Aim/purpose/objective	Psychosocial burden of skin diseases among a general multi-racial population cohort in Singapore
Design	Cross-sectional
Population (Inclusion/Exclusion Criteria)	Participants who took part in first-year follow-up survey of Population Health Survey (Nov. 2016 - Feb.2018)<br>Inclusion:<br>• Singapore citizens/permanent residents in this area ≥ 6 months<br>• ≥ 21 years old<br>• Ability to answer survey questions coherently
Methods	• Surveyor-administered face-to-face interviews
Sample characteristics	• Participants 60-74 years: n = 419<br>• Participants 75+: n = 188
Key findings relevant for review questions	**Any skin disease in the list:**<br>• Total “no” n = 1,163, “yes” n = 347<br>• Age group 60-74, No.(%): No, n = 338 (29.1); Yes, n = 81 (23)<br>• Age group 75+, No.(%): No, n = 131 (11.3); Yes, n = 57 (16.4)<br><br>Skin disease by age group, n (%) (see table below)
Notes	*Population Health Survey : “Representative sample of community-dwelling adult population of central region of Singapore”

Skin disease by age group, n (%)

**Skin disease**	**60-74 years**	**75+ years**
Eczema	27 (6.4)	18 (9.6)
Acne	7 (1.7)	1 (0.5)
Psoriasis	2 (0.5)	3 (1.6)
Vitiligo	4 (1)	1 (0.5)
Viral warts	6 (1.4)	4 (2.1)
Scabies	1 (0.2)	1 (0.5)
Fungal skin infections	12 (2.9)	10 (5.3)
Chronic urticaria	4 (1)	2 (1.1)
Bacterial skin infections	36 (8.6)	26 (13.8)
Chronic ulcers	14 (3.3)	4 (2.1)
Skin cancer	0	3 (1.6)
Alopecia areata	0	1 (0.5)
Unpecified symptom of pruritus	60 (14.3)	34 (18.1)

Author(s)	Shin Shen Yong, Zhenli Kwan, Chin Chwen Ch'ng, Adrian Sze Wai Yong, Leng Leng Tan, Winn Hui Han, Shahrul Bahyah Kamaruzzaman, Ai-Vyrn Chin and Maw Pin Tan
Titel	Self-reported generalised pruritus among community-dwelling older adults in Malaysia
Year of publication	2020[75]
Review Question (1/2/3)	2
Aim/purpose/objective	Burden of generalised pruritus and factors associated with the presence and severity of pruritus among the community-dwelling older population
Design	Longitudinal
Population (Inclusion/Exclusion Criteria)	Study population of first and second wave of "Malaysian Elders Longitudinal (MELoR) study"<br>Inclusion:<br>• Community-dwelling older adults ≥ 55 years
Methods	• Participants were identified from electoral rolls of the parliamentary constituencies of Petaling Jaya North, Petaling Yaya South and Pantai Valley<br>• Detailed home-based computer-aided questionnaire interview<br>• Question if subjects experienced pruritus in preceding week<br>If Yes<br>→ Localised or generalised?<br>→ Severity of pruritus on NRS(incl. VAS) 0-10
Sample characteristics	• n = 1623 recruited<br>• n = 1349 included in follow-up<br>• **n = 770** completed follow-up<br>• Median age 69.86 years
Key findings relevant for review questions	• Participants reporting generalised pruritus: Median age 70.81 years<br>• Participants without generalised pruritus: Median age 69.83 years<br>• Participants reporting generalised pruritus: n = 43 (5.97 %)<br>➔ Participants reporting severe or very severe pruritus: n = 21 (45.7%)<br><br>• Subjects 65 - 74 years: 5.9% (n = 22) reported generalised pruritus<br>• Subjects 75 - 84 years: 6.4% (n = 11) reported generalised pruritus<br>• Subjects 85+ years: none reported generalised pruritus<br><br>• Association between Poor Pittsburgh Sleep Quality Index (PSQI) and generalised pruritus (p = 0.04)<br>• No association between generalised pruritus and general health or psychological comorbidities.
Notes	-

Author(s)	Ruhai Bai, Hui Huang , Minmin Li , and Meng Chu
Titel	Temporal Trends in the Incidence and Mortality of Skin Malignant Melanoma in China from 1990 to 2019
Year of publication	2021[76]
Review Question (1/2/3)	1/2
Aim/purpose/objective	• To determine the long-term trend in Skin Malignant Melanoma (SMM) incidence and mortality rates in China using the 2019 Global Burden of Disease (GBD) data from 1990 to 2019<br>• Use an age-period-cohort (APC) framework to analyze the corresponding effects on incidence and mortality rate between sexes
Design	Secondary data analysis
Population (Inclusion/Exclusion Criteria)	Global Burden of Disease 2019 data
Methods	• Age Period Cohort analysis based on ICD-9 codes 172–172.9 and ICD-10 codes C43–C43.9, D03–D03.9, D22–D23.9, D48.5)<br>• SMM incidence and mortality standardized using the global age-standardized population of GBD 2019
Sample characteristics	No data
Key findings relevant for review questions	1.<br>• Incidence: At age 60 years around 2/100,000 person-years, and increases to app. 15/100,000 person-years with age 95 years<br>2.<br>• Mortality: At age 60 years around 0.5/100,000 person-years, and increases to app. 5/100,000 person-years with age 95 years
Notes	• Incidence, mortality extracted from figure 3<br>• Data for GBD 2019 for China:<br>→ Surveillance data from China Disease Surveillance Points system (covers 24.3% of the total population of the country since 2013)<br>→ Vital Registration data from the Chinese Center for Disease Control and Prevention (accounting for roughly 8% of the national population)

Author(s)	John S. Barbieri; Olaf Rodriguez; Misha Rosenbach; David Margolis
Titel	Incidence and Prevalence of Granuloma Annulare in the United States
Year of publication	2021[77]
Review Question (1/2/3)	1
Aim/purpose/objective	• Estimate the population-based incidence and prevalence of granuloma annulare in the United States<br>• Understand the most common treatments prescribed for the patients
Design	Secondary data analysis
Population (Inclusion/Exclusion Criteria)	Optum Clinformatics Data Mart Database*
Methods	• Deidentified data was analysed<br>• Entire population was considered at risk while continuously enrolled in the data set<br>• Incident granuloma annulare (GA): ICD-10 code for GA + no prior ICD-10 codes for granuloma GA + at least 180 days of continuous enrollment prior to ICD-10 code for GA<br>• Prevalent GA: ICD-10 code for GA during the calendar year<br>• Medical record review on a sample of 100 with code for GA and 100 without was conducted in the outpatient dermatological and primary care clinics at the University of Pennsylvania Health System between Jan. 2016 and Oct. 2020<br>• Data period analysed: January 1,2017 to December 31,2018
Sample characteristics	Incident, n (%) (Total n = 11608)<br>• ≥ 60 years: 5978 (51.5%)<br>Prevalent, n (%) (Total n = 17862)<br>• ≥ 60 years: 9514 (53.3%)
Key findings relevant for review questions	Mean annualised incidence of GA (“in USA”), age group ≥60:<br>• GA cases, n = 2989<br>• Incidence (95% CI): 54.5/100 000 (52.6-56.5)<br>• Incidence, %: 0.05<br>Mean annualised prevalence of GA (“in USA”), age group ≥60:<br>• GA cases, n = 4757<br>• Prevalence (95% CI): 86.8/100 000 (84.3-89.3)<br>• Prevalence, %: 0.09
Notes	*Optum Clinformatics Data Mart Database: “includes deidentified commercial claims data for approximately 18 million to 20 million covered individuals in the United States annually.” (Medical and pharmacy claims as well as demographic data. “Demographic information of the patient population available in the Optum Clinformatics Data Mart is similar to that of the United States population with respect to sex, age, and geographical distribution”)

Author(s)	Lauro Bucchi, Silvia Mancini, Emanuele Crocetti et al.
Titel	Mid-term trends and recent birth-cohort-dependent changes in incidence rates of cutaneous malignant melanoma in Italy
Year of publication	2021[78]
Review Question (1/2/3)	1
Aim/purpose/objective	To obtain a formal confirmation of the suggested stabilisation and decline of cutaneous malignant melanoma rates in southern Europe by investigating the incidence trends by birth cohort
Design	Analyses of registry data
Population (Inclusion/Exclusion Criteria)	Data from the Italian Association of Cancer Registries (AIRTUM) database<br>Inclusion:<br>• Individuals >15 years and <84 years
Methods	• Age-standardised (2013 European standard population) incidence rates<br>• Primary invasive cutaneous malignant melanoma via ICD-10 codes C43.0 to –C43.9<br>• Estimated annual percentage change (EAPC)
Sample characteristics	• Cases registered between 1994 and 2013: n = 45,264<br>• Cases ≥ 65 years: n = 17,535 (38.8%)
Key findings relevant for review questions	Highest incidence rates in older age-groups:<br>• M (60/65+): 40 to 50/100,000<br>• F (60/65+): 20 to 30/100,000<br><br>• "…rates rose for all cohorts born until the mid-1970s and decreased for supsequent cohorts."<br>• "Incidence rate by cohort increased consistently in all age groups" Exceptions:<br>➔ Women, 70 years and older: "risk of disease was substantially stable over the study period"<br><br>(Younger age groups: "stabilisation and a small decrease in age-specific incidence rates were observed")<br><br>Estimated annual percentage change (EAPC)<br>• EAPC Women 1.5 to 1.6<br>• EAPC Men 4.2 to 4.4
Notes	• Incidence rate presented on figure with semilog- scale<br>ATRIUM database:<br>• 21 registries included for core period 1994 to 2013 analysed<br>• Population covered by included registries: n = 15,814,455 (27.5% of the Italian population)

Author(s)	Karl Philipp Drewitz, Klaus J. Stark, Martina E. Zimmermann, Iris M. Heid, Christian J. Apfelbacher
Titel	Frequency of hand eczema in the elderly: Cross-sectional findings from the German AugUR study
Year of publication	2021[79]
Review Question (1/2/3)	1
Aim/purpose/objective	• Estimate the frequency of HE in the elderly to determine its burden of disease in this particular population
Design	Cross-sectional<br>(Data from cohort AugUR study)
Population (Inclusion/Exclusion Criteria)	Older persons living in Regensburg, Germany and the surrounding county<br>Inclusion:<br>• 70 years and older
Methods	• Recruited between March 2013 and November 2015 in Regensburg, Germany<br>• Thorough medical examination and a standardized in-person interview<br>• Raw frequencies estimated using self-reports on physician-diagnosed HE from standardized personal interview<br>• Frequencies standardized to the Bavarian population weighted by gender and 5-year age-groups
Sample characteristics	• n = 1,133<br>• Mean age 77.6, Age range 70-95 years<br>• 45.1% women
Key findings relevant for review questions	Raw frequencies:<br>• 2.7%, 95% CI 1.6-4.3 reported a previous diagnosis of HE<br>• Frequency of HE was highest in age group 85-95 years (3.5%, 95% CI 0.9-8.6)<br>• Lowest frequency in age group 80-84 years (0.5%, 95% CI 0.0-2.7)<br>• Frequency in age group 75-79 was 3.2% (CI 1.7-5.3)<br>• Frequency in age group 70-74 was 3.0% (CI 1.6-5.2)<br><br>Standardized frequencies (Bavarian population) (95% CI in brackets)<br>• 70-74: 3.4 % (1.8-5.6)<br>• 75-79: 2.3 %(1.7-4.2)<br>• 80-84: 0.5 %(0.0-2.7)<br>• 85-95: 5.1 %(2.0-10.9)<br>• Entire sample: 2.8 % (1.9-3.9)
Notes	-

<table>
<tr><td>Author(s)</td><td>S Madani; S Marwaha; JR Dusendang; S Alexeeff; N Pham; EH Chen; S Han; LJ Herrinton</td></tr>
<tr><td>Titel</td><td>Ten-Year Follow-up of Persons With Sun-Damaged Skin Associated With Subsequent Development of Cutaneous Squamous Cell Carcinoma</td></tr>
<tr><td>Year of publication</td><td>2021[80]</td></tr>
<tr><td>Review Question (1/2/3)</td><td>1</td></tr>
<tr><td>Aim/purpose/objective</td><td>• To assess cutaneous squamous cell carcinoma (cSCC) risk for 10 years after the diagnosis of AK.</td></tr>
<tr><td>Design</td><td>Secondary data analysis</td></tr>
<tr><td>Population (Inclusion/Exclusion Criteria)</td><td>Adults insured with Kaiser Permanente Northern California
Exlcusion:
• <2 years enrollment in health care plan (prior to AK-diagnosis)
• Missing data
• Diagnosis cSCC, melanoma, organ transplant, or HIV prior to index date</td></tr>
<tr><td>Methods</td><td>• AK diagnosis (ICD-9 code 702.0, ICD-10 code L57.0)
→ Recorded during in-person visit with a primary care practitioner or dermatologist
→ January 1, 2009, to December 31, 2019
→ Follow-up through February 29, 2020
• Controls: General health plan members without a diagnosis of AK
→ Randomly selected and individually matched (also on primary care medical center)
• Data collection from electronic medical record</td></tr>
<tr><td>Sample characteristics</td><td>• n = 140,198 per cohort (60 + years)</td></tr>
<tr><td>Key findings relevant for review questions</td><td><table>
<tr><td>Age group</td><td>Proportions % (AK cohort)</td><td>Proportions % (control)</td></tr>
<tr><td>60-69</td><td>8.7%</td><td>3.3%</td></tr>
<tr><td>70-79</td><td>12.9%</td><td>5.3%</td></tr>
<tr><td>≥80</td><td>16.4%</td><td>6.5%</td></tr>
</table></td></tr>
<tr><td>Notes</td><td>• Proportions were calculated with data in table 2 and 4</td></tr>
</table>

Author(s)	A. Memon; P. Bannister; I. Rogers; J. Sundin; B. Al-Ayadhy; P. W. James; R. J. Q. McNally
Title	Changing epidemiology and age-specific incidence of cutaneous malignant melanoma in England: An analysis of the national cancer registration data by age, gender and anatomical site, 1981-2018
Year of publication	2021[81]
Review Question (1/2/3)	1
Aim/purpose/objective	• To determine the changing epidemiology and trends in age-specific incidence of melanoma.
Design	Analyses of registry data
Population (Inclusion/exclusion Criteria)	Registry data from England<br>Inclusion:<br>• Diagnosis of cutaneous malignant melanoma
Methods	• Data collection from Office for National Statistics (ONS)<br>• Time period 1981-2018<br>• (ONS) and Public Health England* (PHE) Office for data release using the ICD-9 and -10 codes for cutaneous malignant melanoma. (ICD-9 1720-1729 and ICD-10 codes: C43.0-C43.9)<br>• Denominator data were obtained from the Population Estimates Unit, ONS.<br>• Calculation of average annual incidence rates of malignant melanoma (per 100,000 population) by age (3 categories: 0-34, 35-64, 65+ years), gender and anatomical site during seven five-year time periods (1981-85 to 2011-15) and the three-year period 2016-18.<br>• Calculation of the percentage change in incidence per time period.<br>• Estimation of the Average Annual Percentage Change (AAPC)
Sample characteristics	• n= 117,107 cases (65+ years) → n = 22,235 (2016-2018)<br>• 45.7% males, 54.3% females
Key findings relevant for review question(s)	Average annual incidence rate (per 100,000 population), 2016-18 in age group (≥ 65 years):<br>→ 60.1 females<br>→ 90.4 males<br>• The average annual incidence rate of cutaneous malignant melanoma was higher in the older age group (□ 65 years) compared to the younger age groups during the study period.<br>• For the older age group (≥ 65 years), there was "a steady and substantial increase" in the average annual incidence rates during the study period.<br>• "In the old age-group (≥ 65 years) the rates increased by +842% (AAPC, 25.7%) in males (from 9.6/100,000 in 1981-85 to 90.4/100,000 in 2016-18) and +381% (AAPC, 11.2%) in females (from 12.5/100,000 in 1981-85 to 60.1/100,000 in 2016-18)".
Notes	*The office is responsible for the collection and collation of registrations from the nine regional population-based cancer registries in England.

<table>
<tr><td>Author(s)</td><td>V. Neena; N. Asokan; R. Jose; A. Sarin</td></tr>
<tr><td>Titel</td><td>Prevalence of eczema among older persons: A population-based cross-sectional study</td></tr>
<tr><td>Year of publication</td><td>2021[82]</td></tr>
<tr><td>Review Question (1/2/3)</td><td>1/2/3</td></tr>
<tr><td>Aim/purpose/objective</td><td>• To estimate the prevalence and types of eczema in people aged ≥65 years in the community.
• To evaluate the effectiveness of community-based interventions for case finding.</td></tr>
<tr><td>Design</td><td>Cross-sectional</td></tr>
<tr><td>Population (Inclusion/exclusion criteria)</td><td>Residents in Puthurkkara division of the Thrissur Municipal Corporation, India
Inclusion:
• Permanent residents or residency for more than a year
• Age ≥ 65 years</td></tr>
<tr><td>Methods</td><td>• Time period: Jan 2018 to Jun 2019
• Two trained health workers surveyed the eligible population via home visits and identified persons likely to have eczema (based on pre-defined features).
• Direct patient interview and examination of the suspected eczema cases by the study investigators/dermatologists.
• Estimation of eczema prevalence and comparison between age groups and sex.</td></tr>
<tr><td>Sample characteristics</td><td>• n= 385 (total)
• n = 98 (examined by dermatologists)</td></tr>
<tr><td>Key findings relevant for review question(s)</td><td>1.
• Prevalence of eczema: 11.4% (44/385).
• The prevalence of eczema was 18.2% among persons aged 81 years or more, 13.1% among those aged 66–70 years and 6.3% in the 71–80 year age group.
<table>
<tr><th>Clinical type of eczema</th><th>N (%)</th></tr>
<tr><td>Asteatotic eczema</td><td>12 (3.1)</td></tr>
<tr><td>Gravitational eczema</td><td>10 (2.6)</td></tr>
<tr><td>Lichen simplex chronicus</td><td>9 (2.3)</td></tr>
<tr><td>Allergic contact dermatitis</td><td>6 (1.6)</td></tr>
<tr><td>Atopic dermatitis, Nummular eczema</td><td>4 (1)</td></tr>
<tr><td>Seborrhoeic dermatitis
Irritant contact dermatitis,</td><td>2 (0.5)</td></tr>
<tr><td>Hand eczema
Infective eczema</td><td>1 (0.26)</td></tr>
</table>
2.
• Symptoms in the study subjects with eczema
→ Pruritus: 100 %
→ Scaling: 50%
→ Oozing: 31.8%
→ Dryness: 29.5%
→ Thickening of skin lesion: 18.2%
3.
• The diagnostic accuracy (precision) of the health workers was 46.3% (44/95)</td></tr>
<tr><td>Notes</td><td>-</td></tr>
</table>

Author(s)	L. Tang; F. Li; F. Xu; S. Yan; J. Zhou; J. Li; W. Fu; J. Chen; J. Xu
Titel	Prevalence of vitiligo and associated comorbidities in adults in Shanghai, China: a community-based, cross-sectional survey
Year of publication	2021[83]
Review Question (1/2/3)	1
Aim/purpose/objective	• To estimate the prevalence of vitiligo and associated comorbidities in adults in Shanghai
Design	Cross-sectional study
Population (Inclusion/exclusion Criteria)	Residents in the urban-rural Beixinjing community of Shanghai<br>Inclusion:<br>• Residency in Shanghai for more than a year<br>• Age ≥ 18 years old
Methods	• Cross-sectional study was conducted October 2009 to January 2010<br>• Recruitment by cluster sampling<br>• Residential apartment buildings were survey units (125-155 people)<br>• 85 buildings randomly selected<br>• Conduction of Face-to-face interviews at home and dermatological examination (by at least two dermatologists).<br>• Calculation of age-adjusted prevalence of vitiligo using the population of Shanghai from the 2010 Shanghai Population Census.
Sample characteristics	• n = 2,912 (≥61 years)
Key findings relevant for review question(s)	Prevalence of vitiligo in adults in Shanghai stratified according to age:<br><table><tr><td>Age range</td><td>Prevalence (%)</td></tr><tr><td>61-70</td><td>1.19</td></tr><tr><td>71-80</td><td>1.59</td></tr><tr><td>≥ 81</td><td>1.20</td></tr></table><br>The prevalence of vitiligo was different among different age groups […], with a lower prevalence in younger participants.
Notes	-

Author(s)	Waldmann, A.; Pritzkuleit, R.; Labohm, L.; Katalinic, A.;
Titel	Epidemiology of cancer in the elderly
Year of publication	2021[84]
Review Question (1/2/3)	1/2
Aim/purpose/objective	• To present a description of cancer epidemiology in the elderly.<br>• To identify the most common cancers, the prognosis and the temporal trends of incidence and mortality in older cancer patients.
Design	Analysis of registry data
Population (Inclusion/exclusion Criteria)	Data of interactive database of the Center for Cancer Registry, Germany<br>Inclusion:<br>• Age: ≥ 40 years
Methods	• Time period analysed: 1999 to 2016<br>• Data on incidence, mortality and 5-year relative survival from the interactive database of the Center for Cancer Registry<br>• Most current data is mean of years "2015/2016"
Sample characteristics	• Males: 65-74 years: n = 2,847 cases<br>• Females: unclear
Key findings relevant for review question(s)	**1.**<br>Melanoma<br>➔ Fifth most frequent diagnosis group (5th place in the ranking) in age group 65-74 years in men<br>➔ Incidence rate 72.4/100,000 males/year<br><br>Melanoma incidence rate ≥ 65 years:<br>• App. 50/100,000 (females)<br>• App. 75 to 100/100,000 (males)<br><br>**2.**<br>Melanoma mortality rate ≥ 65 years:<br>• App. 5 to 20/100,000 (females)<br>• App. 10 to 35/100,000 (males)<br>➔ Increasing with age, highest rate in oldest age group
Notes	Rates are interpreted from figure 1

Author(s)	Katrina Blazek, Erin Furestad, David Ryan, Diona Damian, Pablo Fernandez-Penas, Shannon Tong
Titel	The impact of skin cancer prevention efforts in New South Wales, Australia: Generational trends in melanoma incidence and mortality
Year of publication	2022[85]
Review Question (1/2/3)	1/2/3
Aim/purpose/objective	• Trends in melanoma incidence and mortality in New South Wales<br>• Impact of skin cancer prevention
Design	Analyses of registry data
Population (Inclusion/Exclusion Criteria)	Cases of NSW Cancer Registry
Methods	• Patients diagnosed with first primary melanoma (ICD-O-3 C44 and morphology code 8720-8790)<br>• Time period, invasive melanoma: 1988-2014<br>• Time period, in situ melanoma: 2002-2014<br>• Only first primary melanoma counted<br>• Only invasive included in case of in situ and invasive melanoma<br>• Yearly rates: direct age-standardised using 2001 Australian standard population<br>• Incidence and mortality trends (annual percentage change (APC))<br>• Cohort rates<br>• Age-period-cohort model<br><br>Information on mortality:<br>• NSW Cancer Registry with NSW Registry of Births, Deaths and Marriages<br>• Australian Coordinating Registry Cause of Death Unit Record File (COD URF)
Sample characteristics	• n = 69,136 cases (≥60 years of age)
Key findings relevant for review questions	**1.**<br>**Age-standardised incidence rate (per 100,000)**<br>**In situ 2002-2014, ≥ 60 years**<br>• Highest incidence rates in age group 60+ years, with males higher rates than females<br>**Age-standardised incidence rate (per 100,000)**<br>**Invasive 1988-2014, ≥ 60 years**<br>• Highest incidence rates in age group 60+ years, with males higher rates than females<br>→ F: 100 to 150/100,000<br>→ M: 250 to 300/100,000<br><br>**Age-standardised incidence rate (per 100,000)**<br>**In situ 2002-2014, ≥ 60 years**<br>• Incidence rate increased over time period (all age groups, both sexes)<br>• Annual percentage change (APC): Females: 9.1 %; males: 9.2 %

	**Age-standardised incidence rate (per 100,000)**<br>**Invasive 1988-2014, ≥ 60 years**<br>• Females: Incidence rate increased by 2.3% per year (strongest increase)<br>• Males: Incidence rate increased by 2.8% from 1988 to 2005, then stabilising trend (APC 0.6)<br><br>**2.**<br>**Invasive melanoma mortality trends over the period 1988–2014, ≥ 60 years**<br>• Highest rate in age group 60 years and older<br><br>**Age-standardised mortality rate (per 100,000)**<br>**1988-2014, ≥ 60 years**<br>• Females: stable (APC 0.5)<br>• Males: APC of 1.8% (significant) from 1988 to 2009, then decrease of 2.1% per year (non-significant)<br><br>**3.**<br>**Cohort rates**<br>• In situ melanoma incidence rate increased for both sexes -> "each successive cohort had a higher incidence rate"<br>• Invasive melanoma incidence rates increased for cohorts born before 1950, then stabilised for cohorts born 1950 – 1970.<br><br>**Age-period-cohort model**<br>• Rate ratios increased for birth cohorts 1905 to 1945, after it stabilised for cohorts until 1970<br><br>**Discussion**<br>• Significantly decreasing in invasive melanoma in cohorts aged <40 years<br>• Indicates that "cohorts exposed to extensive skin cancer prevention efforts are experiencing a reduction in the burden of disease due to skin cancer compared to earlier age cohorts."<br>• Incidence in NWS started to stabilise from 1950 onwards and declined from 1970 vs. incidence rates started to stabilise in Queensland from 1960 and declined from 1980 birth cohort<br>• Nationwide skin cancer prevention campaigns in Australia in 1980s, additionally state-wide campaigns in 80s and 90s in NSW vs. state-wide campaigns in Queensland beginning in 2000s
Notes	• Incidence, mortality and cohort rates based on figures using log scales

Author(s)	Sofia H. C. Botvid, Lone Storgaard Hove, Marie Balslev Backe, Nils Skovgaard, Michael Lynge Pedersen, Carsten Sauer Mikkelsen
Titel	Low prevalence of patients diagnosed with psoriasis in Nuuk: a call for increased awareness of chronic skin disease in Greenland
Year of publication	2022[86]
Review Question (1/2/3)	1
Aim/purpose/objective	• To estimate the age- and gender-specific prevalence of psoriasis in Nuuk, Greenland.<br>• To describe and compare basic characteristics of the patients to an age- and gender-matched control group.
Design	Secondary data analysis
Population (Inclusion/Exclusion Criteria)	Data from electronic medical record (EMR) used in Greenland<br>Inclusion:<br>• permanent address in Nuuk<br>• diagnosis code of psoriasis S91 (ICPC-2) or DL40 (ICD-10)
Methods	• Data extracted from the electronic medical record (EMR) used in Greenland<br>→ 1st January 2022<br>→ age- and gender- matched control group identified<br><br>Background population:<br>• obtained from Statistics Greenland<br>• all people living in Nuuk by January 1st, 2022<br>→ n = 19,261 (total population of Nuuk)
Sample characteristics	• n = 175 (79 females and 96 males) (0.9%) diagnosed with psoriasis<br>• n = 37 diagnosed with psoriasis and ≥ 60 years of age
Key findings relevant for review questions	See table below.<br>Age- and gender specific prevalence (%)<br>n/N = number of patients/population
Notes	-

Age-group	Females % (95% CI) (n/N)	Males % (95% CI) (n/N)	Total % (95% CI) (n/N)
60-69 years	0.8 (0.2–1.4) (7/844)	1.8 (1.0–2.6) (19/1.054)	1.4 (0.9–1.9) (26/1,898)
70-79 years	2.1 (0.3–3.9) (5/241)	1.9 (0.3–3.5) (5/269)	2.0 (0.8–3.2) (10/510)
80+ years	0.0 (0.0–0.0) (0/58)	1.7 (−1.6–4.9) (1/60)	0.9 (−0.8–2.5) (1/118)

Author(s)	Siew Eng Choon, Alison K. Wright, Christopher E.M. Griffiths, Kwee Eng Tey, Kit Wan Wong, Yoong Wei Lee, Ushananthiny Suvelayutnan, Jeevitha Mariapun, Darren M. Ashcroft, and on behalf of the Global Psoriasis Atlas
Titel	Incidence and prevalence of psoriasis in multiethnic Johor Bahru, Malaysia: a population-based cohort study using electronic health data routinely captured in the Teleprimary Care (TPC®) clinical information system from 2010 to 2020*
Year of publication	2022[87]
Review Question (1/2/3)	1
Aim/purpose/objective	• Analyse trends in the incidence and prevalence of psoriasis over an 11-year period from 2010 to 2020<br>• Determine whether there were any variations in the prevalence and incidence by sex, age and ethnicity
Design	Secondary data analysis
Population (Inclusion/Exclusion Criteria)	Individuals registered with Teleprimary Care (TPC®) in Johor Bahru, Malaysia<br><br>Exclusion:<br>• non-Malaysian patients and patients with only pustular psoriasis without associated plaque psoriasis
Methods	• Study period 2010 – 2020<br>• Data analyses regarding prevalence and incidence (annual prevalence and incidence)<br>• Person-time at risk<br>• Denominator: any patient who contributed at least 1 day in TPC in the year of interest
Sample characteristics	• n = 1,164,724 individuals had been registered with TPC®<br>• n = 3,932 patients with dermatologist-confirmed psoriasis from 2010 to 2020
Key findings relevant for review questions	see table below

Key findings relevant for review questions:

Year	60-69		70+	
	**Prev†**	**Incid‡**	**Prev†**	**Incid‡**
Overall	0.66	45.9 (39.6-52.1)	0.48	39.9 (32.4-47.4)
2010	0.40	46.8 (27.6-79.4)	0.39	41.8 (20.5-85.4)
2011	0.46	49.9 (30.8-81.1)	0.39	49.9 (26.9-92.6)
2012	0.51	39.5 (23.6-66.2)	0.41	40.1 (20.6-77.9)
2013	0.55	52.8 (34.4-81.1)	0.42	40.7 (21.6-76.8)
2014	0.61	36.5 (22.2-60.1)	0.42	40.1 (21.6-74.5)
2015	0.65	45.0 (29.1-69.4)	0.46	45.3 (25.5-80.5)
2016	0.71	49.0 (32.5-73.7)	0.52	47.8 (27.8-82.2)
2017	0.75	37.8 (23.7-60.4)	0.51	45.6 (26.5-78.4)
2018	0.78	36.8 (22.8-59.3)	0.52	36.4 (19.9-66.4)
2019	0.81	46.0 (29.6-71.5)	0.58	39.2 (21.9-70.3)
2020	0.83	68.6 (46.4-101.4)	0.61	16.0 (6.0-42.3)

† Prevalence measures: percentage values
‡ Incidence rate per 100,000 person-years (95% CI);

Notes	TPC:<br>• represents around 98% of the estimated population in Johor Bahru<br>• Locally developed clinical information system linking public primary and secondary care facilities

Author(s)	L Lu, L Chen, Y Xu, A Liu
Titel	Global incidence and prevalence of bullous pemphigoid: A systematic review and meta-analysis
Year of publication	2022[88]
Review Question (1/2/3)	1
Aim/purpose/objective	• Estimate the overall incidence and prevalence of BP, in terms of its spatial and population distributions and among various income level
Design	Secondary data analysis
Population (Inclusion/Exclusion Criteria)	Inclusion:<br>• Original observational studies;<br>• Reporting of incidence and/or prevalence of BP;<br>• Including data that could be extracted to calculate the incidence and/or prevalence (e.g., sample size, number of BP, incidence and/or prevalence)<br>• Reported age and gender-specific prevalence and/or incidence.<br>Exclusion:<br>• Duplicate data and retained the most recent and complete articles<br>• Studies that reported the prevalence and/or incidence in specific disease groups (e.g., autoimmune blister disease population) instead of the general population.
Methods	Systematic review<br>➔ Inception of databases to July 1, 2021
Sample characteristics	• n = 57 articles<br>• n = 10 articles (age-specific incidence)
Key findings relevant for review questions	• Age-specific incidence 60–69: 0.004 per 1000 person-years (95% CI: 0.004–0.004)<br>• Age-specific incidence 70–79: 0.007 per 1000 person-years (95% CI: 0.007–0.008)<br>• Age-specific incidence 80–89: 0.011 per 1000 person-years (95% CI: 0.011–0.012)<br>• Age-specific incidence 90+ : 0.017 per 1000 person-years (95% CI: 0.015–0.018)
Notes	Prevalence not reported by age group

Author(s)	M. Matsumoto; S. Wack; M. A. Weinstock; A. Geller; H. Wang; F. X. Solano; J. M. Kirkwood; L. K. Ferris
Title	Five-Year Outcomes of a Melanoma Screening Initiative in a Large Health Care System
Year of publication	2022[89]
Review Question (1/2/3)	1/3
Aim/purpose/objective	• To compare thickness-specific incidence of melanoma in screened vs unscreened patients following the initiation of a primary care–based skin cancer screening initiative.
Design	Cohort/longitudinal
Population (Inclusion/exclusion Criteria)	Patients presented to a University of Pittsburgh Medical Center (UPMC)-employed primary care clinician for a primary care visit<br>Inclusion:<br>• Age: ≥ 35 years<br>Exclusion:<br>• Screening date prior to first primary care visit<br>• Patients with a diagnosis of melanoma prior to the first primary care visit.
Methods	• Time period data collection: January 1, 2014 to December 31, 2018.<br>• Primary care clinicians employed by UPMC were invited and encouraged to complete a web-based, validated, skin cancer training.<br>• Eligible patients presenting to a University of Pittsburgh Medical Center were offered a skin cancer screening.<br>• Each patient was assigned a screening status (screened or unscreened).<br>• Secondary analyses for all melanomas and for patients ≥ 65 y.
Sample characteristics	• N= 620,371<br>• Unscreened cohort:<br>– n = 450,948 (75.7%)<br>– mean age (SD): 56.5 (14.7)<br>– **n= 127,777 ≥65 years old (28.4%)**<br>• Screened cohort:<br>– n = 144 851 (24.3%)<br>– mean age (SD): 58.5 (12.9)<br>– **n= 47,603 ≥65 years old (32.9%)**
Key findings relevant for review question(s)	**1/3**<br>Total melanoma cases in screened and unscreened patients ≥ 65 years. Age-sex adjusted incidence [per 100 000 person-years] (95% CI):

	Unscreened	Screened
All melanomas	66.5 (58.7-75.3)	98.7 (83.2-117.2)
In situ	25.7 (21.0-31.3)	43.4 (33.5-56.4)
≤1 mm	22.3 (17.8-27.9)	36.1 (27.2-47.9)
>1 mm	18.5 (14.8-23.2)	19.3 (13.1-28.3)
>2 mm	14.1 (10.9-18.3)	12.0 (7.3-19.9)
>4 mm	7.6 (5.3-10.8)	3.4 (1.4-8.4)

	Interval* Melanomas in Screened and Unscreened Patients ≥ 65 years. Age-sex adjusted incidence [per 100 000 person-years] (95% CI):<br><table><tr><td></td><td>Unscreened</td><td>Screened</td></tr><tr><td>All melanomas</td><td>60.8 (53.3-69.3)</td><td>82.1 (68.0-99.1)</td></tr><tr><td>In situ</td><td>23.3 (18.9-28.7)</td><td>37.2 (28.1-49.2)</td></tr><tr><td>≤1 mm</td><td>20.3 (16.0-25.7)</td><td>28.7 (20.8-39.5)</td></tr><tr><td>>1 mm</td><td>17.2 (13.6-21.8)</td><td>16.2 (10.7-24.6)</td></tr><tr><td>>2 mm</td><td>13.5 (10.3-17.7)</td><td>9.0 (5.0-16.0)</td></tr><tr><td>>4 mm</td><td>7.0 (4.8-10.1)</td><td>2.2 (0.7-7.2)</td></tr></table><br>• For patients ≥65 years, the incidence of thin (≤ 1mm) and in situ melanoma (per 100 000 person-years) was higher in screened vs. unscreened patients.<br>• For patients ≥65 years, the incidence of thick melanoma (>2 mm) was lower in screened vs. unscreened patients.
Notes	*Defined as those diagnosed at least 60 days after the first screening visit/the first screen-eligible visit.

Author(s)	L. Navsaria; Y. Li; M. K. Nowakowska; C. Hinkston; L. Wheless; S. Giordano; M. Wehner
Title	LB911 Incidence and treatments of actinic keratosis in the Medicare population: A cohort study
Year of publication	2022[90]
Review Question (1/2/3)	1/3
Aim/purpose/objective	• To estimate the incidence rate of actinic keratosis (AK)<br>• To investigate the encounter characteristics (providers and treatments)
Design	Secondary data analysis
Population (Inclusion/exclusion Criteria)	Medicare* beneficiaries<br>Inclusion:<br>• Age ≥ 65 years
Methods	• Random selection of 4,999,999, de-identified Medicare beneficiaries aged ≥ 65 years<br>• Time period: 2009-2018<br>• Follow-up for a mean of 5.7 years (SD 3.6)<br>• Estimation of incidence rate of AK diagnosis encounter and identification of encounter characteristics (providers and treatments)
Sample characteristics	• n = 4,999,999
Key findings relevant for review question(s)	**1.**<br>• n= 1,462,985 (29.3%) with ≥ 1 AK diagnosis encounter were identified during the follow-up.<br>• Incidence rate of AK diagnosis encounters: 28,656 per 100,000 person-years (42,970 for men; 20,492 for women; 28,788 age-adjusted)<br>• “Most of the encounters were with dermatologists (78.6%), followed by physician assistants and nurse practitioners (10.8%), family physicians (4.5%).”<br>**3.**<br>• “The majority of encounters had a destruction treatment (81.5%), while a minority had a potential field treatment (topical medications [2.9%], photodynamic therapy [1.5%])“
Notes	*Medicare is a government national health insurance program in the United States.

<table>
<tr><td>Author(s)</td><td>Catharina C. van Niekerk, J. Hans D. M. Otten, Michelle M. van Rossum, Juul M. P. A. van den Reek, Erik Brummelkamp, Martijn Mol, J. Hans M. M. Groenewoud and Andre L. M. Verbeek</td></tr>
<tr><td>Titel</td><td>Trends in three major histological subtypes of cutaneous melanoma in the Netherlands between 1989 and 2016</td></tr>
<tr><td>Year of publication</td><td>2022[91]</td></tr>
<tr><td>Review Question (1/2/3)</td><td>1</td></tr>
<tr><td>Aim/purpose/objective</td><td>To describe the contribution of age, calendar period, and year of birth to trends in incidence regarding three major histological CM subtypes.</td></tr>
<tr><td>Design</td><td>Analyses of registry data</td></tr>
<tr><td>Population (Inclusion/exclusion Criteria)</td><td>Data from the Netherlands Cancer Registry and Statistics Netherlands</td></tr>
<tr><td>Methods</td><td>Identification of patients diagnosed with a primary cutaneous melanoma in the Netherlands
Including superficial spreading melanoma (SSM), lentigo maligna melanoma (LMM), nodular melanoma (NM)
Time period: 1989 to 2016
Number and rates of cases diagnosed were analyzed by age, calendar period, and birth cohort of people born in successive periods from 1925 to 1973.
Only first diagnosis included (if multiple in study period)</td></tr>
<tr><td>Sample characteristics</td><td><u>All</u>
Total, n = 118,588
Male, n = 52,000 (43.8%)
Female, n = 66,588 (56.2%)
The median age of diagnosis was 60 years</td></tr>
<tr><td>Key findings relevant for review question(s)</td><td><u>1.</u>
<u>Age and period-specific incidence rates of cutaneous melanoma per 100,000 person-years in the Netherlands between 1989 and 2016:</u>

<table>
<tr><td>Period/ age</td><td>60-64</td><td>65-69</td><td>70-74</td><td>75–79</td><td>80–84</td><td>85+</td></tr>
<tr><td colspan="7">Males</td></tr>
<tr><td>2014-2016</td><td>84.4</td><td>110.9</td><td>152.4</td><td>158,.5</td><td>157.7</td><td>159.7</td></tr>
<tr><td colspan="7">Females</td></tr>
<tr><td>2014-2016</td><td>79.6</td><td>100.6</td><td>123.6</td><td>108.7</td><td>101.2</td><td>81.7</td></tr>
</table></td></tr>
<tr><td>Notes</td><td>Netherlands Comprehensive Cancer Organization (IKNL) is responsible for Netherlands Cancer Registry: Data from Dutch nationwide pathology archive</td></tr>
</table>

Author(s)	C. Radkiewicz; J. Järkvik Krönmark; H. O. Adami; G. Edgren
Titel	Declining Cancer Incidence in the Elderly: Decreasing Diagnostic Intensity or Biology?
Year of publication	2022[92]
Review Question (1/2/3)	1
Aim/purpose/objective	• To delineate the cancer age-incidence pattern for a range of malignancies
Design	Analysis of registry data
Population (Inclusion/exclusion Criteria)	Swedish population<br>Inclusion:<br>• Age 20-99 years<br>• Diagnosis of one of the following cancers: pancreatic, lung, non-meningioma Brain, anorectal, urinary bladder, non–Hodgkin lymphoma, melanoma skin, breast, prostate
Methods	• Data extraction on nine common, adult cancers using the Swedish Cancer Register*<br>• Time period: 1970 to 2014<br>• Calculation of incidence and the proportion of autopsy-detected cancers by age<br>• Calculation of a projected cancer incidence by age (5-year age groups)<br>• Calculation of incidence rates (IR) per 100,000 person-years<br>• To contrast the reported and autopsy-detected cancer age incidence patterns:<br>➔ Calculation of proportion of performed autopsies where cancer was unexpectedly detected.<br>➔ Number of unexpected cancer findings in autopsy divided with autopsy counts in strata of age, calendar year<br>• “Total” incidence rate: Sum of the reported number of cancer cases and the “projected” number of incident autopsy cancers as the numerator.
Sample characteristics	• Total n= 1,020,397<br>• Melanoma skin cancer: n= 69,941 (6.9%)<br>• Age range: 20–99 years
Key findings relevant for review question(s)	• “there was a general pattern of declining incidence in high age in all cancers, except melanoma skin”<br>• Incidence rate increasing with age<br>• Peak at age 95 to 99: 64.8 /100,000 person-years
Notes	* “nationwide Swedish Cancer Register held by the Swedish National Board of Health and Welfare. Reporting is mandatory by law for both pathology and clinical departments, ensuring a high national coverage of approximately 95%”

<table>
<tr><td>Author(s)</td><td>A. S. Raghuwanshi; S. Diwan; H. Singh; K. C. Raghuwanshi</td></tr>
<tr><td>Titel</td><td>A Cross-Sectional Study to Assess the Psychosocial impact of Skin Diseases</td></tr>
<tr><td>Year of publication</td><td>2022[93]</td></tr>
<tr><td>Review Question (1/2/3)</td><td>1/2</td></tr>
<tr><td>Aim/purpose/objective</td><td>• To evaluate the psychosocial burden of skin disease among community- dwelling adults in Madhya Pradesh</td></tr>
<tr><td>Design</td><td>Cross-sectional</td></tr>
<tr><td>Population (Inclusion/exclusion Criteria)</td><td>Permanent residents of Madhya Pradesh, India
Inclusion:
• Age >21 years
• Lived in the chosen home for at least six months
• Capable of providing thoughtful responses to the survey questions</td></tr>
<tr><td>Methods</td><td>• Face-to-face interviews conducted by surveyor
• Question directly asking if any of the listed skin diseases have been experienced (list of 13 skin diseases)
• Outcomes: Depressive symptoms, Social isolation, Loneliness, Health-related quality of life
• Relationship between the 4 outcomes investgated via Multiple linear regression</td></tr>
<tr><td>Sample characteristics</td><td>• n = 1000</td></tr>
<tr><td>Key findings relevant for review question(s)</td><td>Distribution of individual skin diseases by gender and age group(%):
<table>
<tr><td rowspan="2">Skin disease</td><td colspan="2">Distribution by age groups (%)</td></tr>
<tr><td>60-74 years</td><td>≥ 75 years</td></tr>
<tr><td>Eczema</td><td>7.5</td><td>10.1</td></tr>
<tr><td>Acne</td><td>2.8</td><td>0.6</td></tr>
<tr><td>Psoriasis</td><td>0.7</td><td>2.7</td></tr>
<tr><td>Vitiligo</td><td>2</td><td>0.6</td></tr>
<tr><td>Viral warts</td><td>1.4</td><td>2.1</td></tr>
<tr><td>Scabies</td><td>0.3</td><td>0.6</td></tr>
<tr><td>Fungal skin infections</td><td>3.1</td><td>6.4</td></tr>
<tr><td>Chronic urticaria</td><td>2</td><td>2.2</td></tr>
<tr><td>Bacterial skin infections</td><td>9.7</td><td>14.9</td></tr>
<tr><td>Chronic ulcers</td><td>4.4</td><td>3.2</td></tr>
<tr><td>Skin cancer</td><td>-</td><td>1.8</td></tr>
<tr><td>Alopecia areata</td><td>-</td><td>0.6</td></tr>
<tr><td>Unspecific symptom of Pruritus (RQ2)</td><td>15.2</td><td>19.2</td></tr>
</table></td></tr>
<tr><td>Notes</td><td>• Association with depressive symptoms not reported by age group</td></tr>
</table>

Author(s)	J. D. Rodriguez-Betancourt; N. Arias-Ortiz
Title	Cutaneous melanoma incidence, mortality, and survival in Manizales, Colombia: a population-based study
Year of publication	2022[94]
Review Question (1/2/3)	1/2
Aim/purpose/objective	• To estimate the cutaneous melanoma (CM) incidence, mortality, and survival in Manizales, Colombia
Design	Analysis of registry data
Population (Inclusion/exclusion Criteria)	Data of the Manizales Cancer Registry*
Methods	• Time period analysed: 01.01.2006 to 31.12.2015<br>• Passive follow-up was performed for 60 months or until 30 November 2020 to identify the event (death owing to melanoma) and the time-to-event.<br>• Calculation of incidence rates and mortality rates by sex and age per 100,000 person-years. Age-standardisation using Segi's world standard population as reference.<br>• Age-standardized rates were corrected for cases with unknown age (IARC)<br>• To calculate the denominators (population at risk), the Manizales age- and sex-specific population per year were obtained from DANE population estimates according to the 2018 census<br>• Calculation of cause-specific survival using the Kaplan-Meier method
Sample characteristics	• n = 42 ≥70 years<br>• Mean age at diagnosis: 60 years (SD 17.8)
Key findings relevant for review question(s)	**1.**<br>**Incidence of cutaneous melanoma**<br>Incident cases ≥70 years, n (%)<br>• Male: 16 (29.6)<br>• Female: 26 (33.3)<br>• Total: 42 (31.8)<br>**2.**<br>**Cause-specific survival estimates of cutaneous melanoma**<br>Age group ≥70 years<br>Deaths: n = 18<br>2-year survival (95% CI): 64.6 (47.6–77.3)<br>5-year survival (95% CI): 56.4 (39.4–70.3)<br><br>HR 2.07 (univariate)<br>HR 3.37 to 3.47 (multivariate)
Notes	• "The MCR includes all primary malignant tumors in all locations identified through active collection of data on patient and tumor variables from health care providers and pathology laboratories according to IARC rules."<br>• In total incident cases ≥70 years are n = 42 (Table 1) but in Table 3 the total n = 41 in cases ≥70 years.

Author(s)	Huang J, Zhang L, Shi L, Wu M, Lv T, Zhang Y, Lai Y, Tu Q, Wang X, Wang H.
Title	An epidemiological study on skin tumors of the elderly in a community in Shanghai, China.
Year of publication	2023[95]
Review Question (1/2/3)	• To assess the prevalence of skin tumours and associated factors.
Aim/purpose/objective	1
Design	Cross-sectional
Population (Inclusion/exclusion Criteria)	Permanent residents in a community in Shanghai, China<br>Inclusion:<br>• Residency for at least 6 months<br>• Community setting (excluding hospitalized patients and residents in nursing homes)<br>• Age: >60 years<br>• Written informed consent
Method	• Questionnaire survey from May 1, 2011 to November 30, 2011 involving basic information, categories of SC, risk factors and concomitant diseases.<br>• Dermatological examination and medical history by board certified dermatologists.<br>• Diagnoses identified as skin cancer (based on ICD-10): malignant melanoma, squamous cell carcinoma (SCC), basal cell cancinoma, actinic keratosis, keratoacanthoma and cutaneous horn with malignant cells.<br>• Rest of diagnoses identified as benign skin tumours (seborrheic keratoses, skin tags).
Sample characteristics	• n = 2038<br>→ n= 524 (25.7%) from 60 to 64 years old<br>→ n= 291 (14.3%) from 65 to 69 years old<br>→ n= 309 (15.2%) from 70 to 74 years old<br>→ n= 291 (14.3%) from 75 to 79 years old<br>→ n= 297 (14.6%) from 80 to 84 years old<br>→ n= 106 (5.2%) over ≥ 85 years old<br>• Average age: 72.05 ± 8.17 years<br>• 62.4% female

Key findings relevant for review question(s)	<table><tr><th>Age group</th><th>Prevalence, n [% (95% CI)]</th></tr><tr><td>60–64</td><td>8 (1.5, 0.5–2.6)</td></tr><tr><td>65–69</td><td>7 (2.4, 0.6–4.2)</td></tr><tr><td>70–74</td><td>7 (2.3, 0.6–3.9)</td></tr><tr><td>75–79</td><td>30 (5.9, 3.8–7.9)</td></tr><tr><td>80–84</td><td>15 (5.1, 2.5–7.6)</td></tr><tr><td>≥ 85</td><td>11 (10.4, 4.5–16.3)</td></tr></table><br>Standardised prevalence rates of Skin cancer per 100,000 by age group (shanghai older people population as standard):<br>• 60-69 year olds: 954.8<br>• 70-79 year olds: 1355.1<br>• 80+ year olds: 1167.3<br><br>Prevalence of various cutaneous malignancies in different age groups. AK Actinic keratosis, SCC Squamous cell carcinoma, BCC Basal cell carcinoma, BD Bowen’s disease, KA Keratoacanthoma, CH Cutaneous horn:<table><tr><th rowspan="2">Age (years)</th><th colspan="6">Prevalence, n (%)</th></tr><tr><th>AK</th><th>SCC</th><th>BCC</th><th>BD</th><th>KA</th><th>CH</th></tr><tr><td>60–64</td><td>5 (0.9)</td><td>0</td><td>0</td><td>0</td><td>0</td><td>0</td></tr><tr><td>65–69</td><td>5 (1.7)</td><td>0</td><td>1(0.3)</td><td>0</td><td>0</td><td>0</td></tr><tr><td>70–74</td><td>6 (1.9)</td><td>1 (0.3)</td><td>2 (0.6)</td><td>0</td><td>0</td><td>0</td></tr><tr><td>75–79</td><td>26 (5.1)</td><td>1 (0.2)</td><td>4 (0.8)</td><td>0</td><td>0</td><td>0</td></tr><tr><td>80–84</td><td>13 (4.4)</td><td>1 (0.3)</td><td>2 (0.7)</td><td>1 (0.3)</td><td>1 (0.3)</td><td>0</td></tr><tr><td>≥ 85</td><td>8 (7.5)</td><td>0</td><td>0</td><td>0</td><td>0</td><td>1 (0.9)</td></tr><tr><td>Sum</td><td>63 (3.1)</td><td>3 (0.1)</td><td>9 (0.4)</td><td>1 (0.0)</td><td>1 (0.0)</td><td>1 (0.0)</td></tr></table>
Notes	-

Author(s)	Keim, U.; Katalinic, A.; Holleczek, B.; Wakkee, M.; Garbe, C.; Leiter, U.
Titel	Incidence, mortality and trends of cutaneous squamous cell carcinoma in Germany, the Netherlands, and Scotland
Year of publication	2023[96]
Review Question (1/2/3)	1
Aim/purpose/objective	• Analyse incidence rates of cSCC over three decades with an extrapolation to 2040
Design	Registry data
Population (Inclusion/Exclusion Criteria)	Cases in cancer registry of of German federal states Schleswig-Holstein and Saarland, the Netherlands and Scotland
Methods	• Incident cases of cSCC (ICD-10: C44, ICD-O codes: 8050-8084)<br>→ 1989/1990/1993/1999 to 2018/2020/2021Only information on the first tumour of cSCC in a patient was used in the analyses<br>• Age-standardised rates of incidence (ASIR) and mortality (ASMR) per 100,000 persons per year are based on the New European Standard Population from 2013<br>• Age-specific incidence rates by sex and calculated for four different age groups (<40 years, 40-59 years, 60-79 years and 80+ years)<br>• estimates of the annual percentage changes (EAPC) over time<br>• Additionally, the average annual percentage change (AAPC)<br>• Age-standardised mortality rates (ASMR)
Sample characteristics	• No data
Key findings relevant for review questions	• Incidence rates of cSCC increase with age, highest in oldest age group (80+ years)<br><br>Saarland:<br>→ Males (80+):847/100,000 persons per year<br>→ Females (80+):285/100,000 persons per year<br>→ 2019<br>→ Incidence rates highest in age groups 60+<br>Schleswig-Holstein:<br>→ Males (80+):600 /100,000 persons per year<br>→ Females (80+): 278 /100,000 persons per year<br>→ 2020<br>Netherlands:<br>→ Males (80+): 966 /100,000 persons per year<br>→ Females (80+): 560//100,000 persons per year<br>→ 2021<br>Scotland:<br>→ Males (80+):1107/100,000 persons per year<br>→ Females (80+):346/100,000 persons per year<br>→ 2017<br><br>Saarland:<br>→ Males 80+: Rate increased from 163 in 1990 to 847 in 2019

	→ Females 80+: Rate increased from 127 to 285<br>Schleswig-Holstein:<br>→ Strongest increases in age group 60+<br>→ Males 80+: Rate increased from 308 in 1999 to 600 in 2020<br>→ Females 80+: Rate increased from 173 to 278<br>Netherlands:<br>→ Steepest increase of rates in age groups 60+<br>→ Males 80+: Rate increased from 267 in 1989 to 966 in 2021<br>→ Females 80+: Rate increased from 100 to around 560<br>Scotland:<br>→ Continous increase in all age groups 60+<br>→ Males 80+: Rates increased from 361 in 1993 to 1107 in 2017<br>→ Females 80+: Rates increased from 179 to 346
Notes	-

Author(s)	Qingqiang Xu, Xiaoyan Wang, Yan Bai , Yan Zheng, Junbo Duan, Jianqiang Du and Xiaoming Wu
Titel	Trends of non-melanoma skin cancer incidence in Hong Kong and projection up to 2030 based on changing demographics
Year of publication	2023[97]
Review Question (1/2/3)	1
Aim/purpose/objective	• To assess the trends in non-melanoma skin cancer (NMSC) incidence in Hong Kong from 1990 to 2019 and the assocication of age, calendar period, and birth cohort.<br>• To make projections to 2030.<br>• To examine the drivers of NMSC incidence.
Design	Analyses of registry data
Population (Inclusion/exclusion Criteria)	Data of Hong Kong Cancer Registry (HKCaR)<br>Inclusion:<br>• Age ≥ 20 years
Methods	• Time period analysed: 1990 to 2019<br>• ICD-9 code 173 or the ICD-10 code C44<br>• Hong Kong population estimates from the Union Nations World Population Prospects 2019 Revision<br>• Age-standardised incidence rates<br>• Age-period-cohort (APC) analysis<br>• Projection of future incidence rates
Sample characteristics	• n = 19,568 (9812 male patients [50.14%]) cases<br>• No data on number in age groups and mean age
Key findings relevant for review question(s)	• Fitted age-specific rates in reference cohort adjusted for period effects<br>→ Females: app. 20 (60 years) to 310 (90 years)<br>→ Males: app. 40 (60 years) to 400 (90 years)
Notes	• HKCaR “is maintained according to the International Agency for Research on Cancer (IARC) of the World Health Organization.”<br>• “Rates” are extracted from Figure 3<br>• Projections: Continues to increase, especially for people 60 years and older

REFERENCES

1. Akbari ME, Rafiee M, Khoei MA et al. Incidence and survival of cancers in the elderly population in Iran: 2001-2005. *Asian Pac J Cancer Prev* 2011; **12**:3035-9.
2. Augustin M, Herberger K, Hintzen S et al. Prevalence of skin lesions and need for treatment in a cohort of 90 880 workers. *Br J Dermatol* 2011; **165**:865-73.
3. Frese T, Herrmann K, Sandholzer H. Pruritus as reason for encounter in general practice. *J Clin Med Res* 2011; **3**:223-9.
4. Paul C, Maumus-Robert S, Mazereeuw-Hautier J et al. Prevalence and risk factors for xerosis in the elderly: a cross-sectional epidemiological study in primary care. *Dermatology* 2011; **223**:260-5.
5. Ritchie SR, Fraser JD, Libby E et al. Demographic variation in community-based MRSA skin and soft tissue infection in Auckland, New Zealand. *New Zealand Medical Journal* 2011; **124**.
6. Wu J, Guo Z, Berman R et al. P2-119: Occurrence of nonmelanoma skin cancer in the elderly with and without Alzheimer's disease in the US [abstract]. In: Alzheimer's Association International Conference, AAIC 11, Paris, France. *Alzheimer's & Dementia* 2011; 7:S347. doi: 10.1016/j.jalz.2011.05.1007. Available from: https://alz-journals.onlinelibrary.wiley.com/doi/10.1016/j.jalz.2011.05.1007. [accessed 20.04.2023].
7. Wu J, Guo Z, Berman R et al. 698. Risk of Non-Melanoma Skin Cancer in Elderly Patients with Alzheimer's Disease [abstract]. In: 27th International Conference on Pharmacoepidemiology and Therapeutic Risk Management, Chicago, United States. *Pharmacoepidemiology and Drug Safety* 2011; 20:S303-4. doi: 10.1002/pds.2206. Available from: https://onlinelibrary.wiley.com/doi/10.1002/pds.2206. [accessed 02.05.2023].
8. Hollestein LM, van den Akker SA, Nijsten T et al. Trends of cutaneous melanoma in The Netherlands: increasing incidence rates among all Breslow thickness categories and rising mortality rates since 1989. *Ann Oncol* 2012; **23**:524-30.
9. Joly P, Baricault S, Sparsa A et al. Incidence and mortality of bullous pemphigoid in France. *J Invest Dermatol* 2012; **132**:1998-2004.
10. Bonaccorsi G, Lorini C, Santomauro F et al. 202 Impact of different pads in elderly assisted in home care [abstract]. In: 43rd Annual Meeting of the International Continence Society, ICS 2013, Barcelona, Spain. *Neurourol. Urodyn.* 2013:802-3. doi: 10.1002/nau.22472. Available from: https://onlinelibrary.wiley.com/doi/10.1002/nau.22472. [accessed 02.05.2023].
11. Danielsen K, Olsen AO, Wilsgaard T, Furberg AS. Is the prevalence of psoriasis increasing? A 30-year follow-up of a population-based cohort. *Br J Dermatol* 2013; **168**:1303-10.
12. Etzkorn JR, Parikh RP, Marzban SS et al. Identifying risk factors using a skin cancer screening program. *Cancer Control* 2013; **20**:248-54.
13. Flohil SC, van der Leest RJ, Dowlatshahi EA et al. Prevalence of actinic keratosis and its risk factors in the general population: the Rotterdam Study. *J Invest Dermatol* 2013; **133**:1971-8.
14. Okuno Y, Takao Y, Miyazaki Y et al. Assessment of skin test with varicella-zoster virus antigen for predicting the risk of herpes zoster. *Epidemiol Infect* 2013; **141**:706-13.
15. Robsahm TE, Bergva G, Hestvik UE, Moller B. Sex differences in rising trends of cutaneous malignant melanoma in Norway, 1954-2008. *Melanoma Res* 2013; **23**:70-8.
16. Wysong A, Linos E, Hernandez-Boussard T et al. Nonmelanoma skin cancer visits and procedure patterns in a nationally representative sample: national ambulatory medical care survey 1995-2007. *Dermatol Surg* 2013; **39**:596-602.
17. Gontijo Guerra S, Vasiliadis HM, Preville M, Berbiche D. Skin conditions in community-living older adults: prevalence and characteristics of medical care service use. *J Cutan Med Surg* 2014; **18**:186-94.
18. Gontijo Guerra S, Preville M, Vasiliadis HM, Berbiche D. Association between skin conditions and depressive disorders in community-dwelling older adults. *J Cutan Med Surg* 2014; **18**:256-64.
19. Hsieh C-F, Huang W-F, Chiang Y-T. 157. The Incidence of Actinic Keratosis and Risk of Non-Melanoma Skin Cancer in Taiwan [abstract]. In: 30th International Conference on Pharmacoepidemiology and Therapeutic Risk Management, Taipei, Taiwan. *Pharmacoepidemiol.*

Drug Saf. 2014; 23(S1):84-5. doi: 10.1002/pds.3701. Available from: https://onlinelibrary.wiley.com/doi/10.1002/pds.3701. [accessed 20.04.2023].
20. Landis ET, Davis SA, Taheri A, Feldman SR. Top dermatologic diagnoses by age. *Dermatol Online J* 2014; **20**:22368.
21. Caretti KL, Mehregan DR, Mehregan DA. A survey of self-reported skin disease in the elderly African-American population. *Int J Dermatol* 2015; **54**:1034-8.
22. Cybulski M, Krajewska-Kulak E. Skin diseases among elderly inhabitants of Bialystok, Poland. *Clin Interv Aging* 2015; **10**:1937-43.
23. Duim E, Sa FH, Duarte YA et al. Prevalence and characteristics of lesions in elderly people living in the community. *Rev Esc Enferm USP* 2015; **49 Spec No**:51-7.
24. Hay RJ, Fuller LC. Global burden of skin disease in the elderly: a grand challenge to skin health. *G Ital Dermatol Venereol* 2015; **150**:693-8.
25. Kiiski V, Susitaival P, Remitz A, Reitamo S. 086 Is atopic dermatitis more persistent than previously estimated? [abstract]. In: 45th Annual Meeting of the European Society for Dermatological Research, Rotterdam, Netherlands. *Journal of Investigative Dermatology* 2015; 135:S15. doi: 10.1038/jid.2015.266. Available from: https://www.sciencedirect.com/science/article/pii/S0022202X15601993?via%3Dihub. [accessed 02.05.2023].
26. Romani L, Steer AC, Whitfeld MJ, Kaldor JM. Prevalence of scabies and impetigo worldwide: a systematic review. *Lancet Infect Dis* 2015; **15**:960-7.
27. Cinotti E, Perrot JL, Labeille B et al. Skin tumours and skin aging in 209 French elderly people: the PROOF study. *Eur J Dermatol* 2016; **26**:470-6.
28. Hsieh C-F, Chiang Y-T, Chiu H-Y, Huang W-F. A Nationwide Cohort Study of Actinic Keratosis in Taiwan*. *International Journal of Gerontology* 2016; **10**:218-22.
29. Liu T, Brienza R. What brings an older veteran to an urgent visit (UV) A review of the chief concerns by veterans aged 65 and older who presented for uv durin a 6-month period at the West Haven Veteran Affairs Center of excellence in Primary Care Education (VA COEPCE), an interprofessional academic patient aligned care team (PACT) [abstract]. In: Abstracts from the 2016 Society of General Internal Medicine Annual Meeting. *J Gen Intern Med* 2016; 31(Suppl 2):S468-9. doi: 10.1007/s11606-016-3657-7. Available from: https://link.springer.com/article/10.1007/s11606-016-3657-7. [accessed 02.05.2023].
30. Trautmann F, Meier F, Seidler A, Schmitt J. Effects of the German skin cancer screening programme on melanoma incidence and indicators of disease severity. *Br J Dermatol* 2016; **175**:912-9.
31. Tseng HW, Shiue YL, Tsai KW et al. Risk of skin cancer in patients with diabetes mellitus: A nationwide retrospective cohort study in Taiwan. *Medicine (Baltimore)* 2016; **95**:e4070.
32. Alexandridou M, Bollaerts K. Pin10 Zoster Vaccine Effectiveness against incident herpes zoster and post-herpetic neuralgia in elderly in the UK [abstract]. In: ISPOR 20th Annual European Congress, Glasgow, United Kingdom. *Value Health* 2017; 20:A780. doi: 10.1016/j.jval.2017.08.2263. Available from: https://www.sciencedirect.com/science/article/pii/S1098301517325974?via%3Dihub. [accessed 26.04.2023].
33. Asokan N, Binesh VG. Cutaneous problems in elderly diabetics: A population-based comparative cross-sectional survey. *Indian J Dermatol Venereol Leprol* 2017; **83**:205-11.
34. Barbaric J, Laversanne M, Znaor A. Malignant melanoma incidence trends in a Mediterranean population following socioeconomic transition and war: results of age-period-cohort analysis in Croatia, 1989-2013. *Melanoma Res* 2017; **27**:498-502.
35. George LS, Deshpande S, Krishna Kumar MK, Patil RS. Morbidity pattern and its sociodemographic determinants among elderly population of Raichur district, Karnataka, India. *J Family Med Prim Care* 2017; **6**:340-4.
36. Hahnel E, Lichterfeld A, Blume-Peytavi U, Kottner J. The epidemiology of skin conditions in the aged: A systematic review. *J Tissue Viability* 2017; **26**:20-8.

37. Henchoz Y, Bula C, Guessous I et al. Chronic symptoms in a representative sample of community-dwelling older people: a cross-sectional study in Switzerland. *BMJ Open* 2017; **7**:e014485.
38. Iizaka S, Nagata S, Sanada H. Nutritional Status and Habitual Dietary Intake Are Associated with Frail Skin Conditions in Community-Dwelling Older People. *J Nutr Health Aging* 2017; **21**:137-46.
39. Karimkhani C, Colombara DV, Drucker AM et al. The global burden of scabies: a cross-sectional analysis from the Global Burden of Disease Study 2015. *Lancet Infect Dis* 2017; **17**:1247-54.
40. Karimkhani C, Dellavalle RP, Coffeng LE et al. Global Skin Disease Morbidity and Mortality: An Update From the Global Burden of Disease Study 2013. *JAMA Dermatol* 2017; **153**:406-12.
41. Kim J, Choi Y, Shin J et al. Incidence of Pressure Ulcers During Home and Institutional Care Among Long-Term Care Insurance Beneficiaries With Dementia Using the Korean Elderly Cohort. *J Am Med Dir Assoc* 2017; **18**:638 e1- e5.
42. Lee HJ, Ju YJ, Park EC et al. Effects of home-visit nursing services on hospitalization in the elderly with pressure ulcers: a longitudinal study. *Eur J Public Health* 2017; **27**:822-6.
43. Pandeya N, Olsen CM, Whiteman DC. The incidence and multiplicity rates of keratinocyte cancers in Australia. *Med J Aust* 2017; **207**:339-43.
44. Thorslund K, Seifert O, Nilzen K, Gronhagen C. Incidence of bullous pemphigoid in Sweden 2005-2012: a nationwide population-based cohort study of 3761 patients. *Arch Dermatol Res* 2017; **309**:721-7.
45. Abuabara K, Magyari A, Margolis DJ, Langan M. The prevalence of atopic eczema across the lifespan: a U.K. population-based cohort study [abstract]. In: Abstracts of the 10th George Rajka International Symposium on Atopic Dermatitis, Utrecht, Netherlands. *British Journal of Dermatology* 2018; 179:e58. doi: 10.1111/bjd.16718. Available from: https://academic.oup.com/bjd/article/179/1/e5/6732334?login=true. [accessed 20.04.2023].
46. Aitken JF, Youlden DR, Baade PD et al. Generational shift in melanoma incidence and mortality in Queensland, Australia, 1995-2014. *Int J Cancer* 2018; **142**:1528-35.
47. Cowdell F, Dyson J, Long J, Macleod U. Self-reported skin concerns: An epidemiological study of community-dwelling older people. *Int J Older People Nurs* 2018; **13**:e12195.
48. Drewitz KP, Stark K, Zimmermann ME et al. P102 | Prevalence and determinants of Psoriasis in a cross- sectional study of the elderly—results from the German AugUR study [abstract]. In: 45th Annual Meeting of the Arbeitsgemeinschaft Dermatologische Forschung, ADF 2018, Zurich, Switzerland. *Experimental Dermatology* 2018; 27:e43-4. doi: 10.1111/exd.13486. Available from: https://onlinelibrary.wiley.com/doi/10.1111/exd.13486. [accessed 20.04.2023].
49. Dziunycz PJ, Schuller E, Hofbauer GFL. Prevalence of Actinic Keratosis in Patients Attending General Practitioners in Switzerland. *Dermatology* 2018; **234**:214-9.
50. Hu L, Jin S, Chen L, Wang Y. Trends in the incidence and mortality of cutaneous melanoma in Hong Kong between 1983 and 2015. *Int J Clin Exp Med* 2018; **11**:8259-66.
51. Lichterfeld-Kottner A, Lahmann N, Blume-Peytavi U et al. Dry skin in home care: A representative prevalence study. *J Tissue Viability* 2018; **27**:226-31.
52. Sanders MGH, Pardo LM, Franco OH et al. Prevalence and determinants of seborrhoeic dermatitis in a middle-aged and elderly population: the Rotterdam Study. *Br J Dermatol* 2018; **178**:148-53.
53. Steglich RB, Coelho K, Cardoso S et al. Epidemiological and histopathological aspects of primary cutaneous melanoma in residents of Joinville, 2003-2014. *An Bras Dermatol* 2018; **93**:45-53.
54. Augustin M, Kirsten N, Korber A et al. Prevalence, predictors and comorbidity of dry skin in the general population. *J Eur Acad Dermatol Venereol* 2019; **33**:147-50.
55. Augustin M, Kirsten N, Koerber A et al. Epidemiology of dry skin in the general population [abstract]. In: 24th World Congress of Dermatology, Milan, Italy. 2019. Available from: https://www.wcd2019milan-dl.org/abstract-book/documents/abstracts/12-epidemiology/epidemiology-of-dry-skin-in-2502.pdf. [accessed 27.04.2023].
56. Drewitz KP, Stark K, Zimmermann ME et al. P086 | Frequency and comorbidities of eczema in an elderly population in Germany: results from augur [abstract]. In: 46th Annual Meeting of the

Arbeitsgemeinschaft Dermatologische Forschung, ADF, Munich, Germany. *Experimental Dermatology* 2019; 28:e41-2. doi: 10.1111/exd.13859. Available from: https://onlinelibrary.wiley.com/doi/10.1111/exd.13859. [accessed 20.04.2023].
57. Mekic S, Jacobs LC, Gunn DA et al. Prevalence and determinants for xerosis cutis in the middle-aged and elderly population: A cross-sectional study. *J Am Acad Dermatol* 2019; **81**:963-9 e2.
58. Meyers JL, Candrilli SD, Rausch DA et al. Costs of herpes zoster complications in older adults: A cohort study of US claims database. *Vaccine* 2019; **37**:1235-44.
59. Sari SP, Everink IH, Sari EA et al. The prevalence of pressure ulcers in community-dwelling older adults: A study in an Indonesian city. *Int Wound J* 2019; **16**:534-41.
60. Silva ESD, Dumith SC. Non-use of sunscreen among adults and the elderly in southern Brazil. *An Bras Dermatol* 2019; **94**:567-73.
61. Tizek L, Schielein MC, Seifert F et al. Skin diseases are more common than we think: screening results of an unreferred population at the Munich Oktoberfest. *J Eur Acad Dermatol Venereol* 2019; **33**:1421-8.
62. Venables ZC, Nijsten T, Wong KF et al. Epidemiology of basal and cutaneous squamous cell carcinoma in the U.K. 2013-15: a cohort study. *Br J Dermatol* 2019; **181**:474-82.
63. Venables ZC, Autier P, Nijsten T et al. Nationwide Incidence of Metastatic Cutaneous Squamous Cell Carcinoma in England. *JAMA Dermatol* 2019; **155**:298-306.
64. Bianchi M, Santos A, Cordioli E. Benefits of Teledermatology for Geriatric Patients: Population-Based Cross-Sectional Study. *J Med Internet Res* 2020; **22**:e16700.
65. Chang C-Y, Park H, Lo-Ciganic J. 3946 | The prevalence of sun protective behaviors across different age groups in the US population: Findings from the 2015 US Health Interview Survey [abstract]. In: Special Issue: Abstracts of the 36th International Conference on Pharmacoepidemiology & Therapeutic Risk Management, Virtual. *Pharmacoepidemiol Drug Saf* 2020; 29(Suppl 3):306-7. doi: 10.1002/pds.5114. Available from: https://onlinelibrary.wiley.com/doi/10.1002/pds.5114. [accessed 02.05.2023].
66. Everink IHJ, Kottner J, van Haastregt JCM et al. Skin areas, clinical severity, duration and risk factors of intertrigo: A secondary data analysis. *J Tissue Viability* 2020; **30**:102-7.
67. Fors M, Gonzalez P, Viada C et al. Actinic keratoses in subjects from la Mitad del Mundo, Ecuador. *BMC Dermatol* 2020; **20**:11.
68. Kottner J, Everink I, van Haastregt J et al. Prevalence of intertrigo and associated factors: A secondary data analysis of four annual multicentre prevalence studies in the Netherlands. *Int J Nurs Stud* 2020; **104**:103437.
69. Prasad S, Hussain N, Sharma S et al. Impact of Pressure Injury Prevention Protocol in Home Care Services on the Prevalence of Pressure Injuries in the Dubai Community. *Dubai Medical Journal* 2020; **3**:99-104.
70. Sideris E, Thomas SJ. Patients' sun practices, perceptions of skin cancer and their risk of skin cancer in rural Australia. *Health Promot J Austr* 2020; **31**:84-92.
71. Sinikumpu SP, Jokelainen J, Haarala AK et al. The High Prevalence of Skin Diseases in Adults Aged 70 and Older. *J Am Geriatr Soc* 2020; **68**:2565-71.
72. Tokez S, Wakkee M, Louwman M et al. Assessment of Cutaneous Squamous Cell Carcinoma (cSCC) In situ Incidence and the Risk of Developing Invasive cSCC in Patients With Prior cSCC In situ vs the General Population in the Netherlands, 1989-2017. *JAMA Dermatol* 2020; **156**:973-81.
73. Tseng HF, Bruxvoort K, Ackerson B et al. The Epidemiology of Herpes Zoster in Immunocompetent, Unvaccinated Adults >/=50 Years Old: Incidence, Complications, Hospitalization, Mortality, and Recurrence. *J Infect Dis* 2020; **222**:798-806.
74. Yew YW, Kuan AHY, Ge L et al. Psychosocial impact of skin diseases: A population-based study. *PLoS One* 2020; **15**:e0244765.
75. Yong SS, Kwan Z, Ch'ng CC et al. Self-reported generalised pruritus among community-dwelling older adults in Malaysia. *BMC Geriatr* 2020; **20**:223.
76. Bai R, Huang H, Li M, Chu M. Temporal Trends in the Incidence and Mortality of Skin Malignant Melanoma in China from 1990 to 2019. *J Oncol* 2021; **2021**:9989824.

77. Barbieri JS, Rodriguez O, Rosenbach M, Margolis D. Incidence and Prevalence of Granuloma Annulare in the United States. *JAMA Dermatol* 2021; **157**:824-30.
78. Bucchi L, Mancini S, Crocetti E et al. Mid-term trends and recent birth-cohort-dependent changes in incidence rates of cutaneous malignant melanoma in Italy. *Int J Cancer* 2021; **148**:835-44.
79. Drewitz KP, Stark KJ, Zimmermann ME et al. Frequency of hand eczema in the elderly: Cross-sectional findings from the German AugUR study. *Contact Dermatitis* 2021; **85**:489-93.
80. Madani S, Marwaha S, Dusendang JR et al. Ten-Year Follow-up of Persons With Sun-Damaged Skin Associated With Subsequent Development of Cutaneous Squamous Cell Carcinoma. *JAMA Dermatol* 2021; **157**:559-65.
81. Memon A, Bannister P, Rogers I et al. Changing epidemiology and age-specific incidence of cutaneous malignant melanoma in England: An analysis of the national cancer registration data by age, gender and anatomical site, 1981-2018. *Lancet Reg Health Eur* 2021; **2**:100024.
82. Neena V, Asokan N, Jose R, Sarin A. Prevalence of eczema among older persons: A population-based cross-sectional study. *Indian J Dermatol Venereol Leprol* 2021; **89**:426-30.
83. Tang L, Li F, Xu F et al. Prevalence of vitiligo and associated comorbidities in adults in Shanghai, China: a community-based, cross-sectional survey. *Ann Palliat Med* 2021; **10**:8103-11.
84. Waldmann A, Pritzkuleit R, Labohm L, Katalinic A. Epidemiologie von Krebs im hohen Lebensalter. *best practice onkologie* 2021; **16**:586-97.
85. Blazek K, Furestad E, Ryan D et al. The impact of skin cancer prevention efforts in New South Wales, Australia: Generational trends in melanoma incidence and mortality. *Cancer Epidemiol* 2022; **81**:102263.
86. Botvid SHC, Storgaard Hove L, Backe MB et al. Low prevalence of patients diagnosed with psoriasis in Nuuk: a call for increased awareness of chronic skin disease in Greenland. *Int J Circumpolar Health* 2022; **81**:2068111.
87. Choon SE, Wright AK, Griffiths CEM et al. Incidence and prevalence of psoriasis in multiethnic Johor Bahru, Malaysia: a population-based cohort study using electronic health data routinely captured in the Teleprimary Care (TPC(R)) clinical information system from 2010 to 2020: Classification: Epidemiology. *Br J Dermatol* 2022; **187**:713-21.
88. Lu L, Chen L, Xu Y, Liu A. Global incidence and prevalence of bullous pemphigoid: A systematic review and meta-analysis. *J Cosmet Dermatol* 2022; **21**:4818-35.
89. Matsumoto M, Wack S, Weinstock MA et al. Five-Year Outcomes of a Melanoma Screening Initiative in a Large Health Care System. *JAMA Dermatol* 2022; **158**:504-12.
90. Navsaria L, Li Y, Nowakowska M et al. LB911 Incidence and treatments of actinic keratosis in the Medicare population: A cohort study [abstract]. In: Society for Investigative Dermatology (SID) 2022 Meeting, Portland, United States. *Journal of Investigative Dermatology* 2022; 142:B10. doi: 10.1016/j.jid.2022.05.929. Available from: https://www.sciencedirect.com/science/article/pii/S0022202X22013707?via%3Dihub. [accessed 20.04.2023].
91. van Niekerk CC, Otten J, van Rossum MM et al. Trends in three major histological subtypes of cutaneous melanoma in the Netherlands between 1989 and 2016. *Int J Dermatol* 2023; **62**:508-13.
92. Radkiewicz C, Jarkvik Kronmark J, Adami HO, Edgren G. Declining Cancer Incidence in the Elderly: Decreasing Diagnostic Intensity or Biology? *Cancer Epidemiol Biomarkers Prev* 2022; **31**:280-6.
93. Raghuwanshi AS, Diwan S, Singh H, Raghuwanshi KC. A Cross-Sectional Study to Assess the Psychosocial impact of Skin Diseases. *International Journal of Pharmaceutical and Clinical Research* 2022; **14**:1061-7.
94. Rodriguez-Betancourt JD, Arias-Ortiz N. Cutaneous melanoma incidence, mortality, and survival in Manizales, Colombia: a population-based study. *J Int Med Res* 2022; **50**:3000605221106706.
95. Huang J, Zhang L, Shi L et al. An epidemiological study on skin tumors of the elderly in a community in Shanghai, China. *Sci Rep* 2023; **13**:4441.

96. Keim U, Katalinic A, Holleczek B et al. Incidence, mortality and trends of cutaneous squamous cell carcinoma in Germany, the Netherlands, and Scotland. *Eur J Cancer* 2023; **183**:60-8.
97. Xu Q, Wang X, Bai Y et al. Trends of non-melanoma skin cancer incidence in Hong Kong and projection up to 2030 based on changing demographics. *Ann Med* 2023; **55**:146-54.
