# Supplementary material for: Skin health of community-living older people: a scoping review
Source: Arch Dermatol Res. 2024 Jun 1;316(6):319. doi: 10.1007/s00403-024-03059-0 (PMC11144137; doi:10.1007/s00403-024-03059-0)
Supplement: Supplementary file 3 — Supplementary Material 3 [file 403_2024_3059_MOESM3_ESM.pdf]

Supplementary material 3

Article title: Skin health of community-living older people: a scoping review

Authors: Jan Kottner[1], Alexandra Fastner[1], Dimitra-Aikaterini Lintzeri[2], Ulrike Blume-Peytavi[2], Christopher E. M. Griffiths[3,4]

Institutions

[1]Institute of Clinical Nursing Science, Charité Universitätsmedizin Berlin, Berlin, Germany.

[2]Department of Dermatology, Venerology and Allergology, Charité Universitätsmedizin Berlin, Berlin, Germany.

[3]Department of Dermatology, King's College Hospital, King's College London, London, UK.

[4]Centre for Dermatology Research, NIHR Manchester Biomedical Research Centre, The University of Manchester, Manchester, UK.

Corresponding author Jan Kottner

Charité – Universitätsmedizin Berlin,

Institute of Clinical Nursing Science,

Chariteplatz 1,

10117 Berlin, Germany

Email: jan.kottner@charite.de

Table S3: Included citations.

No.	Author (year)	Evidence			Design		Region
		Epidemiol-ogy	Burden	Effects	Descrip-tive	Interven-tional	
1	Akbari (2011)[1]	●	-	-	●	-	Asia
2	Augustin (2011)[2]	●	-	-	●	-	Europe
3	Frese (2011)[3]	-	●	-	●	-	Europe
4	Paul (2011)[4]	●	-	-	●	-	Europe
5	Ritchie (2011)[5]	●	-	-	●	-	Australia
6	Wu (2011)[6, 7]	●	-	-	●	-	North America
7	Hollestein (2012)[8]	●	●	-	●	-	Europe
8	Joly (2012)[9]	●	●	-	●	-	Europe
9	Bonaccorsi (2013)[10]	●	-	●	-	●	Europe
10	Danielsen (2013)[12]	●	-	-	●	-	Europe
11	Etzkorn (2013)[11]	●	-	-	●	-	North America
12	Flohil (2013)[13]	●	-	-	●	-	Europe
13	Okuno (2013)[14]	-	-	●	-	●	Asia
14	Robsahm (2013)[15]	●	-	-	●	-	Europe
15	Wysong (2013)[16]	-	●	-	●	-	North America
16	Gontijo Guerra (2014)[17]	●	●	-	●	-	North America
17	Gontijo Guerra (2014)[18]	-	●	-	●	-	North America
18	Hsieh (2014)[19]	●	-	-	●	-	Asia
19	Landis (2014)[20]	-	●	-	●	-	North America
20	Caretti (2015)[21]	●	●	-	●	-	North America
21	Cybulski (2015)[22]	●	-	-	●	-	Europe
22	Duim (2015)[23]	●	-	-	●	-	South America
23	Hay (2015)[24]	-	●	-	●	-	Global
24	Kiiski (2015)[25]	●	-	-	●	-	Europe
25	Romani (2015)[26]	●	-	-	●	-	Global
26	Cinotti (2016)[27]	●	-	-	●	-	Europe
27	Hsieh (2016)[28]	●	-	-	●	-	Asia
28	Liu (2016)[29]	-	●	-	●	-	North America
29	Trautmann (2016)[30]	●	-	●	●	-	Europe
30	Tseng (2016)[31]	●	-	-	●	-	Asia
31	Alexandridou (2017)[32]	-	-	●	●	-	Europe
32	Asokan (2017)[33]	●	●	-	●	-	Asia
33	Barbaric (2017)[34]	●	-	-	●	-	Europe
34	George (2017)[35]	●	-	-	●	-	Asia
35	Hahnel (2017)[36]	●	●	-	●	-	Global
36	Henchoz (2017)[37]	-	●	-	●	-	Europe
37	Iizaka (2017)[38]	●	-	-	●	-	Asia
38	Karimkhani (2017)[39]	-	●	-	●	-	Global
39	Karimkhani (2017)[40]	-	●	-	●	-	Global
40	Kim (2017)[41]	●	-	-	●	-	Asia
41	Lee (2017)[42]	-	●	-	●	-	Asia

42	Pandeya (2017)[43]	●	-	-	●	-	Australia
43	Thorslund (2017)[44]	●	-	-	●	-	Europe
44	Abuabara (2018)[45]	●	-	-	●	-	Europe
45	Aitken (2018)[46]	●	●	●	●	-	Australia
46	Cowdell (2018)[47]	-	●	-	●	-	Europe
47	Drewitz (2018)[48]	●	-	-	●	-	Europe
48	Dziunycz (2018)[49]	●	-	-	●	-	Europe
49	Hu (2018)[50]	●	●	-	●	-	Asia
50	Lichterfeld-Kottner (2018)[51]	●	●	-	●	-	Europe
51	Sanders (2018)[52]	●	-	-	●	-	Europe
52	Steglich (2018)[53]	●	-	-	●	-	South America
53	Augustin (2019)[54, 55]	●	-	-	●	-	Europe
54	Drewitz (2019)[56]	●	-	-	●	-	Europe
55	Mekic (2019)[57]	●	-	-	●	-	Europe
56	Meyers (2019)[58]	-	●	-	●	-	North America
57	Sari (2019)[59]	●	-	-	●	-	Asia
58	d. Silva (2019)[60]	-	-	●	●	-	South America
59	Tizek (2019)[61]	●	-	-	●	-	Europe
60	Venables (2019)[62]	●	-	-	●	-	Europe
61	Venables (2019)[63]	●	-	-	●	-	Europe
62	Bianchi (2020)[64]	●	-	●	●	●	South America
63	Chang (2020)[65]	-	-	●	●	-	North America
64	Everink (2020)[66]	●	-	-	●	-	Europe
65	Fors (2020)[67]	●	-	-	●	-	South America
66	Kottner (2020)[68]	●	-	-	●	-	Europe
67	Prasad (2020)[69]	●	-	●	-	●	Asia
68	Sideris (2020)[70]	-	-	●	●	-	Australia
69	Sinikumpu (2020)[71]	●	●	-	●	-	Europe
70	Tokez (2020)[72]	●	-	-	●	-	Europe
71	Tseng (2020)[73]	●	●	-	●	-	North America
72	Yew (2020)[74]	●	-	-	●	-	Asia
73	Yong (2020)[75]	-	●	-	●	-	Asia
74	Bai (2021)[76]	●	●	-	●	-	Asia
75	Barbieri (2021)[77]	●	-	-	●	-	North America
76	Bucchi (2021)[78]	●	-	-	●	-	Europe
77	Drewitz (2021)[79]	●	-	-	●	-	Europe
78	Madani (2021)[80]	●	-	-	●	-	North America
79	Memon (2021)[81]	●	-	-	●	-	Europe
80	Neena (2021)[82]	●	●	●	●	-	Asia
81	Tang (2021)[83]	●	-	-	●	-	Asia
82	Waldmann (2021)[84]	●	●	-	●	-	Europe
83	Blazek (2022)[85]	●	●	●	●	-	Australia
84	Botvid (2022)[86]	●	-	-	●	-	North America
85	Choon (2022)[87]	●	-	-	●	-	Asia
86	Lu (2022)[88]	●	-	-	●	-	Global
87	Matsumoto (2022)[89]	●	-	●	●	-	North America

88	Navsaria (2022)[90]	●	-	●	●	-	North America
89	v. Niekerk (2022)[91]	●	●	-	●	-	Europe
90	Radkiewicz (2022)[92]	●	-	-	●	-	Europe
91	Raghuwanshi (2022)[93]	●	●	-	●	-	Asia
92	Rodriguez-Betancourt (2022)[94]	●	●	-	●	-	South America
93	Huang (2023)[95]	●	-	-	●	-	Asia
94	Keim (2023)[96]	●	-	-	●		Europe
95	Xu (2023)[97]	●	-	-	●	-	Asia
	**Total**	**77**	**30**	**14**	**92**	**4**	

REFERENCES

1. Akbari ME, Rafiee M, Khoei MA et al. Incidence and survival of cancers in the elderly population in Iran: 2001-2005. *Asian Pac J Cancer Prev* 2011; **12**:3035-9.
2. Augustin M, Herberger K, Hintzen S et al. Prevalence of skin lesions and need for treatment in a cohort of 90 880 workers. *Br J Dermatol* 2011; **165**:865-73.
3. Frese T, Herrmann K, Sandholzer H. Pruritus as reason for encounter in general practice. *J Clin Med Res* 2011; **3**:223-9.
4. Paul C, Maumus-Robert S, Mazereeuw-Hautier J et al. Prevalence and risk factors for xerosis in the elderly: a cross-sectional epidemiological study in primary care. *Dermatology* 2011; **223**:260-5.
5. Ritchie SR, Fraser JD, Libby E et al. Demographic variation in community-based MRSA skin and soft tissue infection in Auckland, New Zealand. *New Zealand Medical Journal* 2011; **124**.
6. Wu J, Guo Z, Berman R et al. P2-119: Occurrence of nonmelanoma skin cancer in the elderly with and without Alzheimer's disease in the US [abstract]. In: Alzheimer's Association International Conference, AAIC 11, Paris, France. *Alzheimer's & Dementia* 2011; 7:S347. doi: 10.1016/j.jalz.2011.05.1007. Available from: https://alz-journals.onlinelibrary.wiley.com/doi/10.1016/j.jalz.2011.05.1007. [accessed 20.04.2023].
7. Wu J, Guo Z, Berman R et al. 698. Risk of Non-Melanoma Skin Cancer in Elderly Patients with Alzheimer's Disease [abstract]. In: 27th International Conference on Pharmacoepidemiology and Therapeutic Risk Management, Chicago, United States. *Pharmacoepidemiology and Drug Safety* 2011; 20:S303-4. doi: 10.1002/pds.2206. Available from: https://onlinelibrary.wiley.com/doi/10.1002/pds.2206. [accessed 02.05.2023].
8. Hollestein LM, van den Akker SA, Nijsten T et al. Trends of cutaneous melanoma in The Netherlands: increasing incidence rates among all Breslow thickness categories and rising mortality rates since 1989. *Ann Oncol* 2012; **23**:524-30.
9. Joly P, Baricault S, Sparsa A et al. Incidence and mortality of bullous pemphigoid in France. *J Invest Dermatol* 2012; **132**:1998-2004.
10. Bonaccorsi G, Lorini C, Santomauro F et al. 202 Impact of different pads in elderly assisted in home care [abstract]. In: 43rd Annual Meeting of the International Continence Society, ICS 2013, Barcelona, Spain. *Neurourol. Urodyn.* 2013:802-3. doi: 10.1002/nau.22472. Available from: https://onlinelibrary.wiley.com/doi/10.1002/nau.22472. [accessed 02.05.2023].
11. Etzkorn JR, Parikh RP, Marzban SS et al. Identifying risk factors using a skin cancer screening program. *Cancer Control* 2013; **20**:248-54.
12. Danielsen K, Olsen AO, Wilsgaard T, Furberg AS. Is the prevalence of psoriasis increasing? A 30-year follow-up of a population-based cohort. *Br J Dermatol* 2013; **168**:1303-10.
13. Flohil SC, van der Leest RJ, Dowlatshahi EA et al. Prevalence of actinic keratosis and its risk factors in the general population: the Rotterdam Study. *J Invest Dermatol* 2013; **133**:1971-8.
14. Okuno Y, Takao Y, Miyazaki Y et al. Assessment of skin test with varicella-zoster virus antigen for predicting the risk of herpes zoster. *Epidemiol Infect* 2013; **141**:706-13.
15. Robsahm TE, Bergva G, Hestvik UE, Moller B. Sex differences in rising trends of cutaneous malignant melanoma in Norway, 1954-2008. *Melanoma Res* 2013; **23**:70-8.
16. Wysong A, Linos E, Hernandez-Boussard T et al. Nonmelanoma skin cancer visits and procedure patterns in a nationally representative sample: national ambulatory medical care survey 1995-2007. *Dermatol Surg* 2013; **39**:596-602.
17. Gontijo Guerra S, Vasiliadis HM, Preville M, Berbiche D. Skin conditions in community-living older adults: prevalence and characteristics of medical care service use. *J Cutan Med Surg* 2014; **18**:186-94.
18. Gontijo Guerra S, Preville M, Vasiliadis HM, Berbiche D. Association between skin conditions and depressive disorders in community-dwelling older adults. *J Cutan Med Surg* 2014; **18**:256-64.
19. Hsieh C-F, Huang W-F, Chiang Y-T. 157. The Incidence of Actinic Keratosis and Risk of Non-Melanoma Skin Cancer in Taiwan [abstract]. In: 30th International Conference on Pharmacoepidemiology and Therapeutic Risk Management, Taipei, Taiwan. *Pharmacoepidemiol.*

Drug Saf. 2014; 23(S1):84-5. doi: 10.1002/pds.3701. Available from: https://onlinelibrary.wiley.com/doi/10.1002/pds.3701. [accessed 20.04.2023].
20. Landis ET, Davis SA, Taheri A, Feldman SR. Top dermatologic diagnoses by age. *Dermatol Online J* 2014; **20**:22368.
21. Caretti KL, Mehregan DR, Mehregan DA. A survey of self-reported skin disease in the elderly African-American population. *Int J Dermatol* 2015; **54**:1034-8.
22. Cybulski M, Krajewska-Kulak E. Skin diseases among elderly inhabitants of Bialystok, Poland. *Clin Interv Aging* 2015; **10**:1937-43.
23. Duim E, Sa FH, Duarte YA et al. Prevalence and characteristics of lesions in elderly people living in the community. *Rev Esc Enferm USP* 2015; **49 Spec No**:51-7.
24. Hay RJ, Fuller LC. Global burden of skin disease in the elderly: a grand challenge to skin health. *G Ital Dermatol Venereol* 2015; **150**:693-8.
25. Kiiski V, Susitaival P, Remitz A, Reitamo S. 086 Is atopic dermatitis more persistent than previously estimated? [abstract]. In: 45th Annual Meeting of the European Society for Dermatological Research, Rotterdam, Netherlands. *Journal of Investigative Dermatology* 2015; 135:S15. doi: 10.1038/jid.2015.266. Available from: https://www.sciencedirect.com/science/article/pii/S0022202X15601993?via%3Dihub. [accessed 02.05.2023].
26. Romani L, Steer AC, Whitfeld MJ, Kaldor JM. Prevalence of scabies and impetigo worldwide: a systematic review. *Lancet Infect Dis* 2015; **15**:960-7.
27. Cinotti E, Perrot JL, Labeille B et al. Skin tumours and skin aging in 209 French elderly people: the PROOF study. *Eur J Dermatol* 2016; **26**:470-6.
28. Hsieh C-F, Chiang Y-T, Chiu H-Y, Huang W-F. A Nationwide Cohort Study of Actinic Keratosis in Taiwan*. *International Journal of Gerontology* 2016; **10**:218-22.
29. Liu T, Brienza R. What brings an older veteran to an urgent visit (UV) A review of the chief concerns by veterans aged 65 and older who presented for uv durin a 6-month period at the West Haven Veteran Affairs Center of excellence in Primary Care Education (VA COEPCE), an interprofessional academic patient aligned care team (PACT) [abstract]. In: Abstracts from the 2016 Society of General Internal Medicine Annual Meeting. *J Gen Intern Med* 2016; 31(Suppl 2):S468-9. doi: 10.1007/s11606-016-3657-7. Available from: https://link.springer.com/article/10.1007/s11606-016-3657-7. [accessed 02.05.2023].
30. Trautmann F, Meier F, Seidler A, Schmitt J. Effects of the German skin cancer screening programme on melanoma incidence and indicators of disease severity. *Br J Dermatol* 2016; **175**:912-9.
31. Tseng HW, Shiue YL, Tsai KW et al. Risk of skin cancer in patients with diabetes mellitus: A nationwide retrospective cohort study in Taiwan. *Medicine (Baltimore)* 2016; **95**:e4070.
32. Alexandridou M, Bollaerts K. Pin10 Zoster Vaccine Effectiveness against incident herpes zoster and post-herpetic neuralgia in elderly in the UK [abstract]. In: ISPOR 20th Annual European Congress, Glasgow, United Kingdom. *Value Health* 2017; 20:A780. doi: 10.1016/j.jval.2017.08.2263. Available from: https://www.sciencedirect.com/science/article/pii/S1098301517325974?via%3Dihub. [accessed 26.04.2023].
33. Asokan N, Binesh VG. Cutaneous problems in elderly diabetics: A population-based comparative cross-sectional survey. *Indian J Dermatol Venereol Leprol* 2017; **83**:205-11.
34. Barbaric J, Laversanne M, Znaor A. Malignant melanoma incidence trends in a Mediterranean population following socioeconomic transition and war: results of age-period-cohort analysis in Croatia, 1989-2013. *Melanoma Res* 2017; **27**:498-502.
35. George LS, Deshpande S, Krishna Kumar MK, Patil RS. Morbidity pattern and its sociodemographic determinants among elderly population of Raichur district, Karnataka, India. *J Family Med Prim Care* 2017; **6**:340-4.
36. Hahnel E, Lichterfeld A, Blume-Peytavi U, Kottner J. The epidemiology of skin conditions in the aged: A systematic review. *J Tissue Viability* 2017; **26**:20-8.

37. Henchoz Y, Bula C, Guessous I et al. Chronic symptoms in a representative sample of community-dwelling older people: a cross-sectional study in Switzerland. *BMJ Open* 2017; **7**:e014485.
38. Iizaka S, Nagata S, Sanada H. Nutritional Status and Habitual Dietary Intake Are Associated with Frail Skin Conditions in Community-Dwelling Older People. *J Nutr Health Aging* 2017; **21**:137-46.
39. Karimkhani C, Colombara DV, Drucker AM et al. The global burden of scabies: a cross-sectional analysis from the Global Burden of Disease Study 2015. *Lancet Infect Dis* 2017; **17**:1247-54.
40. Karimkhani C, Dellavalle RP, Coffeng LE et al. Global Skin Disease Morbidity and Mortality: An Update From the Global Burden of Disease Study 2013. *JAMA Dermatol* 2017; **153**:406-12.
41. Kim J, Choi Y, Shin J et al. Incidence of Pressure Ulcers During Home and Institutional Care Among Long-Term Care Insurance Beneficiaries With Dementia Using the Korean Elderly Cohort. *J Am Med Dir Assoc* 2017; **18**:638 e1- e5.
42. Lee HJ, Ju YJ, Park EC et al. Effects of home-visit nursing services on hospitalization in the elderly with pressure ulcers: a longitudinal study. *Eur J Public Health* 2017; **27**:822-6.
43. Pandeya N, Olsen CM, Whiteman DC. The incidence and multiplicity rates of keratinocyte cancers in Australia. *Med J Aust* 2017; **207**:339-43.
44. Thorslund K, Seifert O, Nilzen K, Gronhagen C. Incidence of bullous pemphigoid in Sweden 2005-2012: a nationwide population-based cohort study of 3761 patients. *Arch Dermatol Res* 2017; **309**:721-7.
45. Abuabara K, Magyari A, Margolis DJ, Langan M. The prevalence of atopic eczema across the lifespan: a U.K. population-based cohort study [abstract]. In: Abstracts of the 10th George Rajka International Symposium on Atopic Dermatitis, Utrecht, Netherlands. *British Journal of Dermatology* 2018; 179:e58. doi: 10.1111/bjd.16718. Available from: https://academic.oup.com/bjd/article/179/1/e5/6732334?login=true. [accessed 20.04.2023].
46. Aitken JF, Youlden DR, Baade PD et al. Generational shift in melanoma incidence and mortality in Queensland, Australia, 1995-2014. *Int J Cancer* 2018; **142**:1528-35.
47. Cowdell F, Dyson J, Long J, Macleod U. Self-reported skin concerns: An epidemiological study of community-dwelling older people. *Int J Older People Nurs* 2018; **13**:e12195.
48. Drewitz KP, Stark K, Zimmermann ME et al. P102 | Prevalence and determinants of Psoriasis in a cross- sectional study of the elderly—results from the German AugUR study [abstract]. In: 45th Annual Meeting of the Arbeitsgemeinschaft Dermatologische Forschung, ADF 2018, Zurich, Switzerland. *Experimental Dermatology* 2018; 27:e43-4. doi: 10.1111/exd.13486. Available from: https://onlinelibrary.wiley.com/doi/10.1111/exd.13486. [accessed 20.04.2023].
49. Dziunycz PJ, Schuller E, Hofbauer GFL. Prevalence of Actinic Keratosis in Patients Attending General Practitioners in Switzerland. *Dermatology* 2018; **234**:214-9.
50. Hu L, Jin S, Chen L, Wang Y. Trends in the incidence and mortality of cutaneous melanoma in Hong Kong between 1983 and 2015. *Int J Clin Exp Med* 2018; **11**:8259-66.
51. Lichterfeld-Kottner A, Lahmann N, Blume-Peytavi U et al. Dry skin in home care: A representative prevalence study. *J Tissue Viability* 2018; **27**:226-31.
52. Sanders MGH, Pardo LM, Franco OH et al. Prevalence and determinants of seborrhoeic dermatitis in a middle-aged and elderly population: the Rotterdam Study. *Br J Dermatol* 2018; **178**:148-53.
53. Steglich RB, Coelho K, Cardoso S et al. Epidemiological and histopathological aspects of primary cutaneous melanoma in residents of Joinville, 2003-2014. *An Bras Dermatol* 2018; **93**:45-53.
54. Augustin M, Kirsten N, Koerber A et al. Epidemiology of dry skin in the general population [abstract]. In: 24th World Congress of Dermatology, Milan, Italy. 2019. Available from: https://www.wcd2019milan-dl.org/abstract-book/documents/abstracts/12-epidemiology/epidemiology-of-dry-skin-in-2502.pdf. [accessed 27.04.2023].
55. Augustin M, Kirsten N, Korber A et al. Prevalence, predictors and comorbidity of dry skin in the general population. *J Eur Acad Dermatol Venereol* 2019; **33**:147-50.
56. Drewitz KP, Stark K, Zimmermann ME et al. P086 | Frequency and comorbidities of eczema in an elderly population in Germany: results from augur [abstract]. In: 46th Annual Meeting of the

Arbeitsgemeinschaft Dermatologische Forschung, ADF, Munich, Germany. *Experimental Dermatology* 2019; 28:e41-2. doi: 10.1111/exd.13859. Available from: https://onlinelibrary.wiley.com/doi/10.1111/exd.13859. [accessed 20.04.2023].
57. Mekic S, Jacobs LC, Gunn DA et al. Prevalence and determinants for xerosis cutis in the middle-aged and elderly population: A cross-sectional study. *J Am Acad Dermatol* 2019; **81**:963-9 e2.
58. Meyers JL, Candrilli SD, Rausch DA et al. Costs of herpes zoster complications in older adults: A cohort study of US claims database. *Vaccine* 2019; **37**:1235-44.
59. Sari SP, Everink IH, Sari EA et al. The prevalence of pressure ulcers in community-dwelling older adults: A study in an Indonesian city. *Int Wound J* 2019; **16**:534-41.
60. Silva ESD, Dumith SC. Non-use of sunscreen among adults and the elderly in southern Brazil. *An Bras Dermatol* 2019; **94**:567-73.
61. Tizek L, Schielein MC, Seifert F et al. Skin diseases are more common than we think: screening results of an unreferred population at the Munich Oktoberfest. *J Eur Acad Dermatol Venereol* 2019; **33**:1421-8.
62. Venables ZC, Nijsten T, Wong KF et al. Epidemiology of basal and cutaneous squamous cell carcinoma in the U.K. 2013-15: a cohort study. *Br J Dermatol* 2019; **181**:474-82.
63. Venables ZC, Autier P, Nijsten T et al. Nationwide Incidence of Metastatic Cutaneous Squamous Cell Carcinoma in England. *JAMA Dermatol* 2019; **155**:298-306.
64. Bianchi M, Santos A, Cordioli E. Benefits of Teledermatology for Geriatric Patients: Population-Based Cross-Sectional Study. *J Med Internet Res* 2020; **22**:e16700.
65. Chang C-Y, Park H, Lo-Ciganic J. 3946 | The prevalence of sun protective behaviors across different age groups in the US population: Findings from the 2015 US Health Interview Survey [abstract]. In: Special Issue: Abstracts of the 36th International Conference on Pharmacoepidemiology & Therapeutic Risk Management, Virtual. *Pharmacoepidemiol Drug Saf* 2020; 29(Suppl 3):306-7. doi: 10.1002/pds.5114. Available from: https://onlinelibrary.wiley.com/doi/10.1002/pds.5114. [accessed 02.05.2023].
66. Everink IHJ, Kottner J, van Haastregt JCM et al. Skin areas, clinical severity, duration and risk factors of intertrigo: A secondary data analysis. *J Tissue Viability* 2020; **30**:102-7.
67. Fors M, Gonzalez P, Viada C et al. Actinic keratoses in subjects from la Mitad del Mundo, Ecuador. *BMC Dermatol* 2020; **20**:11.
68. Kottner J, Everink I, van Haastregt J et al. Prevalence of intertrigo and associated factors: A secondary data analysis of four annual multicentre prevalence studies in the Netherlands. *Int J Nurs Stud* 2020; **104**:103437.
69. Prasad S, Hussain N, Sharma S et al. Impact of Pressure Injury Prevention Protocol in Home Care Services on the Prevalence of Pressure Injuries in the Dubai Community. *Dubai Medical Journal* 2020; **3**:99-104.
70. Sideris E, Thomas SJ. Patients' sun practices, perceptions of skin cancer and their risk of skin cancer in rural Australia. *Health Promot J Austr* 2020; **31**:84-92.
71. Sinikumpu SP, Jokelainen J, Haarala AK et al. The High Prevalence of Skin Diseases in Adults Aged 70 and Older. *J Am Geriatr Soc* 2020; **68**:2565-71.
72. Tokez S, Wakkee M, Louwman M et al. Assessment of Cutaneous Squamous Cell Carcinoma (cSCC) In situ Incidence and the Risk of Developing Invasive cSCC in Patients With Prior cSCC In situ vs the General Population in the Netherlands, 1989-2017. *JAMA Dermatol* 2020; **156**:973-81.
73. Tseng HF, Bruxvoort K, Ackerson B et al. The Epidemiology of Herpes Zoster in Immunocompetent, Unvaccinated Adults >/=50 Years Old: Incidence, Complications, Hospitalization, Mortality, and Recurrence. *J Infect Dis* 2020; **222**:798-806.
74. Yew YW, Kuan AHY, Ge L et al. Psychosocial impact of skin diseases: A population-based study. *PLoS One* 2020; **15**:e0244765.
75. Yong SS, Kwan Z, Ch'ng CC et al. Self-reported generalised pruritus among community-dwelling older adults in Malaysia. *BMC Geriatr* 2020; **20**:223.
76. Bai R, Huang H, Li M, Chu M. Temporal Trends in the Incidence and Mortality of Skin Malignant Melanoma in China from 1990 to 2019. *J Oncol* 2021; **2021**:9989824.

77. Barbieri JS, Rodriguez O, Rosenbach M, Margolis D. Incidence and Prevalence of Granuloma Annulare in the United States. *JAMA Dermatol* 2021; **157**:824-30.
78. Bucchi L, Mancini S, Crocetti E et al. Mid-term trends and recent birth-cohort-dependent changes in incidence rates of cutaneous malignant melanoma in Italy. *Int J Cancer* 2021; **148**:835-44.
79. Drewitz KP, Stark KJ, Zimmermann ME et al. Frequency of hand eczema in the elderly: Cross-sectional findings from the German AugUR study. *Contact Dermatitis* 2021; **85**:489-93.
80. Madani S, Marwaha S, Dusendang JR et al. Ten-Year Follow-up of Persons With Sun-Damaged Skin Associated With Subsequent Development of Cutaneous Squamous Cell Carcinoma. *JAMA Dermatol* 2021; **157**:559-65.
81. Memon A, Bannister P, Rogers I et al. Changing epidemiology and age-specific incidence of cutaneous malignant melanoma in England: An analysis of the national cancer registration data by age, gender and anatomical site, 1981-2018. *Lancet Reg Health Eur* 2021; **2**:100024.
82. Neena V, Asokan N, Jose R, Sarin A. Prevalence of eczema among older persons: A population-based cross-sectional study. *Indian J Dermatol Venereol Leprol* 2021; **89**:426-30.
83. Tang L, Li F, Xu F et al. Prevalence of vitiligo and associated comorbidities in adults in Shanghai, China: a community-based, cross-sectional survey. *Ann Palliat Med* 2021; **10**:8103-11.
84. Waldmann A, Pritzkuleit R, Labohm L, Katalinic A. Epidemiologie von Krebs im hohen Lebensalter. *best practice onkologie* 2021; **16**:586-97.
85. Blazek K, Furestad E, Ryan D et al. The impact of skin cancer prevention efforts in New South Wales, Australia: Generational trends in melanoma incidence and mortality. *Cancer Epidemiol* 2022; **81**:102263.
86. Botvid SHC, Storgaard Hove L, Backe MB et al. Low prevalence of patients diagnosed with psoriasis in Nuuk: a call for increased awareness of chronic skin disease in Greenland. *Int J Circumpolar Health* 2022; **81**:2068111.
87. Choon SE, Wright AK, Griffiths CEM et al. Incidence and prevalence of psoriasis in multiethnic Johor Bahru, Malaysia: a population-based cohort study using electronic health data routinely captured in the Teleprimary Care (TPC(R)) clinical information system from 2010 to 2020: Classification: Epidemiology. *Br J Dermatol* 2022; **187**:713-21.
88. Lu L, Chen L, Xu Y, Liu A. Global incidence and prevalence of bullous pemphigoid: A systematic review and meta-analysis. *J Cosmet Dermatol* 2022; **21**:4818-35.
89. Matsumoto M, Wack S, Weinstock MA et al. Five-Year Outcomes of a Melanoma Screening Initiative in a Large Health Care System. *JAMA Dermatol* 2022; **158**:504-12.
90. Navsaria L, Li Y, Nowakowska M et al. LB911 Incidence and treatments of actinic keratosis in the Medicare population: A cohort study [abstract]. In: Society for Investigative Dermatology (SID) 2022 Meeting, Portland, United States. *Journal of Investigative Dermatology* 2022; 142:B10. doi: 10.1016/j.jid.2022.05.929. Available from: https://www.sciencedirect.com/science/article/pii/S0022202X22013707?via%3Dihub. [accessed 20.04.2023].
91. van Niekerk CC, Otten J, van Rossum MM et al. Trends in three major histological subtypes of cutaneous melanoma in the Netherlands between 1989 and 2016. *Int J Dermatol* 2023; **62**:508-13.
92. Radkiewicz C, Jarkvik Kronmark J, Adami HO, Edgren G. Declining Cancer Incidence in the Elderly: Decreasing Diagnostic Intensity or Biology? *Cancer Epidemiol Biomarkers Prev* 2022; **31**:280-6.
93. Raghuwanshi AS, Diwan S, Singh H, Raghuwanshi KC. A Cross-Sectional Study to Assess the Psychosocial impact of Skin Diseases. *International Journal of Pharmaceutical and Clinical Research* 2022; **14**:1061-7.
94. Rodriguez-Betancourt JD, Arias-Ortiz N. Cutaneous melanoma incidence, mortality, and survival in Manizales, Colombia: a population-based study. *J Int Med Res* 2022; **50**:3000605221106706.
95. Huang J, Zhang L, Shi L et al. An epidemiological study on skin tumors of the elderly in a community in Shanghai, China. *Sci Rep* 2023; **13**:4441.

96. Keim U, Katalinic A, Holleczek B et al. Incidence, mortality and trends of cutaneous squamous cell carcinoma in Germany, the Netherlands, and Scotland. *Eur J Cancer* 2023; **183**:60-8.
97. Xu Q, Wang X, Bai Y et al. Trends of non-melanoma skin cancer incidence in Hong Kong and projection up to 2030 based on changing demographics. *Ann Med* 2023; **55**:146-54.
