# Supplementary material for: Skin health of community-living older people: a scoping review
Source: Arch Dermatol Res. 2024 Jun 1;316(6):319. doi: 10.1007/s00403-024-03059-0 (PMC11144137; doi:10.1007/s00403-024-03059-0)
Supplement: Supplementary file 4 — Supplementary Material 4 [file 403_2024_3059_MOESM4_ESM.pdf]

Supplementary material 4

Article title: Skin health of community-living older people: a scoping review

Authors: Jan Kottner[1], Alexandra Fastner[1], Dimitra-Aikaterini Lintzeri[2], Ulrike Blume-Peytavi[2], Christopher E. M. Griffiths[3,4]

Institutions

[1]Institute of Clinical Nursing Science, Charité Universitätsmedizin Berlin, Berlin, Germany.

[2]Department of Dermatology, Venerology and Allergology, Charité Universitätsmedizin Berlin, Berlin, Germany.

[3]Department of Dermatology, King's College Hospital, King's College London, London, UK.

[4]Centre for Dermatology Research, NIHR Manchester Biomedical Research Centre, The University of Manchester, Manchester, UK.

Corresponding author

Jan Kottner

Charité – Universitätsmedizin Berlin,

Institute of Clinical Nursing Science,

Charitéplatz 1,

10117 Berlin, Germany

Email: jan.kottner@charite.de

Table S4: Summary of included studies for review question 1

No.	First author (year)	Design				Geographic location	Sample characteristics		Results				Notes
		Cross-sec-tional	Longi-tudinal	Secondary data analysis/ review	Registry data		Sample size, n (Time period)	Age (years)	Skin condition	Prevalence/ Proportion	Incidence	Other	
1	Akbari (2011)[1]	-	-	-	x	Iran	Unclear (Five years)	60 to 74	Skin cancer	-	301.2/100,000		‚Skin cancer' not specified
								75 to 90			450.1/100,000		
								> 90			2191/100,000		
								≥ 60			371.0/100,000		
2	Augustin (2011)[2]	x	-	-	-	Germany	6,860	61-70	Solar (senile) lentigines	71.6%	-	-	Proportion of AK extracted from figure 1
									SK	66.7%			
									Dermal naevi	25.4%			
									At least one dermatomycoses condition	23.5%			
									Histiocytoma dermatofibroma	20.4%			
									Onychomycosis	17.6%			
									Papillomatous naevi	15.8%			
									AK	12.0%			
									Tinea pedis	9.5%			
									More than 40 naevi	7.2%			
									Rosacea	6.2%			
									Cafe´-au-lait patches	4.1%			
									Bacterial folliculitis	3.6%			
									Lipoma	2.8%			
									Warts of the feet	2.3%			
									Psoriasis	2.2%			
									Xerotic eczema	1.9%			
									Other inflammatory diseases	1.8%			
									AD	1.0%			
									Pyoderma	0.9%			
									Other viral diseases of the skin	0.8%			
									Pityriasis versicolor	0.8%			
									Hand eczema, (cumulative toxic, allergic)	0.8%			

No.	First author (year)	Design				Geographic location	Sample characteristics		Results				Notes
		Cross-sec-tional	Longi-tudinal	Secondary data analysis/ review	Registry data		Sample size, n (Time period)	Age (years)	Skin condition	Prevalence/ Proportion	Incidence	Other	
									Vitiligo	0.7%			
									Tinea corporis	0.6%			
									Intertrigo	0.6%			
									Other cysts	0.5%			
									Warts of the hands	0.4%			
									Other mycotic diseases	0.4%			
									Contact dermatitis	0.3%			
									Acne	0.2%			
									Other bacterial diseases	0.2%			
									Lichen planus	0.1%			
3	Paul (2011)[3]	x	-	-	-	France	756	75.1 (mean)	Xerosis cutis	55.6%	-	-	-
4	Ritchie (2011)[4]	-	-	x	-	New Zealand (NZ)	Unclear (One year)	> 80	mr-MRSA (colonisation/ infection in wound)	-	627/100,000 people /year (in NZ European)	-	-
5	Wu (2011)[5, 6]	-	-	x	-	USA	27,076 (subjects with Alzheimer Disease diagnosis) (Three years)	84.1 (mean)	NMSC	-	3.0 to 4.1/100 person-years	-	-
							27,076 (subjects without Alzheimer Disease diagnosis) (Three years)				4.2 to 5.6/100 person-years		
6	Hollestein (2012)[7]	-	-	-	x	Netherlands	Unclear	≥ 65	Melanoma	-	M: 5,7/100,000 person-years F: 4.7/100,000 person-years (2004 to 2008)	-	-
7	Joly (2012)[8]	-	-	x	-	France	502 cases (Five years)	≥70	BP	-	162/1,000,000 inhabitants per year	-	-
								≥75			224/1,000,000 inhabitants per year		

No.	First author (year)	Design				Geographic location	Sample characteristics		Results				Notes
		Cross-sectional	Longitudinal	Secondary data analysis/ review	Registry data		Sample size, n (Time period)	Age (years)	Skin condition	Prevalence/ Proportion	Incidence	Other	
								≥80			329/1,000,000 inhabitants per year		
								≥85			507/1,000,000 inhabitants per year		
8	Bonaccorsi (2013)[9]	-	x	-	-	Italy	377	85.4 (mean)	Pressure injury	22.6%	6.2 %	-	-
9	Danielsen (2013)[10]	-	x	-	-	Norway	Unclear	60 to 69	Psoriasis	12% (2007/2008)	-	-	Self-reported<br><br>Proportions extracted from figure 1
								70 to 79		12% (2007/2008)			
10	Etzkorn (2013)[11]	x	-	-	-	USA	1,202	60-69	AK, SCC, BCC	30.9%	-	-	-
									Melanoma, Atypical Naevi	7.7%			
							501	70-79	AK, SCC, BCC	43.9%			
									Melanoma, Atypical Naevi	6.0%			
							87	≥ 80	AK, SCC, BCC	49.4%			
									Melanoma, Atypical Naevi	3.4%			
11	Flohil (2013)[12]	x	-	-	-	Netherlands	947	70-79	1–3 AKs	23.1%	-	-	Proportions recalculated based on table 1
									4–9 AKs	10.3%			
									≥ 10 AKs	10.3%			
							240	≥ 80	1–3 AKs	24.2%			
									4–9 AKs	12.1%			
									≥ 10 AKs	13.3%			
12	Robsahm (2013)[13]	-	-	-	x	Norway	Unclear	≥70	Melanoma	-	F: 60 /100,000 population<br>M: 100 /100,000 population<br>(Years 2004-2008)	-	Incidence extracted from figure 2
13	Gontijo - Guerra (2014a)[14]	-	x	-	-	Canada	2,494 (T1)<br>1,985 (T2)	73.8 (mean)	Any physician diagnosed skin condition	22.8% (T1)<br>20.7% (T2)	-	Two-year period prevalence rate : 31.4%	One-year prevalence rates calculated for the 12-month period prior to interviews (T1, T2)
									Any self-reported skin condition	13.3% (T1)<br>13.1% (T2)<br>6.2% (Both)	-	Presence of skin condition over two years (self-reported) 18.7%	
14	Hsieh (2014)[15]	-		x	-	Taiwan	Unclear (Ten years)	≥ 65	AK	-	7.8 to 14.0 /10,000 persons	-	-

No.	First author (year)	Design				Geographic location	Sample characteristics		Results				Notes
		Cross-sec-tional	Longi-tudinal	Secondary data analysis/ review	Registry data		Sample size, n (Time period)	Age (years)	Skin condition	Prevalence/ Proportion	Incidence	Other	
15	Caretti (2015)[16]	x	-	-	-	USA (Detroit)	101	71 (median)	Eczema/ dermatitis	28.7%	-	-	Self-reported
									Fungal skin infection	16.8%			
									Alopecia	6.9%			
									Viral skin infection	4.9%			
									Urticaria	4.9%			
									Keloid	2.9%			
									Pseudofolliculitis barbae	1.9%			
									Bacterial skin infection	1.9%			
									Psoriasis	0.9%			
									Seborrheic dermatitis	0.9%			
									Lupus	0.9%			
									Rosacea	0.9%			
									BCC	0.9%			
16	Cybulski (2015)[17]	x	-	-	-	Poland	100	>60	Any dermatologist confirmed skin disease	27.0%	-	-	-
									Fungal infection	33.3%			
									AD	18.5%			
									Balding	18.5%			
									Psoriasis	14.8%			
									Shingles	11.1%			
									Epidermal cyst	7.4%			
									Acne	7.4%			
									Eczema	7.4%			
									Vitiligo	3.7%			
									Furuncle	3.7%			
									Lichen planus	3.7%			
									Dandruff	3.7%			
									Angioma	3.7%			
									Rash	3.7%			
									Erysipelas	3.7%			

No.	First author (year)	Design				Geographic location	Sample characteristics		Results				Notes
		Cross-sectional	Longitudinal	Secondary data analysis/ review	Registry data		Sample size, n (Time period)	Age (years)	Skin condition	Prevalence/ Proportion	Incidence	Other	
									Erythema	3.7%			
									Granuloma	3.7%			
17	Duim (2015)[18]	x	-	-	-	Brazil	1,344	60-69	Pressure injury	30.6%	-	-	Authors use concept of "skin lesions"
								70-79		0%			
								≥80		27.2%			
18	Kiiski (2015)[19]	x	-	-	-	Finland	Unclear	≥ 70	AD	12-month prevalence: 5.6% Prevalence 'adulthood ≥70': 9.5%	-		-
19	Romani (2015)[20]	-	-	x	-	Fiji	Unclear	65, 70 (median)	Scabies	15, 20%	-	-	Age and prevalence data extracted from figure 5
						Vanuatu		70 (median)		10%			
						Brazil		70, 75 (median)		5%			
						Panama		65 (median)		10%			
						Solomon islands		70 (median)		0%			
						Timor-Leste		70 (median)		15%			
20	Cinotti (2016)[21]	x	-	-	-	France	209	77.5 (mean)	AK	69.4%	-	-	-
									Actinic lentigo	47.4%			Proportions recalculated based on table 2
									Cherry angioma	45.9%			
									SK	43.5%			
									Acrochordon	34.9%			
									Dermal naevus	33.5%			
									BCC	5.3%			
									Melanoma	1.0%			
									SCC	0.4%			
21	Hsieh (2016)[22]	-	-	x	-	Taiwan	17,004 cases (Eight years)	≥ 65	AK	-	7.2 to 10.6/10,000 people		Unclear reporting of data collection period.
									Skin malignant neoplasm after AK diagnosis		122.7/ 10,000 patient-year		

No.	First author (year)	Design				Geographic location	Sample characteristics		Results				Notes
		Cross-sec-tional	Longi-tudinal	Secondary data analysis/ review	Registry data		Sample size, n (Time period)	Age (years)	Skin condition	Prevalence/ Proportion	Incidence	Other	
22	Trautmann (2016)[23]	-	-	x	-	Germany	276,484 (Eight years)	60 to 69	Melanoma	0.2%	-	70.4% in people ≥ 60 years	Proportions recalculated based on table 2 and 3
							260,943 (Eight years)	70 to 79		0.4%			
							149,472 (Eight years)	≥80		0.5%			
							276,484 (Eight years)	60 to 69	NMSC	2.0%		88,3% in people ≥ 60 years	
							260,943 (Eight years)	70 to 79		4.3%			
							149,472 (Eight years)	≥80		6.9%			
23	Tseng (2016)[24]	-	-	x	-	Taiwan	18,318 (Diabetes mellitus cohort) (Five years)	≥ 60	Skin cancer (melanoma and non-melanoma)	-	6.8/10,000 person-years	-	-
							18,318 (Non-Diabetes mellitus cohort) (Five years)	≥ 60			4.7/10,000 person-years		
24	Asokan (2017)[25]	x	-	-	-	India	562	73.7 (mean)	SK / dermatosis papulosa nigra	55.2%	-	-	Mean age recalculated
									Ichthyosis/ xerosis	52.0%			
									Idiopathic guttate hypomelanosis	35.1%			
									Dermatophytosis	24.4%			
									Candidiasis	19.2%			
									Eczema/ dermatitis	12.8%			
									Skin tags	6.0%			
									Acanthosis nigricans	2.7%			
									Tinea versicolor	2.3%			
									Thick skin	2.0%			
									Vitiligo	1.8%			
									Waxy skin	0.7%			
									Impetigo	0.5%			
									Folliculitis	0.4%			
									Lichen planus	0.4%			
									Furuncle	0.2%			

No.	First author (year)	Design				Geographic location	Sample characteristics		Results				Notes
		Cross-sectional	Longi-tudinal	Secondary data analysis/ review	Registry data		Sample size, n (Time period)	Age (years)	Skin condition	Prevalence/ Proportion	Incidence	Other	
25	Barbaric (2017)[26]	-	-	-	x	Croatia	Unclear	60-64	Melanoma	-	M: 25/100,000<br>F: 18/100,000	-	Incidence rates extracted from figures 1a and 1b (semi-log plot)
								65-69			M: app. 40/100,000<br>F: app. 22/100,000		
								70-74			M: 50/100,000<br>F: 28/100,000		
								75-79			M: 45/100,000<br>F: 27/100,000		
26	George (2017)[27]	x	-	-	-	India	230	65 (mean)	Vitiligo	1.7%	-	-	Estimates recalculated
									Pediculosis	0.4%			
									Ichthyosis	2.6%			
27	Hahnel (2017)[28]	-	-	x	-	Iran	Unclear	≥ 65	Dermatomycosis	1% to 40% (home care)	-	-	-
						USA			Onychomycosis	6.1-8.8% (home care)	-		
						Brazil<br>Tunisia				4%-41.4% (medical practices)	-		
						Italy			Pressure injury	22.9% (home care)	6.7% (home care)		
						UK				0.3%-0.7% (medical practices)	-		
						France			Xerosis cutis	55.6% (medical practices)	-		
						UK<br>Tunisia			Eczema	6.5% to 12.5% (medical practices)	-		
						Sweden				1.6% to 30.6% (domesticity)	-		
						Tunisia			Benign skin tumors/cancer	10.7% (medical practices)	-		
						Sri Lanka				18.6% (domesticity)	-		

No.	First author (year)	Design				Geographic location	Sample characteristics		Results				Notes
		Cross-sec-tional	Longi-tudinal	Secondary data analysis/ review	Registry data		Sample size, n (Time period)	Age (years)	Skin condition	Prevalence/ Proportion	Incidence	Other	
						Japan Tunisia			Fungal (skin) infections	16.9%-64% (medical practices)	-		
						Sri Lanka				14.3% (domesticity)	-		
						Japan			Tinea pedis	12%-24% (medical practices)	-		
						USA				6.1% to 8.8% (home care)	-		
						Tunisia			Bacterial skin infections	8.7% (medical practices)	-		
									Viral skin infections	6.8% (medical practices)	-		
						Belgium			Tinea pedis + Onychomycosis	-	25.7% (medical practices)		
									Erysipelas	-	3-5/ 1,000 patient-years (medical practices)		
						USA			Mycoses	-	0.04% (domesticity)		
						Netherlands Germany			AK	4.7% to 24.2% (domesticity)	-		
28	Iizaka (2017)[29]	x	-	-	-	Japan	118	74.1 (mean)	Eczema	15.3%	-	-	-
									Herpes zoster	9.3%			
									Drug rush	5.1%			
									AD	0.9%			
29	Kim (2017)[30]	-	-	x	-	South Korea	4,617	≥60	Pressure injury	0.5%	-	-	-
30	Pandeya (2017)[31]	-	-	x	-	Australia	Unclear (Four years)	80 to 84	Keratinocyte cancers (BCC and SCC)	-	>6000 / 100,000 person-years	-	-
31	Thorslund (2017)[32]	-	-	-	x	Sweden	3,053 cases (Eight years)	≥ 70	BP	-	-	81.2% of cases were ≥70 years.	-
							2,062 cases (Eight years)	≥ 80			-	54.8% of cases were ≥80 years.	
							523 cases (Eight years)	90 to 99			81.9 /100,000	-	
32	Abuabara (2018)[33]	-	-	x	-	UK	Unclear (20 years)	≥ 75	AD	8%	-	-	

No.	First author (year)	Design				Geographic location	Sample characteristics		Results				Notes
		Cross-sectional	Longitudinal	Secondary data analysis/ review	Registry data		Sample size, n (Time period)	Age (years)	Skin condition	Prevalence/ Proportion	Incidence	Other	
33	Aitken (2018)[34]	-	-	-	x	Australia (Queensland)	38,271 cases (20 years)	≥ 60	Melanoma	-	(Year 2014) M: app. 200 to 500 /100,000 F: app. 100 to 200 /100,000	-	Rates extracted from figure 2 (log-scale)
34	Drewitz (2018)[35]	x	-	-	-	Germany	1,133	76.7 (mean)	Psoriasis	5.5%	--	-	Self-report of a previous diagnosis of psoriasis
							No data	80- 84		7.8%			
								90- 95		0%			
35	Dziunycz (2018)[36]	x	-	-	-	Switzerland	Unclear	60 to <70	AK	25%	-	-	Estimates extracted from figure 2
								70 to <80		45%			
								80 to <90		55%			
								90 to <100		69.4%			-
36	Hu (2018)[37]	-	-	-	x	Hong Kong/China	807 cases (32 years)	≥65	Melanoma	-	3.3 /100,000 person-years (age-standardised)	-	-
37	Lichterfeld-Kottner (2018)[38]	x	-	-	-	Germany	923	80.6 (mean)	Xerosis cutis	52.0%	-	Severity: Mild: 20.6 to 23.3% Severe: 2.0 to 6.5% Skin cracks: 1.3 to 2.5%	-
									Incontinence associated dermatitis	14.7%		Severity: Moderate: 10.2%	
									Pressure injury	3.6%		-	
									Ulcus cruris	2.4%			
									Diabetic foot syndrom	1.0%			
									Chronic wound due to pAVK	0.3%			
38	Sanders (2018)[39]	x	-	-	-	Netherlands	5,498	67.9 (median)	Seborrheic dermatitis	14.3%	-	-	-
39	Steglich (2018)[40]	-	-	x	-	Brazil	347 cases (12 years)	≥ 60	Melanoma	-	-	38.9% of cases were ≥ 60	Proportions recalculated based on table 2
40	Augustin (2019) [41]	x	-	-	-	Germany	4,525	60-70	Dry skin	36.4%	-	-	-

No.	First author (year)	Design				Geographic location	Sample characteristics		Results				Notes
		Cross-sectional	Longi-tudinal	Secondary data analysis/ review	Registry data		Sample size, n (Time period)	Age (years)	Skin condition	Prevalence/ Proportion	Incidence	Other	
-	Augustin (2019)[42]	x	-	-	-	Germany	Unclear	60-70	Dry skin	38.4%	-	-	Conference abstract
41	Drewitz (2019)[43]	x	-	-	-	Germany	1,133	76.7 (mean)	AD	3.3%	-	-	Self-report of a previous diagnosis
									Hand eczema	2.7%			
							No data	75-79	AD	2.4%			
								85-89		4.4%			
								80-84	Hand eczema	0.5%			
								90-95		3.9%			
42	Mekic (2019)[44]	x	-	-	-	Netherlands	5,547	70.0 (mean)	Xerosis cutis	60%	-	1 in 5 individuals with xerosis were affected severely	-
43	Sari (2019)[45]	x	-	-	-	Indonesia	325	72.1 (mean)	Pressure injury	All categories: 10.8%	-	-	
										Excluding category 1: 5.2%			
44	Tizek (2019)[46]	x	-	-	-	Germany	661	60 to 69	Any skin disease	74.4%	-	-	-
									AK	41.8%			
									Rosacea	33.7%			
									Keratinocyte carcinomas (including BCC and SCC)	5.4%			
									Eczema	10.6%			
									Psoriasis	1.2%			
									Acne	0.3%			
							225	≥ 70	Any skin disease	83.1%			
									AK	65.3%			
									Rosacea	32.9%			
									Eczema	8.0%			
									Keratinocyte carcinomas (including BCC and SCC)	6.7%			
									Psoriasis	0.9%			
									Acne	0%			
45	Venables (2019a)[47]	-	-	-	x	United Kingdom	211,992 cases (Two years)	≥ 60	BCC	-	F: 200 to 700 /100,000 person-years M: 300 to 1300 /100,000 person-years		Cases recalculated based on table 1

No.	First author (year)	Design				Geographic location	Sample characteristics		Results				Notes
		Cross-sectional	Longitudinal	Secondary data analysis/ review	Registry data		Sample size, n (Time period)	Age (years)	Skin condition	Prevalence/ Proportion	Incidence	Other	
							71,948 cases (Two years)	≥ 60	SCC	-	F: 20 to 500/100,000 person-years<br>M: 50 to 1000/100,000 person-years		Incidence extracted from figures 4a and b
46	Venables (2019b)[48]	-	-	-	x	England	71,948 cases (Two years)	≥ 60 years	SCC	-	-	78.8% of cases ≥ 70 years	6.1% of cases ≤ 59 years
							93,890 cases (Two years)	All ages			F: 34.1 /100,000 person-years (age-standardised)<br><br>M: 77.3 /100,000 person-years (age-standardised)	-	
47	Bianchi (2020)[49]	x	-	-	-	Brazil	12,614 skin lesions	60 to 79	Leuchoderma	2.8%	-	-	Proportions recalculated based on figure 1<br><br>Most frequent diseases of skin lesions assessed via telederma-tology consultations.
									Wart	1.9%			
									Xerosis	2.9%			
									Solar lentigo	6.6%			
									Benign neoplasm	4.9%			
									Epidermoid cyst	3.5%			
									Onychomycosis	6.4%			
									AK	4.0%			
									Melanocytic nevus	6.0%			
									SK	12.4%			
								≥80	Leuchoderma	0.7%			
									Wart	0.2%			
									Xerosis	0.4%			
									Solar lentigo	0.6%			
									Benign neoplasm	0.1%			
									Epidermoid cyst	0.2%			
									Onychomycosis	0.4%			
									AK	0.8%			
									Melanocytic nevus	0.3%			
									SK	1.5%			
								60 to 79	Benign tumors	54%			Proportions recalculated based on figure 2
									Pigmentary disorders	26%			
									Eczemas	23%			
									Precancerous/malign tumors	16%			

No.	First author (year)	Design				Geographic location	Sample characteristics		Results				Notes
		Cross-sectional	Longitudinal	Secondary data analysis/ review	Registry data		Sample size, n (Time period)	Age (years)	Skin condition	Prevalence/ Proportion	Incidence	Other	
									Infectious diseases	23%			Most frequent disease groups of skin lesions assessed via telederma-tology consultations.
								≥ 80	Benign tumors	47%			
									Pigmentary disorders	17%			
									Eczemas	27%			
									Precancerous/malign tumors	52%			
									Infectious diseases	18%			
48	Everink (2020)[50]	-	-	x	-	Netherlands	234	80.1 (mean)	Intertrigo	9.4%	-	-	Mean age also includes subjects from other settings
49	Fors (2020)[51]	x	-	-	-	Ecuador	92	> 65	AK	27.1%	-	-	Reviewers recalculated proportion based on table 1.
50	Kottner (2020)[52]	-	-	x	-	Netherlands	3,410	78.1 (mean) 82.0 (median)	Intertrigo	9.6%	-	-	Mean and median age also includes subjects from other settings
51	Prasad (2020)[53]	-	x	-	-	UAE	249	75.5	Pressure Injury	Prevalence rate: 9.0 to 12.0%	Incidence rate: 3.0 to 6.0%	-	Estimates extracted from text and figure 1
52	Sinikumpu (2020)[54]	x	-	-	-	Finland	552	78.4 (mean)	Androgenetic alopecia	83.0%	-	-	-
									SK	78.8%			
									Lentigo senilis	69.5%			
									Cherry angioma	63.2%			
									Melanocytic nevi	50.1%			
									Tinea pedis	48.6%			
									Onychomycosis	29.9%			
									Rosacea	25.0%			

No.	First author (year)	Design				Geographic location	Sample characteristics		Results				Notes
		Cross-sectional	Longi-tudinal	Secondary data analysis/ review	Registry data		Sample size, n (Time period)	Age (years)	Skin condition	Prevalence/ Proportion	Incidence	Other	
									Female pattern hair loss	22.5%			
									AK	22.3%			
									Asteatotic eczema	20.8%			
									Seborrheic dermatitis	10.1 %			
									Nummular eczema	9.2%			
									Moisture-associated skin damage	9.1%			
									Hand eczema	8.5%			
									BCC	5.1%			
									Verruca plantaris	3.8%			
									Folliculitis	3.6%			
									Lichen planus	2.5%			
									Verruca palmaris	2.4%			
									Vitiligo	2.0%			
									Bowen's disease	1.6%			
									AD	1.6%			
									Tinea corporis	1.5%			
									Psoriasis	1.1%			
									Pityriasis versicolor	1.1%			
									Alopecia areata	0.9%			
									Urticaria	0.7%			
									Contact eczema	0.7 %			
									Melanoma	0.5%			
									Hyperhidrosis	0.5%			
									SCC	0.4%			
									Hidradenitis suppurativa	0.2%			

No.	First author (year)	Design				Geographic location	Sample characteristics		Results				Notes
		Cross-sectional	Longi-tudinal	Secondary data analysis/ review	Registry data		Sample size, n (Time period)	Age (years)	Skin condition	Prevalence/ Proportion	Incidence	Other	
53	Tokez (2020)[55]	-	-	-	x	Netherlands	13,246 cases (28 years)	70 to 79	Cutaneous SCC (in situ)	-	M: 273.0 /100,000 person-years F: 299.9 /100,000 person-years (2017)	-	-
							9,529 cases (28 years)	≥ 80		-	M: 540.9 /100,000 person-years F: 482.7 /100,000 person-years (in 2017)	-	
54	Tseng (2020)[56]	-	-	x	-	Southern California/ USA	24,551 cases (Three months)	≥ 60	Herpes zoster	-	10.2 to 14.3/1000 person-years	-	-
55	Yew (2020)[57]	x	-	-	-	Singapore	419	60 to 74	Any skin disease in list (below)	19.3%			Self-reported<br>Proportions recalculated based on table 1
									Bacterial skin infections	8.6%			Self-reported
									Eczema	6.4%			
									Chronic ulcers	3.3%			
									Fungal skin infections	2.9%			
									Acne	1.7%			
									Viral warts	1.4%			
									Chronic urticaria	1.0%			
									Vitiligo	1.0%			
									Psoriasis	0.5%			
									Scabies	0.2%			
							188	≥ 75	Any skin disease in list (below)	30.3%			Self-reported<br>Proportions recalculated based on table 1
									Bacterial skin infections	13.8%			Self-reported
									Eczema	9.6%			
									Fungal skin infections	5.3%			
									Viral warts	2.1%			

No.	First author (year)	Design				Geographic location	Sample characteristics		Results				Notes
		Cross-sectional	Longi-tudinal	Secondary data analysis/ review	Registry data		Sample size, n (Time period)	Age (years)	Skin condition	Prevalence/ Proportion	Incidence	Other	
									Chronic ulcers	2.1%			
									Psoriasis	1.6%			
									Skin cancer	1.6%			
									Chronic urticaria	1.1%			
									Acne	0.5%			
									Vitiligo	0.5%			
									Scabies	0.5%			
									Alopecia areata	0.5%			
56	Bai (2021)[58]	-	-	x	-	China	Unclear	≥60	Melanoma	-	2 to 15/100,000 person-years		Incidence rate extracted from figure 3
57	Barbieri (2021)[59]	-	-	x	-	USA	Prevalent cases: 9,514 Incident cases: 5,978 (Two years)	≥60	Granuloma annulare	86.8/100 000 person-years<br><br>Prevalence, %: 0.09	54.5/100 000 person-years<br><br>Incidence, %: 0.05	-	-
58	Bucchi (2021)[60]	-	-	-	x	Italy	17,535 cases (20 years)	≥ 65	Melanoma	-	M: 40 to 50/100,000 F: 20 to 30/100,000 (2009-2013)	-	Incidence rate extracted from figure 3 (semi-log-scale)
59	Drewitz (2021)[61]	x	-	-	-	Germany	1,133	77.6 (mean)	Hand eczema	2.8 % (standardised frequency)	-		Self-report of previous diagnosis
60	Madani (2021)[62]	-	-	x	-	USA	140,198 (subjects with previous AK) (Ten years)	60 to 69	SCC	8.7%	-		Proportions recalculated based on tables 2 and 4
								70 to 79		12.9%			
								≥ 80		16.4%			
							140,198 (subjects without AK previous diagnosis) (Ten years)	60 to 69		3.3%			
								70 to 79		5.3%			
								≥ 80		6.5%			

No.	First author (year)	Design				Geographic location	Sample characteristics		Results				Notes
		Cross-sectional	Longi-tudinal	Secondary data analysis/ review	Registry data		Sample size, n (Time period)	Age (years)	Skin condition	Prevalence/ Proportion	Incidence	Other	
61	Memon (2021)[63]	-	-	-	x	England	22,235 cases (Two years)	≥ 65	Melanoma	-	F: 60.1 /100,000 population<br>M: 90.4 /100,000 population<br>(in 2016-2018)	-	-
62	Neena (2021)[64]	x	-	-	-	India	385	≥ 65	Eczema (not specified)	11.4%	-	-	-
									Asteatotic eczema	3.1%			
									Gravitational eczema	2.6%			
									Lichen simplex chronicus	2.3%			
									Allergic contact dermatitis	1.6%			
									AD	0.5%			
									Nummular eczema	0.5%			
									Hand eczema	0.3%			
									Infective eczema	0.3%			
63	Tang (2021)[65]	x	-	-	-	China/ Shanghai	2,912	61 to 70	Vitiligo	1.2%	-	-	Age-adjusted prevalence using the population of Shanghai from the 2010 Shanghai Population Census
								71 to 80		1.6%			
								≥81		1.2%			
64	Waldmann (2021)[66]	-	-	-	x	Germany	2,847 cases (Two years)	65 to 74	Melanoma	-	M: 72.4 /100,000 males/year (in 2015/2016)	-	-
							Unclear	≥ 65			F: 50 /100,000 females/year<br>M: 50 to 100 /100,000 males/year<br>(in 2015/2016)	-	Incidence extracted from figure 1

No.	First author (year)	Design				Geographic location	Sample characteristics		Results				Notes
		Cross-sec-tional	Longi-tudinal	Secondary data analysis/ review	Registry data		Sample size, n (Time period)	Age (years)	Skin condition	Prevalence/ Proportion	Incidence	Other	
65	Blazek (2022)[67]	-	-	-	x	New South Wales/ Australia	69,136 cases (26 years)	≥60	Melanoma	-	F: 100 to 150/100,000 M: 250 to 300/100,000	-	Incidence rates based on figure 1 (log-scale)
66	Botvid (2022)[68]	-	-	x	-	Greenland	37 cases	60-69	Psoriasis	1.4%	-	-	-
								70-79		2.0%			
								≥80		0.9%			
67	Choon (2022)[69]	-	-	x	-	Malaysia	Unclear (Ten years)	60-69	Psoriasis	0.7%	45.9 /100,000 person-years	-	-
								≥ 70		0.5%	39.9 /100,000 person-years		
68	Lu (2022)[70]	-	-	x	-	Global	Unclear	60-79	BP	-	0.004 to 0.007 /1000 person-years (age-specific incidence)	-	-
								≥80			0.011 to 0.017 /1000 person-years (age-specific incidence)		
69	Matsumoto (2022)[71]	-	x	-	-	USA	127,777 (unscreened cohort) (Four years)	≥ 65	Melanoma	-	66.5 /100,000 person-years (age-sex adjusted)	Incidence, thick melanoma (>2 mm): screened < unscreened	-
							47,603 (screened cohort) (Four years)			-	98.7 /100,000 person-years (age-sex adjusted)	Incidence, thin/in situ melanoma: screened > unscreened	
70	Navsaria (2022)[72]	-	-	x	-	USA	4,999,999 (Ten years)	≥ 65	AK	-	28,788 /100,000 person-years (age-adjusted) M: 42,970 /100,000 F: 20,492 /100,000 person-years	29.3% had ≥1 AK encounter	Estimates based on AK diagnosis encounters (incl. multiple specialities)
71	van Niekerk (2022)[73]	-	-	-	x	Netherlands	Unclear	60 to 69	Melanoma	-	F: 79.6 to 100.6 /100,000 person-years M: 84.4 to 110.9 /100,000 person-years (2014 to 2016)	-	-

No.	First author (year)	Design				Geographic location	Sample characteristics		Results				Notes
		Cross-sec-tional	Longi-tudinal	Secondary data analysis/ review	Registry data		Sample size, n (Time period)	Age (years)	Skin condition	Prevalence/ Proportion	Incidence	Other	
								70 to 79			F: 108.7 to 123.6 /100,000 person-years<br>M: 152.4 to 158.5 /100,000 person-years<br>(2014 to 2016)		
								≥ 80			F: 81.7 to 101.2 /100,000 person-years<br>M: 157.7 to 159.7 /100,000 person-years<br>(2014 to 2016)		
72	Radkiewicz (2022)[74]	-	-	-	x	Sweden	Unclear (44 years)	95 to 99	Melanoma	-	64.8 /100,000 person-years (peak)	-	-
73	Raghuwan-shi (2022)[75]	x	-	-	-	India	Unclear	60 to 74	Eczema	7.5%	-	-	-
									Acne	2.8%			
									Psoriasis	0.7%			
									Vitiligo	2.0%			
									Viral warts	1.4%			
									Scabies	0.3%			
									Fungal skin infections	3.1%			
									Chronic urticaria	2.0%			
									Bacterial skin infections	9.7%			
									Chronic ulcers	4.4%			
								≥ 75	Eczema	10.1%			
									Acne	0.6%			
									Psoriasis	2.7%			
									Vitiligo	0.6%			
									Viral warts	6.4%			
									Scabies	2.2%			
									Fungal skin infections	14.9%			

No.	First author (year)	Design				Geographic location	Sample characteristics		Results				Notes
		Cross-sectional	Longi-tudinal	Secondary data analysis/ review	Registry data		Sample size, n (Time period)	Age (years)	Skin condition	Prevalence/ Proportion	Incidence	Other	
									Chronic urticaria	3.2%			
									Skin cancer	1.8%			
									Alopecia areata	0.6%			
74	Rodriguez-Betancourt (2022)[76]	-	-	-	x	Colombia	42 cases (Nine years)	≥ 70	Melanoma	-	-	31.8% of cases in people ≥70 years	-
75	Huang (2023)[77]	x	-	-	-	Shanghai, China	2,038	60-64	NMSC (incl. AK)	1.5%	-	Prevalence rate 954.8 / 100,000	Prevalence rate standardised to Shanghai older population
								64-69		2.4 %			
								70–74		2.3 %		Prevalence rate 1355.1/ 100,000	
								75–79		5.9%			
								80–84		5.1%		Prevalence rate 1167.3 / 100,000	
								≥ 85		10.4%			
								60-64	AK	0.9%		-	-
								64-69		1.7%			
								70–74		1.9%			
								75–79		5.1%			
								80–84		4.4%			
								≥ 85		7.5%			
76	Keim (2023)[78]	-	-	-	x	Germany	Unclear	≥80	SCC	-	M: 600 to 847 /100,000 persons per year F: 278 to 285 /100,000 persons per year (in 2019/2020)	-	-
						Netherlands					M: 966 /100,000 persons per year F: 560 /100,000 persons per year (in 2021)		
						Scotland					M: 1107 /100,000 persons per year F: 346 /100,000 persons per year (in 2017)		
77	Xu (2023)[79]	-	-	-	x	China/ Hong Kong	Unclear	60 to 79	NMSC	-	F: 30 to 150 M: 50 to 230		Rate ratios are age-specific rates in reference cohort adjusted for

No.	First author (year)	Design				Geographic location	Sample characteristics		Results				Notes
		Cross-sectional	Longi-tudinal	Secondary data analysis/ review	Registry data		Sample size, n (Time period)	Age (years)	Skin condition	Prevalence/ Proportion	Incidence	Other	
								≥80			F:150 to 310<br>M: 230 to 400		period effects<br><br>Rates extracted from figure 3

Abbreviations:

Seborrhoeic keratosis (SK)
Actinic keratosis (AK)
Atopic dermatitis (AD)
Non-melanoma skin cancer (NMSC)
Melanoma (malignant melanoma, cutaneous melanoma…)
Bullous pemphigoid (BP)
(cutaneous) Squamous cell carcinoma (SCC)
Basal cell carcinoma (BCC)

Notes:

All proportions and numbers extracted from figures are estimates
'Skin cancer' = not specified

Table S5: Summary of focus of included studies for review question 1

**Author (year)**	**Any skin condition assessed**	**Selection of skin conditions /diseases assessed**	**One skin condition /disease (or group) assessed**	**Melanoma**	**NMSC**	**PI**	**Eczema (incl. AD)**	**Xerosis cutis /Ichthyosis**	**Psoriasis**	**BP**	**Intertrigo**	**Seborrheic dermatitis**	**Vitiligo**	**Granuloma annulare**	**Parasites (Scabies, Pediculosis)**	**Herpes Zoster**	**MRSA**
Akbari (2011)[1]	-	-	x	X?*	X?*	-	-	-	-	-	-	-	-	-	-	-	-
Augustin (2011)[2]	x	-	-	-	-	-	-	-	-	-	-	-	-	-	-	-	-
Paul (2011)[3]	-	-	x	-	-	-	-	x	-	-	-	-	-	-	-	-	-
Ritchie (2011)[4]	-	-	x	-	-	-	-	-	-	-	-	-	-	-	-	-	x
Wu (2011)[5,6]	-	-	x	-	x	-	-	-	-	-	-	-	-	-	-	-	-
Hollestein (2012)[7]	-	-	x	x	-	-	-	-	-	-	-	-	-	-	-	-	-
Joly (2012)[8]	-	-	x	-	-	-	-	-	-	x	-	-	-	-	-	-	-
Bonaccorsi (2013)[9]	-	-	x	-	-	x	-	-	-	-	-	-	-	-	-	-	-
Danielsen (2013)[10]	-	-	x	-	-	-	-	-	x	-	-	-	-	-	-	-	-
Etzkorn (2013)[11]	-	x	-	x	x	-	-	-	-	-	-	-	-	-	-	-	-
Flohil (2013)[12]	-	-	x	-	x	-	-	-	-	-	-	-	-	-	-	-	-
Robsahm (2013)[13]	-	-	x	x	-	-	-	-	-	-	-	-	-	-	-	-	-
Gontijo-Guerra (2014a)[14]	x	-	-	-	-	-	-	-	-	-	-	-	-	-	-	-	-
Hsieh (2014)[15]	-	-	x	-	x	-	-	-	-	-	-	-	-	-	-	-	-
Caretti (2015)[16]	x	-	-	-	-	-	-	-	-	-	-	-	-	-	-	-	-
Cybulski (2015)[17]	x	-	-	-	-	-	-	-	-	-	-	-	-	-	-	-	-
Duim (2015)[18]	-	-	x	-	-	x	-	-	-	-	-	-	-	-	-	-	-
Kiiski (2015)[19]	-	-	x	-	-	-	x	-	-	-	-	-	-	-	-	-	-
Romani (2015)[20]	-	-	x	-	-	-	-	-	-	-	-	-	-	-	x	-	-
Cinotti (2016)[21]	-	x	-	x	x	-	-	-	-	-	-	-	-	-	-	-	-
Hsieh (2016)[22]	-	-	x	-	x	-	-	-	-	-	-	-	-	-	-	-	-
Trautmann (2016)[23]	-	x	-	x	x	-	-	-	-	-	-	-	-	-	-	-	-
Tseng (2016)[24]	-	-	x	x	x	-	-	-	-	-	-	-	-	-	-	-	-

Author (year)	Any skin condition assessed	Selection of skin conditions /diseases assessed	One skin condition /disease (or group) assessed	Melanoma	NMSC	PI	Eczema (incl. AD)	Xerosis cutis /Ichthyosis	Psoria-sis	BP	Intertrigo	Seborrheic dermatitis	Vitiligo	Granuloma annulare	Parasites (Scabies, Pediculosis)	Herpes Zoster	MRSA
Asokan (2017)[25]	x	-	-	-	-	-	-	-	-	-	-	-	-	-	-	-	-
Barbaric (2017)[26]	-	-	x	x	-	-	-	-	-	-	-	-	-	-	-	-	-
George (2017)[27]	-	x	-	-	-	-	-	x	-	-	-	-	x	-	x	-	-
Hahnel (2017)[28]	x	-	-	-	-	-	-	-	-	-	-	-	-	-	-	-	-
Iizaka (2017)[29]	-	x	-	-	-	-	x	-	-	-	-	-	-	-	-	x	-
Kim (2017)[30]	-	-	x	-	-	x	-	-	-	-	-	-	-	-	-	-	-
Pandeya (2017)[31]	-	-	x	-	x	-	-	-	-	-	-	-	-	-	-	-	-
Thorslund (2017)[32]	-	-	x	-	-	-	-	-	-	x	-	-	-	-	-	-	-
Abuabara (2018)[33]	-	-	x	-	-	-	x	-	-	-	-	-	-	-	-	-	-
Aitken (2018)[34]	-	-	x	x	-	-	-	-	-	-	-	-	-	-	-	-	-
Drewitz (2018)[35]	-	-	x	-	-	-	-	-	x	-	-	-	-	-	-	-	-
Dziunycz (2018)[36]	-	-	x	-	x	-	-	-	-	-	-	-	-	-	-	-	-
Hu (2018)[37]	-	-	x	x	-	-	-	-	-	-	-	-	-	-	-	-	-
Lichterfeld-Kottner (2018)[38]	-	x	-	-	-	x	-	x	-	-	-	-	-	-	-	-	-
Sanders (2018)[39]	-	-	x	-	-	-	-	-	-	-	-	x	-	-	-	-	-
Steglich (2018)[40]	-	-	x	x	-	-	-	-	-	-	-	-	-	-	-	-	-
Augustin (2019a,b)[41, 42]	-	-	x	-	-	-	-	x	-	-	-	-	-	-	-	-	-
Drewitz (2019)[43]	-	-	x	-	-	-	x	-	-	-	-	-	-	-	-	-	-
Mekic (2019)[44]	-	-	x	-	-	-	-	x	-	-	-	-	-	-	-	-	-
Sari (2019)[45]	-	-	x	-	-	x	-	-	-	-	-	-	-	-	-	-	-
Tizek (2019)[46]	x	-	-	-	-	-	-	-	-	-	-	-	-	-	-	-	-
Venables (2019a)[47]	-	-	x	-	x	-	-	-	-	-	-	-	-	-	-	-	-
Venables (2019b)[48]	-	-	x	-	x	-	-	-	-	-	-	-	-	-	-	-	-
Bianchi (2020)[49]	x	-	-	-	-	-	-	-	-	-	-	-	-	-	-	-	-

Author (year)	Any skin condition assessed	Selection of skin conditions /diseases assessed	One skin condition /disease (or group) assessed	Melanoma	NMSC	PI	Eczema (incl. AD)	Xerosis cutis /Ichthyosis	Psoria-sis	BP	Intertrigo	Seborheic dermatitis	Vitiligo	Granuloma annulare	Parasites (Scabies, Pediculosis)	Herpes Zoster	MRSA
Everink (2020)[50]	-	-	x	-	-	-	-	-	-	-	x	-	-	-	-	-	-
Fors (2020)[51]	-	-	x	-	x	-	-	-	-	-	-	-	-	-	-	-	-
Kottner (2020)[52]	-	-	x	-	-	-	-	-	-	-	x	-	-	-	-	-	-
Prasad (2020)[53]	-	-	x	-	-	x	-	-	-	-	-	-	-	-	-	-	-
Sinikumpu (2020)[54]	x	-	-	-	-	-	-	-	-	-	-	-	-	-	-	-	-
Tokez (2020)[55]	-	-	x	-	x	-	-	-	-	-	-	-	-	-	-	-	-
Tseng (2020)[56]	-	-	x	-	-	-	-	-	-	-	-	-	-	-	-	x	-
Yew (2020)[57]	-	x	-	X?*	x?*	-	x	-	x	-	-	-	x	-	x	-	-
Bai (2021)[58]	-	-	x	x	-	-	-	-	-	-	-	-	-	-	-	-	-
Barbieri (2021)[59]	-	-	x	-	-	-	-	-	-	-	-	-	-	x	-	-	-
Bucchi (2021)[60]	-	-	x	x	-	-	-	-	-	-	-	-	-	-	-	-	-
Drewitz (2021)[61]	-	-	x	-	-	-	x	-	-	-	-	-	-	-	-	-	-
Madani (2021)[62]	-	-	x	-	x	-	-	-	-	-	-	-	-	-	-	-	-
Memon (2021)[63]	-	-	x	x	-	-	-	-	-	-	-	-	-	-	-	-	-
Neena (2021)[64]	-	-	x	-	-	-	x	-	-	-	-	-	-	-	-	-	-
Tang (2021)[65]	-	-	x	-	-	-	-	-	-	-	-	-	x	-	-	-	-
Waldmann (2021)[66]	-	-	x	x	-	-	-	-	-	-	-	-	-	-	-	-	-
Blazek (2022)[67]	-	-	x	x	-	-	-	-	-	-	-	-	-	-	-	-	-
Botvid (2022)[68]	-	-	x	-	-	-	-	-	x	-	-	-	-	-	-	-	-
Choon (2022)[69]	-	-	x	-	-	-	-	-	x	-	-	-	-	-	-	-	-
Lu (2022)[70]	-	-	x	-	-	-	-	-	-	x	-	-	-	-	-	-	-
Matsumoto (2022)[71]	-	-	x	x	-	-	-	-	-	-	-	-	-	-	-	-	-
Navsaria (2022)[72]	-	-	x	-	x	-	-	-	-	-	-	-	-	-	-	-	-
van Niekerk (2022)[73]	-	-	x	x	-	-	-	-	-	-	-	-	-	-	-	-	-
Radkiewicz (2022)[74]	-	-	x	x	-	-	-	-	-	-	-	-	-	-	-	-	-

Author (year)	Any skin condition assessed	Selection of skin conditions /diseases assessed	One skin condition /disease (or group) assessed	Melanoma	NMSC	PI	Eczema (incl. AD)	Xerosis cutis /Ichthyosis	Psoria-sis	BP	Intertrigo	Seborheic dermatitis	Vitiligo	Granuloma annulare	Parasites (Scabies, Pediculosis)	Herpes Zoster	MRSA
Raghuwanshi (2022)[75]	-	x	-	X?*	X?*	-	x	-	x	-	-	-	x	-	x	-	-
Rodriguez-Betancourt (2022) [76]	-	-	x	x	-	-	-	-	-	-	-	-	-	-	-	-	-
Huang (2023)[77]	-	-	x	-	x	-	-	-	-	-	-	-	-	-	-	-	-
Keim (2023)[78]	-	-	x	-	x	-	-	-	-	-	-	-	-	-	-	-	-
Xu (2023)[79]	-	-	x	-	x	-	-	-	-	-	-	-	-	-	-	-	-
**Total**	9	8	60	22 (19)	22 (19)	6	8	5	6	3	2	1	4	1	4	2	1

*Skin cancer not specified/defined

REFERENCES

1. Akbari ME, Rafiee M, Khoei MAet al. Incidence and survival of cancers in the elderly population in Iran: 2001-2005. *Asian Pac J Cancer Prev* 2011; **12**:3035-9.
2. Augustin M, Herberger K, Hintzen Set al. Prevalence of skin lesions and need for treatment in a cohort of 90 880 workers. *Br J Dermatol* 2011; **165**:865-73.
3. Paul C, Maumus-Robert S, Mazereeuw-Hautier Jet al. Prevalence and risk factors for xerosis in the elderly: a cross-sectional epidemiological study in primary care. *Dermatology* 2011; **223**:260-5.
4. Ritchie SR, Fraser JD, Libby Eet al. Demographic variation in community-based MRSA skin and soft tissue infection in Auckland, New Zealand. *New Zealand Medical Journal* 2011; **124**.
5. Wu J, Guo Z, Berman Ret al. P2-119: Occurrence of nonmelanoma skin cancer in the elderly with and without Alzheimer's disease in the US. 2011. In: Abstracts [Internet]. Alzheimer's & Dementia; [S347].
6. Wu J, Guo Z, Berman Ret al. 698. Risk of Non-Melanoma Skin Cancer in Elderly Patients with Alzheimer’s Disease. 2011. In: Abstracts of the 27th ICPE 2011 [Internet]. Pharmacoepidemiology and Drug Safety; [S303-4].
7. Hollestein LM, van den Akker SA, Nijsten Tet al. Trends of cutaneous melanoma in The Netherlands: increasing incidence rates among all Breslow thickness categories and rising mortality rates since 1989. *Ann Oncol* 2012; **23**:524-30.
8. Joly P, Baricault S, Sparsa Aet al. Incidence and mortality of bullous pemphigoid in France. *J Invest Dermatol* 2012; **132**:1998-2004.
9. Bonaccorsi G, Lorini C, Santomauro Fet al. 202 Impact of different pads in elderly assisted in home care. In: *International Continence Society (ICS), 2013*: Journal of Neurology and Urodynamics 2013: p. 802-3.
10. Danielsen K, Olsen AO, Wilsgaard T, Furberg AS. Is the prevalence of psoriasis increasing? A 30-year follow-up of a population-based cohort. *Br J Dermatol* 2013; **168**:1303-10.
11. Etzkorn JR, Parikh RP, Marzban SSet al. Identifying risk factors using a skin cancer screening program. *Cancer Control* 2013; **20**:248-54.
12. Flohil SC, van der Leest RJ, Dowlatshahi EAet al. Prevalence of actinic keratosis and its risk factors in the general population: the Rotterdam Study. *J Invest Dermatol* 2013; **133**:1971-8.
13. Robsahm TE, Bergva G, Hestvik UE, Moller B. Sex differences in rising trends of cutaneous malignant melanoma in Norway, 1954-2008. *Melanoma Res* 2013; **23**:70-8.
14. Gontijo Guerra S, Vasiliadis HM, Preville M, Berbiche D. Skin conditions in community-living older adults: prevalence and characteristics of medical care service use. *J Cutan Med Surg* 2014; **18**:186-94.
15. Hsieh C-F, Huang W-F, Chiang Y-T. 157. The Incidence of Actinic Keratosis and Risk of Non-Melanoma Skin Cancer in Taiwan. 2014. In: Pharmacoepidemiology and Drug Safety [Internet]. [84-5].
16. Caretti KL, Mehregan DR, Mehregan DA. A survey of self-reported skin disease in the elderly African-American population. *Int J Dermatol* 2015; **54**:1034-8.
17. Cybulski M, Krajewska-Kulak E. Skin diseases among elderly inhabitants of Bialystok, Poland. *Clin Interv Aging* 2015; **10**:1937-43.
18. Duim E, Sa FH, Duarte YAet al. Prevalence and characteristics of lesions in elderly people living in the community. *Rev Esc Enferm USP* 2015; **49 Spec No**:51-7.
19. Kiiski V, Susitaival P, Remitz A, Reitamo S. 086 Is atopic dermatitis more persistent than previously estimated? 2015. In: Auto-immunity, allergy and Inflammation | Abstracts [Internet]. Journal of Investigative Dermatology; [S15].
20. Romani L, Steer AC, Whitfeld MJ, Kaldor JM. Prevalence of scabies and impetigo worldwide: a systematic review. *Lancet Infect Dis* 2015; **15**:960-7.
21. Cinotti E, Perrot JL, Labeille Bet al. Skin tumours and skin aging in 209 French elderly people: the PROOF study. *Eur J Dermatol* 2016; **26**:470-6.
22. Hsieh C-F, Chiang Y-T, Chiu H-Y, Huang W-F. A Nationwide Cohort Study of Actinic Keratosis in Taiwan*. *International Journal of Gerontology* 2016; **10**:218-22.
23. Trautmann F, Meier F, Seidler A, Schmitt J. Effects of the German skin cancer screening programme on melanoma incidence and indicators of disease severity. *Br J Dermatol* 2016; **175**:912-9.
24. Tseng HW, Shiue YL, Tsai KWet al. Risk of skin cancer in patients with diabetes mellitus: A nationwide retrospective cohort study in Taiwan. *Medicine (Baltimore)* 2016; **95**:e4070.
25. Asokan N, Binesh VG. Cutaneous problems in elderly diabetics: A population-based comparative cross-sectional survey. *Indian J Dermatol Venereol Leprol* 2017; **83**:205-11.

26. Barbaric J, Laversanne M, Znaor A. Malignant melanoma incidence trends in a Mediterranean population following socioeconomic transition and war: results of age-period-cohort analysis in Croatia, 1989-2013. *Melanoma Res* 2017; **27**:498-502.
27. George LS, Deshpande S, Krishna Kumar MK, Patil RS. Morbidity pattern and its sociodemographic determinants among elderly population of Raichur district, Karnataka, India. *J Family Med Prim Care* 2017; **6**:340-4.
28. Hahnel E, Lichterfeld A, Blume-Peytavi U, Kottner J. The epidemiology of skin conditions in the aged: A systematic review. *J Tissue Viability* 2017; **26**:20-8.
29. Iizaka S, Nagata S, Sanada H. Nutritional Status and Habitual Dietary Intake Are Associated with Frail Skin Conditions in Community-Dwelling Older People. *J Nutr Health Aging* 2017; **21**:137-46.
30. Kim J, Choi Y, Shin Jet al. Incidence of Pressure Ulcers During Home and Institutional Care Among Long-Term Care Insurance Beneficiaries With Dementia Using the Korean Elderly Cohort. *J Am Med Dir Assoc* 2017; **18**:638 e1-e5.
31. Pandeya N, Olsen CM, Whiteman DC. The incidence and multiplicity rates of keratinocyte cancers in Australia. *Med J Aust* 2017; **207**:339-43.
32. Thorslund K, Seifert O, Nilzen K, Gronhagen C. Incidence of bullous pemphigoid in Sweden 2005-2012: a nationwide population-based cohort study of 3761 patients. *Arch Dermatol Res* 2017; **309**:721-7.
33. Abuabara K, Magyari A, Margolis DJ, Langan M. The prevalence of atopic eczema across the lifespan: a U.K. population-based cohort study. 2018. In: Abstracts of the 10th George Rajka International Symposium on Atopic Dermatitis [Internet]. British Journal of Dermatology; [e58].
34. Aitken JF, Youlden DR, Baade PDet al. Generational shift in melanoma incidence and mortality in Queensland, Australia, 1995-2014. *Int J Cancer* 2018; **142**:1528-35.
35. Drewitz KP, Stark K, Zimmermann MEet al. P102 | Prevalence and determinants of Psoriasis in a cross-sectional study of the elderly—results from the German AugUR study. 2018. In: 45th Annual Meeting of the Arbeitsgemeinschaft Dermatologische Forschung (ADF) [Internet]. Experimental Dermatology; [e43-4].
36. Dziunycz PJ, Schuller E, Hofbauer GFL. Prevalence of Actinic Keratosis in Patients Attending General Practitioners in Switzerland. *Dermatology* 2018; **234**:214-9.
37. Hu L, Jin S, Chen L, Wang Y. Trends in the incidence and mortality of cutaneous

melanoma in Hong Kong between 1983 and 2015. *Int J Clin Exp Med* 2018; **11**:8259-66.
38. Lichterfeld-Kottner A, Lahmann N, Blume-Peytavi Uet al. Dry skin in home care: A representative prevalence study. *J Tissue Viability* 2018; **27**:226-31.
39. Sanders MGH, Pardo LM, Franco OHet al. Prevalence and determinants of seborrhoeic dermatitis in a middle-aged and elderly population: the Rotterdam Study. *Br J Dermatol* 2018; **178**:148-53.
40. Steglich RB, Coelho K, Cardoso Set al. Epidemiological and histopathological aspects of primary cutaneous melanoma in residents of Joinville, 2003-2014. *An Bras Dermatol* 2018; **93**:45-53.
41. Augustin M, Kirsten N, Korber Aet al. Prevalence, predictors and comorbidity of dry skin in the general population. *J Eur Acad Dermatol Venereol* 2019; **33**:147-50.
42. Augustin M, Kirsten N, Koerber Aet al. Epidemiology of dry skin in the general population. 24th World Congress of Dermatology; 10-15 June 2019; Milan2019.
43. Drewitz KP, Stark K, Zimmermann MEet al. P086 | Frequency and comorbidities of eczema in an elderly population in Germany: results from augur. 2019. In: 46th Annual Meeting of the Arbeitsgemeinschaft Dermatologische Forschung (ADF) [Internet]. Experimental Dermatology; [e41-2].
44. Mekic S, Jacobs LC, Gunn DAet al. Prevalence and determinants for xerosis cutis in the middle-aged and elderly population: A cross-sectional study. *J Am Acad Dermatol* 2019; **81**:963-9 e2.
45. Sari SP, Everink IH, Sari EAet al. The prevalence of pressure ulcers in community-dwelling older adults: A study in an Indonesian city. *Int Wound J* 2019; **16**:534-41.
46. Tizek L, Schielein MC, Seifert Fet al. Skin diseases are more common than we think: screening results of an unreferred population at the Munich Oktoberfest. *J Eur Acad Dermatol Venereol* 2019; **33**:1421-8.
47. Venables ZC, Nijsten T, Wong KFet al. Epidemiology of basal and cutaneous squamous cell carcinoma in the U.K. 2013-15: a cohort study. *Br J Dermatol* 2019; **181**:474-82.
48. Venables ZC, Autier P, Nijsten Tet al. Nationwide Incidence of Metastatic Cutaneous Squamous Cell Carcinoma in England. *JAMA Dermatol* 2019; **155**:298-306.
49. Bianchi M, Santos A, Cordioli E. Benefits of Teledermatology for Geriatric Patients: Population-Based Cross-Sectional Study. *J Med Internet Res* 2020; **22**:e16700.

50. Everink IHJ, Kottner J, van Haastregt JCMet al. Skin areas, clinical severity, duration and risk factors of intertrigo: A secondary data analysis. *J Tissue Viability* 2020; **30**:102-7.
51. Fors M, Gonzalez P, Viada Cet al. Actinic keratoses in subjects from la Mitad del Mundo, Ecuador. *BMC Dermatol* 2020; **20**:11.
52. Kottner J, Everink I, van Haastregt Jet al. Prevalence of intertrigo and associated factors: A secondary data analysis of four annual multicentre prevalence studies in the Netherlands. *Int J Nurs Stud* 2020; **104**:103437.
53. Prasad S, Hussain N, Sharma Set al. Impact of Pressure Injury Prevention Protocol in Home Care Services on the Prevalence of Pressure Injuries in the Dubai Community. *Dubai Medical Journal* 2020; **3**:99-104.
54. Sinikumpu SP, Jokelainen J, Haarala AKet al. The High Prevalence of Skin Diseases in Adults Aged 70 and Older. *J Am Geriatr Soc* 2020; **68**:2565-71.
55. Tokez S, Wakkee M, Louwman Met al. Assessment of Cutaneous Squamous Cell Carcinoma (cSCC) In situ Incidence and the Risk of Developing Invasive cSCC in Patients With Prior cSCC In situ vs the General Population in the Netherlands, 1989-2017. *JAMA Dermatol* 2020; **156**:973-81.
56. Tseng HF, Bruxvoort K, Ackerson Bet al. The Epidemiology of Herpes Zoster in Immunocompetent, Unvaccinated Adults >/=50 Years Old: Incidence, Complications, Hospitalization, Mortality, and Recurrence. *J Infect Dis* 2020; **222**:798-806.
57. Yew YW, Kuan AHY, Ge Let al. Psychosocial impact of skin diseases: A population-based study. *PLoS One* 2020; **15**:e0244765.
58. Bai R, Huang H, Li M, Chu M. Temporal Trends in the Incidence and Mortality of Skin Malignant Melanoma in China from 1990 to 2019. *J Oncol* 2021; **2021**:9989824.
59. Barbieri JS, Rodriguez O, Rosenbach M, Margolis D. Incidence and Prevalence of Granuloma Annulare in the United States. *JAMA Dermatol* 2021; **157**:824-30.
60. Bucchi L, Mancini S, Crocetti Eet al. Mid-term trends and recent birth-cohort-dependent changes in incidence rates of cutaneous malignant melanoma in Italy. *Int J Cancer* 2021; **148**:835-44.
61. Drewitz KP, Stark KJ, Zimmermann MEet al. Frequency of hand eczema in the elderly: Cross-sectional findings from the German AugUR study. *Contact Dermatitis* 2021; **85**:489-93.
62. Madani S, Marwaha S, Dusendang JRet al. Ten-Year Follow-up of Persons With Sun-Damaged Skin Associated With Subsequent Development of Cutaneous Squamous Cell Carcinoma. *JAMA Dermatol* 2021; **157**:559-65.
63. Memon A, Bannister P, Rogers Iet al. Changing epidemiology and age-specific incidence of cutaneous malignant melanoma in England: An analysis of the national cancer registration data by age, gender and anatomical site, 1981-2018. *Lancet Reg Health Eur* 2021; **2**:100024.
64. Neena V, Asokan N, Jose R, Sarin A. Prevalence of eczema among older persons: A population-based cross-sectional study. *Indian J Dermatol Venereol Leprol* 2021; **89**:426-30.
65. Tang L, Li F, Xu Fet al. Prevalence of vitiligo and associated comorbidities in adults in Shanghai, China: a community-based, cross-sectional survey. *Ann Palliat Med* 2021; **10**:8103-11.
66. Waldmann A, Pritzkuleit R, Labohm L, Katalinic A. Epidemiologie von Krebs im hohen Lebensalter. *best practice onkologie* 2021; **16**:586-97.
67. Blazek K, Furestad E, Ryan Det al. The impact of skin cancer prevention efforts in New South Wales, Australia: Generational trends in melanoma incidence and mortality. *Cancer Epidemiol* 2022; **81**:102263.
68. Botvid SHC, Storgaard Hove L, Backe MBet al. Low prevalence of patients diagnosed with psoriasis in Nuuk: a call for increased awareness of chronic skin disease in Greenland. *Int J Circumpolar Health* 2022; **81**:2068111.
69. Choon SE, Wright AK, Griffiths CEMet al. Incidence and prevalence of psoriasis in multiethnic Johor Bahru, Malaysia: a population-based cohort study using electronic health data routinely captured in the Teleprimary Care (TPC(R)) clinical information system from 2010 to 2020: Classification: Epidemiology. *Br J Dermatol* 2022; **187**:713-21.
70. Lu L, Chen L, Xu Y, Liu A. Global incidence and prevalence of bullous pemphigoid: A systematic review and meta-analysis. *J Cosmet Dermatol* 2022; **21**:4818-35.
71. Matsumoto M, Wack S, Weinstock MAet al. Five-Year Outcomes of a Melanoma Screening Initiative in a Large Health Care System. *JAMA Dermatol* 2022; **158**:504-12.
72. Navsaria L, Li Y, Nowakowska Met al. LB911Incidence and treatments of actinic keratosis in the Medicare population: A cohort study. 2022. In: ABSTRACTS | Clinical Research — Epidemiology and Observational Research [Internet]. Journal of Investigative Dermatology; [B10].
73. van Niekerk CC, Otten J, van Rossum MMet al. Trends in three major histological subtypes of cutaneous melanoma in the Netherlands between 1989 and 2016. *Int J Dermatol* 2023; **62**:508-13.

74. Radkiewicz C, Jarkvik Kronmark J, Adami HO, Edgren G. Declining Cancer Incidence in the Elderly: Decreasing Diagnostic Intensity or Biology? *Cancer Epidemiol Biomarkers Prev* 2022; **31**:280-6.
75. Raghuwanshi AS, Diwan S, Singh H, Raghuwanshi KC. A Cross-Sectional Study to Assess the Psychosocial impact of Skin Diseases. *International Journal of Pharmaceutical and Clinical Research* 2022; **14**:1061-7.
76. Rodriguez-Betancourt JD, Arias-Ortiz N. Cutaneous melanoma incidence, mortality, and survival in Manizales, Colombia: a population-based study. *J Int Med Res* 2022; **50**:3000605221106706.
77. Huang J, Zhang L, Shi Let al. An epidemiological study on skin tumors of the elderly in a community in Shanghai, China. *Sci Rep* 2023; **13**:4441.
78. Keim U, Katalinic A, Holleczek Bet al. Incidence, mortality and trends of cutaneous squamous cell carcinoma in Germany, the Netherlands, and Scotland. *Eur J Cancer* 2023; **183**:60-8.
79. Xu Q, Wang X, Bai Yet al. Trends of non-melanoma skin cancer incidence in Hong Kong and projection up to 2030 based on changing demographics. *Ann Med* 2023; **55**:146-54.
