# Supplementary material for: Skin health of community-living older people: a scoping review
Source: Arch Dermatol Res. 2024 Jun 1;316(6):319. doi: 10.1007/s00403-024-03059-0 (PMC11144137; doi:10.1007/s00403-024-03059-0)
Supplement: Supplementary file 5 — Supplementary Material 5 [file 403_2024_3059_MOESM5_ESM.pdf]

Supplementary material 5

Article title: Skin health of community-living older people: a scoping review

Authors: Jan Kottner[1], Alexandra Fastner[1], Dimitra-Aikaterini Lintzeri[2], Ulrike Blume-Peytavi[2], Christopher E. M. Griffiths[3,4]

Institutions

[1]Institute of Clinical Nursing Science, Charité Universitätsmedizin Berlin, Berlin, Germany.

[2]Department of Dermatology, Venerology and Allergology, Charité Universitätsmedizin Berlin, Berlin, Germany.

[3]Department of Dermatology, King's College Hospital, King's College London, London, UK.

[4]Centre for Dermatology Research, NIHR Manchester Biomedical Research Centre, The University of Manchester, Manchester, UK.

Corresponding author Jan Kottner

Charité – Universitätsmedizin Berlin,

Institute of Clinical Nursing Science,

Charitéplatz 1,

10117 Berlin, Germany

Email: jan.kottner@charite.de

Table S6: Summary of included studies for review question 2

**No.**	**First author (year)**	**Design**				**Geographic location**	**Sample characteristics**		**Results**					**Notes**
		Cross-sectional	Longi-tudinal	Secondary data analysis	Registry data		Sample size, n (Time period)	Age (years)	Condition	Self-reported prevalence /proportion	Mortality	DALYs lost per 100,000	Other	
1	Frese (2011)[1]	x	-	-	-	Germany	Unclear	65 to 74	Pruritus	0.5%	-	-	-	Estimates based on consultations
								> 75		1.0%				
								65 to 74		3.7%				
								> 75		3.4%				
								65 to 74		1.9%				
								> 75		1.8%				
2	Hollestein (2012)[2]	-	-	-	x	Netherlands	Unclear	≥ 65	Melanoma	-	Year 2008: F: 11/100,000 person-years M: 18/100,000 person-years (European Standardised rate)	-	-	Data extracted from figure 3
3	Joly (2012)[3]	-	x	-	-	France	312 (3 years)	≥ 70 years	Bullous pemphigoid	-	Standardised mortality rate 6.5	-	-	-
4	Wysong (2013)[4]	-	-	x	-	USA	Unclear	> 65	NMSC	-	-	-	Physician visits in the year 2007; M: 160 /1,000 persons F: 60 /1,000 persons (fitted population-adjusted rate)	-
5	Gontijo-Guerra (2014a)[5]	-	x	-	-	Canada	569 (T1), 411 (T2) (1 year)	73.8 (mean)	Participants with physician-diagnosed skin condition	-	-	-	Average number of dermatology-related medical visits: 2.37 to 2.52 per year<br><br>31.9 % visited GP exclusively	Time period of one year assumed
6	Gontijo-Guerra (2014b)[6]	-	x	-	-	Canada	2,494 (T1), 1,985(T2)	73.8 (mean)	Depressive disorder in persons **with** a “skin condition”	T1:10.5% T2: 10.8 %	-	-	-	-

No.	First author (year)	Design				Geographic location	Sample characteristics		Results					Notes
		Cross-sectional	Longi-tudinal	Secondary data analysis	Registry data		Sample size, n (Time period)	Age (years)	Condition	Self-reported prevalence /proportion	Mortality	DALYs lost per 100,000	Other	
									Depressive disorder in persons **without** a “skin condition”	T1: 5.5%<br>T2: 5.2%				
7	Landis (2014)[7]	-	-	x	-	USA	Unclear	65 to 84	Any skin condition	-	-	-	Skin-related visits to specialties:<br>15% General practitioner<br>8-9% Internal Medicine	-
								≥85					Visits to leading specialties:<br>17% General practitioner<br>6% Internal Medicine	
8	Caretti (2015)[8]	x	-	-	-	USA	101	71 (median)	Any type of skin concern/complaint	69.3%	-	-	Most subjects reported multiple concerns	-
									Dry skin/pruritus	40.6%			17.8% would consult a physician about it	
									Moles	27.7%			-	
									Hair loss	25.7%				
									Skin discoloration	20.8%				
									Wrinkles	15.8%				
									Facial hair	13.9%				
									Rashes	13.9%			20.8% would consult a physician about it	
9	Hay (2015)[9]	-	-	x	-	Global	Unclear	70 to 74	NMSC	-	3.2	-	-	Global mortality rates
									Melanoma		5.8			
									Cellulitis		2.3			
									Other bacterial skin infection		3.0			

No.	First author (year)	Design				Geographic location	Sample characteristics		Results					Notes
		Cross-sectional	Longi-tudinal	Secondary data analysis	Registry data		Sample size, n (Time period)	Age (years)	Condition	Self-reported prevalence /proportion	Mortality	DALYs lost per 100,000	Other	
								75 to 79	NMSC		3.8			
									Melanoma		5.6			
									Cellulitis		2.3			
									Other bacterial skin infection		3.2			
								≥80	NMSC		12.6			
									Melanoma		10.9			
									Cellulitis		4.5			
									Other bacterial skin infection		7.4			
								60 to 64, ≥70	Eczema	-	-	M: 92.8 to 96.1 F: 113.8 to 116.2	-	Age group 65 to 69 not included in analysis
									Alopecia areata			M: 14.7 to 16.3 F: 24.0 to 26.5		
									Pruritus			M: 46.6 to 95.6 F: 62.0 to 131.4		
									Urticaria			M: 29.0 to 31.8 F: 42.7 to 49.2		
									Pressure injury			M: 45.1 to 211.2 F: 36.5 to 209.3		
									Psoriasis			M: 31.5 to 37.8 F: 25.8 to 30.9		
									Cellulitis			M: 33.5 to 55.1 F: 25.3 to 41.1		
									Abscess, impetigo			M: 45.1 to 73.7 F: 37.0 to 62.2		
									Scabies			M: 8.5 to 16.7 F: 7.4 to 16.1		
									Fungal skin disease			M: 44.8 to 56.6 F: 39.1 to 51.6		
									Viral skin disease			M: 21.4 to 21.8 F: 20.3 to 20.6		

**No.**	**First author (year)**	**Design**				**Geographic location**	**Sample characteristics**		**Results**					**Notes**
		Cross-sectional	Longi-tudinal	Secondary data analysis	Registry data		Sample size, n (Time period)	Age (years)	Condition	Self-reported prevalence /proportion	Mortality	DALYs lost per 100,000	Other	
									Other skin diseases			M: 194.5 to 358.2<br>F: 144.3 to 223.1		
10	Liu (2016)[10]	-	-	x	-	USA	388	≥ 65	Unknown	-	-	-	Urgent visits due to dermatological concerns 6%	-
11	Asokan (2017)[11]	x	-	-	-	Kerala/India	562	73.7 (mean)	Tingling sensation of extremities	37.4%	-	-	-	-
									Ache in the extremities	30.1%				
									Nonspecific itching	21.7%				
									Finger pebbling	14.4%				
									Burning sensation on the extremities	13.2%				
12	Hahnel (2017)[12]	-	-	x	-	Tunisia	Unclear	≥ 65	Itch/pruritus	6.4% (medical practices)	-	-	-	-
						Norway				13.8% (domesticity)				
						USA			"Sensitive skin"	16.0% (domesticity)				
13	Henchoz (2017)[13]	x	-	-	-	Switzerland	5,300	≥ 68	"Skin problems"	9.8% (weighted prevalence)	-	-	-	-
									Association between chronic symptom "skin problems" and unfavourable Quality of Life	-			OR 1.7 ($p < 0.05$) (Women, adjusted for age, canton of residence, main city, Swiss citizenship, living arrangement, children, education, financial difficulties	

No.	First author (year)	Design				Geographic location	Sample characteristics		Results					Notes
		Cross-sectional	Longi-tudinal	Secondary data analysis	Registry data		Sample size, n (Time period)	Age (years)	Condition	Self-reported prevalence /proportion	Mortality	DALYs lost per 100,000	Other	
													and depressive symptom)	
14	Karimkhani (2017a)[14]	-	-	x	-	Global	Unclear	60-64	Scabies	-	-	38.8	-	-
								65-69				40.0		
								70-74				42.8		
								75-79				52.3		
								≥80				46.4		
15	Karimkhani (2017b)[15]	-	-	x	-	Global	Unclear	≥ 60	Melanoma	-	-	30 to 50	-	DALYs extracted from figure 1
									Keratinocyte carcinoma			20 to 80 (increases with age)		
									Dermatitis			40 to 80 (decreases with age)		
									Psoriasis			40 to 70 (decreases with age)		
									Cellulitis			10 to 20 (increase with age)		
									Pyoderma			10 to 30 (increase with age)		
									Scabies			5 to 10 (decrease with age)		
									Fungal skin diseases			30 to 40		
									Viral skin diseases			10 to 20		
									Acne vulgaris			≤5		
									Alopecia areata			≤10		
									Pruritus			not visible in graph		
									Urticaria			50 to 90 (increases with age)		

**No.**	**First author (year)**	**Design**				**Geographic location**	**Sample characteristics**		**Results**					**Notes**
		Cross-sectional	Longi-tudinal	Secondary data analysis	Registry data		Sample size, n (Time period)	Age (years)	Condition	Self-reported prevalence /proportion	Mortality	DALYs lost per 100,000	Other	
									Pressure injury			10 to 100 (increases with age)		
									Other skin and subcutaneous diseases			60 to 70		
16	Lee (2017)[16]	-	-	x	-	South Korea	4,807	81.0 (mean)	Pressure injury	-	-	-	859 (17.9%) admissions to hospitals during the study period.<br><br>Home-care was associated with a lower risk of hospitalization related to pressure ulcers (odds ratio [OR] = 0.68, 95% confidence interval [CI] = 0.49–0.93; reference, no use).<br><br>Older patients had a lower risk of hospitalization (OR = 0.99, 95% CI = 0.98–1.00).	-
17	Aitken (2018)[17]	-	-	-	x	Australia (Queensland)	Unclear	≥ 60.0	Melanoma	-	Year 2014:<br><br>M: app. 40/100,000<br>F: app. 15/100,000	-	-	Rates extracted from figure 3 (log-scale)
18	Cowdell (2018)[18]	x	-	-	-	England	1116	81.1 (mean)	Itch	9.3%	-	-	Bothersome:<br>• A little 46.4%<br>• A lot 27.7%<br>• Very much 5.4%	-
									Dry skin	12.1%			• A little 58.8%<br>• A lot 11.5%	

No.	First author (year)	Design				Geographic location	Sample characteristics		Results					Notes
		Cross-sectional	Longi-tudinal	Secondary data analysis	Registry data		Sample size, n (Time period)	Age (years)	Condition	Self-reported prevalence /proportion	Mortality	DALYs lost per 100,000	Other	
													• Very much 4.1%	
									Inflammation	4.9%			• A little 44.8%<br>• A lot 27.7%<br>• Very much 1.5%	
									Leg or pressure ulcer	1.0%			-	
									Precancerous or cancerous lesion	1.5%				
									Skin infection	0.8%				
									Aged appearance	10.2%				
									Nail infection	2.2%				
									Abnormal hair loss	1.0%				
19	Hu (2018)[19]	-	-	-	x	China (Hong Kong)	807 cases (32 years)	≥ 65	Melanoma		1.8 /100,000 person-years		-	-
20	Lichterfeld-Kottner (2018)[20]	x	-	-	-	Germany	923	80.6 (mean)	Pruritus	19.5%	-	-	-	-
21	Meyers (2019)[21]	-	-	x	-	USA	Unclear	60 to 64	Any non-postherpetic neuralgia complication (herpes zoster)	-	-	-	8.9%	-
								65 to 69					9.7%	
								70 to 79					11.1%	
								≥ 80					13.3%	
									Neurologic complications (herpes zoster)				Proportion compared to aged 50–59: 2:1	

No.	First author (year)	Design				Geographic location	Sample characteristics		Results					Notes
		Cross-sectional	Longi-tudinal	Secondary data analysis	Registry data		Sample size, n (Time period)	Age (years)	Condition	Self-reported prevalence /proportion	Mortality	DALYs lost per 100,000	Other	
22	Sinikumpu (2020)[22]	x	-	-	-	Finland	552	78.4 (mean)	Any skin disease (excluding benign skin tumours and pattern hair loss)	-	-	-	• Self-treatment required in 32.6%<br>• Further care by physician required in 43.1%<br>• Presence of three or more skin diseases in 39.1%	-
23	Tseng (2020)[23]	-	-	x	-	USA	24,551 cases (3 months)	≥ 60	Herpes zoster - cutaneous complications	-	-	-	4.97 to 13.28% (confirmed)	Weighted proportions, among confirmed incident herpes zoster patients
									Herpes zoster - neurological complications				Up to 1.74% (confirmed)	
									Herpes zoster - other complications				Up to 2.95% (confirmed)	
									Herpes zoster - hospitalisation rate				9.74 to 39.57 /100,000 person-years<br>-<br>Rate is sharply increasing with age	-
24	Yong (2020)[24]	-	x	-	-	Malaysia	770	69.9 (median)	Generalised pruritus	6.0%	-	-	45.7% reported severe or very severe pruritus	-
									Association between Pittsburgh Sleep Quality Index (PSQI) and generalised pruritus	-			PSQI was associated with generalized pruritus (p = 0.04)	
									Association between General Health				No association	

No.	First author (year)	Design				Geographic location	Sample characteristics		Results					Notes
		Cross-sectional	Longi-tudinal	Secondary data analysis	Registry data		Sample size, n (Time period)	Age (years)	Condition	Self-reported prevalence /proportion	Mortality	DALYs lost per 100,000	Other	
									and generalised pruritus					
									Association between Psychological comorbidities and generalised pruritus				No association	
25	Bai (2021)[25]	-	-	x	-	China	Unclear	≥ 60	Melanoma	-	0.5 to 5 /100,000 person-years<br><br>Rate increases with age	-		Rates extracted from figure 3
26	Neena (2021)[26]	x	-	-	-	India	385	≥65	Pruritus	100%	-	-	-	Symptoms reported by subjects with eczema
									Scaling	50%				
									Oozing	31.8%				
									Dryness	29.5%				
									Thickening of skin lesion	18.2%				
27	Waldmann (2021)[27]	-	-	-	x	Germany	Unclear	≥ 65	Melanoma	-	M: 5 to 20 /100,000 males/year<br>F: 10 to 35 /100,000 females/year	-	-	Rate interpreted from figure 3
28	Blazek (2022)[28]	-	-	-	x	Australia	Unclear	≥ 60 (26 years)	Melanoma	-	Year 2014:<br>F: 15/100,000<br>M: 30 to 35/100,000 persons	-	-	Rate extracted from figure 2
29	Raghuwanshi (2022)[29]	x	-	-	-	India	1,000	60-74	Pruritus	15.2%	-	-	-	-
								≥75		19.2%				
30	Rodriguez-Betancourt (2022)[30]	-	-	-	x	Colombia	42 cases (Ten years)	≥70	Melanoma	-	43.9% (2 / 5-year survival rate	-	-	-

**No.**	**First author (year)**	**Design**				**Geographic location**	**Sample characteristics**		**Results**					**Notes**
		Cross-sectional	Longi-tudinal	Secondary data analysis	Registry data		Sample size, n (Time period)	Age (years)	Condition	Self-reported prevalence /proportion	Mortality	DALYs lost per 100,000	Other	
											64.6 / 56.4)			

Abbreviations:

Non-melanoma skin cancer (NMSC)

Table S7: Summary of included studies for review question 3

No.	First author (year)	Skin disease	Type of evidence		Geographic location	Sample characteristics		Type of Intervention				Outcomes
			Descriptive	Interventional		Sample size, n	Age in years	Screening/ diagnoses	Risk assessment	Prevention	Treatment	
1	Bonaccorsi (2013)[31]	Pressure injury (“cutaneous lesions due to maceration”)	-	x	Italy	377	85.4 (mean)	-	-	x (different pads)	-	• Pressure ulcer incidence<br>• Subjective nurse evaluation of skin condition (moisture, maceration, desquamation, scratching lesions)<br>• Compliance (patient and caregiver opinion on pads regarding quality of life, burden of care, technical aspects)
2	Okuno (2013)[32]	Herpes Zoster	-	x	Japan	5,683	≥ 60	-	x (prick test)	-	-	• Erythema diameter<br>• Oedema diameter<br>• Herpes zoster incidence rate
3	Trautmann (2016)[33]	Skin cancer (Melanoma and NMSC)	x	-	Germany	686,899	≥ 60	x	-	-	-	• Melanoma incidence<br>• Non-melanoma skin cancer incidence<br>• Screening participation<br>• Disease severity of cases (metastasis and/or interferon alpha treatment)
4	Alexandridou (2017)[34]	Herpes Zoster	x	-	UK	127,467	≥ 70	-	-	x (vaccination)	-	• Herpes zoster, post-herpetic neuralgia, other herpes zoster complications<br>• Vaccine effectiveness/herpes zoster incidence? (first two years)<br>• Vaccine effectiveness/ post-herpetic neuralgia incidence? (first two years)<br>• Vaccine effectiveness/herpes zoster incidence? (2+ years)
5	Aitken (2018)[17]	Melanoma	x	-	Australia (Queensland)	Unclear	≥ 60	x	-	x (education campaigns)	-	• Melanoma incidence (trends 1995 – 2014)<br>• Melanoma mortality(trends 1995 – 2014)

No.	First author (year)	Skin disease	Type of evidence		Geographic location	Sample characteristics		Type of Intervention				Outcomes
			Descriptive	Interventional		Sample size, n	Age in years	Screening/ diagnoses (early detection campaigns)	Risk assessment	Prevention	Treatment	
6	d. Silva (2019)[35]	Skin cancer (not specified)	x	-	Brazil	315	≥60	-	-	x (sun screen use)	-	• Non-use of sunscreen
7	Bianchi (2020)[36]	Various skin lesions	-	x	Brazil	6,320	≥60	x (Teledermatology)	-	-	x	• Referral to primary care physician<br>• Referral to consultation with dermatologist<br>• Referral to biopsy followed by in-presence dermatologist appointment with result<br>• Most common prescriptions by teledermatologists<br>• Mean waiting time for face-to-face consultation with dermatologist
8	Chang (2020)[37]	Skin cancer (Not specified)	x	-	USA	Unclear	≥70	-	-	X (sun protection)	-	• Prevalence of sun protective behavior and any individual behaviour (wearing sun-protective clothing, shade use, hat use, sunscreen use, infrequent indoor tanning device use)
9	Prasad (2020)[38]	Pressure injury	-	x	UAE	249	75.5 (mean)	x	x	x	-	• Prevalence pressure injuries before/after protocol implementation<br>• Incidence pressure injuries before/after protocol implementation<br>• Protocol compliance
10	Sideris (2020)[39]	Skin cancer (Melanoma and NMSC)	x	-	Australia	Unclear	>60	-	-	x (sun protection)	-	• Knowledge/awareness of skin cancer (knowledge of skin cancer types, warning signs, risk factors)

No.	First author (year)	Skin disease	Type of evidence		Geographic location	Sample characteristics		Type of Intervention				Outcomes
			Descriptive	Interventional		Sample size, n	Age in years	Screening/ diagnoses	Risk assessment	Prevention	Treatment	
												• Risky/protective sun behaviour
11	Neena (2021)[26]	Eczema	x	-	India	385	≥65	X (by health workers)	-	-	-	• Diagnostic accuracy of diagnoses made by health care workers
12	Blazek (2022)[28]	Skin cancer (Melanoma)	x	-	Australia	69,136	≥60	-	-	X (skin cancer awareness/ education campaigns)	-	• Melanoma incidence (trends since late 80s (invasive)/ early 2000s (in situ))
13	Matsumoto (2022)[40]	Skin cancer (Melanoma)	x	-	USA	175,380	≥65	x	-	-	-	• Melanoma incidence screened vs. unscreened<br>• Incidence melanoma in situ screened vs. unscreened<br>• Incidence melanoma ≤1mm/>1mm/>2mm/>4mm screened vs. unscreened
14	Navsaria (2022)[41]	AK	x	-	USA	4,999,999	≥65	-	-	-	x	• Most common types of treatments (destruction treatment, topical medication, photodynamic therapy)

Abbreviations:

Non-melanoma skin cancer (NMSC)

REFERENCES

1. Frese T, Herrmann K, Sandholzer H. Pruritus as reason for encounter in general practice. *J Clin Med Res* 2011; **3**:223-9.
2. Hollestein LM, van den Akker SA, Nijsten T et al. Trends of cutaneous melanoma in The Netherlands: increasing incidence rates among all Breslow thickness categories and rising mortality rates since 1989. *Ann Oncol* 2012; **23**:524-30.
3. Joly P, Baricault S, Sparsa A et al. Incidence and mortality of bullous pemphigoid in France. *J Invest Dermatol* 2012; **132**:1998-2004.
4. Wysong A, Linos E, Hernandez-Boussard T et al. Nonmelanoma skin cancer visits and procedure patterns in a nationally representative sample: national ambulatory medical care survey 1995-2007. *Dermatol Surg* 2013; **39**:596-602.
5. Gontijo Guerra S, Vasiliadis HM, Preville M, Berbiche D. Skin conditions in community-living older adults: prevalence and characteristics of medical care service use. *J Cutan Med Surg* 2014; **18**:186-94.
6. Gontijo Guerra S, Preville M, Vasiliadis HM, Berbiche D. Association between skin conditions and depressive disorders in community-dwelling older adults. *J Cutan Med Surg* 2014; **18**:256-64.
7. Landis ET, Davis SA, Taheri A, Feldman SR. Top dermatologic diagnoses by age. *Dermatol Online J* 2014; **20**:22368.
8. Caretti KL, Mehregan DR, Mehregan DA. A survey of self-reported skin disease in the elderly African-American population. *Int J Dermatol* 2015; **54**:1034-8.
9. Hay RJ, Fuller LC. Global burden of skin disease in the elderly: a grand challenge to skin health. *G Ital Dermatol Venereol* 2015; **150**:693-8.
10. Liu T, Brienza R. What brings an older veteran to an urgent visit (UV) A review of the chief concerns by veterans aged 65 and older who presented for uv durin a 6-month period at the West Haven Veteran Affairs Center of excellence in Primary Care Education (VA COEPCE), an interprofessional academic patient aligned care team (PACT) [abstract]. In: Abstracts from the 2016 Society of General Internal Medicine Annual Meeting. *J Gen Intern Med* 2016; 31(Suppl 2):S468-9. doi: 10.1007/s11606-016-3657-7. Available from: https://link.springer.com/article/10.1007/s11606-016-3657-7. [accessed 02.05.2023].
11. Asokan N, Binesh VG. Cutaneous problems in elderly diabetics: A population-based comparative cross-sectional survey. *Indian J Dermatol Venereol Leprol* 2017; **83**:205-11.
12. Hahnel E, Lichterfeld A, Blume-Peytavi U, Kottner J. The epidemiology of skin conditions in the aged: A systematic review. *J Tissue Viability* 2017; **26**:20-8.
13. Henchoz Y, Bula C, Guessous I et al. Chronic symptoms in a representative sample of community-dwelling older people: a cross-sectional study in Switzerland. *BMJ Open* 2017; **7**:e014485.
14. Karimkhani C, Colombara DV, Drucker AM et al. The global burden of scabies: a cross-sectional analysis from the Global Burden of Disease Study 2015. *Lancet Infect Dis* 2017; **17**:1247-54.
15. Karimkhani C, Dellavalle RP, Coffeng LE et al. Global Skin Disease Morbidity and Mortality: An Update From the Global Burden of Disease Study 2013. *JAMA Dermatol* 2017; **153**:406-12.
16. Lee HJ, Ju YJ, Park EC et al. Effects of home-visit nursing services on hospitalization in the elderly with pressure ulcers: a longitudinal study. *Eur J Public Health* 2017; **27**:822-6.
17. Aitken JF, Youlden DR, Baade PD et al. Generational shift in melanoma incidence and mortality in Queensland, Australia, 1995-2014. *Int J Cancer* 2018; **142**:1528-35.
18. Cowdell F, Dyson J, Long J, Macleod U. Self-reported skin concerns: An epidemiological study of community-dwelling older people. *Int J Older People Nurs* 2018; **13**:e12195.
19. Hu L, Jin S, Chen L, Wang Y. Trends in the incidence and mortality of cutaneous melanoma in Hong Kong between 1983 and 2015. *Int J Clin Exp Med* 2018; **11**:8259-66.
20. Lichterfeld-Kottner A, Lahmann N, Blume-Peytavi U et al. Dry skin in home care: A representative prevalence study. *J Tissue Viability* 2018; **27**:226-31.

21. Meyers JL, Candrilli SD, Rausch DA et al. Costs of herpes zoster complications in older adults: A cohort study of US claims database. *Vaccine* 2019; **37**:1235-44.
22. Sinikumpu SP, Jokelainen J, Haarala AK et al. The High Prevalence of Skin Diseases in Adults Aged 70 and Older. *J Am Geriatr Soc* 2020; **68**:2565-71.
23. Tseng HF, Bruxvoort K, Ackerson B et al. The Epidemiology of Herpes Zoster in Immunocompetent, Unvaccinated Adults >/=50 Years Old: Incidence, Complications, Hospitalization, Mortality, and Recurrence. *J Infect Dis* 2020; **222**:798-806.
24. Yong SS, Kwan Z, Ch'ng CC et al. Self-reported generalised pruritus among community-dwelling older adults in Malaysia. *BMC Geriatr* 2020; **20**:223.
25. Bai R, Huang H, Li M, Chu M. Temporal Trends in the Incidence and Mortality of Skin Malignant Melanoma in China from 1990 to 2019. *J Oncol* 2021; **2021**:9989824.
26. Neena V, Asokan N, Jose R, Sarin A. Prevalence of eczema among older persons: A population-based cross-sectional study. *Indian J Dermatol Venereol Leprol* 2021; **89**:426-30.
27. Waldmann A, Pritzkuleit R, Labohm L, Katalinic A. Epidemiologie von Krebs im hohen Lebensalter. *best practice onkologie* 2021; **16**:586-97.
28. Blazek K, Furestad E, Ryan D et al. The impact of skin cancer prevention efforts in New South Wales, Australia: Generational trends in melanoma incidence and mortality. *Cancer Epidemiol* 2022; **81**:102263.
29. Raghuwanshi AS, Diwan S, Singh H, Raghuwanshi KC. A Cross-Sectional Study to Assess the Psychosocial impact of Skin Diseases. *International Journal of Pharmaceutical and Clinical Research* 2022; **14**:1061-7.
30. Rodriguez-Betancourt JD, Arias-Ortiz N. Cutaneous melanoma incidence, mortality, and survival in Manizales, Colombia: a population-based study. *J Int Med Res* 2022; **50**:3000605221106706.
31. Bonaccorsi G, Lorini C, Santomauro F et al. 202 Impact of different pads in elderly assisted in home care [abstract]. In: 43rd Annual Meeting of the International Continence Society, ICS 2013, Barcelona, Spain. *Neurourol. Urodyn.* 2013:802-3. doi: 10.1002/nau.22472. Available from: https://onlinelibrary.wiley.com/doi/10.1002/nau.22472. [accessed 02.05.2023].
32. Okuno Y, Takao Y, Miyazaki Y et al. Assessment of skin test with varicella-zoster virus antigen for predicting the risk of herpes zoster. *Epidemiol Infect* 2013; **141**:706-13.
33. Trautmann F, Meier F, Seidler A, Schmitt J. Effects of the German skin cancer screening programme on melanoma incidence and indicators of disease severity. *Br J Dermatol* 2016; **175**:912-9.
34. Alexandridou M, Bollaerts K. Pin10 Zoster Vaccine Effectiveness against incident herpes zoster and post-herpetic neuralgia in elderly in the UK [abstract]. In: ISPOR 20th Annual European Congress, Glasgow, United Kingdom. *Value Health* 2017; 20:A780. doi: 10.1016/j.jval.2017.08.2263. Available from: https://www.sciencedirect.com/science/article/pii/S1098301517325974?via%3Dihub. [accessed 26.04.2023].
35. Silva ESD, Dumith SC. Non-use of sunscreen among adults and the elderly in southern Brazil. *An Bras Dermatol* 2019; **94**:567-73.
36. Bianchi M, Santos A, Cordioli E. Benefits of Teledermatology for Geriatric Patients: Population-Based Cross-Sectional Study. *J Med Internet Res* 2020; **22**:e16700.
37. Chang C-Y, Park H, Lo-Ciganic J. 3946 | The prevalence of sun protective behaviors across different age groups in the US population: Findings from the 2015 US Health Interview Survey [abstract]. In: Special Issue: Abstracts of the 36th International Conference on Pharmacoepidemiology & Therapeutic Risk Management, Virtual. *Pharmacoepidemiol Drug Saf* 2020; 29(Suppl 3):306-7. doi: 10.1002/pds.5114. Available from: https://onlinelibrary.wiley.com/doi/10.1002/pds.5114. [accessed 02.05.2023].
38. Prasad S, Hussain N, Sharma S et al. Impact of Pressure Injury Prevention Protocol in Home Care Services on the Prevalence of Pressure Injuries in the Dubai Community. *Dubai Medical Journal* 2020; **3**:99-104.

39. Sideris E, Thomas SJ. Patients' sun practices, perceptions of skin cancer and their risk of skin cancer in rural Australia. *Health Promot J Austr* 2020; **31**:84-92.
40. Matsumoto M, Wack S, Weinstock MA et al. Five-Year Outcomes of a Melanoma Screening Initiative in a Large Health Care System. *JAMA Dermatol* 2022; **158**:504-12.
41. Navsaria L, Li Y, Nowakowska M et al. LB911 Incidence and treatments of actinic keratosis in the Medicare population: A cohort study [abstract]. In: Society for Investigative Dermatology (SID) 2022 Meeting, Portland, United States. *Journal of Investigative Dermatology* 2022; 142:B10. doi: 10.1016/j.jid.2022.05.929. Available from: https://www.sciencedirect.com/science/article/pii/S0022202X22013707?via%3Dihub. [accessed 20.04.2023].
